# *De Novo* Enantioselective Synthesis of Hexafluorinated d-Glucose

**DOI:** 10.1021/acs.joc.4c01724

**Published:** 2024-09-13

**Authors:** Sébastien Depienne, Clément Q. Fontenelle, Mark E. Light, Kristof Van Hecke, Bruno Linclau

**Affiliations:** † Department of Organic and Macromolecular Chemistry, Ghent University, Campus Sterre, Krijgslaan 281-S4, 9000 Ghent, Belgium; ‡ School of Chemistry, University of Southampton, Highfield, Southampton SO17 1BJ, U.K.; § Department of Chemistry, Ghent University, Campus Sterre, Krijgslaan 281-S3, 9000 Ghent, Belgium

## Abstract

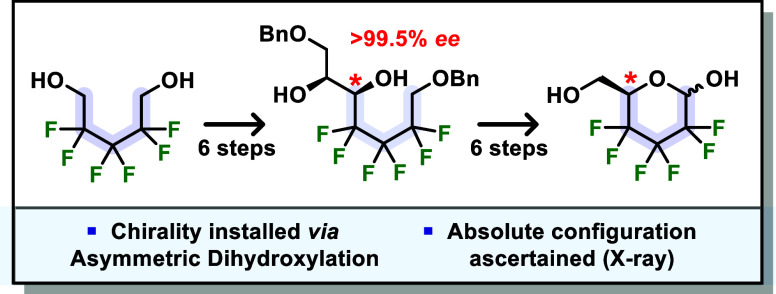

We report a *de novo* enantioselective synthesis of 2,3,4-trideoxy-2,2,3,3,4,4-hexafluoro-d-*glycero*-hexopyranose (hexafluorinated d-glucose), an iconic polar hydrophobic glycomimetic. The 12-step synthesis features robust and reproducible chemistry and was achieved by incorporating an asymmetric dihydroxylation step to install the stereogenic center with excellent enantioselectivity (95:5 *er*). Virtual enantiopurity (>99.5% *ee*) was further reached using a simple crystallization procedure and the absolute confirmation was ascertained by X-ray analysis. The synthetic route also allowed access to the novel hexafluorinated heptose derivative 2,3,4-trideoxy-2,2,3,3,4,4-hexafluoro-l-*threo*-heptopyranose.

## Introduction

**Chart 1:**
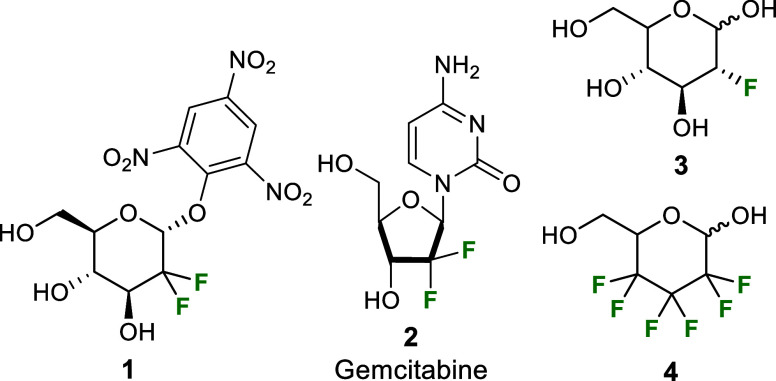
Examples of fluorinated carbohydrates used as drugs or probes

Carbohydrates are ubiquitous biomolecules widely involved in complex biological and pathogenic events (e.g., immunity, inflammation, and host–pathogen interactions).^1,2^ Consequently, both a deeper understanding and a precise control over these processes are of particular interest in chemical biology and in the pharmaceutical industry.^3^ However, the development of carbohydrate-based probes or therapeutics have been hampered by the inherent poor enzymatic stability and limited pharmacokinetics of native sugars, as well as low binding affinities which mainly originates from their highly hydrophilic character.^4,5^ To overcome these limitations, deoxyfluorination is a prevalent structural modification strategically employed to improve their metabolic and hydrolytic stability, and their physicochemical properties.^6−9^ Fluorosugars such as the glycosidase inhibitor **1** and the approved drug gemcitabine **2** (Chart 1) are relevant examples of successful mechanism-based inhibitors and anticancer therapeutics, respectively. Additionally, introducing a fluorine atom in carbohydrates has been used to investigate biomolecular interactions (*e.g*. lectin-carbohydrate recognition, transport across cell membrane, and epitope mapping) via ^19^F NMR techniques,^10−12^ and to detect and diagnose pathological events in vivo using ^18^F glycoprobes such as the well-known tracer 2-[^18^F]fluoro-2-deoxy-d-glucose **3** visualized by positron emission tomography.^13,14^ Interestingly, sugar fluorination has also been central in the recent successful preparation of a fluorodisialoside glycomimetic validated as a vaccine lead against *meningitis* B and C when conjugated to protein carriers (fluoroglycovaccines).^15^

Pioneering work from DiMagno and coworkers proposed that extensive fluorination of carbohydrates could substantially enhance binding affinities based on the “polar hydrophobicity” principle.^11,16^ This principle posits that the strongly polarized C–F bonds are able to interact favorably with cationic/dipolar sites in receptors, while the fluorine atoms collectively provide a large hydrophobic surface area, leading to a significant enthalpic aqueous desolvation benefit. To this extent, racemic 2,3,4-trideoxy-2,2,3,3,4,4-hexafluoro-*glycero*-hexopyranose **4** (Chart 1)^11^ was shown to cross erythrocyte membranes with a 10-fold higher rate as compared to d-glucose. Transmembrane transport of glucose is known to be mediated by membrane proteins transporters such as GLUT1,^17,18^ and the increased rate observed with **4** was attributed to an enhanced affinity to GLUT1. Sugars in which the C2–C4 hydroxyl groups were changed by single fluorine atoms have also been synthesized,^10,19,20^ with 2,3,4-trideoxy-2,3,4-trifluoro-d-glucose shown to also cross erythrocyte membranes, at a slightly lower rate as 3-deoxy-3-fluoro-d-glucose.^10^ Given the ubiquity of carbohydrate-mediated biological processes, (poly)fluorinated polar-hydrophobic glycomimetics such as **4** hold great potential as substrates or inhibitors of carbohydrate-processing/binding proteins, enabling unique avenues to the design and optimization of bioactive probes and pharmaceutically relevant compounds.^21^

**Scheme 1:**
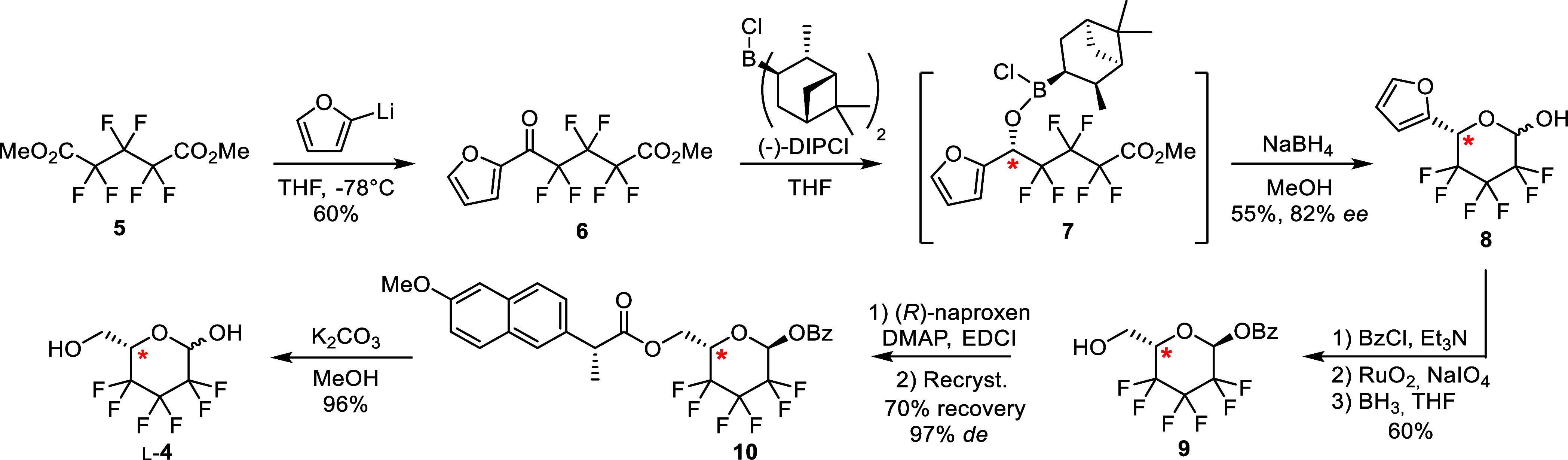
Reported Enantioselective Synthesis of 2,3,4-Trideoxy-2,2,3,3,4,4-hexafluoro-l-hexose (l-**4**)^16^

Our group has a particular interest in developing syntheses of polyfluorinated carbohydrates, as well as in the evaluation of their physical and biological properties,^22−26^ and we required access to the hexafluorinated carbohydrate derivative **4** as its d-enantiomer. The only reported enantioselective synthesis of **4**, as its l-enantiomer, is shown in Scheme 1.^16^ The commercially available dimethyl hexafluoroglutarate **5** was converted to the furyl ketone **6**, which upon enantioselective reduction provides the l-pyranose ring **8** in 82% *ee*. After anomeric protection, the furan moiety was oxidized to carboxylic acid, followed by reduction to the hydroxymethyl group to give **9**. Enantiomeric enrichment was achieved by a resolution process based on crystallization of naproxen ester **10**, leading to an enantiomeric excess of 97%. Final transesterification then gave the reducing hexafluorinated sugar derivative l-**4**. The report mentioned that d-**4** could be accessed with the same strategy using (+)-DIPCl for the reduction of **6**, and by performing the resolution process with (*S*)-naproxen. Experimental details are unfortunately not described, and during attempts to prepare the d-sugar, the enantioselective reduction of **6** proved to be particularly challenging. Additionally, the naproxen derivative required for enantiomeric enrichment is expensive, and its recycling is hampered by potential enantiopurity erosion during final deprotection of the diester **10** in basic conditions and is rather laborious due to its required separation from methyl benzoate.

Here we present an alternative *de novo* enantioselective synthesis of d-**4**. Installation of the chiral center was based on Sharpless asymmetric dihydroxylation (SAD) methodology, which has previously been reliably used in *de novo* sugar synthesis.^27−29^ The obtained absolute configuration was confirmed by X-ray analysis. Additionally, the synthesis allowed access to a novel hexafluorinated heptose derivative.

## Results and Discussion

**Scheme 2:**
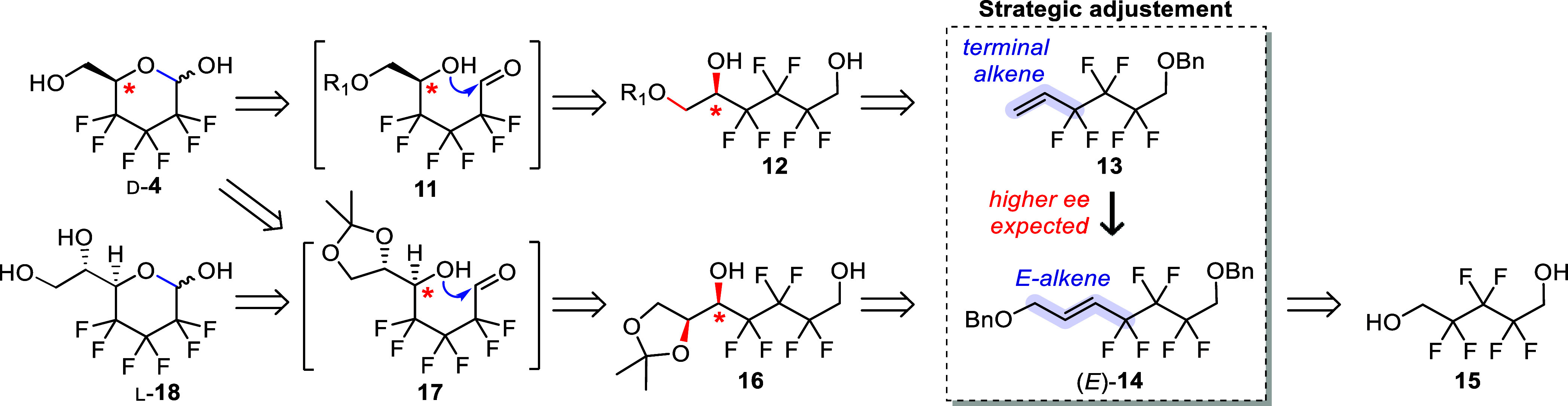
Retrosynthetic Considerations to Access d-**4**, Based on Asymmetric Dihydroxylation of a Disubstituted Alkene The heptose l-**18** would also be accessible from **17**.

Retrosynthetically (Scheme 2), the pyranose scaffold of d-**4** was envisioned to arise from the spontaneous cyclization of the open-chain ∂-hydroxyaldehyde **11**, in turn formed upon primary alcohol oxidation of the key monoprotected chiral triol **12**. The vicinal diol group was planned to be installed by dihydroxylation, leading to the terminal alkene **13** as the substrate. Asymmetric dihydroxylation of perfluoroalkyl-substituted deactivated alkenes has previously been studied by our group and was shown to proceed in excellent yields when enhanced levels of OsO_4_ are used. However, only moderate enantioselectivities (∼80% *ee*) were achievable when working with terminal alkene derivatives similar to **13**, and resolving agents such as naproxen would then have to be also considered here to enhance the enantiopurity level.^30^ Hence, an approach involving an *E*-configured disubstituted alkene such as (*E*)-**14** was envisaged. At a small cost of atom-economy, asymmetric dihydroxylation of (*E*)-**14** is expected to result in much higher enantioselectivity, as previously demonstrated on related tetrafluorinated substrates.^31,32^ The disubstituted alkene would be obtained from commercially available hexafluorodiol **15** using a Wittig olefination strategy. This approach thus requires C–C bond cleavage to eventually eliminate the extra carbon atom, for which a one-pot diol cleavage/aldehyde reduction sequence was envisioned. Hence, acetonide **16** would be the substrate for the oxidation/pyranose ring formation sequence. Additionally, this synthetic strategy allows access to the corresponding novel hexafluorinated heptose analogue l-**18**. Heptose sugars are essential components in Gram-negative bacteria, with interruption of their biosynthesis being regarded as an attractive avenue in the development of antibiotics.^33,34^

**Scheme 3:**
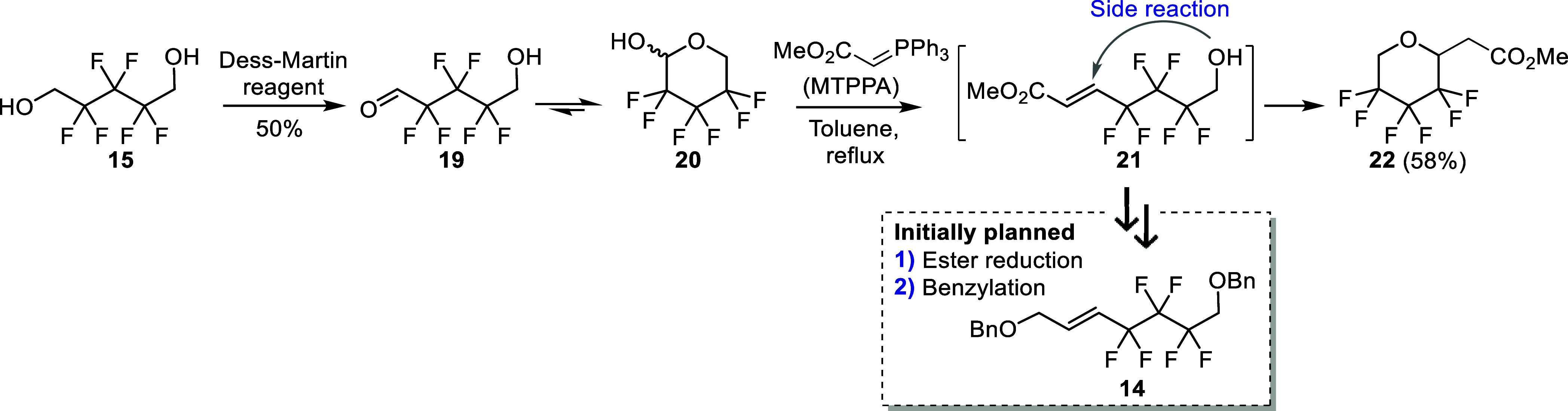
Unsuccessful Synthesis of the Olefin (*E*)-**14** by Initial Oxidative Desymmetrization of **15**

Our efforts started with the synthesis of the olefin **14** (Scheme 3). First, oxidative desymmetrization was performed on diol **15**, expecting that the intermediate aldehyde **19** would spontaneously cyclize to afford the more stable lactol **20**, thereby preventing oxidation of the second hydroxyl group. Dess–Martin periodinane (DMP) reagent proved efficient to form the hemiacetal **20** as observed by ^1^H NMR (1H at 5.14 ppm instead of the typical 10–11 ppm formyl signal, 1x OH visible in DMSO-*d*_6_) and ^13^C NMR analysis (1C at 90.9 ppm instead of the deshielded carbonyl signal at 190–200 ppm). Nonetheless, the hemiacetal was found to be volatile, which resulted in moderate isolated yields (∼50% average yield over six experiments). Subsequent olefination of **20** with methyl triphenylphosphoranylidene acetate (MTPPA) was successful, although the formed **21** underwent spontaneous cyclization with the liberated primary alcohol group. The reaction thus irreversibly afforded the Michael adduct **22** (efforts toward retro-Michael ring opening were unsuccessful, not shown), precluding to access (*E*)-**14** in this way.

**Scheme 4:**
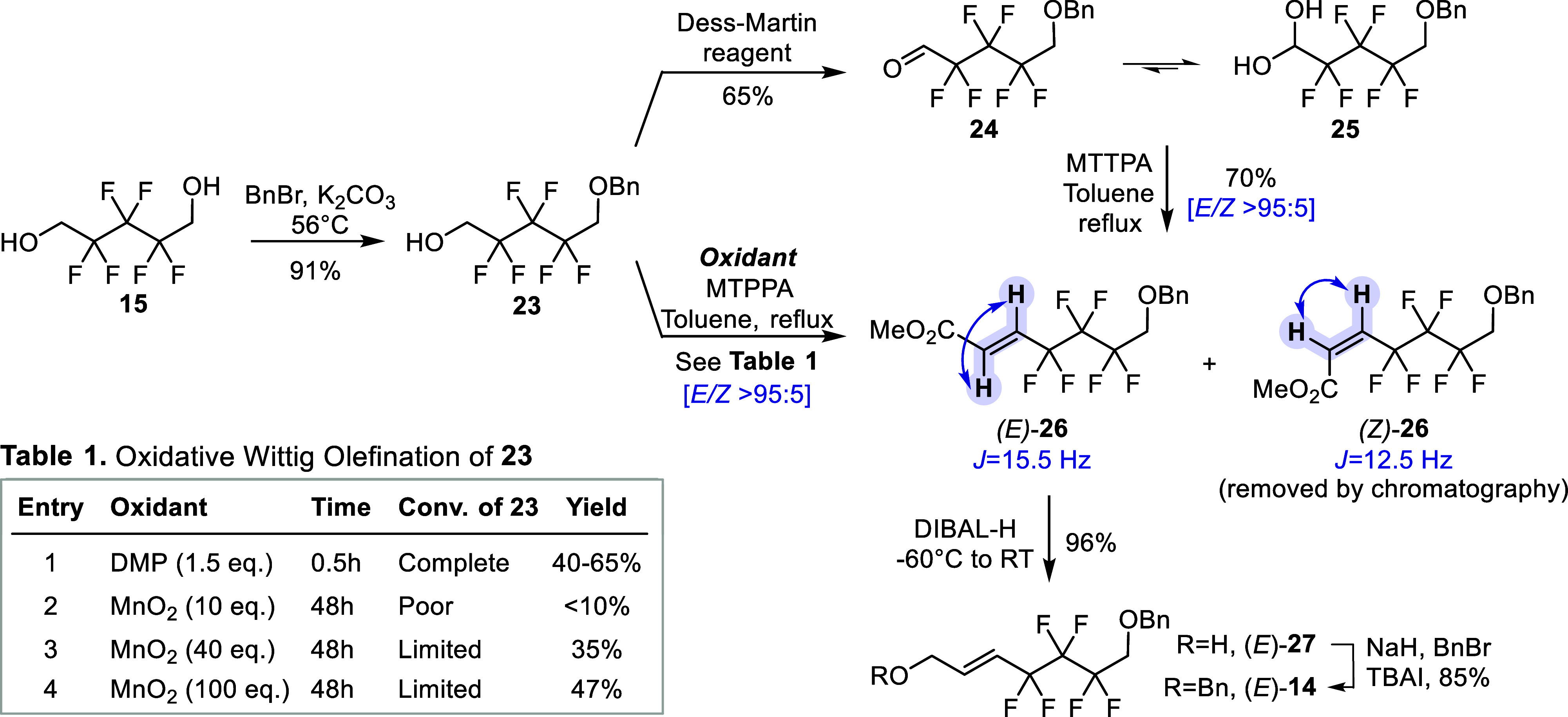
Efforts toward the Synthesis of the Olefin (*E*)-**14** by Initial Monoprotection of **15**

To prevent the undesired intramolecular side reaction observed with **21**, desymmetrization of the diol **15** by monobenzylation was required prior to the oxidation-olefination sequence (Scheme 4). Mild O-alkylation conditions using potassium carbonate in refluxing acetone reliably provided the benzyl ether **23** at the decagram scale with limited dibenzylation. With **23** in hand, the oxidation–olefination sequence was first attempted in a stepwise manner where the intermediate aldehyde was isolated after action of DMP. The pure oxidized compound could be obtained in up to 65% yield, even though the enhanced electrophilicity of the perfluoroalkyl substituted aldehyde **24** resulted in an unavoidable equilibrium with its hydrate **25**, as observed by ^1^H NMR (1H at 5.12 ppm instead of 10–11 ppm for formyl signal, 2× OH visible in DMSO-*d*_6_) and ^13^C NMR (1C at 86.3 ppm instead of 190–200 ppm). The hydrate was then reacted with MTPPA to successfully afford the disubstituted alkene **26** in good yield and with excellent selectivity (*E*/*Z* > 95:5, easily separable by normal phase silica gel chromatography). ^1^H NMR analysis unambiguously confirmed each configuration, with clearly identified ethylenic vicinal H–H coupling constants of *J* = 15.5 Hz for (*E*)-**26** and *J* = 12.5 Hz for (*Z*)-**26**. As the efficiency of the sequence may be hampered by intermediate aldehyde equilibration to the hydrate when isolated, we evaluated the possibility to access (*E*)-**26** in a one-pot procedure from alcohol **23**. Once TLC-analysis indicated completion of oxidation with DMP, MTPPA was directly added to the mixture followed by thermal activation (see inset Table 1, entry 1). This enabled olefination in suitable isolated yields ranging from 40 to 65%, but this reaction was limited by the notoriously tedious removal of byproducts from DMP and of phosphorus derivatives when performed on multigram scale. In this regard, manganese dioxide is a potentially more convenient oxidant for this oxidation–olefination sequence, as it is far easier to remove from the mixture by simple filtration. Even though MnO_2_ is classically used to oxidize activated alcohols (e.g., allylic, benzylic), Taylor et al. reported its efficient usage on nonactivated alcohols when in situ thermally displaced by a tandem Wittig olefination.^35^ Nonetheless, when **23** was reacted in refluxing toluene during more than 2 days, the primary alcohol could only be partially oxidized despite increasing the amount of MnO_2_ (as monitored by thin-layer chromatography (TLC)) and NMR monitoring indicated large remaining of **23** in each cases, as seen in inset Table 1 entries 2, 3, and 4 (Scheme 4). The strong electron-withdrawing effect of the geminal perfluoroalkyl is arguably too deactivating for the alcohol to be efficiently oxidized by mild manganese dioxide. That being said, a reasonable 47% yield could be achieved using a large excess (100 equiv) of MnO_2_. Considering the cheapness of the latter, the experimental simplicity, and the fact that unreacted **23** can easily be recovered and recycled, the manganese dioxide-mediated oxidative olefination offers a convenient alternative to access (*E*)-**26**, especially on larger scale experiments. Asymmetric dihydroxylation attempts of (*E*)-**26**—which is typically performed in basic aqueous conditions—led to a large amount of saponification (not shown). Hence, (*E*)-**26** was efficiently reduced to the corresponding allylic alcohol (*E*)-**27** using an excess of DIBAL-H. The resulting free hydroxyl group was further protected by conventional Williamson ether synthesis using sodium hydride as base and tetrabutylammonium iodide (TBAI) as catalyst to give the key *O*-benzylated (*E*)-**14** as a suitable substrate for the asymmetric dihydroxylation.

**Scheme 5:**
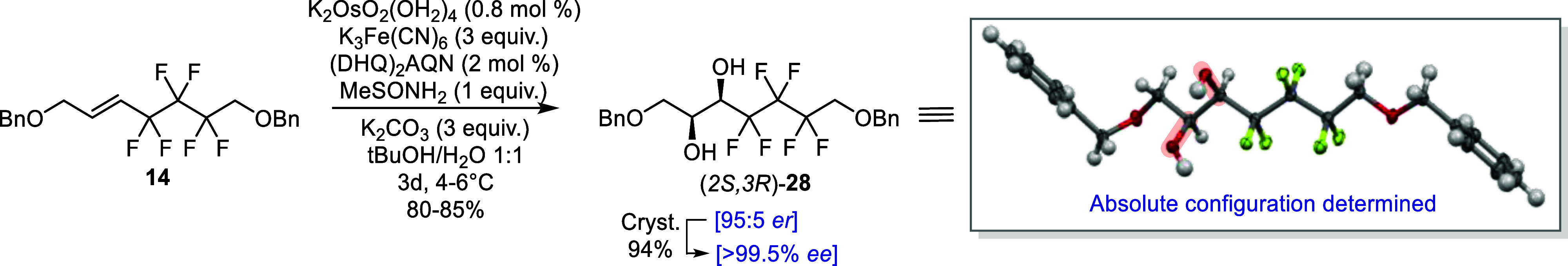
SAD Reaction of Olefin **14**

The required facial selection for the asymmetric dihydroxylation necessitated the use of a dihydroquinine-based (DHQ) chiral ligand (Scheme 5). As previously established with perfluoroalkyl-substituted deactivated alkenes, the anthraquinone spacer was chosen for best enantioselectivity results, and moderately higher amounts (0.8 mol % of potassium osmate and 2 mol % of dihydroquinine ligand (DHQ)_2_AQN)) were used to promote conversion.^36,37^ Potassium ferricyanide was introduced as stoichiometric regenerative co-oxidant, with methanesulfonamide as additive to increase the rate of the reaction by accelerating the basic hydrolysis step of the ester osmylate intermediate.^38^ The dihydroxylation of (*E*)-**14** required 3 days at 4–6 °C to give 80–85% isolated yield of **28**. The chiral diol could be reproducibly accessed (*n* = 3, including multigram scale experiments) with high enantioselectivity (95:5 *er*). Crystallization of **28** (2 g scale, 94% recovery) was further achieved by slow evaporation of a hexane/Et_2_O solution, which allowed to reach virtual enantiopurity (>99.5% *ee*) (Figure S2). The expected absolute configuration of the stereogenic centers in (2*S*,3*R*)-**28** could be unambiguously confirmed by X-ray crystallographic analysis.

**Scheme 6:**
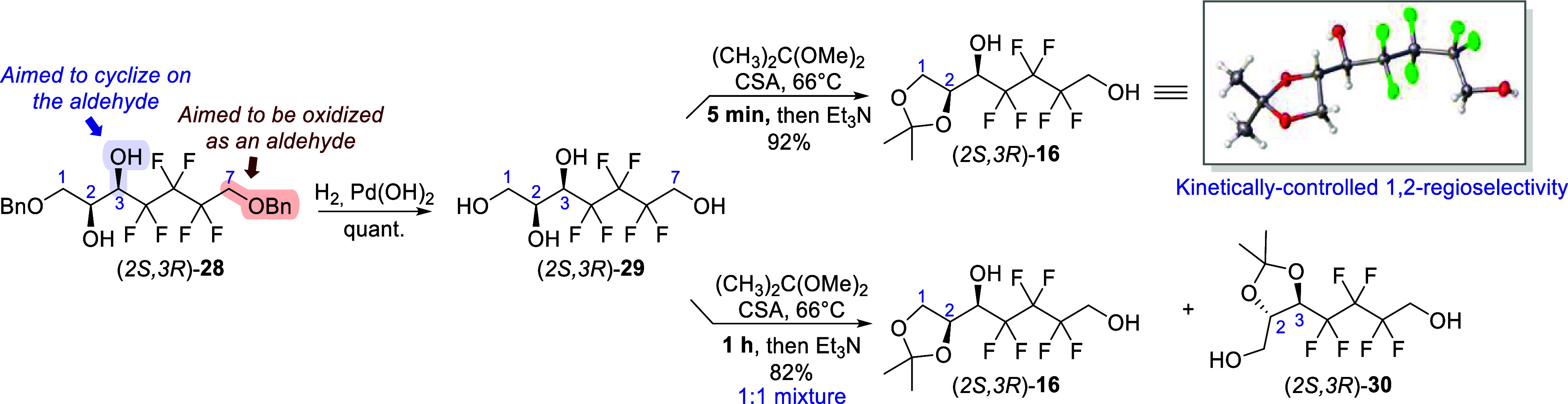
Kinetically Controlled Regioselective Protection of the Terminal Diol

Next, we elected to only have 3,7-diol available to ensure the selective oxidation of the primary alcohol and the formation of the heptopyranose ring with limited side reactions. To achieve this, a strategy to selectively protect the more reactive terminal 1,2-diol unit was required (Scheme 6). Hence, hydrogenolysis of (2*S*,3*R*)- **28** was first conducted to release both primary alcohols, which quantitatively afforded 1,2,3,7-tetraol (2*S*,3*R*)-**29**. Then, the latter was subjected to a kinetically controlled regioselective protection of the terminal diol unit using a procedure developed by our group.^39^ Camphor sulfonic acid was added to a refluxing mixture of dimethoxypropane and substrate, followed by rapid quenching with triethylamine. Virtually complete conversion of (2*S*,3*R*)-**29** could be achieved in less than 5 min—up to 10 min at multigram scale—with unique formation of the terminal acetonide (2*S*,3*R*)-**16** in excellent yield (92%). The 1,2-acetalization was unambiguously confirmed by NMR (Figures S3 and S5) as well as X-ray single crystal analysis. The regioselectivity was remarkable, given the acetonide of an internal *trans* disubstitued diol is expected to have a higher thermodynamic stability.^40,41^ Indeed, when the tetraol (2*S*,3*R*)-**29** was reacted for a longer time (1 h) in otherwise identical conditions, an inseparable mixture of (2*S*,3*R*)-**16** and of the internal 2,3-acetonide (2*S*,3*R*)-**30** was obtained in a ∼1:1 ratio (as determined by NMR analysis, Figures S6–S9). The reduced nucleophilicity of the difluorocarbinol group at position 3 (due to the electron-depleting effect of fluorine atoms) may contribute to the excellent regioselectivity of the kinetic conditions.

**Scheme 7:**
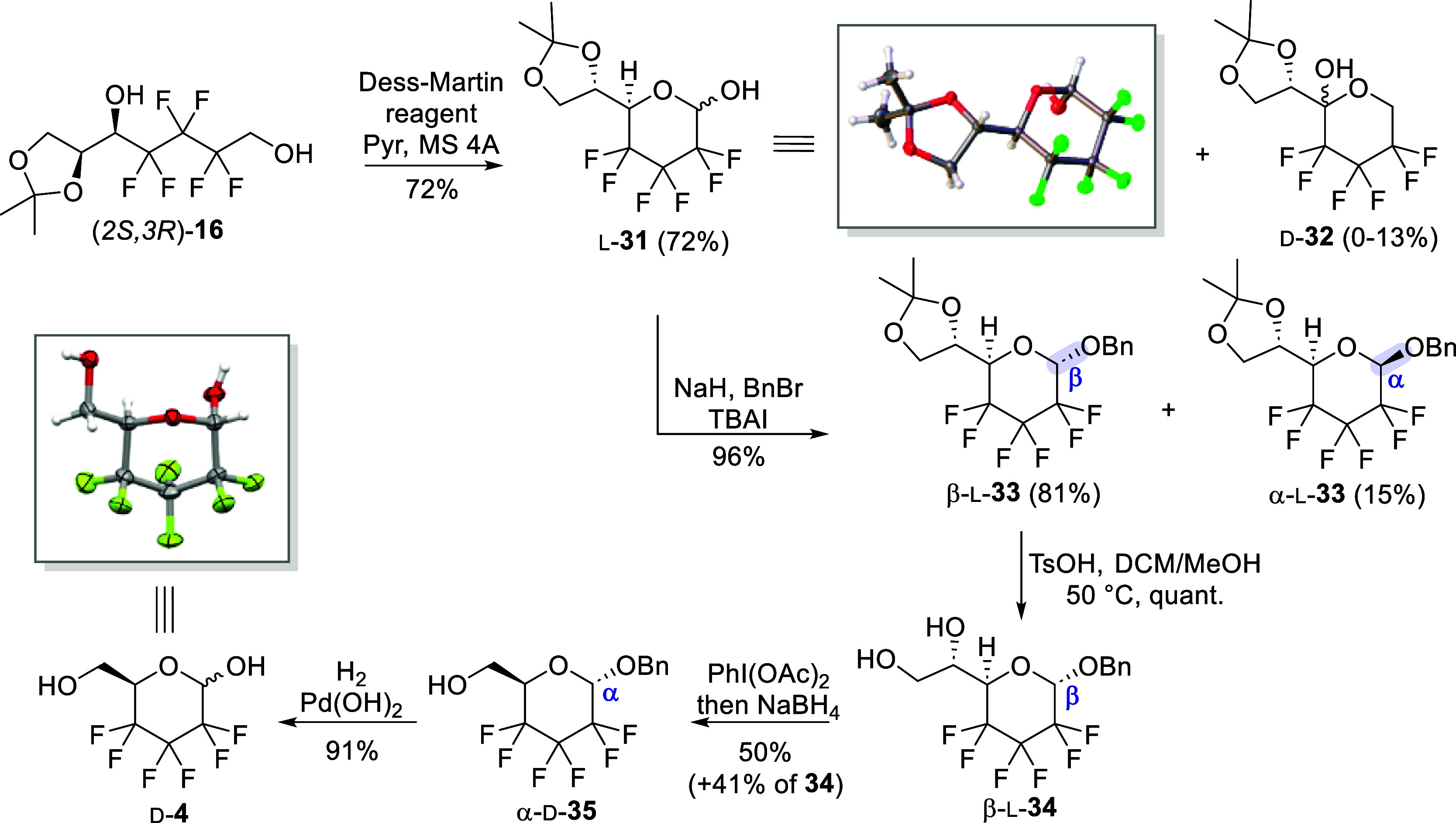
Access to the Pyranose Scaffold and Final Steps toward Synthesis of d-**4** See Figure S1 for anomeric assignments.

**Figure 1:**
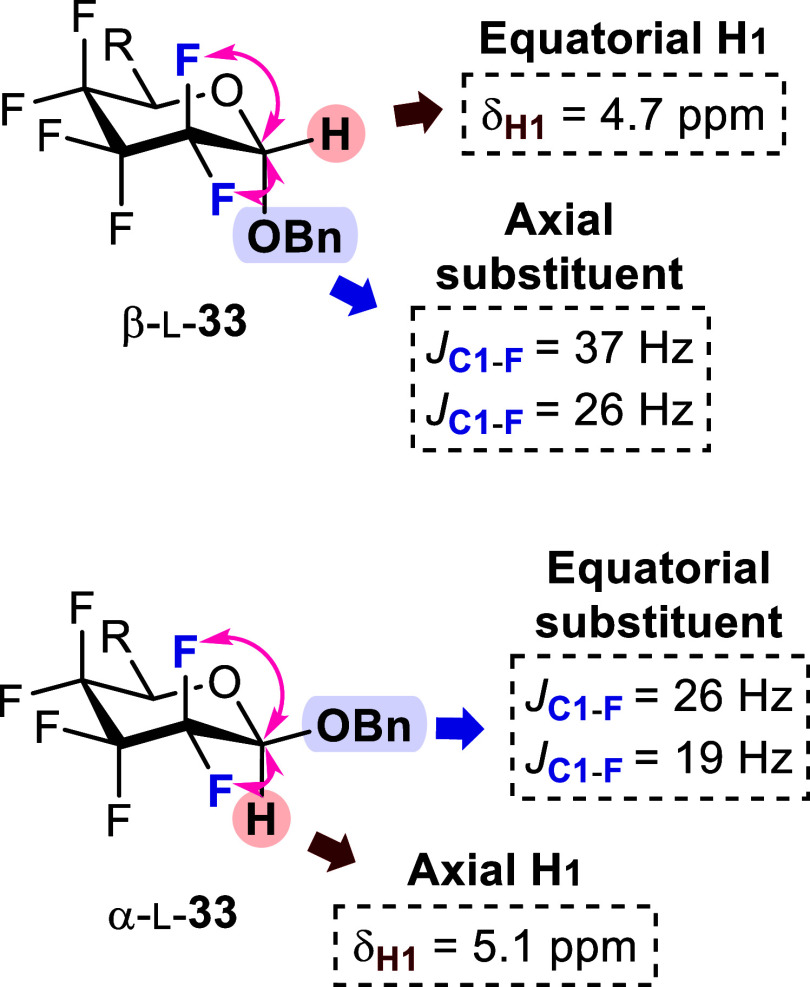
Key NMR observations to determine the anomeric configuration of β-l-**33** and α-l-**33** (for clarity, the C5 substituent was abbreviated as R).

The final steps to access d-**4** are shown in Scheme 7. The oxidative cyclization of (2*S*,3*R*)-**16** was first performed, and Dess-Martin reagent was yet again employed. The latter was added in 4 portions onto (2*S*,3*R*)-**16** to favor progressive primary alcohol oxidation and concomitant cyclization. This gave aldoheptose l-**31** in 72% yield, as a mixture of both α and β anomers. Addition of molecular sieves was found to be crucial, presumably to prevent partial hydration of the intermediate aldehyde and thus promote the desired ring-closure reaction. Interestingly, formation of ketoheptose d-**32**, resulting from oxidation of secondary alcohol of (2*S*,3*R*)-**16** followed by cyclization with the primary alcohol, was sometimes observed (0–13%, depending on batch), but it could be easily removed by chromatography. This compound was isolated as a single enantiomer, but the anomeric configuration could not be determined. To undertake the last steps of the synthesis toward cleavage of the additional carbon introduced during olefination, the anomeric alcohol of l-**31** was protected as a benzyl ether in basic anomeric O-alkylation conditions. Both anomers, β-l-**33** (axial substituent) and α-l-**33** (equatorial substituent), were formed and could be readily separated by chromatography, albeit the formation of the β-benzylated pyranose was markedly preferred (β/α 5:1) (see Figure S1 for a detailed explanation regarding nomenclature and anomeric assignments of these atypical carbohydrate derivatives). The anomeric configuration of β-l-**33** and α-l-**33** was ascertained based on several key NMR observations such as the typical coupling constants of the anomeric carbon with the adjacent fluorine atoms depending on the orientation of the anomeric substituent. The observed values for the major (^2^*J*_C1–F2_ = 37.4 and 25.7 Hz) and minor (^2^*J*_F2–C1_ = 27.1 and 18.3 Hz) glycosides l-**33** were consistent with the respective β (axial substituent) and α (equatorial substituent) configurations (Figure 1). These conclusions were also supported by the equatorial H1 proton of the β anomer being more deshielded (δ_H1β_ = 5.1 ppm, in acetone-*d*_6_) than the axial α anomer proton (δ_H1α_ = 4.7 ppm, in acetone-*d*_6_). Moving forward, it was deemed useful to work with a pure anomer as a safeguard, as it would allow for facile detection of potential C-5 epimerization at the intermediate aldehyde stage during diol cleavage. The synthesis was then continued with the major β-anomer of l-**33** by hydrolysis of the acetonide in acidic conditions which reliably provided β-l-**34** and set the stage for diol cleavage. The latter reaction was conducted with (diacetoxyiodo)benzene, followed by rapid aldehyde reduction in a one-pot procedure. A reasonable 50% yield of the hydroxymethyl derivative α-d-**35** was obtained and up to 40% of the starting sugar β-l-**34** could be easily recovered. The pure diastereoisomer α-d-**35** was detected by ^1^H NMR analysis, which confirmed that epimerization of C-5 did not occur during the oxidative diol cleavage step. Finally, a hydrogenolysis was performed to deprotect the anomeric position, which afforded the desired 2,3,4-trideoxy-2,2,3,3,4,4-hexafluoro-d-*glycero*-hexopyranose d-**4** in an excellent 91% isolated yield (7:3 α/β in acetone-*d*_6_). Crystallization of d-**4** was further achieved by slow evaporation of a 2:1 hexane/Et_2_O solution, and X-ray crystallographic analysis confirmed the expected C-5 configuration.

**Scheme 8:**
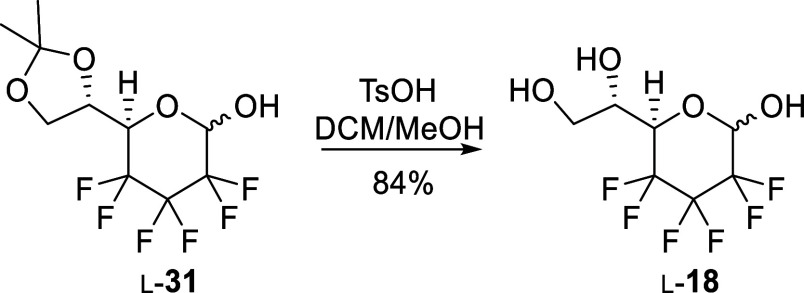
Access to the Hexafluorinated Heptose l-**18**

As discussed during the retrosynthetic analysis, the novel hexafluorinated heptose is also accessible from pyranose intermediate l-**31**, by removing the acetonide protecting group. Thereby, the acetonide of l-**31** was cleaved in similar conditions as for β-l-**33**, efficiently affording 2,3,4-trideoxy-2,2,3,3,4,4-hexafluoro-l-*threo*-heptopyranose (l-**18**) (Scheme 8).

**Figure 2:**
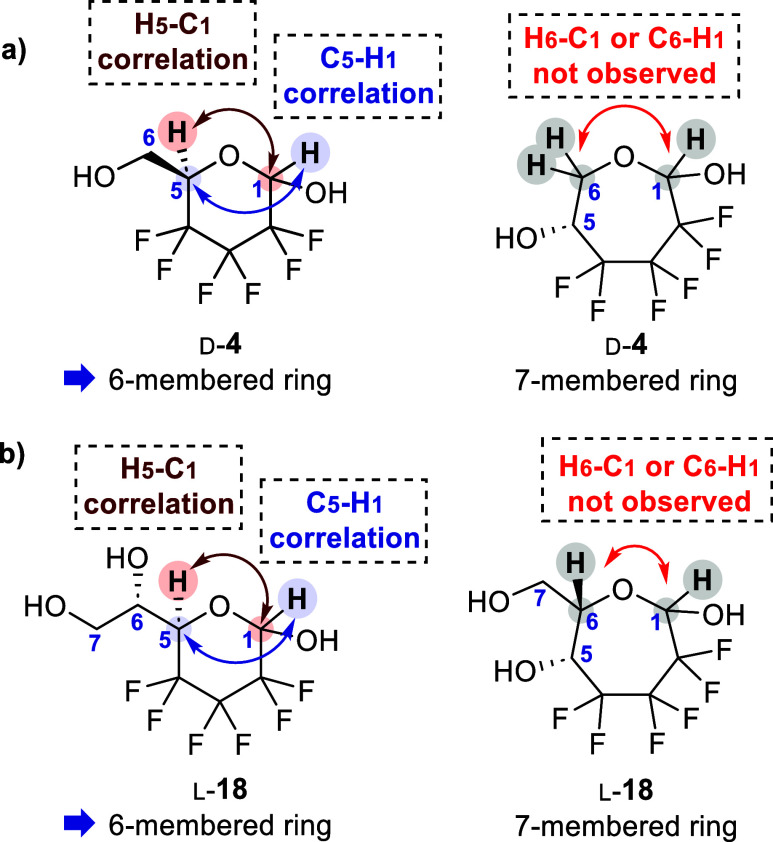
Key NMR correlations observed in HMBC analysis to determine the size of the heterocyclic ring in solution (acetone-*d*_6_) of **a**) the hexose d-**4** and **b**) the heptose l-**18**, after ten h of equilibration.

Vicinal fluorination renders alcohol groups less nucleophilic, which may affect the ring-tautomer composition. Nevertheless, both **d**-4 and l-**18** were unambiguously shown to possess the pyranose ring structure in solution, as proven by the HMBC correlation (acetone solvent, Figures 2, S11 and S12).

## Conclusions

A *de novo* 12-step synthesis of d-**4** was successfully achieved. The proposed approach features steps that are experimentally convenient and reproducible. Our strategy is based on a SAD of a hexafluorinated *trans*-disubstituted alkene, allowing to reliably install chirality with high enantioselectivity (95:5 *er*) without the need for extra derivatization or resolution steps. The expected major enantiomer could readily be crystallized to further reach enantiopurity and ultimately afford the pure 2,3,4-trideoxy-2,2,3,3,4,4-hexafluoro-d-*glycero*-hexopyranose (d-**4**), after an oxidative ring-closing and diol cleavage sequence to remove the extra carbon atom. In addition, the envisioned synthesis allowed access to the novel 2,3,4-trideoxy-2,2,3,3,4,4-hexafluoro-l-*threo*-heptopyranose (l-**18**). We hope that this contribution facilitates access to this polar hydrophobic sugar, which will stimulate their use in molecular recognition studies in glycobiology as well as in the design of bioactive compounds.

## Experimental Section

### General Methods

All chemical reagents were obtained from commercial sources and used without further purification. Anhydrous solvents were purchased from commercial sources. When appropriate, glassware was flame-dried under a vacuum and cooled under Ar prior to use. Water or air sensitive reactions were performed under an inert atmosphere, using dry solvents. Reactions were monitored by TLC (Merck Kieselgel 60 F_254_, aluminum sheet). TLC plates were visualized under UV light (254 nm) or by staining with cerium molybdate (for intermediates with benzyl substituent) or KMnO_4_ (for intermediates without benzyl substituent), followed by brief heating. Column chromatography were performed on silica gel (Merck silica gel 60, particle size 40–63 μm). Nuclear magnetic resonance spectra were recorded using either a Bruker Ultrashield 400 or 500 MHz spectrometer. The chemical shift (δ) is given in ppm using the residual solvent peak as an internal standard. The term “dm” refers to a doublet of multiplet. Atom numbering used for NMR attribution is different from the numbers used in nomenclature of compounds. Structural assignments were made with additional information from DEPT, correlation spectroscopy, HSQC, and HMBC experiments. The signals corresponding to the CF_2_ atoms are poorly visible in the ^13^C NMR spectrum, and a range of chemical shifts is described for each compound. This range was determined using HMBC analysis where the nearby hydrogens correlate to the CF_2_ atoms (see Supporting Information Section S4 and Figure S10). High-resolution mass spectrometry (HRMS) profiles were measured on a Bruker Daltonics MaXis time-of-flight (TOF) mass spectrometer. A tolerance of 5 ppm was applied between the calculated and experimental values. Melting points of ±1 °C were measured on a Kofler heating bar apparatus (Heizbank, Reichert) calibrated with acetanilide (mp 114.5 °C). Optical rotations were measured at 589 nm on a PerkinElmer Polarimeter model 241, and values reported are the average of 5 measurements.

#### 3,3,4,4,5,5-Hexafluorooxane-2-ol (**20**)

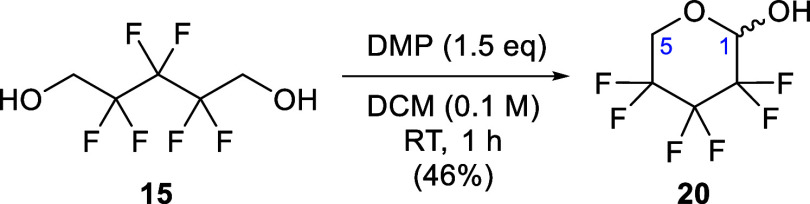



To a stirred solution of commercially available 2,2,3,3,4,4-hexafluoropentane-1,5-diol **15** (500 mg, 2.35 mmol, 1 equiv) in DCM (25 mL) were added 4A molecular sieves (1 spatula) and DMP (1.5 g, 3.54 mmol, 1.5 equiv). After 1 h, completion was confirmed by TLC (6:4 Hex/Et_2_O, *R*_*f*_ (**15**) = 0.25, *R*_*f*_ (**20**) = 0.40), and the mixture was concentrated under reduced pressure. Then, 10 mL of a 6:4 Hex/Et_2_O solution were added in the flask and the thick white foam was directly loaded and purified by silica gel chromatography (60:40 Hex/Et_2_O). Solvents were carefully removed at 500 mbar/35 °C and the desired compound was not concentrated until complete dryness because of its volatility. Pure **20** was obtained (230 mg, 46%, corrected for remaining solvents as quantified by ^1^H NMR analysis) as a colorless oil. ^**1**^**H NMR** (400 MHz, DMSO-*d*_6_): δ 8.47 (d, *J* = 6.0 Hz, 1H, OH), 5.40 (m, 1H, H-1), 4.45–4.15 (m, 2H, H-5a + H-5b) ppm; ^**1**^**H{**^**19**^**F} NMR** (500 MHz, DMSO-*d*_6_): δ 8.47 (d, *J* = 6.0 Hz, 1H, OH), 5.40 (d, *J* = 6.0 Hz, 1H, H-1), 4.35 (d, *J* = 13.6 Hz, 1H, H-5a), 4.24 (d, *J* = 13.6 Hz, 1H, H-5b) ppm; ^**19**^**F NMR** (376 MHz, DMSO-*d*_6_): δ −121.2 to −128.2 (m, 4F), −134.2 (m, 2F) ppm; ^**19**^**F{**^**1**^**H} NMR** (471 MHz, DMSO-*d*_6_): δ −121.2 to −128.2 (m, 4F), −134.2 (m, 2F) ppm; ^**13**^**C{**^**1**^**H} NMR** (101 MHz, DMSO-*d*_6_): δ 120.5–110.5 (3× CF_2_), 90.8 (dd, *J* = 32.5, 23.1 Hz, C-1), 59.1 (t, *J* = 27.6 Hz, C-5) ppm; **HRMS** (ESI^–^) *m*/*z*: [M – H]^−^ calcd for C_5_H_3_F_6_O_2_, 209.0043; found, 209.0043.

#### (rac)-Methyl 2-(3,3,4,4,5,5-hexafluorooxan-2-yl)acetate (**22**)

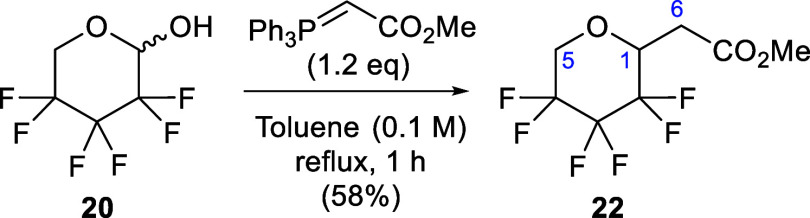



To a stirred solution of **20** (350 mg, 1.66 mmol, 1 equiv) in toluene (15 mL) was added MTPPA (670 mg, 2.00 mmol, 1.2 equiv), and the mixture was refluxed using a heating mantle. After 1 h, near completion was confirmed by TLC (8:2 Hex/Et_2_O, *R*_*f*_ (**20**) = 0.20 *R*_*f*_ (**22**) = 0.5), and the solution was cooled to room temperature. Solids were filtered off, and the mixture was concentrated under reduced pressure. The obtained crude product was purified by silica gel chromatography (80:20 Hex/Et_2_O) to afford **22** (255 mg, 58%) as a white solid. **mp** 57 °C (obtained after solvent evaporation); ^**1**^**H NMR** (400 MHz, DMSO-*d*_6_): δ 4.57–4.44 (m, 2H, H-5a, H-1), 4.25 (ddd, *J* = 32.3, 13.5, 3.7 Hz, 1H, H-5b), 3.67 (s, 3H, −CO_2_CH_3_), 2.97 (dd, *J* = 16.4, 3.4 Hz, 1H, H-6a), 2.67 (dd, *J* = 16.4, 9.2 Hz, 1H, H-6b) ppm; ^**1**^**H{**^**19**^**F} NMR** (500 MHz, DMSO-*d*_6_): δ 4.51 (d, *J* = 13.6 Hz, 1H, H-5a), 4.49 (dd, *J* = 9.1, 3.3 Hz, H-1), 4.25 (d, *J* = 13.6 Hz, 1H, H-5b), 3.67 (s, 3H, −CO_2_CH_3_), 2.97 (dd, *J* = 16.4, 3.3 Hz, 1H, H-6a), 2.67 (dd, *J* = 16.4, 9.1 Hz, 1H, H-6b) ppm; ^**19**^**F NMR** (376 MHz, DMSO-*d*_6_): δ −121.3 (dm, *J* = 263.3 Hz, 1F), −126.5 (br d, *J* = 263.1 Hz, 1F), −129.9 (br d, *J* = 261.9 Hz, 1F), −130.2 (dm, *J* = 265.3 Hz, 1F), −131.8 (dm, *J* = 261.9 Hz, 1F), −148.3 (br d, *J* = 263.0 Hz, 1F) ppm; ^**19**^**F{**^**1**^**H} NMR** (471 MHz, DMSO-*d*_6_): δ −121.3 (dm, *J* = 263.3 Hz, 1F), −126.5 (br d, *J* = 263.1 Hz, 1F), −129.9 (br d, *J* = 261.9 Hz, 1F), −130.2 (dm, *J* = 265.3 Hz, 1F), −131.8 (dm, *J* = 261.9 Hz, 1F), −148.3 (br d, *J* = 263.0 Hz, 1F) ppm; ^**13**^**C{**^**1**^**H} NMR** (101 MHz, DMSO-*d*_6_): δ 168.9 (s, C=O), 120.5–110.5 (3× CF_2_), 72.7 (dd, *J* = 26.3, 21.2 Hz, C-1), 65.9 (dd, *J* = 31.1, 24.9 Hz, C-5), 52.2 (s, −CO_2_CH_3_), 31.5 (s, C-6) ppm; **HRMS** (ESI^+^) *m*/*z*: [M + H]^+^ calcd for C_8_H_8_F_6_O_3_, 267.0450; found, 267.0449.

#### 5-(Benzyloxy)-2,2,3,3,4,4-hexafluoropentan-1-ol (**23**)

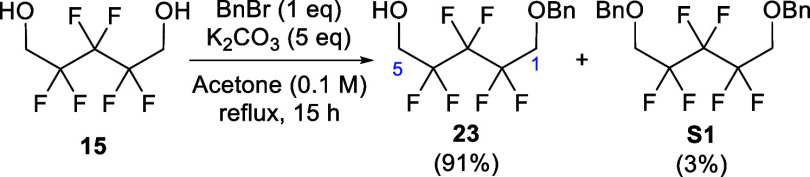



To a stirred solution of commercially available 2,2,3,3,4,4-hexafluoropentane-1,5-diol **15** (5 g, 23.57 mmol, 1 equiv) in acetone (200 mL) were added potassium carbonate (16.3 g, 117.85 mmol, 5 equiv) and benzyl bromide (2.82 mL, 23.57 mmol, 1 equiv), and the mixture was refluxed using a heating mantle. After 15 h, near completion was confirmed by TLC (7:3 Hex/AcOEt, *R*_*f*_ (**15**) = 0.1, *R*_*f*_ (**23**) = 0.3, *R*_*f*_ (**S1**) = 0.6), and the solution was cooled to room temparature. Solids were filtered off, and the mixture was concentrated under reduced pressure. The obtained crude was purified by silica gel chromatography (75:25 Hex/AcOEt) to afford **23** (6.5 g, 91%) and **S1** (250 mg, 3%), both as colorless oils.

##### Characterization of **23**

^**1**^**H NMR** (400 MHz, DMSO-*d*_6_): δ 7.41–7.27 (m, 5H, H–Ar), 5.93 (t, *J* = 6.6 Hz, 1H, OH), 4.65 (s, 2H, –CH_2_Ph), 4.07 (t, *J* = 15.2 Hz, 2H, H-1), 3.91 (dt, *J* = 15.5 Hz, *J* = 6.6 Hz, 2H, H-5) ppm; ^**1**^**H{**^**19**^**F} NMR** (500 MHz, DMSO-*d*_6_): 7.41–7.27 (m, 5H, H–Ar), 5.93 (t, *J* = 6.6 Hz, 1H, OH), 4.65 (s, 2H, –CH_2_Ph), 4.07 (s, 2H, H-1), 3.91 (d, *J* = 6.6 Hz, 2H, H-5) ppm; ^**19**^**F NMR** (376 MHz, DMSO-*d*_6_): δ −119.2 (m, 2F), −121.2 (tt, *J* = 16.4, 8.6, 2F), −125.4 (m, 2F) ppm; ^**19**^**F{**^**1**^**H} NMR** (471 MHz, DMSO-*d*_6_): δ −119.2 (br t, *J* = 9.3 Hz, 2F), −121.2 (br t, *J* = 9.3 Hz, 2F), −125.4 (br s, 2F) ppm; ^**13**^**C{**^**1**^**H} NMR** (101 MHz, DMSO-*d*_6_): δ 137.1 (s, C–Ar), 128.4 (s, C–Ar), 127.9 (s, C–Ar), 127.7 (s, C–Ar), 120.5–110.5 (3× CF_2_), 73.3 (s, –CH_2_Ph), 66.3 (t, *J* = 24.2 Hz, C-1), 58.8 (t, *J* = 24.5 Hz, C-5) ppm; **HRMS** (ESI^+^) *m*/*z*: [M + Na]^+^ calcd for C_12_H_12_F_6_O_2_Na, 325.0634; found, 325.0629.

##### Characterization of **S1**

^**1**^**H NMR** (400 MHz, DMSO-*d*_6_): δ 7.41–7.29 (m, 10H, H–Ar), 4.64 (s, 4H, –CH_2_Ph), 4.09 (t, *J* = 15.0 Hz, 4H, –CH_2_CF_2_) ppm; ^**1**^**H{**^**19**^**F} NMR** (500 MHz, DMSO-*d*_6_): 7.41–7.29 (m, 10H, H–Ar), 4.64 (s, 4H, –CH_2_Ph), 4.09 (s, 4H, –CH_2_CF_2_) ppm; ^**19**^**F NMR** (376 MHz, DMSO-*d*_6_): δ −119.2 (m, 4F), −125.4 (s, 2F) ppm; ^**19**^**F{**^**1**^**H} NMR** (471 MHz, DMSO-*d*_6_): δ −119.2 (s, 4F), −125.3 (s, 2F) ppm; ^**13**^**C{**^**1**^**H} NMR** (101 MHz, DMSO-*d*_6_): δ 137.0 (s, C–Ar), 128.4 (s, C–Ar), 127.9 (s, C–Ar), 127.7 (s, C–Ar), 120.5–110.5 (3× CF_2_), 73.3 (s, –CH_2_Ph), 66.3 (t, *J* = 25.4 Hz, –CH_2_CF_2_) ppm; **HRMS** (ESI^+^) *m*/*z*: [M + NH_4_]^+^ calcd for C_19_H_22_F_6_NO_2_, 410.1555; found, 410.1540.

#### 5-(Benzyloxy)-2,2,3,3,4,4-hexafluoropentane-1,1-diol (**25**)

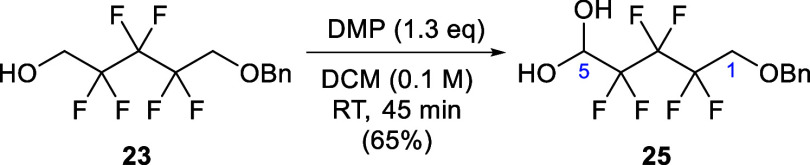



To a stirred solution of **23** (6 g, 19.85 mmol, 1 equiv) in DCM (150 mL) was added DMP (10.95 g, 25.81 mmol, 1.3 equiv). After 45 min at room temperature, completion was confirmed by TLC (60:40 Hex/AcOEt, R_f_ (**23**) = 0.45, R_f_ (**25**) = 0.30), and the solution was concentrated under reduced pressure. The residue was rediluted in AcOEt (100 mL) and a 1:1 NaHCO_3_/Na_2_S_2_O_3_ saturated solution (80 mL) was added. Mixture was stirred for 30 min, and the aqueous layer was extracted with AcOEt (100 mL). The combined organic layer was washed with 1:1 NaHCO_3_/Na_2_S_2_O_3_ saturated solution (2 × 80 mL), dried over MgSO_4_, filtered, and concentrated under reduced pressure. The obtained crude product was purified by silica gel chromatography (75:25 Hex/AcOEt) to afford **25** (3.9 g, 65%) as a colorless oil that becomes a white solid. *Note 1*: switching from DCM to AcOEt for liquid–liquid extraction avoids excessive foam formation. *Note* 2: the NMR sample after purification needs to equilibrate overnight in deuterated dimethyl sulfoxide (contains traces of water) to obtain a pure NMR profile of the hydrate **25**. **mp** 69 °C (obtained after solvent evaporation); ^**1**^**H NMR** (400 MHz, DMSO-*d*_6_): δ 7.41–7.28 (m, 5H, H–Ar), 7.13 (d, *J* = 6.8 Hz, 2H, OH), 5.12 (m, 1H, H-5), 4.64 (s, 2H, –CH_2_Ph), 4.05 (t, *J* = 15.1 Hz, 2H, H-1) ppm; ^**1**^**H{**^**19**^**F} NMR** (500 MHz, DMSO-*d*_6_): 7.41–7.28 (m, 5H, H–Ar), 7.13 (d, *J* = 6.8 Hz, 2H, OH), 5.12 (t, *J* = 6.8 Hz, 1H, H-5), 4.64 (s, 2H, –CH_2_Ph), 4.05 (s, 2H, H-1) ppm; ^**19**^**F NMR** (376 MHz, DMSO-*d*_6_): δ −119.2 (m, 2F), −124.3 (br s, 2F), −127.2 (q, *J* = 9.5 Hz, 2F) ppm; ^**19**^**F{**^**1**^**H} NMR** (471 MHz, DMSO-*d*_6_): δ −119.2 (br t, *J* = 9.6 Hz, 2F), −124.3 (s, 2F), −127.2 (br t, *J* = 9.6 Hz, 2F) ppm; ^**13**^**C{**^**1**^**H} NMR** (101 MHz, DMSO-*d*_6_): δ 137.2 (s, C–Ar), 128.4 (s, C–Ar), 127.9 (s, C–Ar), 127.7 (s, C–Ar), 120.5–110.5 (3× CF_2_), 85.9 (t, *J* = 25.5 Hz, C-5), 73.3 (s, –CH_2_Ph), 66.4 (t, *J* = 23.8 Hz, C-1) ppm; **HRMS** (ESI^+^) *m*/*z*: [M + NH_4_]^+^ calcd for C_12_H_16_NF_6_O_3_, 336.1028; found, 336.1025.

#### Methyl (E)-7-(Benzyloxy)-4,4,5,5,6,6-hexafluorohept-2-enoate (**26**)

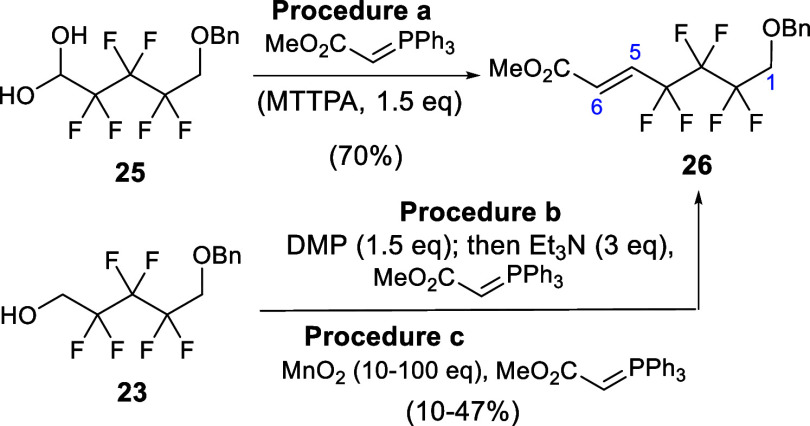



##### Procedure a

To a stirred solution of **25** (6.3 g, 20.98 mmol, 1 equiv) in toluene (200 mL) were added MS 4A (2 spatula) and MTTPA (10.5 g, 31.5 mmol, 1.5 equiv), and the mixture was refluxed using a heating mantle. After 2h, completion was confirmed by TLC (80:20 Hex/AcOEt, R_f_ (**25**) = 0.15, Rf (**26**) = 0.5). Mixture was cooled to room temperature, solids were filtered off and rinsed with DCM, and the filtrate was concentrated under reduced pressure. The obtained crude was purified by silica gel chromatography (95:5 to 90:10 Hex/AcOEt) to afford **26** (5.1 g, 70%) as a colorless oil.

##### Procedure b

To a stirred solution of **23** (0.5 g, 1.65 mmol, 1 equiv) in toluene (15 mL) were added MS 4A (1 spatula) and Dess-Martin reagent (1.1 g, 2.48 mmol, 1.5 equiv). After 45 min at room temperature, completion of oxidation was confirmed by TLC (60:40 Hex/AcOEt, *R*_*f*_ (**23**) = 0.45, *R*_*f*_ (**25**) = 0.30). Et_3_N (0.7 mL, 4.95 mmol, 3 equiv) and MTPPA (830 mg, 2.48 mmol, 1.5 equiv) were then added into the reaction, and the mixture was refluxed using a heating mantle. After 1 h, completion of olefination was confirmed by TLC (80:20 Hex/AcOEt, *R*_*f*_ (**25**) = 0.15, *R*_*f*_ (**26**) = 0.5). Mixture was cooled down to room temperature, solids were filtered off and rinsed with DCM, and the filtrate was concentrated under reduced pressure. The obtained crude was purified by silica gel chromatography (95:5 Hex/AcOEt) to afford **26** (395 mg, 65%) as a colorless oil. Note: a 12 g scale experiment resulted in a 40% yield. Synthesis of **25** followed by Procedure A is a more convenient choice on a large scale.

##### Procedure C

To a stirred solution of **23** (100 mg, 0.33 mmol, 1 equiv) in toluene (4 mL) were added MS 4A (1 spatula), MTPPA (165 mg, 0.50 mmol, 1.5 equiv), and MnO_2_ (2.8 g, 33.33 mmol, 100 equiv), and the mixture was refluxed using a heating mantle. After 30 h, conversion was incomplete, and the mixture was cooled down to room temperature and filtered through a Celite pad; the filtrate was concentrated under reduced pressure. The obtained crude was purified by silica gel chromatography (9:1 Hex/AcOEt) to afford **26** (55 mg, 47%) as a colorless oil.

##### Characterization of **26**

^**1**^**H NMR** (400 MHz, DMSO-*d*_6_): δ 7.42–7.28 (m, 5H, H–Ar), 6.95 (dt, *J* = 15.5, 12.6 Hz, 1H, H-5), 6.70 (dt, *J* = 15.8, 2.1 Hz, 1H, H-6), 4.65 (s, 2H, –CH_2_Ph), 4.12 (t, *J* = 14.9 Hz, 2H, H-1), 3.76 (s, 3H, −CO_2_CH_3_) ppm; ^**1**^**H{**^**19**^**F} NMR** (500 MHz, DMSO-*d*_6_): δ 7.40–7.28 (m, 5H, H–Ar), 6.94 (d, *J* = 15.8 Hz, 1H, H-5), 6.68 (d, *J* = 15.8 Hz, 1H, H-6), 4.65 (s, 2H, –CH_2_Ph), 4.12 (s, 2H, H-1), 3.76 (s, 3H, −CO_2_CH_3_) ppm; ^**19**^**F NMR** (376 MHz, DMSO-*d*_6_): δ −112.6 (m, 2F), −118.2 (m, 2F), −125.2 (br s, 2F) ppm; ^**19**^**F{**^**1**^**H} NMR** (471 MHz, DMSO-*d*_6_): δ −112.6 (m, 2F), −118.2 (br t, *J* = 8.9 Hz, 2F), −125.2 (br s, 2F) ppm; ^**13**^**C{**^**1**^**H} NMR** (101 MHz, DMSO-*d*_6_): δ 163.9 (s, C=O), 137.0 (s, C–Ar), 130.8 (t, *J* = 23.4 Hz, C-5), 130.2 (t, *J* = 8.7 Hz, C-6), 128.4 (s, C–Ar), 127.9 (s, C–Ar), 127.7 (s, C–Ar), 120.5–110.5 (3× CF_2_), 73.3 (s, –CH_2_Ph), 66.2 (t, *J* = 24.7 Hz, C-1), 52.5 (s, −CO_2_CH_3_) ppm; **HRMS** (ESI^+^) *m*/*z*: [M + NH_4_]^+^ calcd for C_15_H_18_NF_6_O_3_, 374.1185; found, 374.1185.

#### (*E*)-7-(Benzyloxy)-4,4,5,5,6,6-hexafluorohept-2-en-1-ol (**27**)

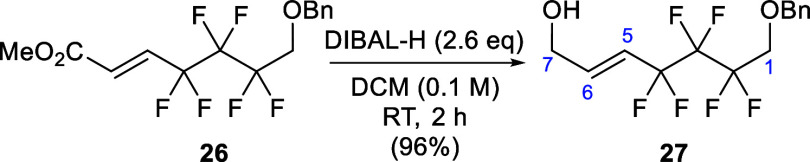



Under inert conditions, a stirred solution of **26** (4.5 g, 12.63 mmol, 1 equiv) in anhydrous DCM (100 mL) was cooled down to −60 °C. Then, a 1 M solution of DIBAL-H in hexane (33 mL, 33 mmol, 2.6 equiv) was dropwise added over 5 min. Mixture was stirred during 1 h and the cooling bath was then removed. After 2 h, completion was confirmed by TLC (8:2 Hex/AcOEt, *R*_*f*_ (**26**) = 0.4, *R*_*f*_ (**27**) = 0.15), and the solution was cooled to 0 °C. Reaction was carefully quenched by dropwise addition of MeOH (30 mL) and stirring during 10 min. Mixture was washed with 1 M HCl (100 mL) and the aqueous layer was extracted with DCM (2 × 75 mL). The combined organic layer was dried over MgSO_4_ and filtered, and the filtrate was concentrated under reduced pressure. The obtained crude was purified by silica gel chromatography (75:25 Hex/AcOEt) to afford **27** (4.0 g, 96%) as a colorless oil. ^**1**^**H NMR** (400 MHz, DMSO-*d*_6_): δ 7.44–7.25 (m, 5H, H–Ar), 6.55 (m, 1H, H-6), 5.94 (m, 1H, H-5), 5.21 (t, *J* = 5.3 Hz, 1H, OH), 4.66 (s, 2H, –CH_2_Ph), 4.13 (m, 2H, H-7), 4.07 (t, *J* = 15.2 Hz, 2H, H-1) ppm; ^**1**^**H{**^**19**^**F} NMR** (500 MHz, DMSO-*d*_6_): 7.44–7.25 (m, 5H, H–Ar), 6.55 (dt, *J* = 15.7, 3.5 Hz, 1H, H-6), 5.92 (dt, *J* = 15.7, 2.3 Hz, 1H, H-5), 5.20 (t, *J* = 5.3 Hz, 1H, OH), 4.66 (s, 2H, –CH_2_Ph), 4.12 (ddd, *J* = 5.3, 3.2, 2.3 Hz, 2H, H-7), 4.07 (s, 2H, H-1) ppm; ^**19**^**F NMR** (376 MHz, DMSO-*d*_6_): δ −109.9 (br s, 2F), −118.4 (m, 2F), −125.3 (br s, 2F) ppm; ^**19**^**F{**^**1**^**H} NMR** (471 MHz, DMSO-*d*_6_): δ −109.9 (br t, *J* = 9.3 Hz, 2F), −118.4 (t, *J* = 9.3 Hz, 2F), −125.3 (br s, 2F) ppm; ^**13**^**C{**^**1**^**H} NMR** (101 MHz, DMSO-*d*_6_): δ 143.3 (t, *J* = 8.3 Hz, C-6), 137.1 (s, C–Ar), 137.1 (s, C–Ar), 128.4 (s, C–Ar), 127.9 (s, C–Ar), 127.7 (s, C–Ar), 120.5–110.5 (3× CF_2_), 114.6 (t, *J* = 22.7 Hz, C-5), 73.3 (s, –CH_2_Ph), 66.4 (t, *J* = 24.2 Hz, C-1), 59.7 (s, C-7) ppm; **HRMS** (ESI^+^) *m*/*z*: [M + Na]^+^ calcd for C_14_H_14_F_6_O_2_Na, 351.0790; found, 351.0786.

#### (*E*)-1,7-bis(benzyloxy)-4,4,5,5,6,6-hexafluorohept-2-ene (**14**)

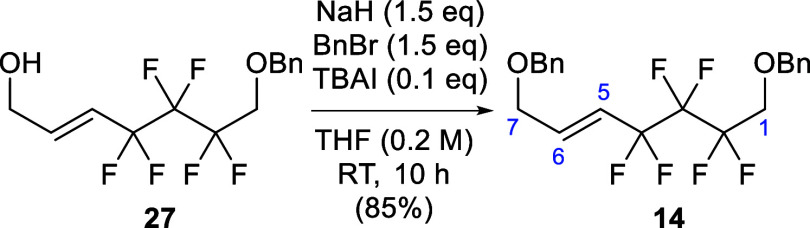



Under inert conditions, a stirred solution of **27** (4.0 g, 12.15 mmol, 1 equiv) in anhydrous tetrahydrofuran (THF) (60 mL) was cooled down to 0 °C. Then, sodium hydride (730 mg of 60% mineral oil, 18.25 mmol, 1.5 equiv) was added, and gas formation was observed during 15 min. Ice bath was removed, followed by addition of TBAI (450 mg, 1.22 mmol, 0.1 equiv) and benzyl bromide (2.2 mL, 18.28 mmol, 1.5 equiv) and reaction was allowed to stir at room temparature. After 10 h, completion was confirmed by TLC (8:2 Hex/AcOEt, *R*_*f*_ (**27**) = 0.15, *R*_*f*_ (**14**) = 0.55) and the solution was diluted with Et_2_O (50 mL) and cooled down to 0 °C. Reaction was carefully quenched by dropwise addition of water (30 mL) and stirring 10 min. Organic layer was washed with water (1 × 50 mL) and brine (1 × 50 mL), dried over MgSO_4_, and filtered, and the filtrate was concentrated under reduced pressure. The obtained crude was purified by silica gel chromatography (95:5 to 85:15 Hex/AcOEt) to afford **14** (4.3 g, 85%) as a colorless oil. ^**1**^**H NMR** (400 MHz, DMSO-*d*_6_): δ 7.40–7.25 (m, 10H, H–Ar), 6.54 (m, 1H, H-6), 6.02 (m, 1H, H-5), 4.65 (s, 2H, –CH_2_Ph), 4.53 (s, 2H, –CH_2_Ph), 4.14 (m, 2H, H-7), 4.07 (t, *J* = 15.2 Hz, 2H, H-1) ppm; ^**1**^**H{**^**19**^**F} NMR** (500 MHz, DMSO-*d*_6_): δ 7.40–7.25 (m, 10H, H–Ar), 6.54 (dd, *J* = 15.8, 4.0 Hz, 1H, H-6), 6.02 (dd, *J* = 15.8, 2.1 Hz, 1H, H-5), 4.65 (s, 2H, –CH_2_Ph), 4.53 (s, 2H, –CH_2_Ph), 4.14 (dd, *J* = 4.0, 2.1 Hz, 2H, H-7), 4.07 (s, 2H, H-1) ppm; ^**19**^**F NMR** (376 MHz, DMSO-*d*_6_): δ −110.5 (br s, 2F), −118.4 (m, 2F), −125.3 (br s, 2F) ppm; ^**19**^**F{**^**1**^**H} NMR** (471 MHz, DMSO-*d*_6_): δ −110.5 (br t, *J* = 8.9 Hz, 2F), −118.4 (t, *J* = 8.9 Hz, 2F), −125.3 (br s, 2F) ppm; ^**13**^**C{**^**1**^**H} NMR** (101 MHz, DMSO-*d*_6_): δ 139.3 (t, *J* = 8.8 Hz, C-6), 137.9 (s, C–Ar), 137.1 (s, C–Ar), 128.4 (s, C–Ar), 128.3 (s, C–Ar), 127.9 (s, C–Ar), 127.7 (s, C–Ar), 127.6 (s, C–Ar), 127.5 (s, C–Ar), 120.5–110.5 (3× CF_2_), 16.5 (t, *J* = 22.7 Hz, C-5), 73.3 (s, –CH_2_Ph), 71.9 (s, –CH_2_Ph), 67.7 (s, C-7), 66.4 (t, *J* = 24.2 Hz, C-1), ppm; **HRMS** (ESI^+^) *m*/*z*: [M + Na]^+^ calcd for C_21_H_20_F_6_O_2_Na, 441.1260; found, 441.1266.

#### (2S,3R)-1,7-Bis(benzyloxy)-4,4,5,5,6,6-hexafluoroheptane-2,3-diol (**28**)

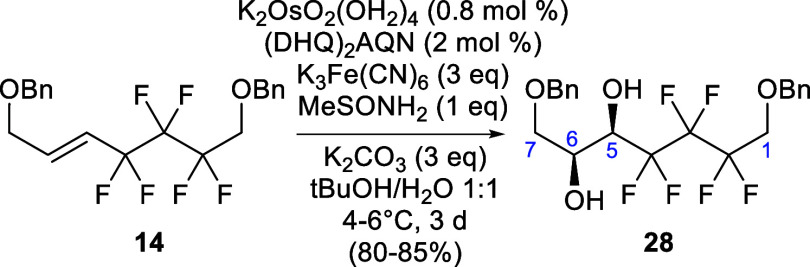



To a stirred solution of (DHQ)_2_AQN (156 mg, 0.18 mmol, 0.02 equiv) in 1:1 tBuOH/H_2_O (80 mL) were sequentially added K_3_Fe(CN)_6_ (8.97 g, 27.2 mmol, 3 equiv), K_2_CO_3_ (3.8 g, 27.2 mmol, 3 equiv), K_2_OsO_2_(H_2_O)_2_ (26 mg, 0.073 mmol, 0.008 equiv) and methanesulfonamide (865 mg, 9.08 mmol, 1 equiv). Mixture was cooled down to 0 °C; a solution of prepared **14** (3.8 g, 9.08 mmol, 1 equiv) in 5 mL of tBuOH was added, and the reaction was left stirred with the cold bath (4–6 °C). After 3 days, near completion was confirmed by TLC (7:3 Hex/AcOEt, R_f_ (**14**) = 0.65, *R*_*f*_ (**28**) = 0.35) and Na_2_S_2_O_3_ (7 g) was added to the mixture which was further stirred during 1 h. Then, water (30 mL) was added, and the aqueous layer was extracted with Et_2_O (3 × 100 mL). The combined organic layer was dried over MgSO_4_, filtered, and the filtrate was concentrated under reduced pressure. The obtained crude product was purified by silica gel chromatography (80:20 to 65:35 Hex/AcOEt) to afford **28** (3.3 g, 82%) as a white solid. Enantiomeric ratio of 95:5 was determined by chiral HPLC-MS: Chiralcel OD-H column, Hex/EtOH 9:1, 1 mL/min, 35 °C, UV(254 nm) detection, rt(A) = 9.3 min (4.6%), rt(B) = 10.2 min (95.4%). **28** (1.95 g) was then suspended in hexane (40 mL) and heated to 40 °C, followed by addition of Et_2_O (5 mL). Slow evaporation at room temperature over 5 days afforded crystalline solids (1.83 g, 94% recovery) that were analyzed by X-ray. Enantiopurity (>99.5% *ee*) of the obtained crystals was confirmed by chiral high-performance liquid chromatography-mass spectrometry analysis. **mp** < 50 °C (obtained after crystallization); **[α]**_**D**_^**23**^ + 1.2 (*c* 0.5, CHCl_3_); ^**1**^**H NMR** (400 MHz, DMSO-*d*_6_): δ 7.40–7.25 (m, 10H, H–Ar), 5.76 (d, *J* = 8.9 Hz, 1H, OH), 5.02 (d, *J* = 7.0 Hz, 1H, OH), 4.65 (s, 2H, –CH_2_Ph), 4.50 (AB system, *J* = 12.1 Hz, 2H, –CH_2_Ph), 4.15–4.00 (m, 3H, H-1, H-5), 3.96 (m, 1H, H-6), 3.50 (d, *J* = 9.2, 7.8 Hz, 1H, H-7a), 3.38 (dd, *J* = 9.4, 5.8 Hz, 1H, H-7b) ppm; ^**1**^**H{**^**19**^**F} NMR** (500 MHz, DMSO-*d*_6_): δ 7.40–7.25 (m, 10H, H–Ar), 5.76 (d, *J* = 9.0 Hz, 1H, OH), 5.02 (d, *J* = 7.1 Hz, 1H, OH), 4.65 (s, 2H, –CH_2_Ph), 4.50 (AB system, *J* = 12.1 Hz, 2H, –CH_2_Ph), 4.09 (dd, *J* = 9.0, 1.5 Hz, 1H, H-5), 4.06 (AB system, *J* = 12.6 Hz, 2H, H-1), 3.96 (m, 1H, H-6), 3.50 (d, *J* = 9.2, 7.8 Hz, 1H, H-7a), 3.38 (dd, *J* = 9.4, 5.8 Hz, 1H, H-7b) ppm; ^**19**^**F NMR** (376 MHz, DMSO-*d*_6_): δ −118.8 (dm, *J* = 271.8 Hz, 1F), −119.1 (dm, *J* = 278.1 Hz, 1F), −119.5 (dm, *J* = 272.4 Hz, 1F), −123.4 (dm, *J* = 279.5, 10.7, 8.6 Hz, 1F), −124.3 (br s, 2F) ppm; ^**19**^**F{**^**1**^**H} NMR** (471 MHz, DMSO-*d*_6_): δ −118.8 (ddd, *J* = 272.5 Hz, 1F), −119.0 (d of br t, *J* = 279.6, 11.4 Hz Hz, 1F), −119.5 (d of br t, *J* = 272.4, 10.0 Hz, 1F), −123.2 (d of br t, *J* = 279.5, 6.4 Hz, 1F), −124.2 (br s, 2F) ppm; ^**13**^**C{**^**1**^**H} NMR** (101 MHz, DMSO-*d*_6_): δ 138.3 (s, C–Ar), 137.2 (s, C–Ar), 128.4 (s, C–Ar), 128.3 (s, C–Ar), 127.9 (s, C–Ar), 127.7 (s, C–Ar), 127.5 (s, C–Ar), 120.5–110.5 (3× CF_2_), 73.3 (s, –CH_2_Ph), 72.2 (s, –CH_2_Ph), 70.1 (s, C-7), 66.9 (dd, *J* = 25.3, 19.4 Hz, C-5), 66.6 (s, C-6), 66.5 (t, *J* = 23.1 Hz, C-1) ppm; **HRMS** (ESI^–^) *m*/*z*: [M – H]^−^ calcd for C_21_H_21_F_6_O_4_, 451.1349; found, 451.1356.

#### (2*S*,3*R*)-4,4,5,5,6,6-Hexafluoroheptane-1,2,3,7-tetraol (**29**)

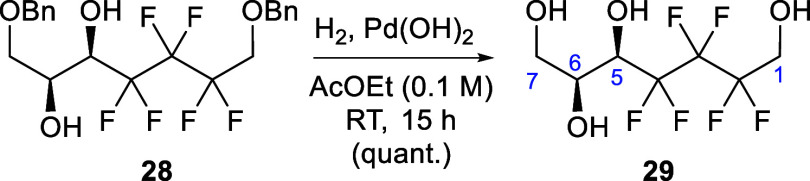



To a stirred and argon-flushed solution of **28** (3 g, 7.05 mmol, 1 equiv) in AcOEt (70 mL) was added Pd(OH)_2_ (600 mg, 20%_w/w_) and three vacuum/argon cycles were performed, followed by three vacuum/H_2_ cycles. The mixture was stirred at room temparature under a positive H_2_ atmosphere. After 15 h, completion was confirmed by TLC (9:1 DCM/MeOH, *R*_*f*_ (**28**) = 0.85, *R*_*f*_ (**29**) = 0.3), mixture was filtrated over a Celite pad, and the latter was washed with MeOH. The obtained filtrate was concentrated under reduced to afford pure **29** (1.9 g, quant.) as a white solid. **mp** 84 °C (obtained after solvent evaporation); **[α]**_**D**_^**23**^ + 21.8 (*c* 0.5, MeOH); ^**1**^**H NMR** (400 MHz, DMSO-*d*_6_): δ 5.84 (br s, *O*H), 5.62 (br s, OH), 4.83 (br s, OH), 4.10 (dd, *J* = 22.4, 5.7 Hz, 1H, H-5), 3.89 (br t, *J* = 16.6 Hz, 2H, H-1), 3.74 (br t, *J* = 6.1 Hz, 1H, H-6), 3.34 (m, 2H, H-7) ppm; ^**1**^**H{**^**19**^**F} NMR** (500 MHz, DMSO-*d*_6_): δ 5.84 (br s, *O*H), 5.51 (br s, OH), 4.79 (br s, OH), 4.10 (br s, 1H, H-5), 3.89 (AB system, *J* = 13.5 Hz, 2H, H-1), 3.74 (br t, *J* = 6.8 Hz, 1H, H-6), 3.34 (m, 2H, H-7) ppm; ^**19**^**F NMR** (376 MHz, DMSO-*d*_6_): δ −118.9 (dm, *J* = 282.4 Hz, 1F), −120.7 (dm, *J* = 266.5 Hz, 1F), −121.6 (dm, *J* = 267.6 Hz, 1F), −123.5 (dm, *J* = 277.8 Hz, 1F), −124.3 (br s, 2F) ppm; ^**19**^**F{**^**1**^**H} NMR** (471 MHz, DMSO-*d*_6_): δ −119.5 (dt, *J* = 278., 12.1 Hz, 1F), −120.6 (ddd, *J* = 266.8, 10.7, 8.6 Hz, 1F), −121.6 (ddd, *J* = 267.5, 12.1, 10.0 Hz, 1F), −123.5 (d of br t, *J* = 278.2, 7.8 Hz, 1F), −124.2 (br s, 2F) ppm; ^**13**^**C{**^**1**^**H} NMR** (101 MHz, DMSO-*d*_6_): δ 120.5–110.5 (3× CF_2_), 68.9 (s, C-6), 66.3 (dd, *J* = 26.1, 20.6 Hz, C-5), 61.4 (s, C-7), 59.0 (t, *J* = 23.5 Hz, C-1) ppm; **HRMS** (ESI^–^) *m*/*z*: [M – H]^−^ calcd for C_7_H_9_F_6_O_4_, 271.0410; found, 271.0419.

#### (2S,3R)-4,4,5,5,6,6-Hexafluoro-1,2-O-isopropylideneheptane-3,7-diol (**16**)

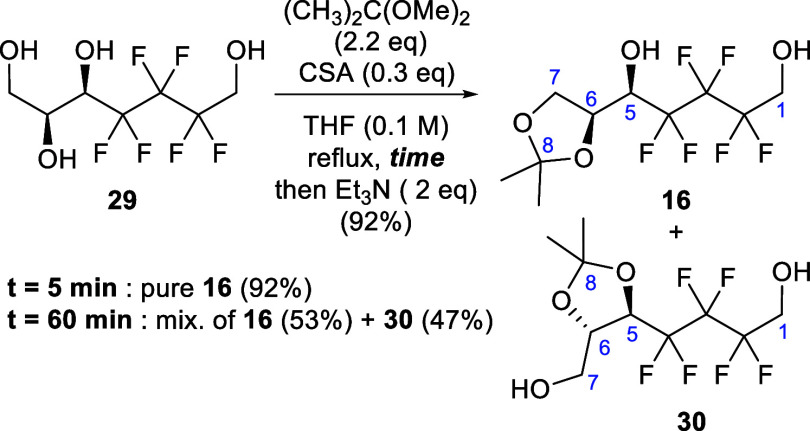



To a stirred solution of **29** (250 mg, 0.91 mmol, 1 equiv) in THF (9 mL) was added 2,2-dimethoxypropane (0.25 mL, 2.00 mmol, 2.2 equiv), and the mixture was refluxed using a heating mantle. Then, camphor sulfonic acid (64 mg, 0.27 mmol, 0.3 equiv) was added. After 5 min stirring at reflux, completion was confirmed by TLC (9:1 DCM/MeOH, *R*_*f*_ (**29**) = 0.3, *R*_*f*_ (**16**) = 0.9; 6:4 Hex/AcOEt, *R*_*f*_ (**29**) = 0, *R*_*f*_ (**16**) = 0.35) which took one more minute, then Et_3_N (0.25 mL, 1.82 mmol, 2 equiv) was added. The reflux was stopped, mixture was cooled down to 30–40 °C and concentrated under reduced pressure. The obtained crude product was purified by silica gel chromatography (60:40 Hex/AcOEt) to afford **16** as a white solid (262 mg, 92%). **mp** 79 °C (obtained after solvent evaporation); **[α]**_**D**_^**23**^ + 6.6 (*c* 0.5, CHCl_3_);^**1**^**H NMR** (400 MHz, DMSO-*d*_6_): 6.05 (d, *J* = 8.4 Hz, 1H, CHOH), 5.86 (t, *J* = 6.6 Hz, 1H, CH_2_OH), 4.26 (m, 1H, H-6), 4.08 (dm, *J* = 22.1 Hz, 1H, H-5), 4.02 (dd, *J* = 8.0, 6.8 Hz, 1H, H-7a), 3.90 (td, *J* = 15.9, 6.6 Hz, 2H, H-1), 3.74 (br t, *J* = 8.0 Hz, 1H, H-7b), 1.35 (s, 3H, CCH_3_), 1.29 (s, 3H, CCH_3_) ppm; ^**1**^**H{**^**19**^**F} NMR** (500 MHz, DMSO-*d*_6_): 6.04 (d, *J* = 8.4 Hz, 1H, CHOH), 5.85 (t, *J* = 6.6 Hz, 1H, CH_2_OH), 4.26 (td, *J* = 7.0, 4.0 Hz, 1H, H-6), 4.08 (dd, *J* = 8.4 Hz, 4.0 Hz, 1H, H-5), 4.02 (dd, *J* = 8.0, 6.8 Hz, 1H, H-7a), 3.90 (AB system, *J* = 7.0 Hz, 2H, H-1), 3.74 (br t, *J* = 8.0 Hz, 1H, H-7), 1.35 (s, 3H, CCH_3_), 1.29 (s, 3H, CCH_3_) ppm; ^**19**^**F NMR** (376 MHz, DMSO-*d*_6_): δ −117.8 (dm, *J* = 279.2 Hz, 1F), −120.3 to −122.0 (m, 2F), −123.4 (dm, *J* = 290.1 Hz, 1F), −123.9 (dm, *J* = 278.9 Hz, 1F), −124.5 (dm, *J* = 290.1 Hz, 1F) ppm; ^**19**^**F{**^**1**^**H} NMR** (471 MHz, DMSO-*d*_6_): δ −117.8 (dm, *J* = 279.2 Hz, 1F), −120.8 (ddd, *J* = 268.2, 12.1, 7.8 Hz, 1F), −121.5 (ddd, *J* = 268.2, 12.8, 9.3, 1F), −123.5 (dm, *J* = 291.1 Hz, 1F), −123.8 (dm, *J* = 278.9 Hz, 1F), −124.4 (dm, *J* = 290.1 Hz, 1F) ppm; ^**13**^**C{**^**1**^**H} NMR** (101 MHz, DMSO-*d*_6_): δ 120.5–110.5 (3× CF_2_), 109.3 (s, C-8), 73.8 (s, C-6), 68.2 (dd, *J* = 26.2, 21.1 Hz, C-5), 65.8 (d, *J* = 5.1 Hz, C-7), 59.4 (t, *J* = 24.7 Hz, C-1), 26.5 (s, CCH_3_), 26.0 (s, CCH_3_) ppm; **HRMS** (ESI^–^) *m*/*z*: [M – H]^−^ calcd for C_10_H_13_F_6_O_4_, 311.0723; found, 311.0735.

When performed during 1 h, two distinguishable (but mostly overlapping) spots are visible on TLC (**30** is slightly more polar than **16**). Silica gel chromatography was performed to isolate the two compounds as a mixture (colorless oil, 82%). NMR spectra comparison with pure **16** confirmed that one of the compounds of the mixture is the terminal acetonide **16** (53%). Observation of the rest of the signals unambiguously led to the conclusion that the second compound is internal acetonide **30** (47%). Details of these observations are provided in Supporting Information Section 3, “Regioselectivity determination for diol isopropylidene protection”. Characterization of **30** (selected signals in the 1:1 mixture with **16**): ^**1**^**H NMR** (400 MHz, DMSO-*d*_6_): 5.92 (t, *J* = 6.4 Hz, 1H, CH_2_OH), 5.11 (t, *J* = 5.7 Hz, 1H, CH_2_OH), 4.49 (doublet of multiplet, *J* = 20.9 Hz, 1H, H-5), 4.33 (m, 1H, H-6), 3.90 (m, 2H, H-1), 3.64 (ddd, *J* = 12.2, 5.3, 3.8 Hz, 1H, H-7a), 3.48 (m, 1H, H-7b), 1.40 (s, 3H, CCH_3_), 1.34 (s, 3H, CCH_3_) ppm; ^**13**^**C{**^**1**^**H} NMR** (101 MHz, DMSO-*d*_6_): δ 120.5–110.5 (3× CF_2_), 112.2 (s, C-8), 77.6 (s, C-6), 73.45 (dd, *J* = 32.7, 21.4 Hz, C-5), 61.4 (s, C-7), 59.4 (t, *J* = 25.4 Hz, C-1), 27.8 (s, CCH_3_), 26.5 (s, CCH_3_) ppm.

#### xy-6,7-O 2,3,4-Trideo-isopropylidene-2,2,3,3,4,4-hexafluoro-l-threo-heptopyranose (l-**31**) and 4,5,6-Trideoxy-1,2-O-isopropylidene-4,4,5,5,6,6-hexafluoro-d-glycero-hept-3-ulopyranose (d-**32**)

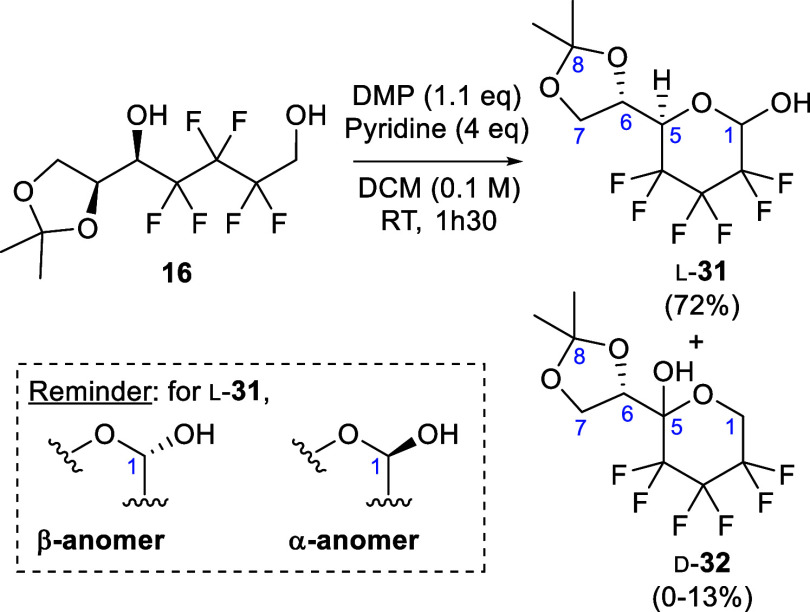



To a stirred solution of **16** (0.98 g, 3.12 mmol, 1 equiv) in dry DCM (30 mL) was added dry pyridine (1 mL, 12.49 mmol, 4 equiv). DMP (292 mg, 0.69 mmol, 0.22 equiv) was added, followed by one more portion (0.22 equiv) every 15 min, up to 1.1 equiv. After 1 h 30 in total, completion was confirmed by TLC (6: Hex/AcOEt, *R*_*f*_ (**16**) = 0.35, *R*_*f*_ (l-**31**) = 0.45), mixture was diluted with DCM (25 mL) and a 1:1 NaHCO_3_/Na_2_S_2_O_3_ saturated solution (25 mL) was added. Mixture was stirred for 10 min, and the aqueous layer was extracted with DCM (2 × 25 mL). The combined organic layer was dried over MgSO_4_, filtered, and concentrated under reduced pressure. The obtained crude was purified by silica gel chromatography (75:25 Hex/AcOEt) to afford l-**31** (700 mg, 72%, 75:25 β/α) as a white solid, and d-**32** (126 mg, 13%) as a colorless oil. Note: d-**32** is not always observed, depending on the batch.

##### Characterization of l-**31** (75:25 β/α Mixture)

**mp** 158 °C (obtained after solvent evaporation); **[α]**_**D**_^**23**^ + 18.6 (*c* 0.5, CHCl_3_);^**1**^**H NMR** (400 MHz, acetone-*d*_6_): 7.46 (br s, 0.75H, OHβ), 5.61 (br t, *J* = 7.2 Hz, 0.75H, H-1β), 5.36 (dd, *J* = 15.8, 3.8 Hz, 0.25H, H-1α), 4.55–4.40 (m, 1.75H, H5β + H6β/α), 4.32–4.19 (m, 0.25H, H-5α), 4.19–4.13 (m, 1H, H-7a-β/α), 3.92 (m, 1H, H-7b-β/α), 1.35 (br s, 3H, CCH_3_), 1.33 (br s, 3H, CCH_3_) ppm; ^**1**^**H{**^**19**^**F} NMR** (500 MHz, acetone-*d*_6_): 7.49 (br d, *J* = 3.9 Hz, 0.75H, OH), 5.61 (d, *J* = 3.8 Hz, 0.75H, H-1β), 5.36 (d, *J* = 7.2 Hz, 0.25H, H-1α), 4.52–4.40 (m, 1.75H, H5β + H6β/α), 4.23 (d, *J* = 5.4 Hz, 0.25H, H-5α), 4.19–4.13 (m, 1H, H-7a-β/α), 3.92 (m, 1H, H-7b-β/α), 1.35 (br s, 3H, CCH_3_), 1.33 (br s, 3H, CCH_3_) ppm; ^**19**^**F NMR** (376 MHz, acetone-*d*_6_): δ −121.8 (dm, *J* = 268.8 Hz, 0.75F, 1Fβ), −124.5 (dm, *J* = 262.7 Hz, 0.75F, 1Fβ), −126.3 (dm, *J* = 270.5 Hz, 0.25F, 1Fα), −128.6 to −130.6 (m, 2F) −131.6 (dm, *J* = 268.8 Hz, 0.75F, 1Fβ), −133.8 (dm, *J* = 260.9 Hz, 0.25F, 1Fα), −140.5 (dm, *J* = 261.4 Hz, 0.25F, 1Fα), −145.0 (dm, *J* = 268.8 Hz, 0.75F, 1Fβ), −146.8 (dm, *J* = 270.5 Hz, 0.25F, 1Fα) ppm; ^**19**^**F{**^**1**^**H} NMR** (471 MHz, acetone-*d*_6_): δ −121.8 (dm, *J* = 268.8 Hz, 0.75F, 1Fβ), −124.5 (dm, *J* = 262.7 Hz, 0.75F, 1Fβ), −126.3 (dm, *J* = 270.5 Hz, 0.25F, 1Fα), −128.6 to −130.6 (m, 2F) −131.6 (dm, *J* = 268.8 Hz, 0.75F, 1Fβ), −133.8 (dm, *J* = 260.9 Hz, 0.25F, 1Fα), −140.5 (dm, *J* = 261.4 Hz, 0.25F, 1Fα), −145.0 (dm, *J* = 268.8 Hz, 0.75F, 1Fβ), −146.8 (dm, *J* = 270.5 Hz, 0.25F, 1Fα); ^**13**^**C{**^**1**^**H} NMR** (101 MHz, acetone-*d*_6_): δ 115.5–105.5 (3× CF_2_), 110.6 (2× s, C-8), 92.8 (ddd, *J* = 27.3, 18.9, 3.7 Hz, C-1α), 92.1 (ddd, *J* = 36.7, 25.7, 1.5 Hz, C-1β), 72.9 (br t, *J* = 24.2 Hz, C-5α), 72.7 (d, *J* = 1.5 Hz, C-6α), 72.5 (br s, C-6β), 67.6 (br t, *J* = 22.2 Hz, C-5β), 66.3 (m, C-7β), 66.2 (m, C-7α), 26.5 (2× s, CCH_3_), 26.0 (s, CCH_3_) ppm; **HRMS** (ESI^–^) *m*/*z*: [M – H]^−^ calcd for C_10_H_11_F_6_O_4_, 309.0567; found, 309.0573.

##### Characterization of d-**32**

^**1**^**H NMR** (400 MHz, CDCl_3_): δ 4.43 (m, 1H, H-6), 4.39 (m, 1H, H-1a), 4.25 (ddd, *J* = 9.2, 4.7, 1.5 Hz, 1H, H-7a), 4.20 (t, *J* = 2.4 Hz, 1H, OH), 4.11 (dd, *J* = 9.1, 6.9 Hz, 1H, H-7b), 4.00 (br td, *J* = 12.5, 5.6 Hz, 1H, H-1b), 1.48 (s, 3H, CCH_3_), 1.42 (s, 3H, CCH_3_) ppm; ^**19**^**F NMR** (376 MHz, CDCl_3_): δ −122.9 (doublet of multiplet, *J* = 268.5 Hz, 1F), −124.8 (doublet of multiplet, *J* = 268.8 Hz, 1F), −126.9 (doublet of multiplet, *J* = 270.5 Hz, 1F), −130.9 (doublet of multiplet, *J* = 267.1 Hz, 1F), −132.1 (doublet of multiplet, *J* = 270.5 Hz, 1F), −145.9 (doublet of multiplet, *J* = 270.5 Hz, 1F) ppm; ^**13**^**C{**^**1**^**H} NMR** (101 MHz, CDCl_3_): δ 115.5–105.5 (3× CF_2_), 110.7 (s, C-8), 95.3 (ddd, *J* = 30.0, 22.8, 2.2 Hz, C-5) 73.7 (s, C-6), 64.85 (s, C-7), 60.1 (ddd, *J* = 32.3, 24.2, 2.2 Hz, C-1), 26.0 (–CCH_3_), 24.9 (–CCH_3_) ppm.

#### Benzyl-2,3,4-trideoxy-6,7-O-isopropylidene-2,2,3,3,4,4-hexafluoro-β-l-threo-heptopyranoside (β-l-**33**) and Benzyl-2,3,4-trideoxy-6,7-O-isopropylidene-2,2,3,3,4,4-hexafluoro-α-l-threo-heptopyranoside (α-l-**33**)

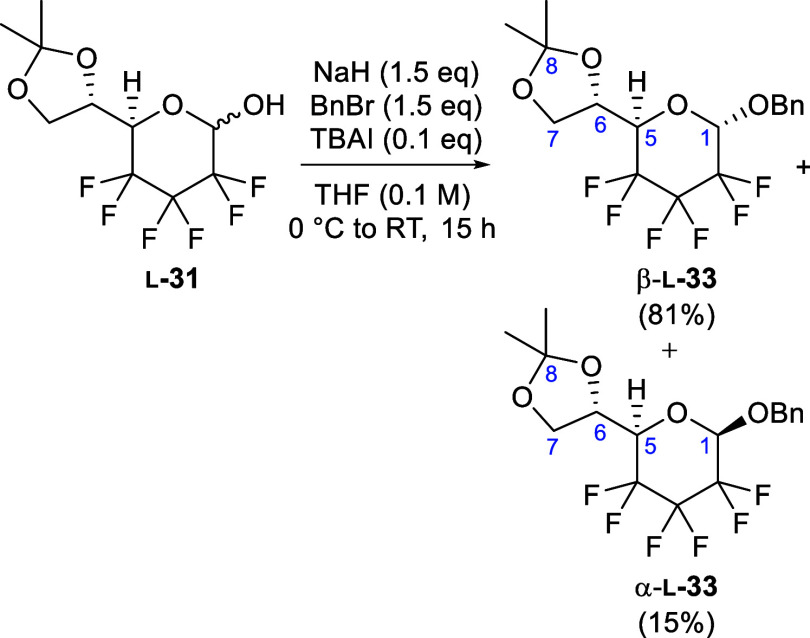



Under inert conditions, a stirred solution of l-**31** (350 mg, 1.13 mmol, 1 equiv) in anhydrous THF (12 mL) was cooled to 0 °C. Then, sodium hydride (68 mg of 60% mineral oil, 1.70 mmol, 1.5 equiv) was added and gas formation was observed during 5 min. Then, the ice bath was removed, TBAI (41 mg, 0.11 mmol, 0.1 equiv) and benzyl bromide (0.2 mL, 1.70 mmol, 1.5 equiv) were added, and the reaction was allowed to stir at room temparature. After 15 h, completion was confirmed by TLC (8:2 Hex/AcOEt, *R*_*f*_ (l-**31**) = 0.2, *R*_*f*_ (β-l-**33**) = 0.5, *R*_*f*_ (α-l-**33**) = 0.45) and the solution was cooled down to 0 °C. Reaction was carefully quenched by dropwise addition of MeOH (5 mL), and the mixture was concentrated under reduced pressure. The obtained crude product was purified by silica gel chromatography (95:5 Hex/AcOEt) to afford β-l-**33** (365 mg, 81%) and α-l-**33** (71 mg, 15%) as white solids.

##### Characterization of β-l-**33**

**mp** 62 °C (obtained after solvent evaporation); **[α]**_**D**_^**23**^ + 67.1 (*c* 0.5, CHCl_3_);^**1**^**H NMR** (400 MHz, CDCl_3_): 7.43–7.34 (m, 5H, H–Ar), 5.14 (ddt, *J* = 7.9, 6.0, 1.3 Hz, 1H, H-1), 4.91 (d, *J* = 12.0 Hz, –CH_a_H_b_Ph), 4.71 (d, *J* = 11.9 Hz, 1H, −CH_a_H_b_Ph), 4.45 (apparent br q, *J* = 6.8 Hz, 1H, H-6), 4.22 (d of br d, *J* = 22.6, 7.6 Hz, 1H, H-5), 4.18 (ddd, *J* = 9.0, 6.1, 1.0 Hz, 1H, H-7a), 3.90 (ddd, *J* = 9.0, 7.3, 2.0 Hz, 1H, H-7b), 1.46 (s, 3H, CCH_3_), 1.45 (s, 3H, CCH_3_) ppm; ^**1**^**H{**^**19**^**F} NMR** (500 MHz, CDCl_3_): 7.43–7.34 (m, 5H, H–Ar), 5.14 (s, 1H, H-1), 4.91 (d, *J* = 12.0 Hz, –CH_a_H_b_Ph), 4.71 (d, *J* = 11.9 Hz, 1H, −CH_a_H_b_Ph), 4.45 (apparent q, *J* = 6.8 Hz, 1H, H-6), 4.22 (d, *J* = 7.7 Hz, 1H, H-5), 4.18 (dd, *J* = 8.9, 6.2 Hz, 1H, H-7a), 3.90 (dd, *J* = 8.9, 7.1 Hz, 1H, H-7b), 1.46 (s, 3H, CCH_3_), 1.45 (s, 3H, CCH_3_) ppm; ^**19**^**F NMR** (376 MHz, CDCl_3_): δ −120.8 (dm, *J* = 273.9 Hz, 1F), −125.4 (dm, *J* = 272.2 Hz, 1F), −129.1 (dm, *J* = 263.6 Hz, 1F), −130.4 (dm, *J* = 263.6 Hz, 1F), −132.1 (dm, *J* = 273.9 Hz, 1F), −145.5 (dm, *J* = 270.5 Hz, 1F) ppm; ^**19**^**F{**^**1**^**H} NMR** (471 MHz, CDCl_3_): δ −120.5 (dtdd, *J* = 273.2, 15.0, 5.7, 1.4 Hz, 1F), −125.1 (dm, *J* = 271.1 Hz, 1F), −128.8 (dm, *J* = 263.6 Hz, 1F), −130.1 (dm, *J* = 263.6 Hz, 1F), −131.9 (ddtd, *J* = 273.9, 15.7, 10.0, 1.4 Hz, 1F), −145.2 (dm, *J* = 270.5 Hz, 1F) ppm; ^**13**^**C{**^**1**^**H} NMR** (101 MHz, CDCl_3_): δ 134.9 (s, C–Ar), 128.8 (s, C–Ar), 128.6 (s, C–Ar), 127.9 (s, C–Ar), 115.5–105.5 (3× CF_2_), 109.9 (s, C-8), 95.4 (ddd, *J* = 37.4, 25.7, 2.2 Hz, C-1), 71.9 (s, C-6), 70.4 (s, –CH_2_Ph), 69.0 (dd, *J* = 25.7, 21.2, 1.5, C-5), 65.5 (s, C-7), 26.4 (–CCH_3_), 25.5 (–CCH_3_) ppm; **HRMS** (ESI^+^) *m*/*z*: [M + H]^+^ calcd for C_17_H_19_F_6_O_4_, 401.1182; found, 401.1183.

##### Characterization of α-l-**33**

**mp** 69 °C (obtained after solvent evaporation); **[α]**_**D**_^**23**^ −58.2 (*c* 0.5, CHCl_3_);^**1**^**H NMR** (400 MHz, CDCl_3_): 7.45–7.34 (m, 5H, H–Ar), 5.05 (d, *J* = 12.0 Hz, –CH_a_H_b_Ph), 4.83 (d, *J* = 12.0 Hz, 1H, −CH_a_H_b_Ph), 4.72 (dd, *J* = 13.8, 3.7 1H, H-1), 4.45 (apparent q, *J* = 6.8 Hz, 1H, H-6), 4.15 (dd, *J* = 8.9, 6.3 Hz, 1H, H-7a), 3.89 (ddd, *J* = 8.9, 6.8, 1.9 Hz, 1H, H-7b), 3.69 (m, 1H, H-5), 1.49 (s, 3H, CCH_3_), 1.43 (s, 3H, CCH_3_) ppm; ^**1**^**H{**^**19**^**F} NMR** (500 MHz, CDCl_3_): 7.45–7.34 (m, 5H, H–Ar), 5.05 (d, *J* = 11.9 Hz, –CH_a_H_b_Ph), 4.83 (d, *J* = 11.9 Hz, 1H, −CH_a_H_b_Ph), 4.72 (s, 1H, H-1), 4.46 (apparent q, *J* = 6.8 Hz, 1H, H-6), 4.15 (dd, *J* = 8.9, 6.3 Hz, 1H, H-7a), 3.89 (dd, *J* = 8.9, 6.8 Hz, 1H, H-7b), 3.69 (d, *J* = 7.2 Hz, 1H, H-5), 1.49 (s, 3H, CCH_3_), 1.43 (s, 3H, CCH_3_) ppm; ^**19**^**F NMR** (376 MHz, CDCl_3_): δ −127.1 (br d, *J* = 274.0 Hz, 1F), −129.5 (dm, *J* = 265.3 Hz, 1F), −130.8 (dm, *J* = 265.3 Hz, 1F), −134.2 (dm, *J* = 261.8 Hz, 1F), −138.7 (dm, *J* = 261.8 Hz, 1F), −146.4 (dm, *J* = 274.0 Hz, 1F) ppm; ^**19**^**F{**^**1**^**H} NMR** (471 MHz, CDCl_3_): −126.9 (apparent d of septuplet, *J* = 274.0, 7.15 Hz, 1F), −129.2 (dm, *J* = 265.3 Hz, 1F), −130.6 (dtd, *J* = 265.3, 14.3, 7.9 Hz, 1F), −134.0 (ddt, *J* = 261.8, 15.0, 9.3 Hz, 1F), −138.5 (ddt, *J* = 261.8, 15.0, 7.1 Hz, 1F), −146.4 (dm, *J* = 274.0 Hz, 1F) ppm; ^**13**^**C{**^**1**^**H} NMR** (101 MHz, CDCl_3_): δ 134.8 (s, C–Ar), 128.7 (s, C–Ar), 128.6 (s, C–Ar), 115.5–105.5 (3× CF_2_), 110.0 (s, C-8), 94.9 (ddd, *J* = 27.1, 18.3, 3.7 Hz, C-1), 73.6 (br dd, *J* = 26.0, 22.4, C-5), 72.1 (s, C-6), 71.5 (s, –CH_2_Ph), 65.5 (2× s, C-7), 26.5 (–CCH_3_), 25.6 (–CCH_3_) ppm; **HRMS** (ESI^+^) *m*/*z*: [M + H]^+^ calcd for C_17_H_29_F_6_O_4_, 401.1182; found, 401.1176.

#### Benzyl-2,3,4-trideoxy-2,2,3,3,4,4-hexafluoro-β-l-threo-heptopyranoside (β-l-**34**)

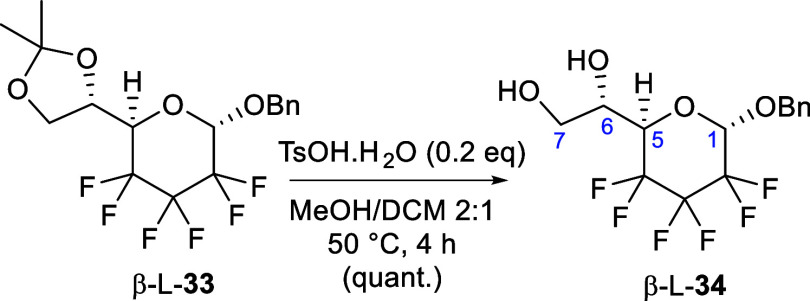



To a stirred solution of β-l-**33** (370 mg, 0.92 mmol, 1 equiv) in 2:1 MeOH/DCM (20 mL) was added PTSA.H_2_O (35 mg, 0.18 mmol, 0.2 equiv) and the reaction was heated to 50 °C using a heating mantle. After 4 h, completion was confirmed by TLC (6:4 Hex/AcOEt, *R*_*f*_ (β-l-**33**) = 0.9, *R*_*f*_ (β-l-**34**) = 0.2), mixture was diluted with 20 mL of DCM and a saturated aqueous solution of NaHCO_3_ (30 mL) was added. Phases were separated, the aqueous layer was extracted with DCM (3 × 30 mL), the combined organic layer was dried over MgSO_4_, filtered, and the filtrate was concentrated under reduced pressure to afford β-l-**34** as a white solid (69 mg, quant.). **mp** 74 °C (obtained after solvent evaporation); **[α]**_**D**_^**23**^ + 76.8 (*c* 0.5, CHCl_3_);^**1**^**H NMR** (400 MHz, CDCl_3_): 7.43–7.34 (m, 5H, H–Ar), 5.14 (ddt, *J* = 7.9, 6.0, 1.3 Hz, 1H, H-1), 4.87 (d, *J* = 11.9 Hz, –CH_a_H_b_Ph), 4.70 (d, *J* = 11.9 Hz, 1H, −CH_a_H_b_Ph), 4.35 (dd, *J* = 24.8, 4.8 Hz, 1H, H-5), 4.19 (apparent q, *J* = 4.9 Hz, 1H, H-6), 3.75 (m, 2H, H-7), 2.48 (br s, 1H, CHOH), 1.98 (br s, 1H, CH_2_OH) ppm; ^**1**^**H{**^**19**^**F} NMR** (500 MHz, CDCl_3_): 7.43–7.34 (m, 5H, H–Ar), 5.14 (s, 1H, H-1), 4.87 (d, *J* = 11.9 Hz, –CH_a_H_b_Ph), 4.70 (d, *J* = 11.9 Hz, 1H, −CH_a_H_b_Ph), 4.35 (d, *J* = 5.0 Hz, 1H, H-5), 4.19 (apparent q, *J* = 4.9 Hz, 1H, H-6), 3.77 (m, 2H, H-7), 2.43 (br s, 1H, CHOH), 1.93 (br s, 1H, CH_2_OH) ppm; ^**19**^**F NMR** (376 MHz, CDCl_3_): δ −120.8 (dm, *J* = 273.9 Hz, 1F), −125.1 (dm, *J* = 272.1 Hz, 1F), −128.7 (dm, *J* = 264.2 Hz, 1F), −130.9 (dm, *J* = 260.8 Hz, 1F), −131.8 (dm, *J* = 273.3 Hz, 1F), −145.2 (dm, *J* = 271.0 Hz, 1F) ppm; ^**19**^**F{**^**1**^**H} NMR** (471 MHz, CDCl_3_): δ −120.7 (dtdd, *J* = 273.2, 16.5, 6.4, 1.4 Hz, 1F), −125.2 (d of apparent br sept, *J* = 272.1, 7.15 Hz, 1F), −128.8 (ddtd, *J* = 264.2, 13.6, 9.3, 1.4 Hz, 1F), −130.9 (dm, *J* = 264.5 Hz, 1F), −131.8 (ddtd, *J* = 273.2, 15.7, 10.0, 1.4 Hz, 1F), −145.2 (dm, *J* = 270.3 Hz, 1F) ppm; ^**13**^**C{**^**1**^**H} NMR** (101 MHz, CDCl_3_): δ 134.8 (C–Ar), 128.8 (C–Ar), 128.7 (C–Ar), 128.0 (C–Ar), 115.5–105.5 (3× CF_2_), 95.6 (ddd, *J* = 37.4, 25.4, 1.8 Hz, C-1), 70.9 (s, –CH_2_Ph), 67.9 (s, C-6), 66.8 (dd, *J* = 27.8, 21.3 Hz, C-5), 62.6 (s, C-7) ppm; **HRMS** (ESI^–^) *m*/*z*: [M – H]^−^ calcd for C_14_H_13_F_6_O_4_, 359.0723; found, 359.0733.

#### Benzyl 2,3,4-Trideoxy-2,2,3,3,4,4-hexafluoro-α-d-glycero-hexopyranoside (α-d-**35**)

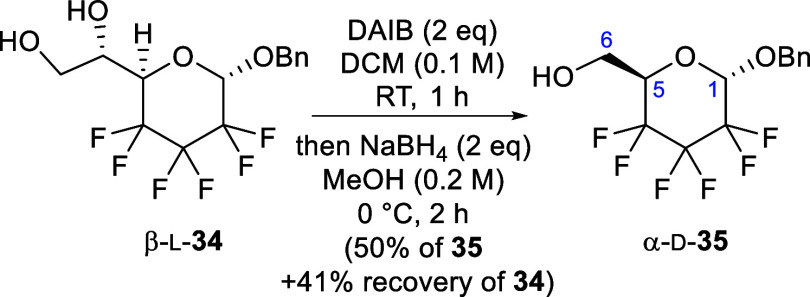



To a stirred solution of β-l-**34** (120 mg, 0.33 mmol, 1 equiv) in DCM (3.5 mL) was added (diacetoxyiodo)benzene (215 mg, 0.66 mmol, 2 equiv). After 1 h at room temperature, the mixture was cooled to 0 °C and diluted with MeOH (1.8 mL) followed by careful addition of NaBH_4_ (100 mg, 2.67 mmol, 8 equiv). After 2 h stirring with the ice bath, the product could be observed by TLC (4:6 Hex/AcOEt, *R*_*f*_ (β-l-**34**) = 0.25, *R*_*f*_ (α-d-**35**) = 0.75) and the reaction was quenched with a saturated aqueous solution of NH_4_Cl (10 mL). The mixture was extracted with Et_2_O (3 × 15 mL) and the combined organic layer was dried over MgSO_4_, filtered, and the filtrate was concentrated under reduced pressure. The obtained crude was purified by silica gel chromatography (70:30 to 50:50 Hex/AcOEt) to afford α-d-**35** as a colorless oil (55 mg, 50%) and recover β-l-**34** (49 mg, 41%). **[α]**_**D**_^**23**^ + 95.2 (*c* 0.5, CHCl_3_);^**1**^**H NMR** (400 MHz, acetone-*d*_6_): 7.46–7.33 (m, 5H, H–Ar), 5.45 (ddt, *J* = 7.9, 6.0, 1.3 Hz, 1H, H-1), 4.95 (d, *J* = 11.8 Hz, –CH_a_H_b_Ph), 4.79 (d, *J* = 11.8 Hz, 1H, −CH_a_H_b_Ph), 4.47 (dd, *J* = 6.6, 5.9 Hz, −CH_2_OH), 4.44 (m, 1H, H-5), 4.01 (dddd, *J* = 12.2, 6.7, 3.4, 1.1 Hz, 1H, H-6a), 3.88 (br dt, *J* = 12.2, 6.6, 1H, H-6b) ppm; ^**1**^**H{**^**19**^**F} NMR** (500 MHz, acetone-*d*_6_): 7.46–7.33 (m, 5H, H–Ar), 5.45 (s, 1H, H-1), 4.95 (d, *J* = 11.8 Hz, –CH_a_H_b_Ph), 4.79 (d, *J* = 11.8 Hz, 1H, −CH_a_H_b_Ph), 4.44 (dd, *J* = 7.3, 3.6 Hz, 1H, H-5), 4.40 (br t, *J* = 6.2 Hz, −CH_2_OH), 4.01 (ddd, *J* = 12.2, 6.7, 3.4 Hz, 1H, H-6a), 3.88 (ddd, *J* = 12.2, 7.3, 6.6 Hz 1H, H-6b) ppm; ^**19**^**F NMR** (376 MHz, acetone-*d*_6_): δ −120.6 (dm, *J* = 272.5 Hz, 1F), −124.9 (dm, *J* = 269.0 Hz, 1F), −130.6 (dm, *J* = 263.7 Hz, 1F), −132.0 (dm, *J* = 272.5 Hz, 1F), −132.3 (dm, *J* = 263.7 Hz, 1F), −145.5 (dm, *J* = 269.0 Hz, 1F) ppm; ^**19**^**F{**^**1**^**H} NMR** (471 MHz, acetone-*d*_6_): δ −120.6 (dtdd, *J* = 272.5, 15.5, 7.17, 1.7 Hz, 1F), −124.9 (ddddd, *J* = 269.0, 15.5, 12.9, 7.5, 6.9 Hz, 1F), −130.6 (dm, *J* = 263.8 Hz, 1F), −132.0 (ddtd, *J* = 272.5, 15.5, 7.8, 2.0 Hz, 1F), −132.3 (ddddd, *J* = 263.7, 16.0, 12.6, 7.5, 2.0 Hz, 1F), −145.5 (ddddd, *J* = 269.0, 15.5, 12.6, 9.8, 8.3 Hz, 1F) ppm; ^**13**^**C{**^**1**^**H} NMR** (101 MHz, acetone-*d*_6_): δ 136.9 (C–Ar), 129.5 (C–Ar), 129.3 (C–Ar), 129.1 (C–Ar), 115.5–105.5 (3× CF_2_), 96.4 (ddd, *J* = 36.7, 24.9, 2.2 Hz, C-1), 71.3 (s, –CH_2_Ph), 70.1 (ddd, *J* = 25.7, 22.0, 1.5 Hz, C-5), 58.4 (m, C-6) ppm; **HRMS** (ESI^+^) *m*/*z*: [M + Na]^+^ calcd for C_13_H_12_F_6_O_3_Na, 353.0583; found, 353.0581

#### 2,3,4-Trideoxy-2,2,3,3,4,4-hexafluoro-d-glycero-hexopyranose (d-**4**)

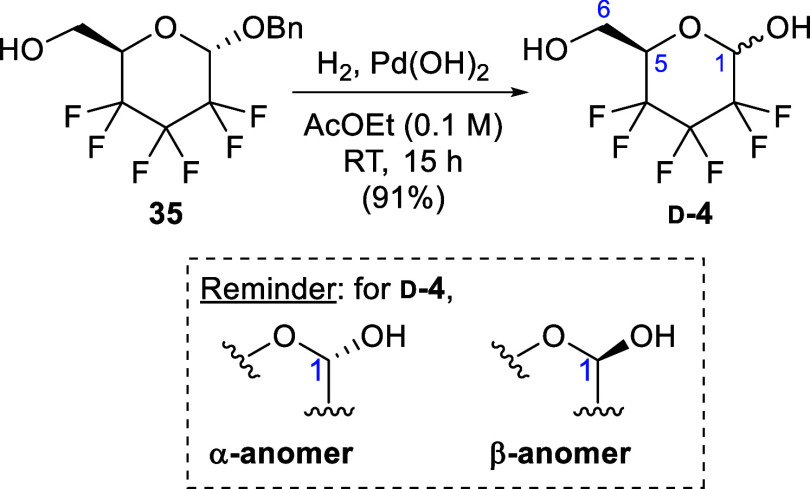



To a stirred and argon-flushed solution of prepared α-d-**35** (130 mg, 0.40 mmol, 1 equiv) in AcOEt (4 mL) was added Pd(OH)_2_ (25 mg, 20%_w/w_). Three vacuum–argon cycles were performed, followed by three vacuum – H_2_ cycles. Mixture was left stirred at room temperature under a positive H_2_ atmosphere. After 15 h, completion was confirmed by TLC (5:5 Hex/AcOEt, *R*_*f*_ (α-d-**35**) = 0.85, *R*_*f*_ (d-**4**) = 0.45), mixture was filtrated over a Celite pad, and the latter was washed with AcOEt and MeOH. The obtained filtrate was concentrated under reduced to afford almost pure d-**4** (94 mg, quant.). The latter can be purified by silica gel chromatography (50:50 Hex/AcOEt) to increase the purity. This afforded d-**4** (84 mg, 91%) as colorless oil. Twenty milligram of the sugar were taken and 1.5 mL of Et_2_O/3 mL of hexane were added. Slow evaporation (1 week) afforded crystalline solids that were analyzed by X-ray. **mp** 64 °C (obtained from crystalline compound); **[α]**_**D**_^**23**^ + 115.3 (*c* 0.5, MeOH), ^**1**^**H NMR** (500 MHz, acetone-*d*_6_): δ 7.47 (br s, 1H, OH-1αβ), 5.53 (br t, *J* = 7.2 Hz, 0.7H, H-1α), 5.30 (dd, *J* = 15.9, 3.8, 0.3H, H-1β), 4.52 (dm, *J* = 24.7 Hz, 0.7H, H-5α), 4.45 (br s, 0.3H, OH-6β), 4.27 (br s, 0.7H, OH-6α), 4.18 (dm, *J* = 23.5, 0.3H, H-5β), 3.98 (m, 1H, H-6a-αβ), 3.81 (m, 1H, H-6b-αβ) ppm; ^**1**^**H{**^**19**^**F} NMR** (500 MHz, acetone-*d*_6_): δ 7.46 (m, 1H, OH-1αβ), 5.53 (s, 0.7H, H-1α), 5.30 (s, 0.3H, H-1β), 4.52 (dd, *J* = 7.0, 3.6 Hz, 0.7H, H-5α), 4.44 (br s, 0.3H, OH-6β), 4.34 (br s, 0.7H, OH-6α), 4.17 (dd, *J* = 7.0, 3.6 Hz, 0.3H, H-5β), 3.96 (m, 1H, H-6a-αβ), 3.81 (m, 1H, H-6b-αβ) ppm; ^**19**^**F NMR** (376 MHz, acetone-*d*_6_): δ −122.7 (dm, *J* = 268.6 Hz, 0.7F, Fα), −125.4 (dm, *J* = 269.3 Hz, 0.7F, Fα), −127.3 (dm, *J* = 270.7 Hz, 0.3F, Fβ), −131.3 (dm, *J* = 263.2 Hz, 0.7F, Fα), −132.4 (dd br t, *J* = 264.6, 13.6, 9.3 Hz, 0.3F, Fβ), −132.7 (dd br t, *J* = 268.2, 16.1, 9.7 Hz, 0.7F, Fα), −133.3 (dm, *J* = 263.0 Hz, 0.7F, Fα), −133.9 (ddd br d, *J* = 264.2, 16.9, 12.9, 7.9 Hz, 0.3F, Fβ), −134.8 (ddtd, *J* = 260.9, 15.4, 9.3, 1.0 Hz, 0.3F, Fβ), −141.4 (dm, *J* = 261.9 Hz, 0.3F, Fβ), −145.9 (ddddd, *J* = 269.3, 15.4, 12.5, 9.7, 8.3 Hz, 0.7F, Fα), −147.6 (dddt, *J* = 271.0, 23.2, 13.9, 9.3 Hz, 0.3F, Fβ) ppm; ^**19**^**F{**^**1**^**H} NMR** (471 MHz, acetone-*d*_6_): δ −122.7 (dtdd, *J* = 268.6, 16.4, 7.2, 1.4 Hz, 0.7F, Fα), −125.4 (dddt, *J* = 269.3, 16.5, 13.2, 7.5 Hz, 0.7F, Fα), −127.3 (dddt, *J* = 270.7, 15.7, 14.3, 7.9 Hz, 0.3F, Fβ), −131.3 (dm, *J* = 263.2 Hz, 0.7F, Fα), −132.4 (ddtd, *J* = 264.6, 13.9, 9.3, 1.0 Hz, 0.3F, Fβ), −132.7 (ddtd, *J* = 268.2, 16.1, 9.7, 1.4 Hz, 0.7F, Fα), −133.3 (dm, *J* = 263.0 Hz, 0.7F, Fα), −133.9 (dddd, *J* = 264.2, 15.4, 8.2, 1.4 Hz, 0.3F, Fβ), −134.8 (ddtd, *J* = 260.9, 15.4, 9.3, 1.0 Hz, 0.3F, Fβ), −141.4 (dddd, *J* = 261.9, 14.9, 7.5, 1.4 Hz, 0.3F, Fβ), −145.9 (ddddd, *J* = 269.3, 15.4, 12.5, 9.7, 8.3 Hz, 0.7F, Fα), −147.6 (dddt, *J* = 271.0, 23.2, 13.9, 9.3 Hz, 0.3F, Fβ) ppm; ^**13**^**C{**^**1**^**H} NMR** (101 MHz, acetone-*d*_6_): δ 115–110 (3× CF_2_), 92.5 (ddd, *J* = 28.9, 18.9, 4.0 Hz, C-1β), 91.9 (br dd, *J* = 35.9, 25.1 Hz, C-1α), 74.4 (dd, *J* = 26.2, 22.4 Hz, C-5β), 69.3 (ddd, *J* = 25.4, 21.4, 1.5 Hz, C-5α), 58.6 (dd, *J* = 4.7, 2.2 Hz, C-6β), 58.5 (dd, *J* = 4.7, 1.8 Hz, C-6α) ppm; **HRMS** (ESI^–^) *m*/*z*: [M – H]^−^ calcd for C_6_H_5_F_6_O_3_, 239.0148; found, 239.0154.

#### 2,3,4-Trideoxy-2,2,3,3,4,4-hexafluoro-l-threo-heptopyranose (l-**18**)

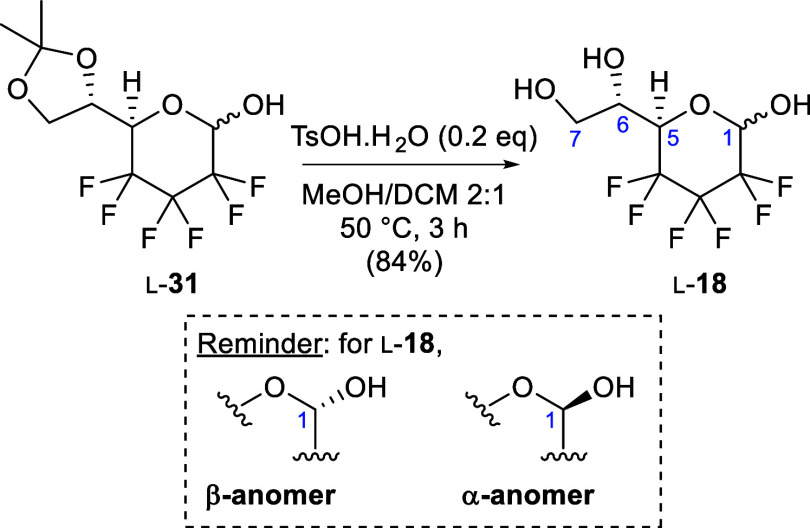



To a stirred solution of l-**31** (75 mg, 0.24 mmol, 1 equiv) in 2:1 MeOH/DCM (5 mL) was added PTSA.H_2_O (9 mg, 0.05 mmol, 0.2 equiv) and the reaction was heated to 50 °C using a heating mantle. After 3 h, completion was confirmed by TLC (4:6 Hex/AcOEt, R_f_ (l-**31**) = 0.8, R_f_ (l-**18**) = 0.15) and a saturated aqueous solution of NaHCO_3_ (10 mL) was added. The solution was extracted with DCM (2 × 20 mL), the combined organic layer was dried over MgSO_4_, filtered, and the filtrate was concentrated under reduced pressure to afford almost pure l-**18** (65 mg, quant) as a colorless oil. The latter can be purified by silica gel chromatography (10:90 Hex/AcOEt) to increase purity. This afforded l-**18** (55 mg, 84%) as a colorless oil. **[α]**_**D**_^**23**^ + 105.7 (*c* 0.5, MeOH);^**1**^**H NMR** (400 MHz, acetone-*d*_6_): 7.44 (br s, 1H, OH-1αβ), 5.57 (br t, *J* = 7.1 Hz, 0.7H, H-1β), 5.29 (dd, *J* = 15.9, 3.9 Hz, 0.3H, H-1α), 4.62 (br d, *J* = 25.6 Hz, 0.7H, H-5β), 4.26 (br s, 0.3H, OH-6α), 4.22–3.95 (m, 3.3H, H-5α (0.3H) + H-6αβ (1H) + OH-6β (1H) + OH-7αβ (1H)), 3.71–3.58 (m, 2H, H-7α/β) ppm; ^**1**^**H{**^**19**^**F} NMR** (500 MHz, acetone-*d*_6_): δ 7.43 (br s, 1H, OH-1αβ), 5.56 (s, 0.7H, H-1β), 5.29 (s, 0.3H, H-1α), 4.62 (d, *J* = 3.0 Hz, 0.7H, H-5β), 4.25 (br s, 0.3H, OH-3α), 4.16 (d, *J* = 3.4 Hz, 0.3H, H-5α), 4.18–3.95 (m, 3H, H-6αβ + OH-6β + OH-7αβ), 3.71–3.58 (m, 2H, H-7α/β) ppm; ^**19**^**F NMR** (376 MHz, acetone-*d*_6_): δ −122.7 (dtdd, *J* = 268.2, 16.1, 7.5, 2.1 Hz, 0.7F, Fα), −125.4 (dddtd, *J* = 267.8, 15.4, 12.9, 7.5, 2.5 Hz, 0.7F, Fα), −127.3 (dm, *J* = 269.7 Hz, 0.3F, Fβ), −130.3 (d of br q, *J* = 261.1, 10.0 Hz, 0.7F, Fα), −131.4 (dm, *J* = 262.9 Hz, 0.3F, Fβ), −131.6 (dm, *J* = 268.2, 0.7F, Fα), −132.2 (m, 0.3F, Fβ), −132.3 (dd of br t, *J* = 268.2, 16.1, 9.7 Hz, 0.7F, Fα), −134.6 (ddtd, *J* = 260.7, 15.4, 9.3, 2.1 Hz, 0.3F, Fβ), −141.2 (dm, *J* = 260.7 Hz, 0.3F, Fβ), −145.8 (dm, *J* = 267.5 Hz, 0.7F, Fα), −147.6 (dddt, *J* = 271.0, 14.7, 12.9, 9.7 Hz, 0.3F, Fβ) ppm; ^**19**^**F{**^**1**^**H} NMR** (471 MHz, acetone-*d*_6_): δ −122.7 (dtdd, *J* = 268.2, 16.1, 7.5, 2.1 Hz, 0.7F, Fα), −125.4 (dddt, *J* = 267.8, 15.4, 12.9, 7.5 Hz, 0.7F, Fα), −127.3 (ddddd, *J* = 269.3, 15.8, 10.7, 7.5, 5.4 Hz, 0.3F, Fβ), −130.3 (ddtd, *J* = 261.1, 14.3, 9.6, 1.4 Hz, 0.7F, Fα), −131.4 (dtdd, *J* = 262.9, 10.7, 8.6, 3.2 Hz, 0.3F, Fβ), −131.6 (ddddd, *J* = 268.2, 16.5, 12.1, 7.5, 1.8 Hz, 0.7F, Fα), −132.2 (m, 0.3F, Fβ), −132.3 (ddtd, *J* = 268.2, 16.1, 9.7, 1.8 Hz, 0.7F, Fα), −134.6 (ddtd, *J* = 260.7, 15.4, 9.3, 2.1 Hz, 0.3F, Fβ), −141.2 (dtdd, *J* = 260.7, 14.7, 7.9, 3.6 Hz, 0.3F, Fβ), −145.8 (ddddd, *J* = 267.5, 15.4, 11.8, 9.6, 8.3 Hz, 0.7F, Fα), −147.6 (dddt, *J* = 271.0, 14.7, 12.9, 9.7 Hz, 0.3F, Fβ) ppm; ^**13**^**C{**^**1**^**H} NMR** (101 MHz, acetone-*d*_6_): δ 115.5–105.5 (3× CF_2_), 92.9 (ddd, *J* = 27.3, 19.2, 3.6 Hz, C-1α), 92.1 (ddd, *J* = 36.5, 27.9, 2.2 Hz, C-1β), 71.6 (ddd, *J* = 25.8, 22.2, 1.5 Hz, C-5β), 69.0 (s, C-6α), 68.9 (s, C-6β), 66.2 (ddd, *J* = 26.8, 20.7, 1.8 Hz, C-5α), 62.9 (d, *J* = 1.5 Hz, C-7β), 62.8 (d, *J* = 1.8 Hz, C-7α) ppm; **HRMS** (ESI^–^) *m*/*z*: [M – H]^−^ calcd for C_7_H_7_F_6_O_4_, 269.0254; found, 269.0256.

## Data Availability

The data underlying this study are available in the published article and its Supporting Information.

## Supporting Information Available

The Supporting Information includes the additional discussions mentioned in text (nomenclature, determination of enantioselectivity, regioselectivity, ring-size), and NMR spectra of the described compounds (PDF)

## Dedication

Dedicated to Prof. Dennis Curran on the occasion of his 70th birthday.
