# Supplementary material for: De Novo Enantioselective Synthesis of Hexafluorinated d-Glucose
Source: J Org Chem. 2024 Sep 13;89(19):14291–304. doi: 10.1021/acs.joc.4c01724 (PMC11460824; doi:10.1021/acs.joc.4c01724)
Supplement: Supplementary file 1 — jo4c01724_si_001.pdf [file jo4c01724_si_001.pdf]

SUPPORTING INFORMATION

De Novo Enantioselective Synthesis of "Hexafluorinated D-Glucose"

Sébastien Depienne,[a] Clément Q. Fontenelle,[b] Mark E. Light,[b] Kristof Van Hecke,[c] and Bruno Linclau[a,b,*]

[a] *Department of Organic and Macromolecular Chemistry, Ghent University, Campus Sterre, Krijgslaan 281-S4, 9000 Ghent, Belgium*

[b] *School of Chemistry, University of Southampton, Highfield, Southampton, SO17 1BJ, UK*

[c] *Department of Chemistry, Ghent University, Campus Sterre, Krijgslaan 281-S3, 9000 Ghent, Belgium*

bruno.linclau@ugent.be

Table of contents

Contents

1 Nomenclature of the sugar derivatives........ SS3
2 Enantioselectivity determination for Sharpless asymmetric dihydroxylation of (*E*)-14SS5
3 Regioselectivity determination for diol protection on tetraol 29........ SS6
3.1 Kinetic control: 5 min reaction........ SS6
3.2 Longer experiment: 60 min reaction........ SS9
4 Determination of the ^{13}C chemical shifts of the CF_2-groups using HMBC analysis........ S13
5 Determination of the size of the heterocyclic ring for hexafluorinated hexose D-4 and heptose L-18........ S14
6 NMR spectra for novel compounds........ S16
6.1 NMR spectra of 3,3,4,4,5,5-hexafluorooxane-2-ol (20)........ S16
6.2 NMR spectra of (*rac*)-methyl 2-(3,3,4,4,5,5-hexafluorooxan-2-yl)acetate (22)........ S19
6.3 NMR spectra of 5-(benzyloxy)-2,2,3,3,4,4-hexafluoropentan-1-ol (23)........ S22
6.4 NMR spectra of 1,5-bis(benzyloxy)-2,2,3,3,4,4-hexafluoropentane (S1)........ S25
6.5 NMR spectra of 5-(benzyloxy)-2,2,3,3,4,4-hexafluoropentane-1,1-diol (25)........ S28
6.6 NMR spectra of methyl (*E*)-7-(benzyloxy)-4,4,5,5,6,6-hexafluorohept-2-enoate (26). S31
6.7 NMR spectra of (*E*)-7-(benzyloxy)-4,4,5,5,6,6-hexafluorohept-2-en-1-ol (27)........ S34
6.8 NMR spectra of (*E*)-1,7-bis(benzyloxy)-4,4,5,5,6,6-hexafluorohept-2-ene (14)........ S37

6.9 NMR spectra of (2*S*,3*R*)-1,7-bis(benzyloxy)-4,4,5,5,6,6-hexafluoroheptane-2,3-diol (28). S40

6.10 NMR spectra of (2*S*,3*R*)-4,4,5,5,6,6-hexafluoroheptane-1,2,3,7-tetraol (29) S43

6.11 NMR spectra of (2*S*,3*R*)-4,4,5,5,6,6-hexafluoro-1,2-*O*-isopropylideneheptane-3,7-diol (16) S46

6.12 NMR spectra of 2,3,4-trideoxy-6,7-*O*-isopropylidene-2,2,3,3,4,4-hexafluoro-L-*threo*-heptopyranose (L-31) S49

6.13 NMR spectra of 4,5,6-trideoxy-1,2-*O*-isopropylidene-4,4,5,5,6,6-hexafluoro-D-*glycero*-hept-3-ulopyranose (D-32) S52

6.14 NMR spectra of benzyl-2,3,4-trideoxy-6,7-*O*-isopropylidene-2,2,3,3,4,4-hexafluoro-β-L-*threo*-heptopyranoside (β-L-33) S54

6.15 NMR spectra of benzyl-2,3,4-trideoxy-6,7-*O*-isopropylidene-2,2,3,3,4,4-hexafluoro-α-L-*threo*-heptopyranoside (α-L-33) S57

6.16 NMR spectra of benzyl-2,3,4-trideoxy-2,2,3,3,4,4-hexafluoro-β-L-*threo*-heptopyranoside (β-L-34) S60

6.17 NMR spectra of benzyl 2,3,4-trideoxy-2,2,3,3,4,4-hexafluoro-α-D-*glycero*-hexopyranoside (α-D-35) S63

6.18 NMR spectra of 2,3,4-trideoxy-2,2,3,3,4,4-hexafluoro-D-*glycero*-hexopyranose (D-4) S66

6.19 NMR spectra of 2,3,4-trideoxy-2,22,3,3,4,4-hexafluoro-L-*threo*-heptopyranose (L-18) S69

7 Crystallographic data S72

7.1 (2*S*,3*R*)-1,7-bis(benzyloxy)-4,4,5,5,6,6-hexafluoroheptane-2,3-diol (28) S72

7.2 (2*S*,3*R*)-4,4,5,5,6,6-hexafluoro-1,2-*O*-isopropylideneheptane-3,7-diol (16) S73

7.3 2,3,4-trideoxy-6,7-*O*-isopropylidene-2,2,3,3,4,4-hexafluoro-L-*threo*-heptopyranose (L-31) S74

7.4 2,3,4-trideoxy-2,2,3,3,4,4-hexafluoro-D-*glycero*-hexopyranose (D-4) S75

8 References S76

1 Nomenclature of the sugar derivatives

The nomenclature of sugars derivatives having less or more chiral stereocenters and/or carbon atoms than classical pentose or hexose carbohydrates is nontrivial. This section is included in order to ensure consistent naming of the derivatives of this work (**31**, **32**, **33**, **34**, **35**, **4** and **18**). Several considerations were taken into account:

- **Configurational prefix**:
  Regardless of the size of the ring, the configurational prefix refers to both a number of stereocenters (without considering the anomeric position) and their configuration. For instance, ***gluco***- is the configurational prefix of sugar having four stereocenters (position 2, 3, 4 and 5) with the well-known combination of configurations of glucose. The derivatives of this work are difluorinated in position 2, 3 and 4, which thus are not chiral centers. The derivatives have one or two stereocenters (excluding anomeric position) and their configurational prefix thus is ***glycero***- (one stereocenter), ***threo***- (two stereocenters, substituents in opposite sides in Fischer projection) or ***erythro-*** (two stereocenters, substituents in same sides in Fischer projection). At this stage, derivative **31** thus is a *threo*-heptopyranose (**Fig. S1**). *Note*: the maximum number of stereocenters for one configurational prefix is four (*e.g.* glucose, galactose). For a sugar having more than four stereocenters, a combination of two configurational prefix is used (*e.g. glycero-galacto* for sialic acid).

- **D- or L- configuration**:
  Each configurational prefix has a D- and L- designation. This refers to the position of the hydroxyl group of their **last chiral center** in Fischer projection (highest numbered stereocenter). For an OH-group on the right-hand side, it is designated by D-, and for an OH-group on the left-hand side, by L-. Derivative **33** thus is a L-*threo*-heptopyranoside (**Fig. S1**).

- **α or β anomeric configuration**:
  This refers to the orientation of the anomeric substituent as compared to the orientation of a reference substituent, in Fischer projection. If both the anomeric substituent and the reference substituent are on same side, the anomeric configuration is α. If the substituents are on opposite sides, it is β. The reference atom to be considered is the last chiral center of the configurational prefix involved in the heterocyclic ring. Derivate **34** is thus a β-L-*threo*-heptopyranose because anomeric carbon bears a benzyloxy group in axial position (right-hand side in the Fischer projection), as opposed to the substituent on carbon six (the last chiral center of the L-*threo* part) being on the left-hand side (**Fig. S1**).

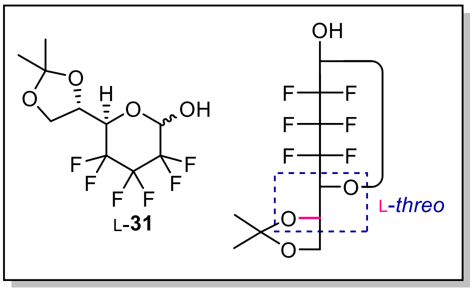

31: 2,3,4-trideoxy-6,7-*O*-isopropylidene-2,2,3,3,4,4-hexafluoro-L-*threo*-heptopyranose

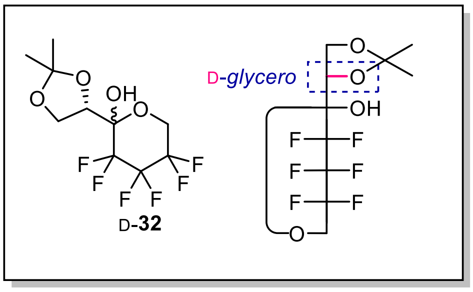

D-**32**: 4,5,6-trideoxy-1,2-*O*-isopropylidene-4,4,5,5,6,6-hexafluoro-D-*glycero*-hept-3-ulopyranose

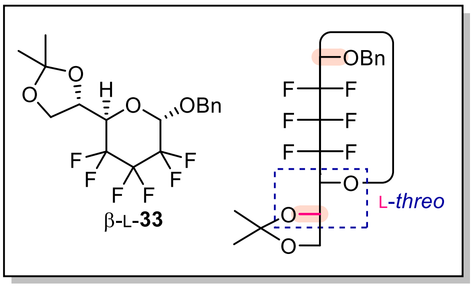

β-L-**33**: benzyl-2,3,4-trideoxy-6,7-*O*-isopropylidene-2,2,3,3,4,4-hexafluoro-β-L-*threo*-heptopyranoside

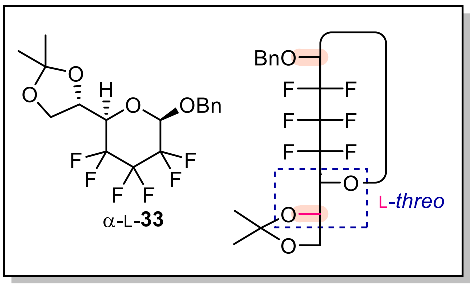

α-L-**33**: benzyl-2,3,4-trideoxy-6,7-*O*-isopropylidene-2,2,3,3,4,4-hexafluoro-α-L-*threo*-heptopyranoside

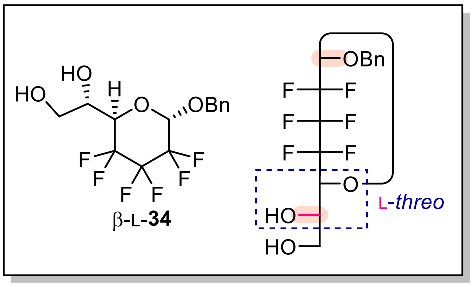

β-L-**34**: benzyl-2,3,4-trideoxy-2,2,3,3,4,4-hexafluoro-β-L-*threo*-heptopyranoside

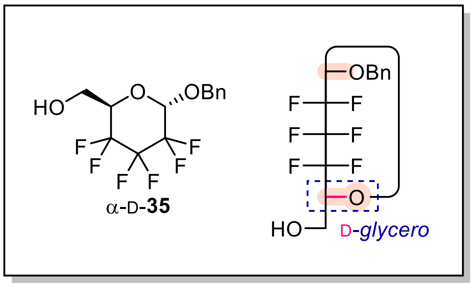

α-D-**35**: benzyl 2,3,4-trideoxy-2,2,3,3,4,4-hexafluoro-α-D-*glycero*-hexopyranoside

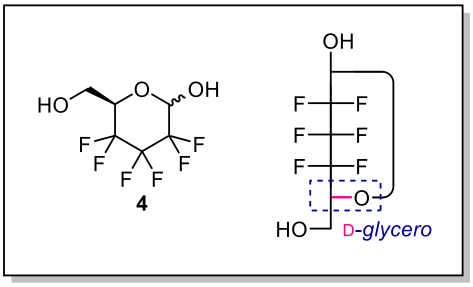

D-**4**: 2,3,4-trideoxy-2,2,3,3,4,4-hexafluoro-D-*glycero*-hexopyranose

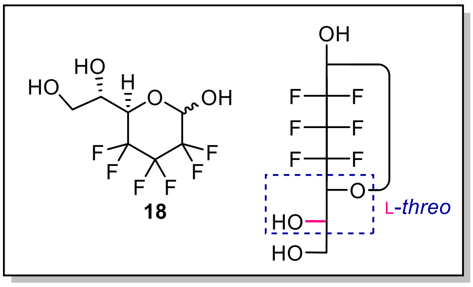

L-**18**: 2,3,4-trideoxy-2,2,3,3,4,4-hexafluoro-L-*threo*-heptopyranose

Fig. S1 – Names and Fischer projections of the sugar derivatives of this work

Derivative 32: The Fischer projection needs to be written with the anomeric carbon being the lowest in numbering. With the direction drawn in **Fig. S1** (CH_2 group from isopropylidene in position 1), the anomeric carbon is numbered as position 3. If top-down reversed (intracyclic CH_2 group in position 1), the anomeric carbon would be numbered as position 5. Having the correct Fischer direction then define the numbering for the other substituents and the side of the only chiral carbon, which is here

to the right, so **32** is a D-*glycero*-heptulopyranose. For this compound, the anomeric configuration is unknown (but the Fischer is drawn in the α-configuration).

2 Enantioselectivity determination for Sharpless asymmetric dihydroxylation of (*E*)-14

Analytical Chiral HPLC Data for diol **28**. Conditions: Chiralcel OD-H column, Hex/EtOH 9:1, 1 mL/min, 35 °C, UV(254 nm) detection.

a) Analysis of **28** after dihydroxylation (racemic diol):

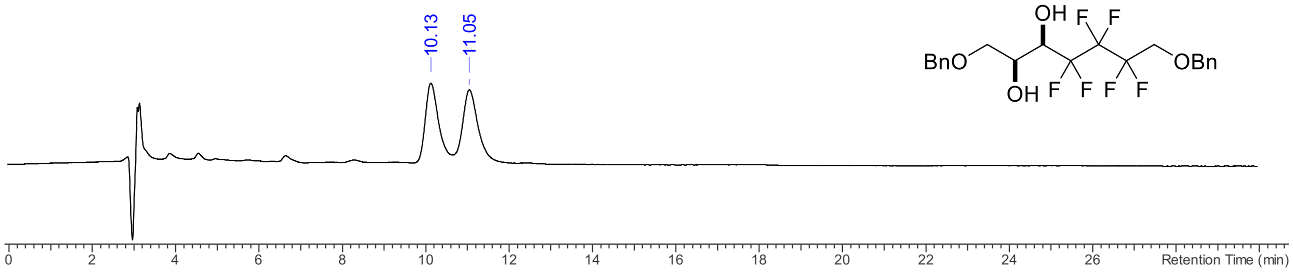

No	r.t. (min)	Peak area (a. u.)	Peak area (%)	Start r.t. (min)	End r.t. (min)	Width
1	10.13	345274	48.280	9.646	10.652	0.571
2	11.05	369871	51.720	10.652	12.079	0.648

b) Analysis of **28** after Sharpless asymmetric dihydroxylation:

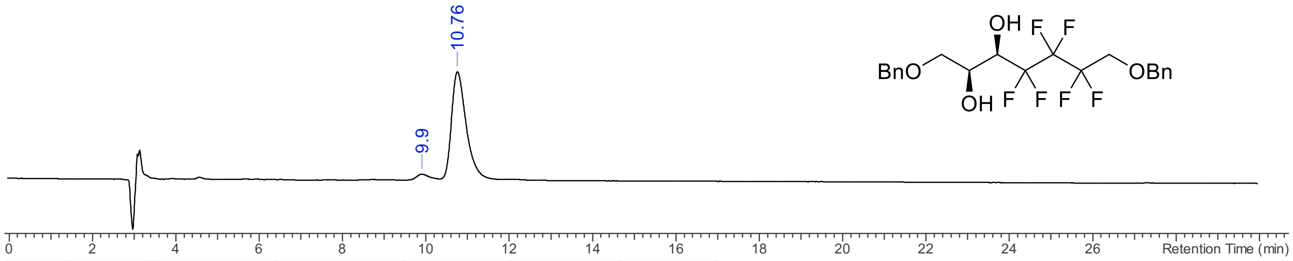

No	r.t. (min)	Peak area (a. u.)	Peak area (%)	Start r.t. (min)	End r.t. (min)	Width
1	9.90	47195	4.985	9.464	10.304	0.602
2	10.76	899618	95.015	10.304	12.071	0.626

c) Analysis of **28** after Sharpless asymmetric dihydroxylation followed by crystallization:

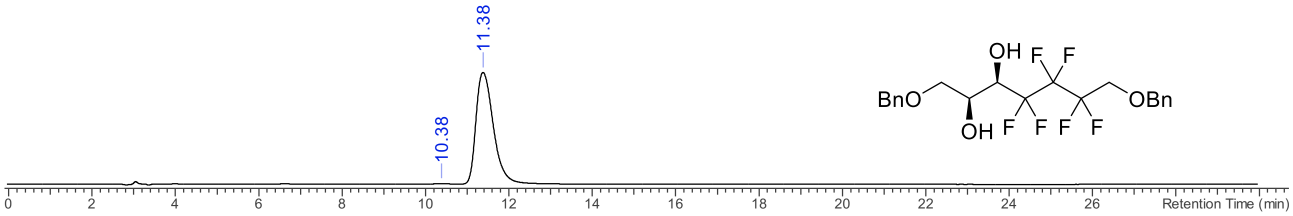

No	r.t. (min)	Peak area (a. u.)	Peak area (%)	Start r.t. (min)	End r.t. (min)	Width
1	10.38	76860	0.293	9.534	10.794	0.604
2	11.38	26177198	99.707	10.794	13.601	0.689

Fig. S2 – Chiral HPLC chromatograms of **28** for enantioselectivity determination

3 Regioselectivity determination for diol protection on tetraol 29

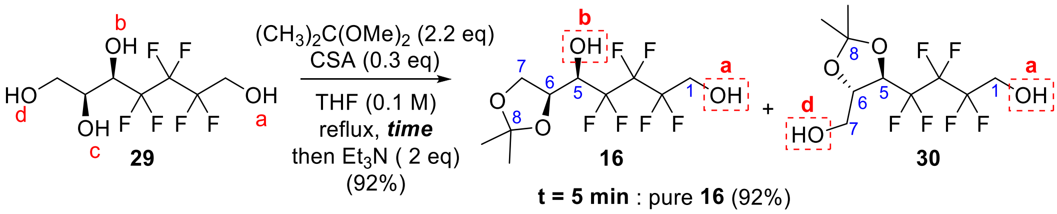

t = 5 min : pure **16** (92%)
t = 60 min : mixture of **16** (53%) and **30** (47%)

3.1 Kinetic control: 5 min reaction

After purification of the major compound formed after 5 min, the ^{1}H NMR spectrum of the latter was measured in DMSO-d^6 to visualize the hydroxyl groups. Only two OH signals can be seen, while the 2× 3H singlets at 1.35 and 1.29 ppm confirmed introduction of the acetonide protecting group (**Fig. S3**). The presence of an acetal carbon (**C-8**) at 109.3 ppm in ^{13}C supports this as well. Then, the strategy was to determine accurately which OH are free, to conclude that the two other ones are protected as the acetonide. To start, one OH signal is a doublet (δ 6.05 ppm) and the other is a triplet (δ 5.86 ppm), meaning they are respectively secondary and primary alcohols.

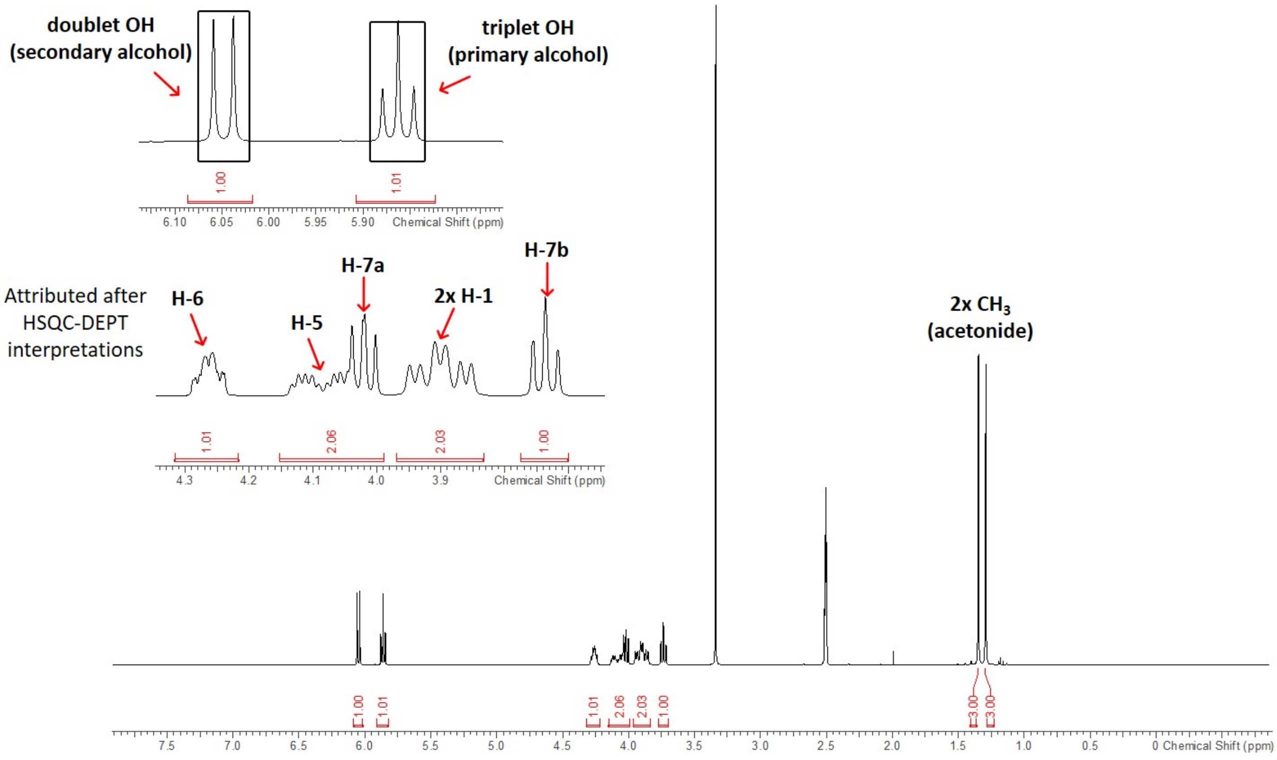

Fig. S3 - ^{1}H NMR spectrum of the pure protected diol after 5 min reaction

Next, the two carbon atoms adjacent to CF_2-groups (**C-1** and **C-5**) can easily be found because $J_{C\text{-}F}$ are observed. **C-1** is part of a CH_2-group and **C-5** is part of a CH-group so they are readily differentiated and identified by DEPT analysis. In the HSQC-DEPT spectrum (**Fig. S4**), it can be seen that **C-1** correlates to the dt (2H) at 3.99 ppm (which thus are the two **H-1**), and **C-5** to the doublet of multiplet (1H) at 4.08 ppm (thus being **H-5**). This leads to **C-6** (CH) and **C-7** (CH_2) being remaining, which are differentiated and identified rapidly in the HSQC-DEPT profile, allowing identification of **H-6** and **H-7** as well.

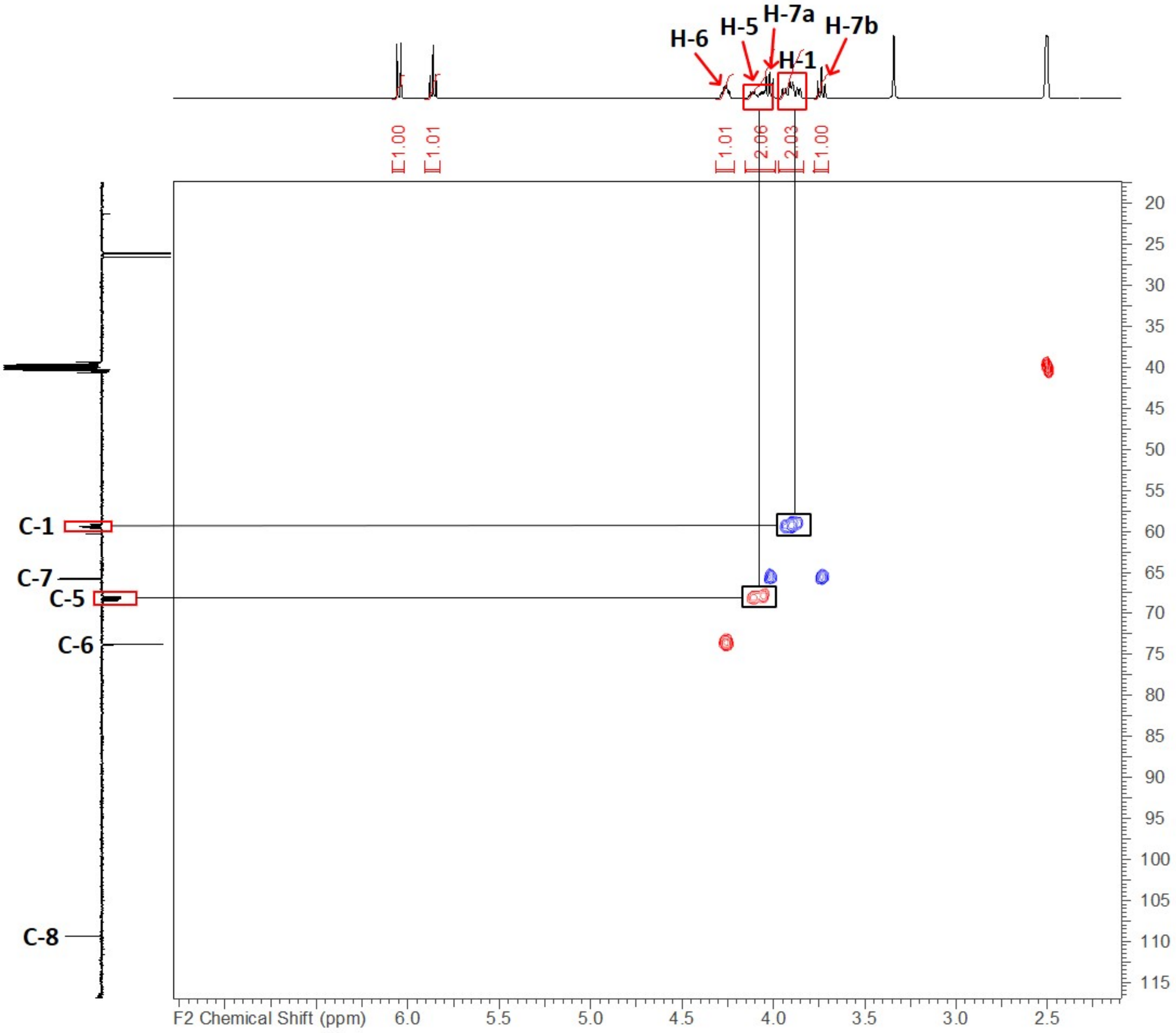

Fig. S4 – HSQC-DEPT spectrum of the pure protected diol after 5 min reaction

In the COSY spectrum (**Fig. S5**), **H-1** is correlated to the primary alcohol signal (triplet at 5.83 ppm), then being **OH-a**, and **H-5** is correlated to the secondary alcohol signal (doublet at 6.05 ppm) thus being **OH-b**. The two missing OH are **OH-c** and **OH-d**, which thereby are protected as the isopropylidene acetal. In HMBC, **C-8** correlates to the two **H-7**, which supports the terminal acetonide formation as well. These conclusions could be further confirmed by X-ray analysis.

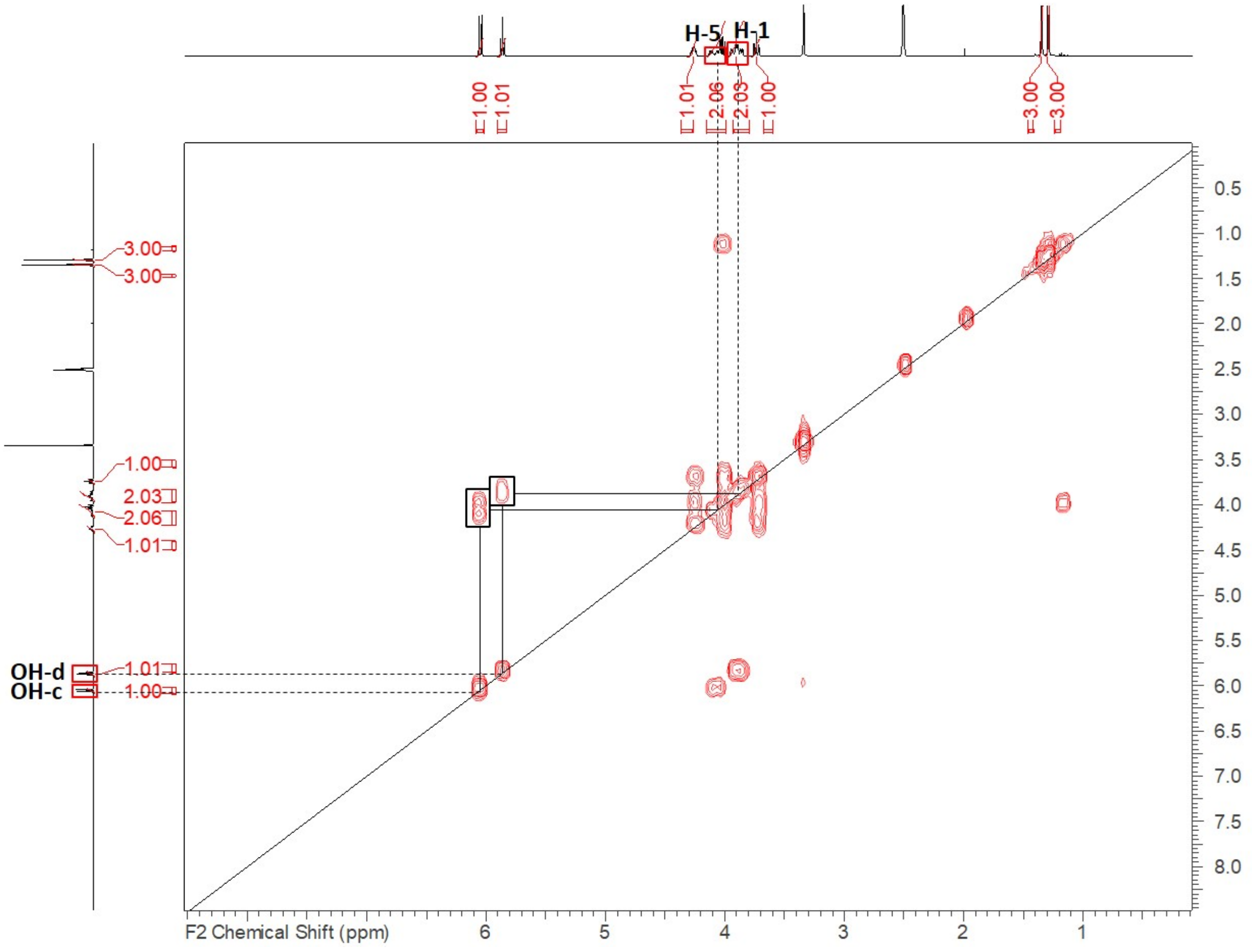

Fig. S5 - COSY spectrum of the pure protected diol after 5 min reaction

3.2 Longer experiment: 60 min reaction

For the 60 min reaction, two distinguishable but mostly overlapping spots were seen by TLC analysis (at the same R_f than **16**) and purified by column chromatography to isolate the mixture. The latter was analysed by NMR spectroscopy. In **Fig. S6**, the layering of ^{1}H NMR spectra (recorded in DMSO-*d*^{6}) of pure **16** (blue) and of the purified **mixture** (red) is presented. In the red spectrum, all signals from **16** can be observed (being 53% of the mixture), as well as additional signals (47% of the mixture) that are selected and marked with red arrows. In the chemical shift range of OH signals, two additional OH signals (δ 5.92 and 5.11 ppm) are seen and they are both triplets, meaning both primary alcohols. The only possibility is then that **OH-a** and **OH-d** are free, leading to the side product being the internal diol **30**. This was further supported by HSQC-DEPT and COSY analysis, based on the same strategy than for the 5 min reaction.

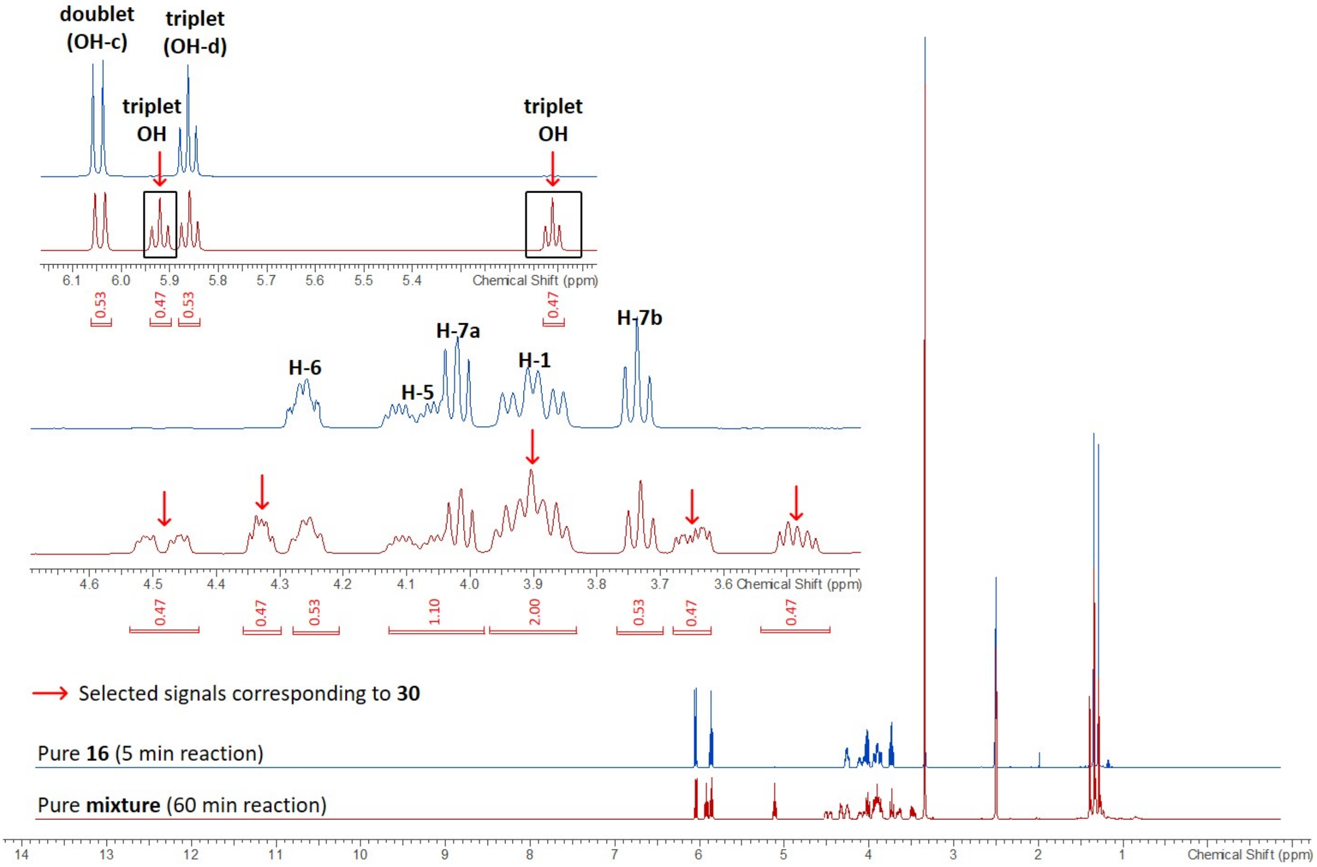

Fig. S6 - Layered ^{1}H spectra of pure **16** (blue) and of pure **mixture** (red)

First, a layered view of the DEPT spectra of pure **16** and pure **mixture** (**Fig. S7**) allowed to select the new signals for the second compound (red arrows). **C-5** was found easily at 73.4 ppm because being part of a CH-group and being near a CF_2-group ($J_{C\text{-}F}$). To find **C-1** (CH_2) a look back to the ^{1}H spectra (**Fig. S6**) led to hypothesize that the two **H-1** of the second compound are under the same multiplet as for **16** at 3.90 ppm: the multiplet integrates for 2H when everything else is split between 0.53H and 0.47H. In the HSQC-DEPT spectra (**Fig. S8**), this multiplet of 2H correlates to the only CH_2 with $J_{C\text{-}F}$ (obviously at the same chemical shift as for pure **16**), supporting that the signals for **C-1** and **H-1** are overlapping for both **16** and the second compound. In the same way as before, knowing where **C-1** and **C-5** are, **C-6** (CH) and **C-7** (CH_2) could be easily identified by DEPT analysis, as well as their corresponding **H-5**, **H-6** and **H-7** using HSQC analysis. Finally, on the COSY spectra (**Fig. S9**), the OH triplet at 5.98 ppm correlates with the **H-1**-overlapped multiplet, so it is **OH-a**, and the OH triplet at 5.17 ppm correlates with **H-7**, so it is **OH-d**. Taken as whole, these correlations lead to the unambiguous conclusion that the second compound is the internal diol **30**.

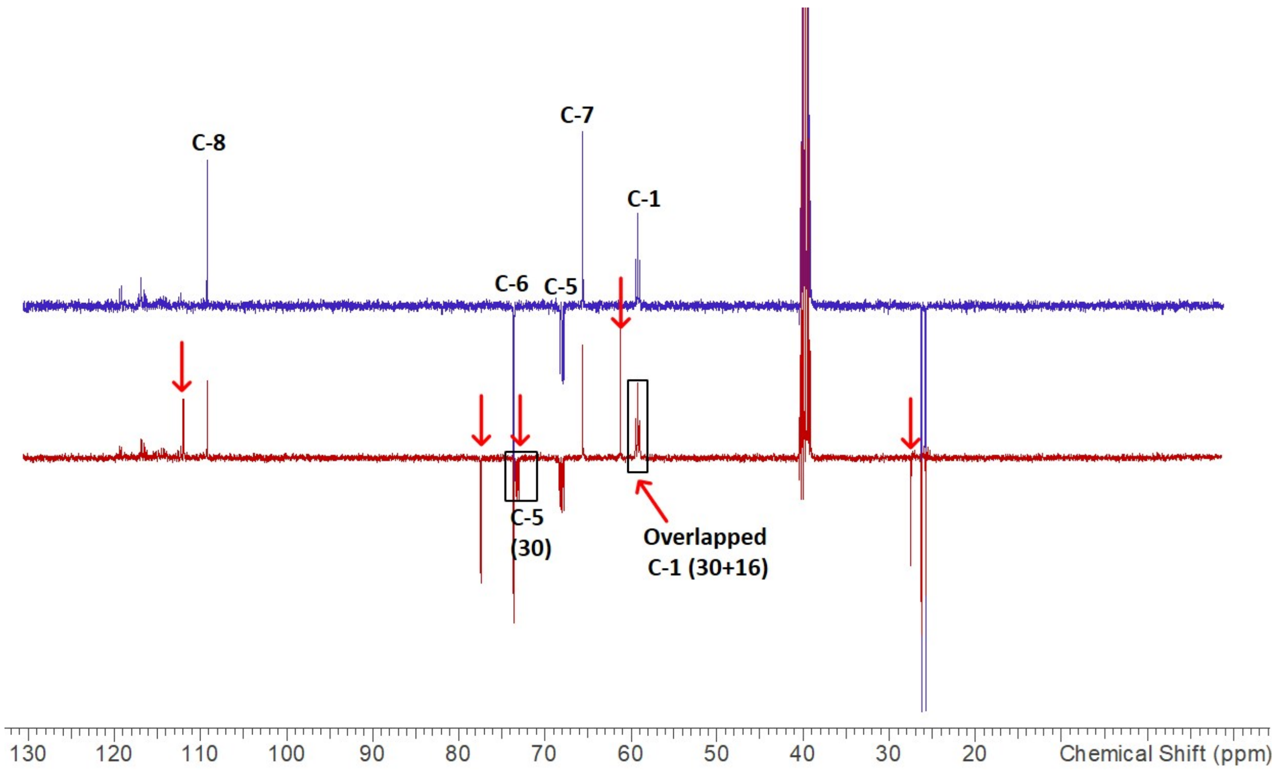

Fig. S7 - Layered DEPT spectra of pure **16** (blue) and of pure **mixture** (red)

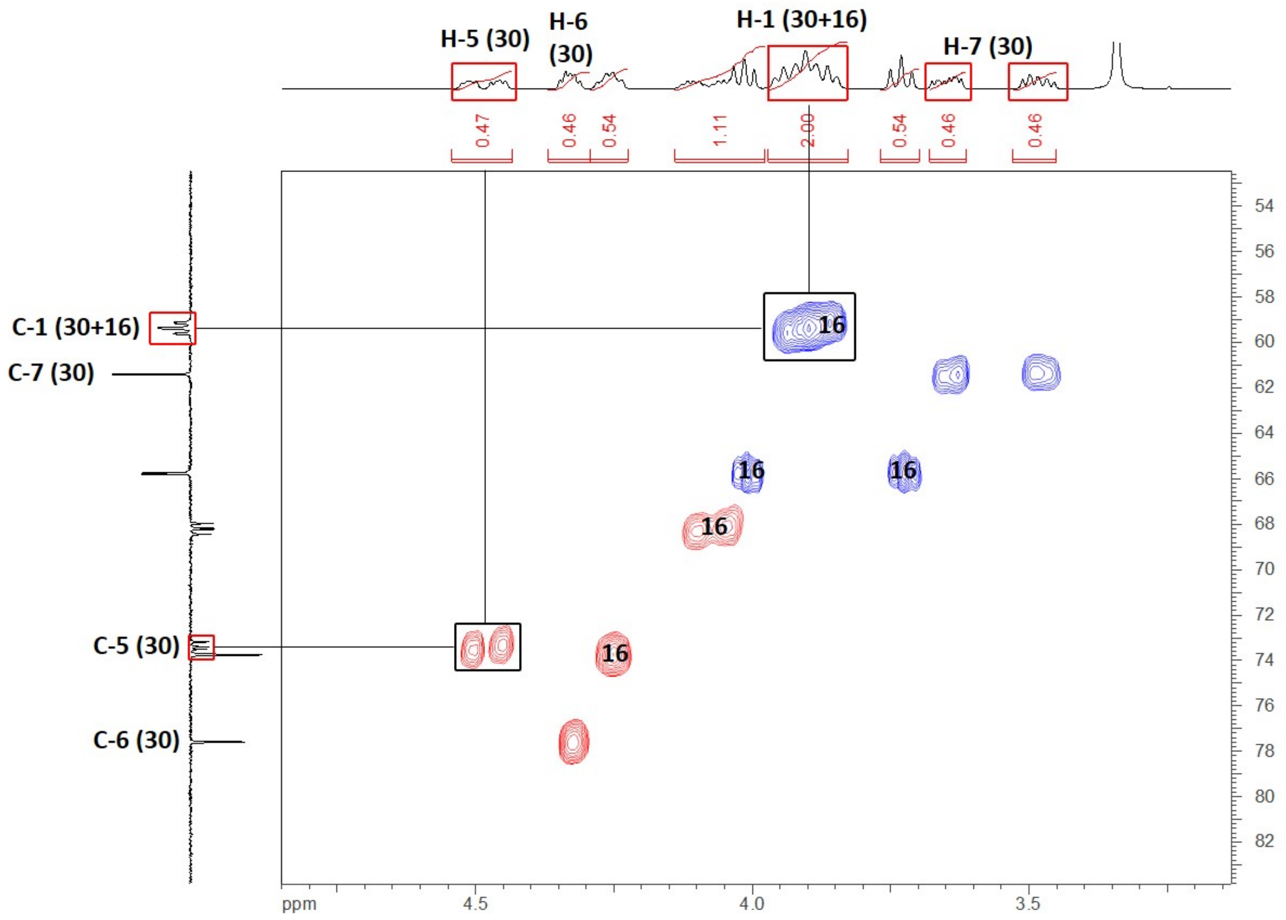

Fig. S8 - HSQC-DEPT spectrum of pure **mixture**

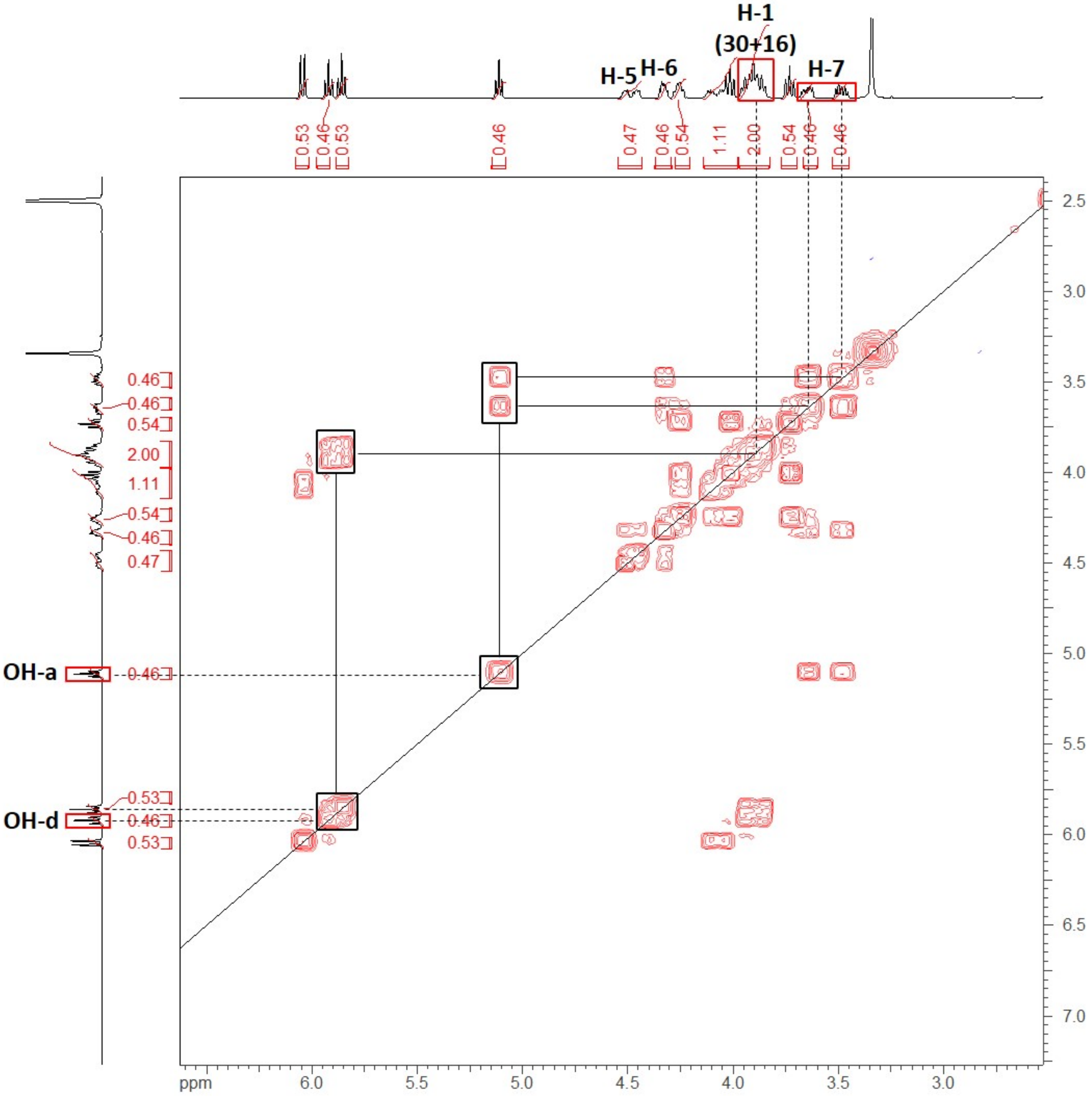

Fig. S9 - COSY spectrum of pure **mixture**

4 Determination of the ^{13}C chemical shifts of the CF_2-groups using HMBC analysis

For all the intermediates of the synthesis, the signals of the quaternary carbon atoms of the CF_2-groups are poorly (or even not) visible in the ^{13}C spectra. Thus, a range of chemical shifts was determined using HMBC analysis. The following **Fig. S10** shows the HMBC spectrum of pure intermediate **28**, as an example. In black squares can be seen correlation dots of **OH-5**, **H-5** and the two **H-1** (and to a lesser extent **H-6**) to three invisible carbon atoms in a range of 110 to 120 ppm, which thus are the three CF_2 carbon atoms. This strategy was used for all the intermediates of this work, and the three CF_2 carbon atoms were always seen in the 105-120 ppm range.

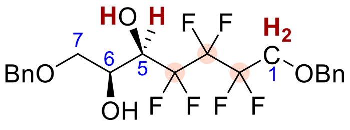

28

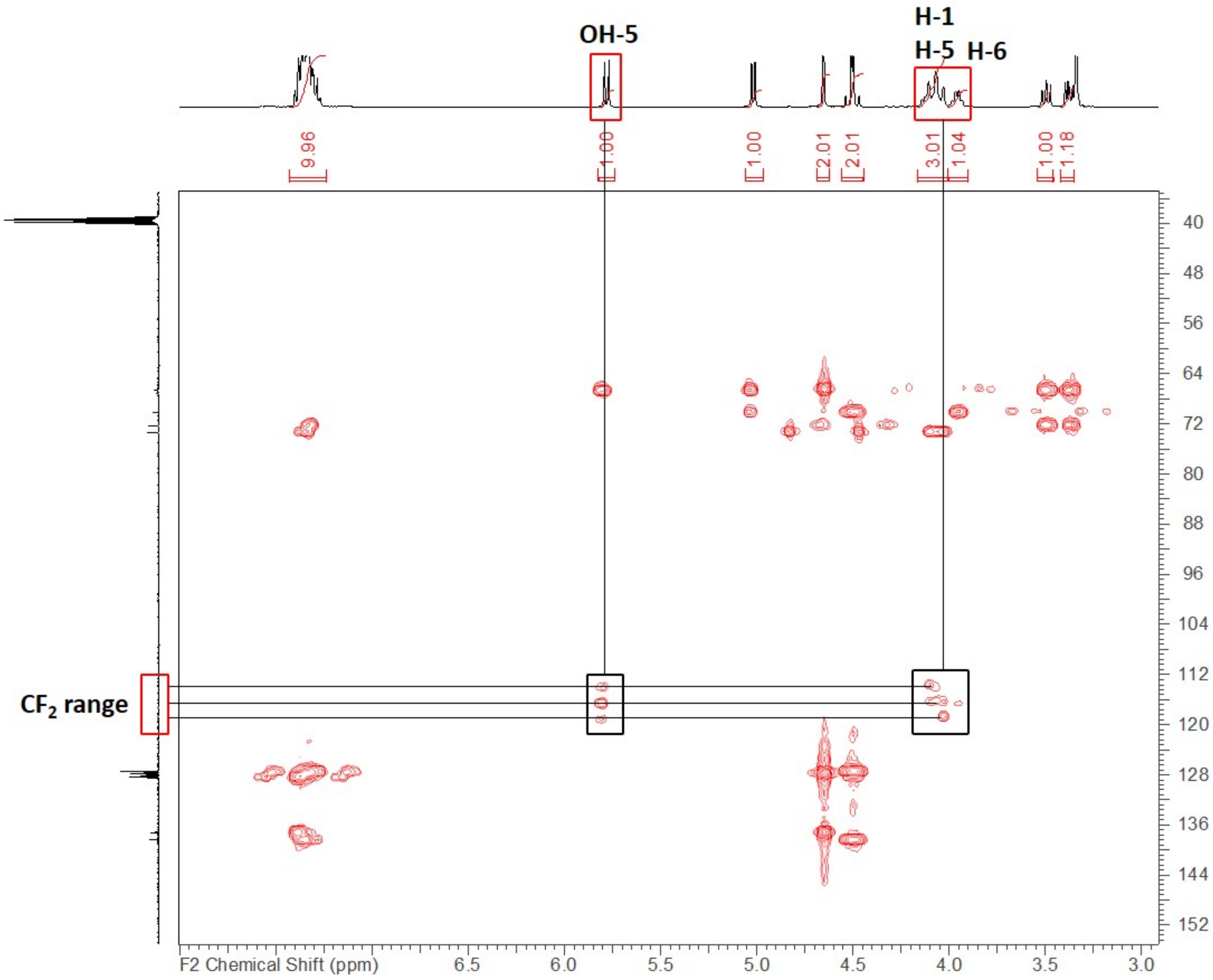

Fig. S10 – HMBC spectrum of pure **28**

5 Determination of the size of the heterocyclic ring for hexafluorinated hexose D-4 and heptose L-18

a) Hexose D-4

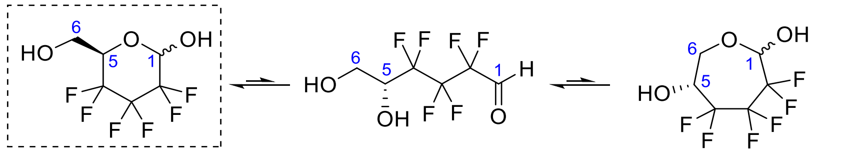

D-**4**, 6-membered ring D-**4**, open chain D-**4**, 7-membered ring

First, for both anomers, **C-1** and **C-5** can easily be identified by ^{13}C NMR analysis because they are doublets of doublets (coupling with fluorine atoms). The anomeric **C-1** of both anomers are in the 100 ppm area (92.5 and 91.9 ppm). As explained in the manuscript and in **Fig. 1**, $J_{C1\text{-}F}$ coupling constants allowed to identify that the α sugar is the major anomer (OH-1 in axial position). Thereby, **C-5α** and **C-5β** are unambiguously identified at 74.4 and 69.3 ppm respectively. Then, using HSQC analysis, **H-5α** and **H-5β** are located on the ^{1}H spectra. Finally, the proof that D-**4** is in the pyranose ring form was deduced using HMBC analysis (**Fig. S11**) based on the following observations:

- Anomeric **H-1** (α and β) correlates with **C-5** (α and β) and not with **C-6**
- Anomeric **C-5** (α and β) correlates with **H-5** (α and β) and not with **H-6**

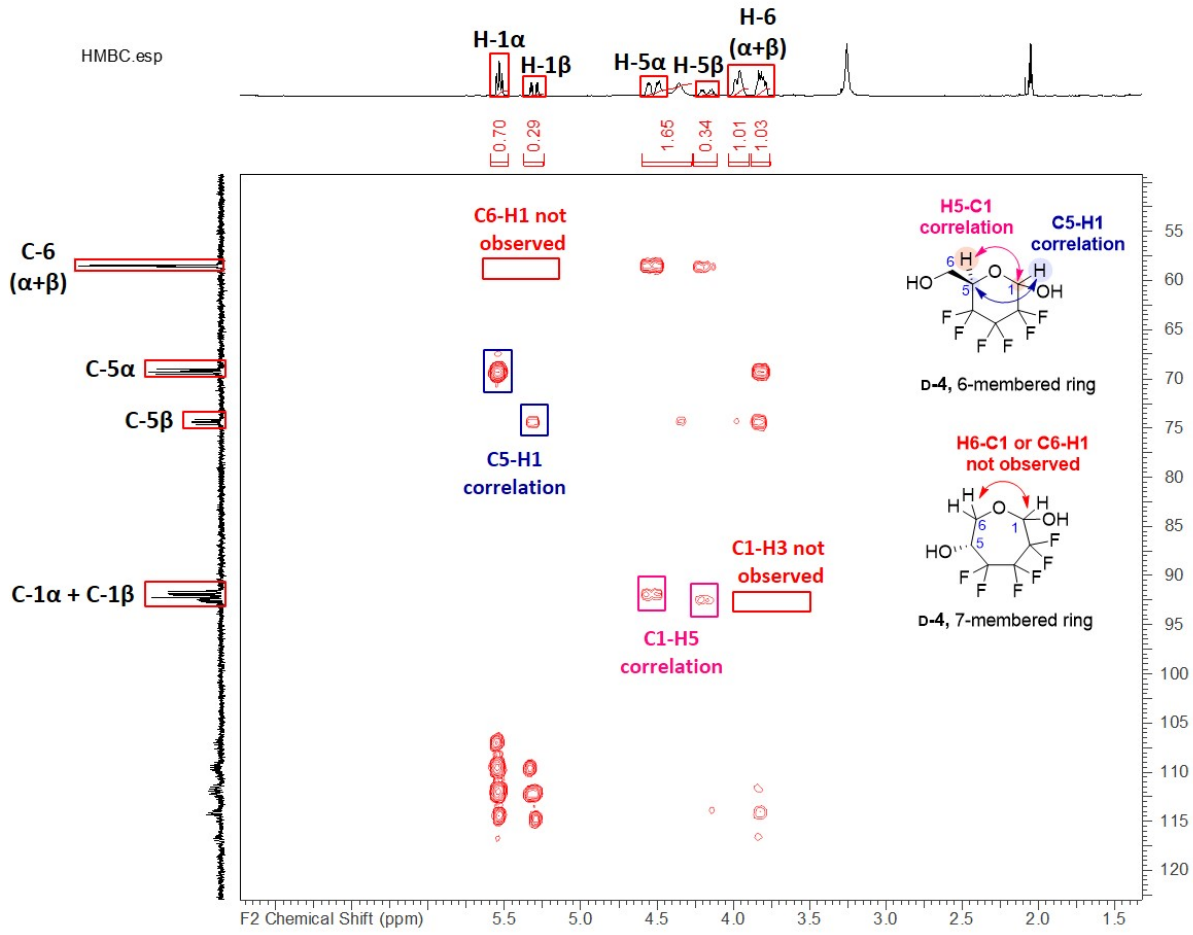

Fig. S11 – HMBC spectrum of pure D-**4**, recorded after 10 hours of equilibration in acetone-d^6

b) **Heptose L-18**

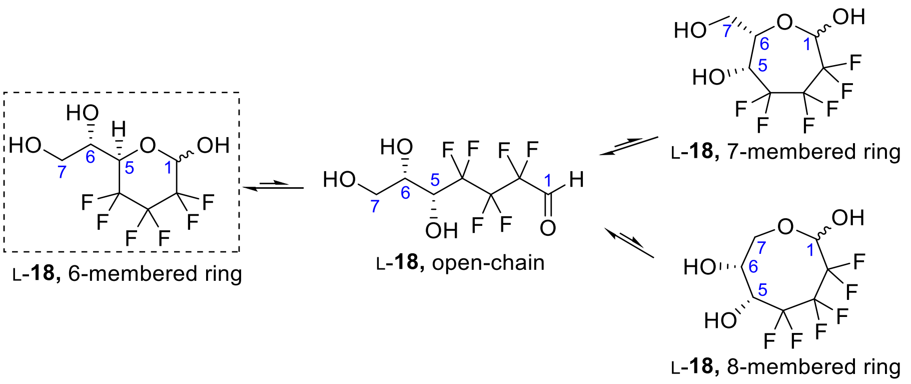

The same strategy as for D-**4** was used to study L-**18**. Using HSQC analysis, all hydrogen and carbon atoms were identified. Then, the proof that L-**18** is in the pyranose ring form was deduced using HMBC analysis (**Fig. S12**) based on the following observations:

- Anomeric **H-1** (α) correlates with **C-5** (α) and not with **C-6** or **C-7**
- Anomeric **C-1** (α and β) correlates with **H-5** (α and β) and not with **H-6** or **H-7**

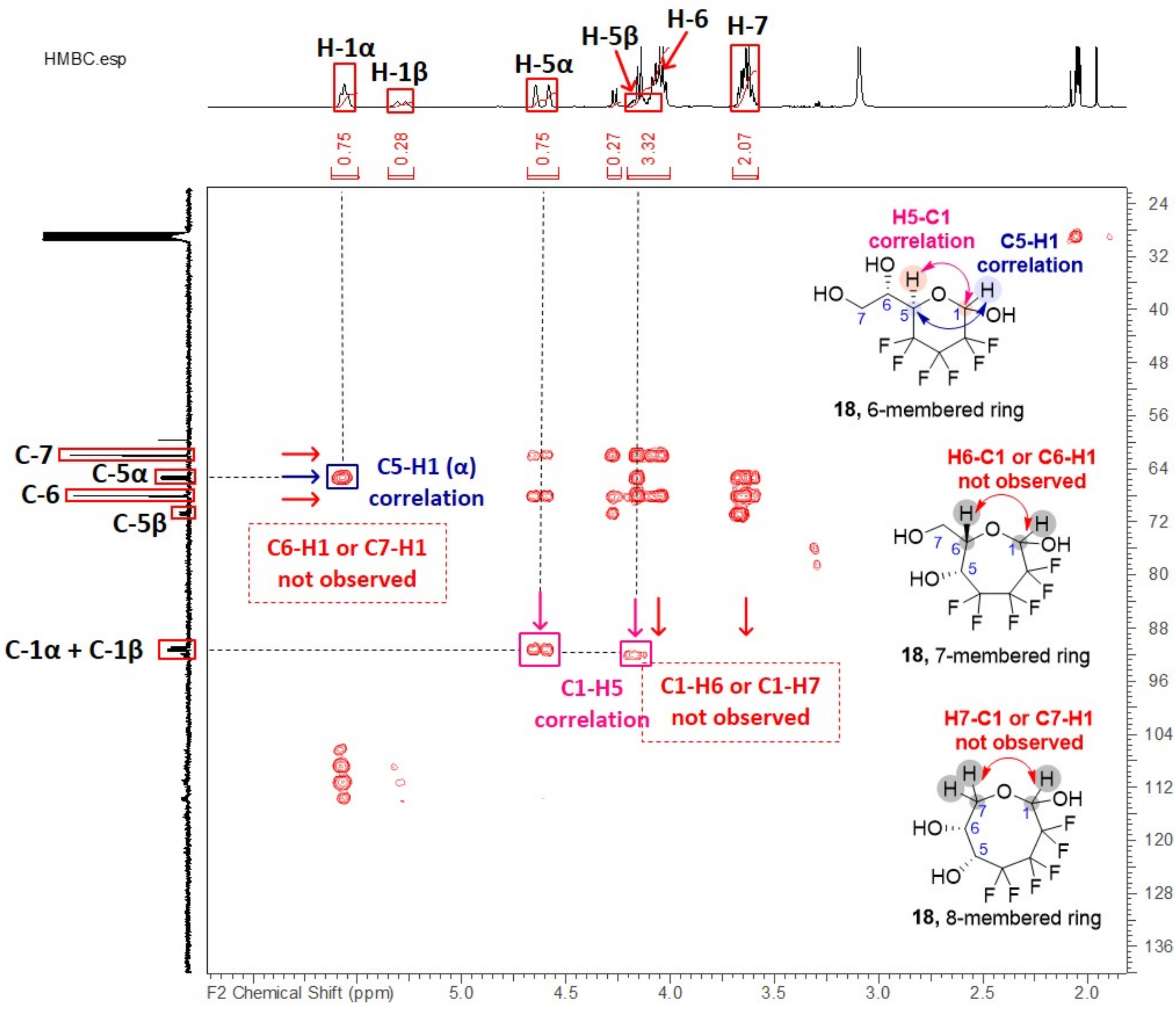

Fig. S12 – HMBC spectrum of pure L-**18**, recorded after 10 hours of equilibration in acetone-d^6

6 NMR spectra for novel compounds

6.1 NMR spectra of 3,3,4,4,5,5-hexafluorooxane-2-ol (20)

a) ^{1}H NMR (DMSO-d^{6}, 400 MHz)

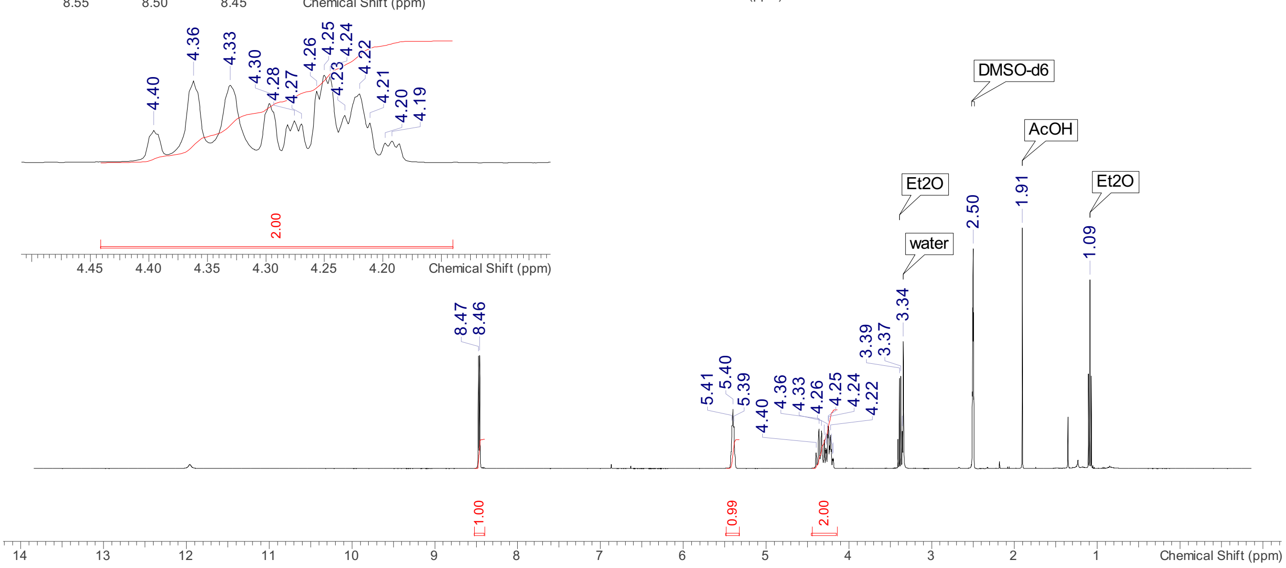

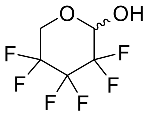

20

b) ^{1}H{^{19}F} NMR (DMSO-d^{6}, 500 MHz)

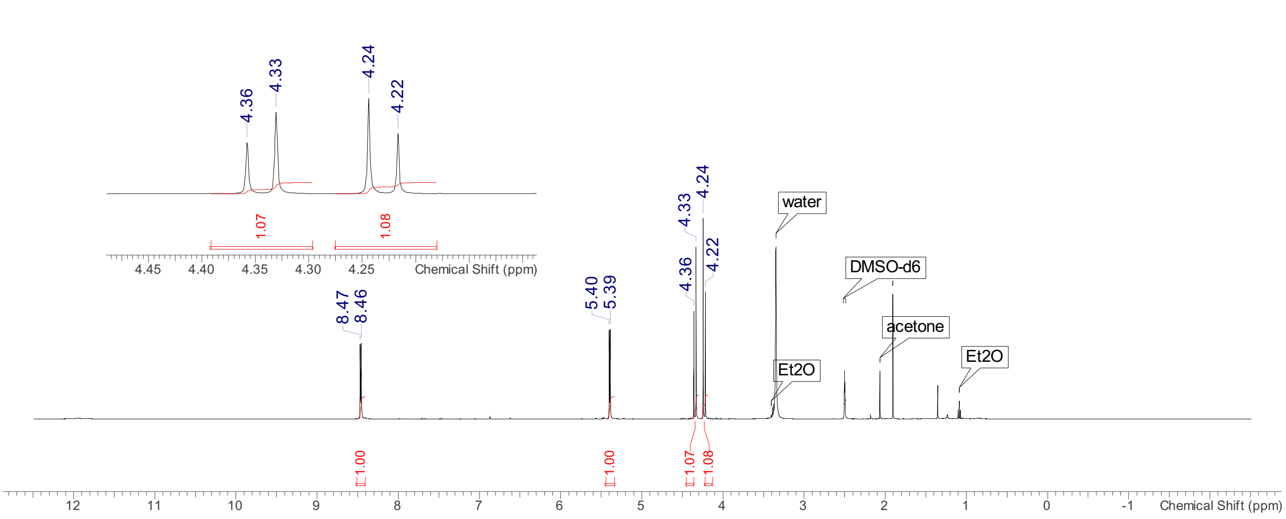

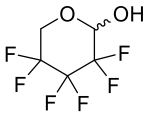

20

c) ^{19}F NMR (DMSO-d^6, 376 MHz)

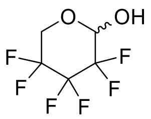

20

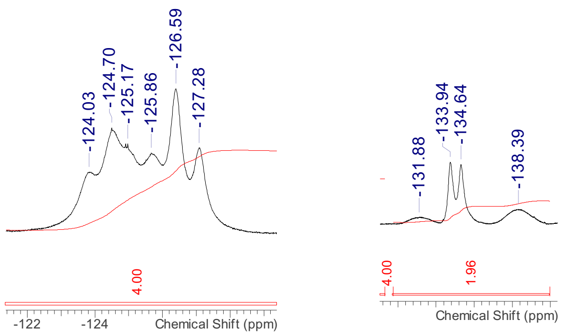
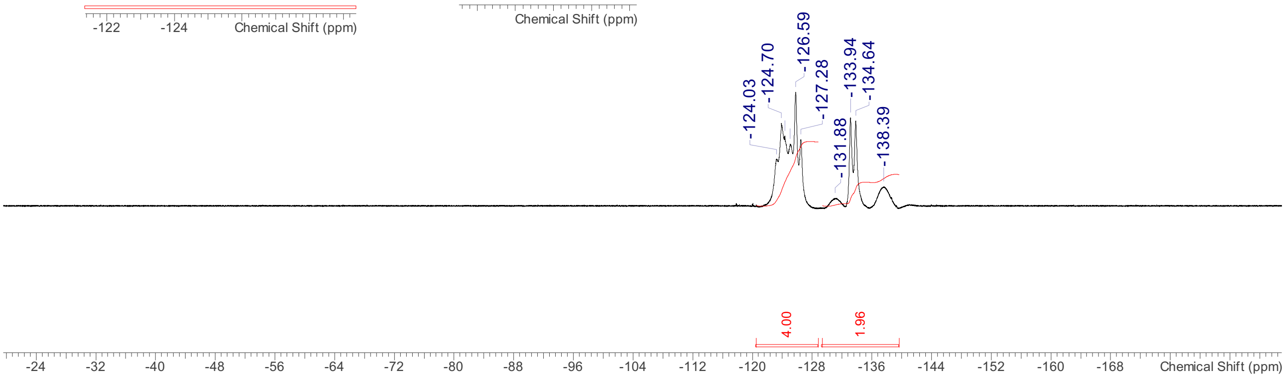

d) ^{19}F{^{1}H} NMR (DMSO-d^6, 471 MHz)

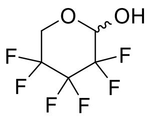

20

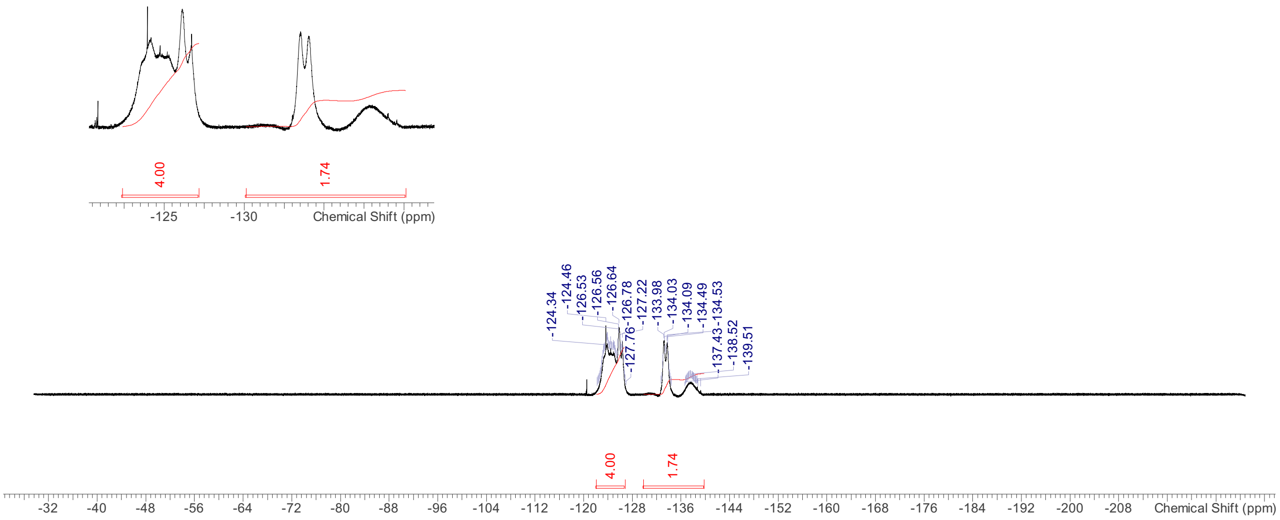

e) $^{13}C\{^{1}H\}$ NMR (DMSO-d^6, 101 MHz)

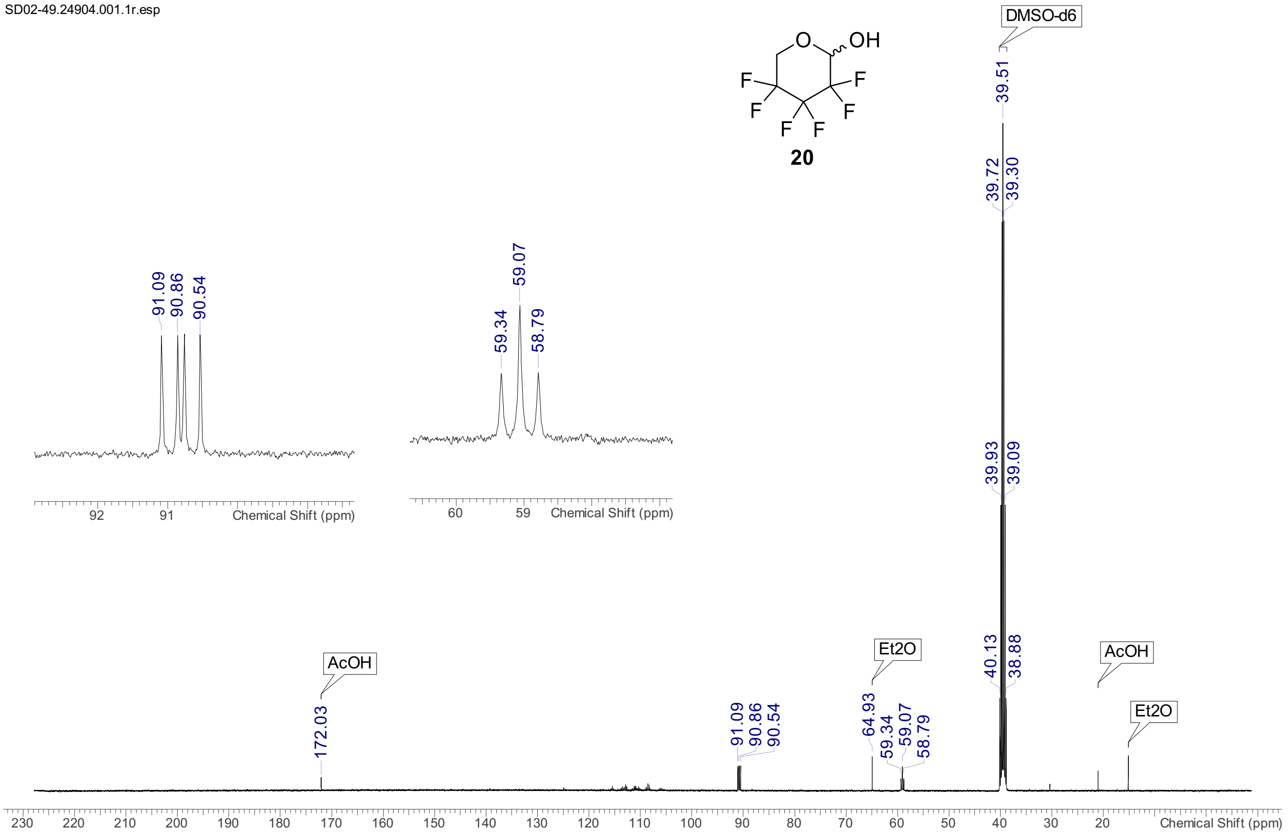

6.2 NMR spectra of (*rac*)-methyl 2-(3,3,4,4,5,5-hexafluorooxan-2-yl)acetate (22)

a) ^{1}H NMR (DMSO-d^6, 400 MHz)

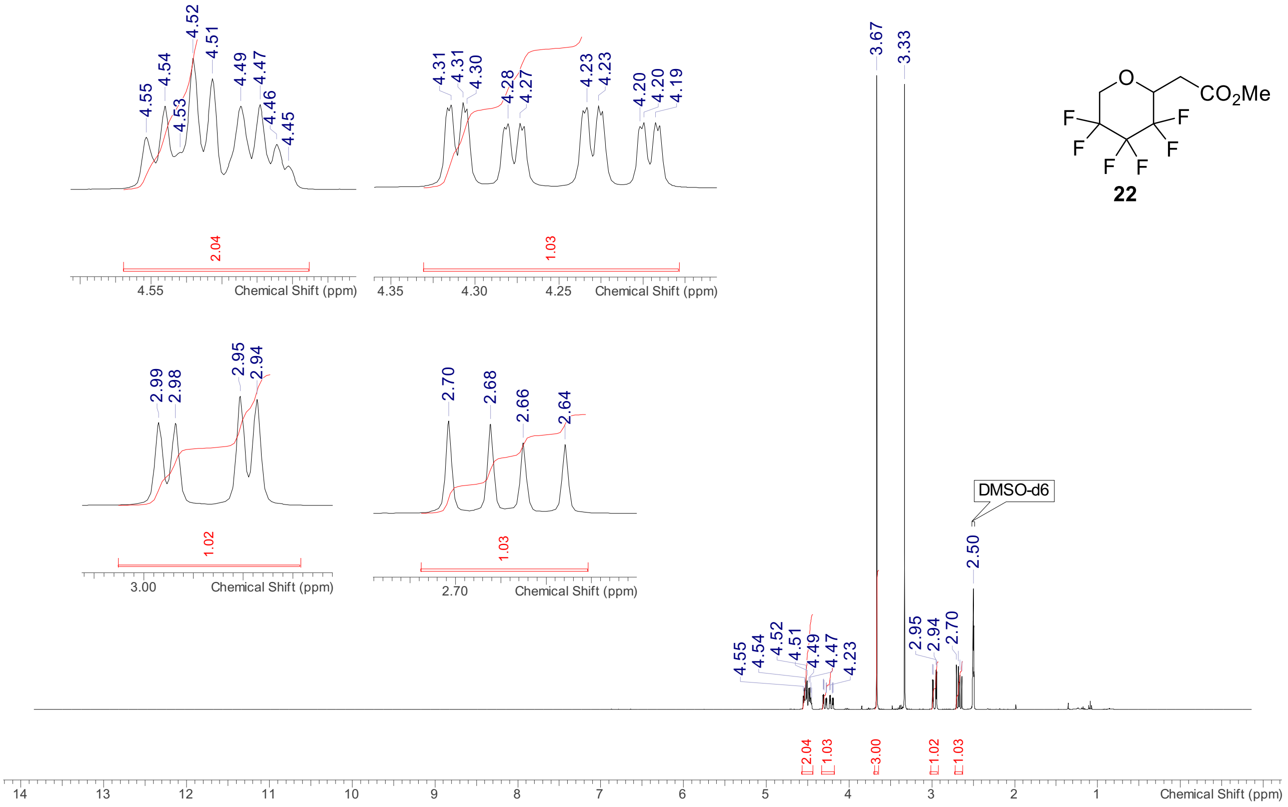

b) ^{1}H {^{19}F} NMR (DMSO-d^6, 500 MHz)

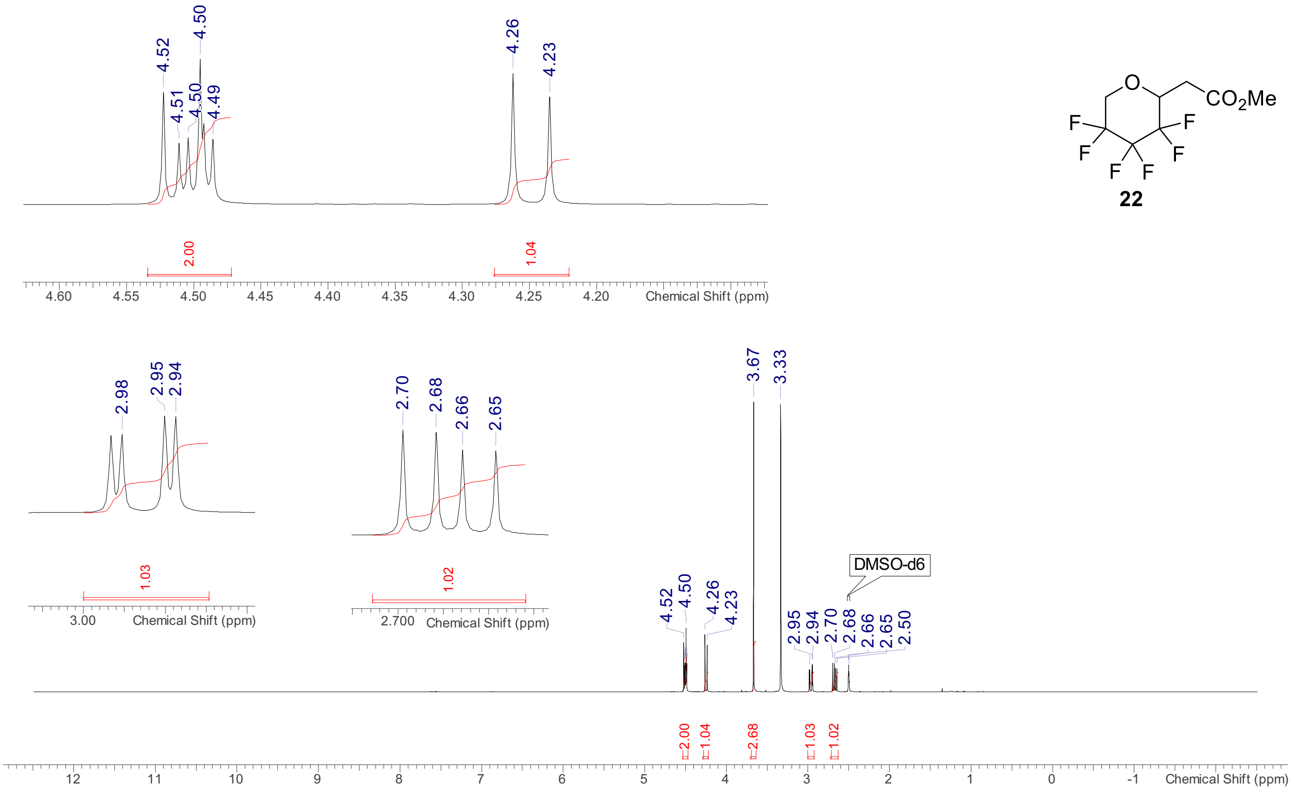

c) ^{19}F NMR (DMSO-*d*[6], 376 MHz)

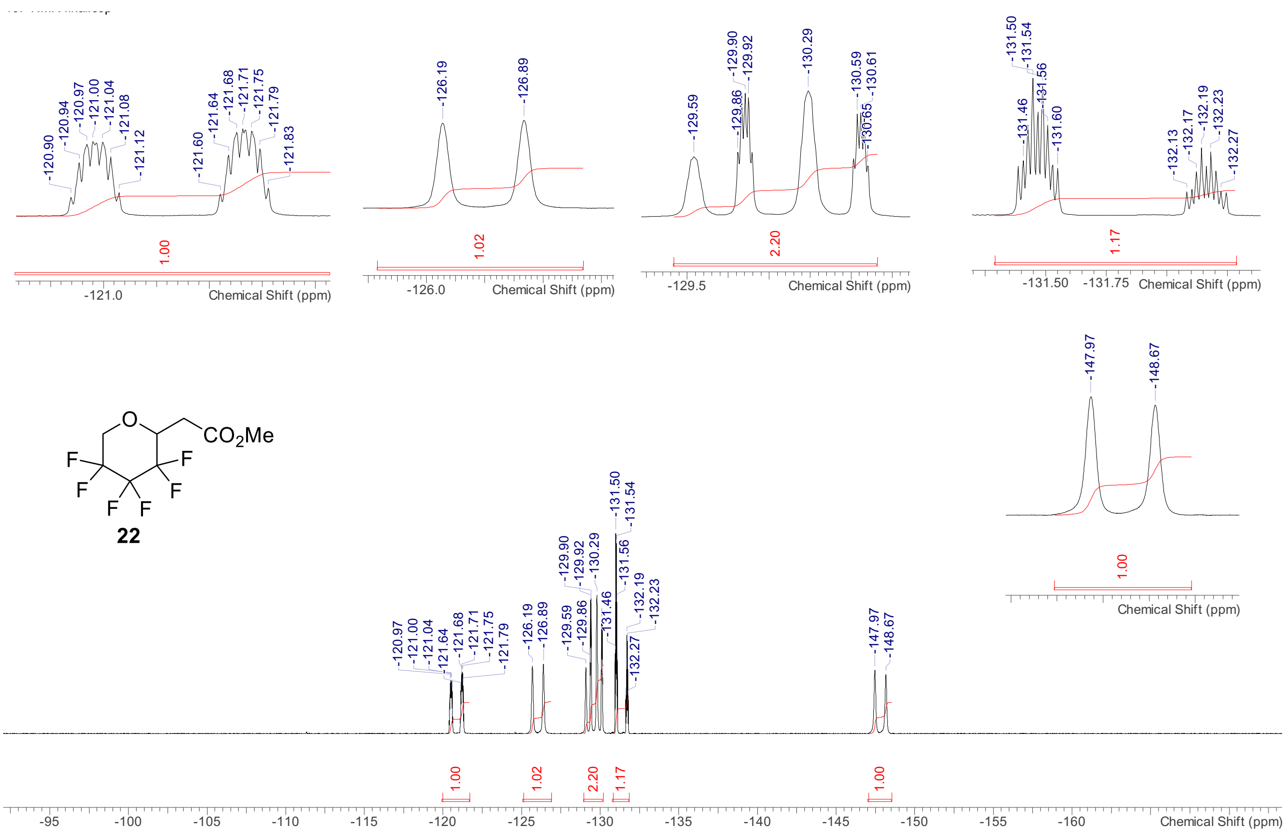

d) ^{19}F{^{1}H} NMR (DMSO-*d*[6], 471 MHz)

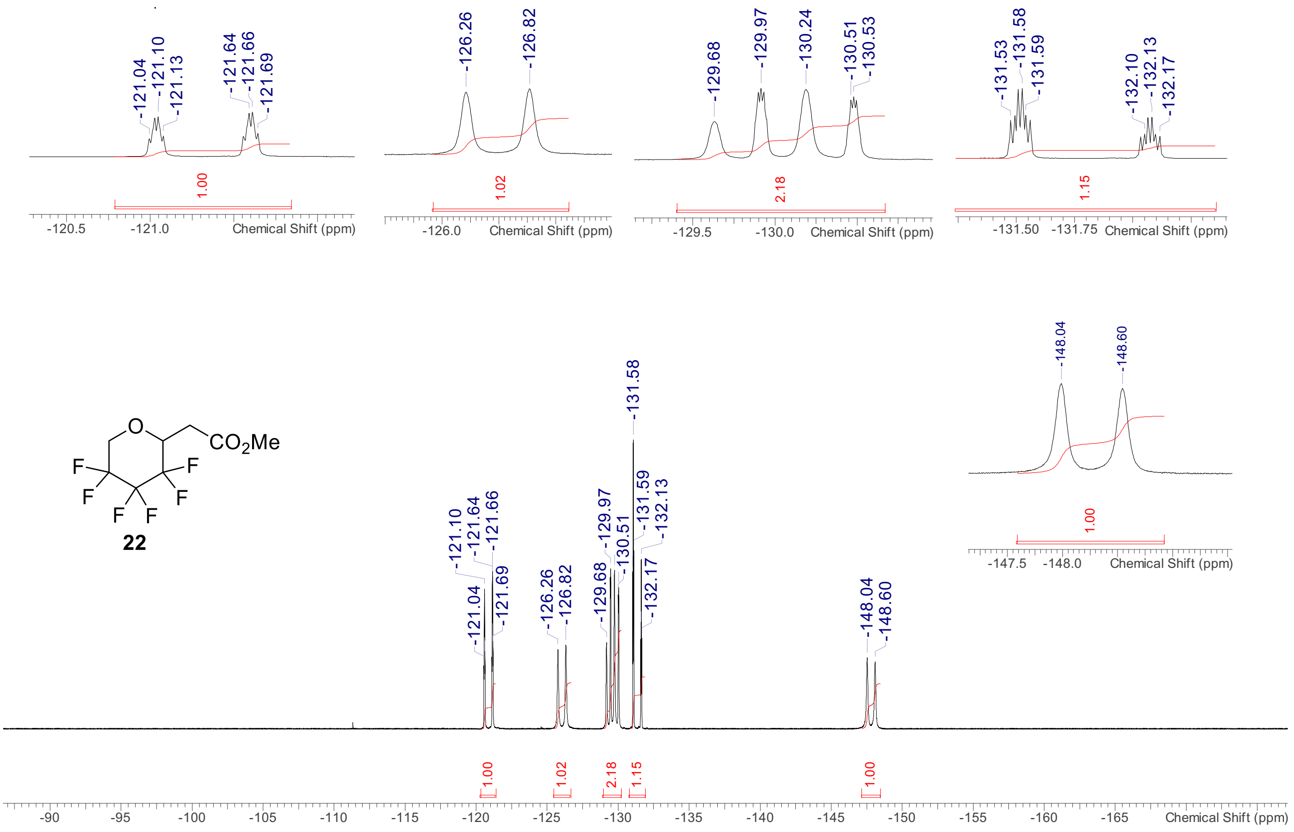

e) $^{13}C\{^{1}H\}$ NMR (DMSO-d^6, 101 MHz)

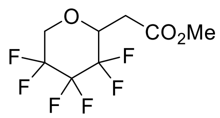

22

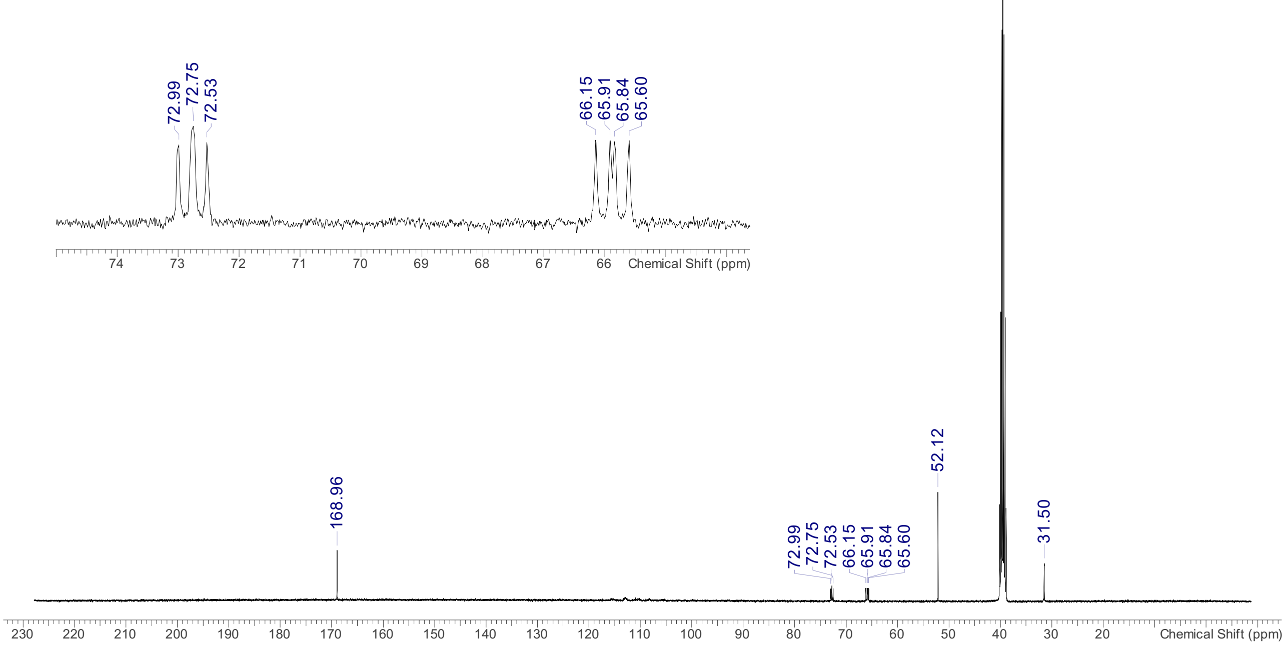

6.3 NMR spectra of 5-(benzyloxy)-2,2,3,3,4,4-hexafluoropentan-1-ol (23)

a) ^{1}H NMR (DMSO-d^{6}, 400 MHz)

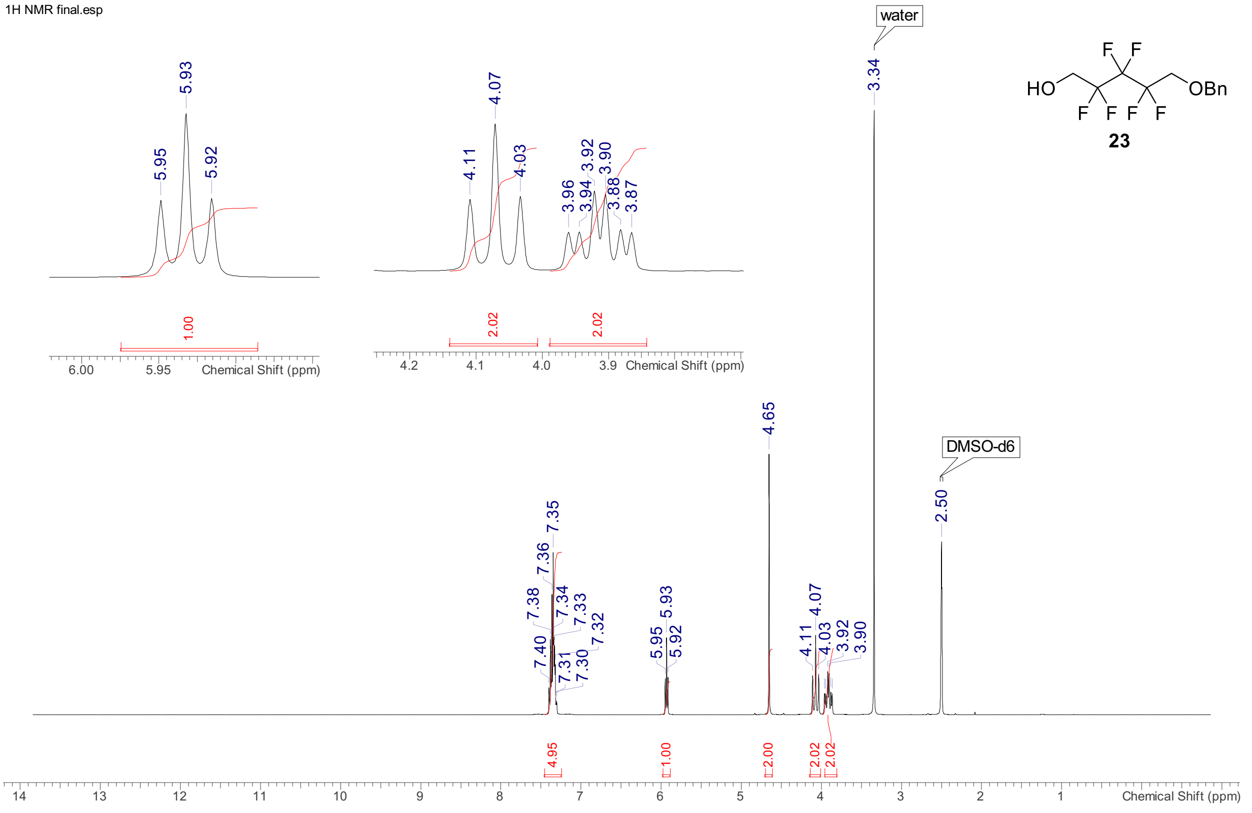

b) ^{1}H {^{19}F} NMR (DMSO-d^{6}, 500 MHz)

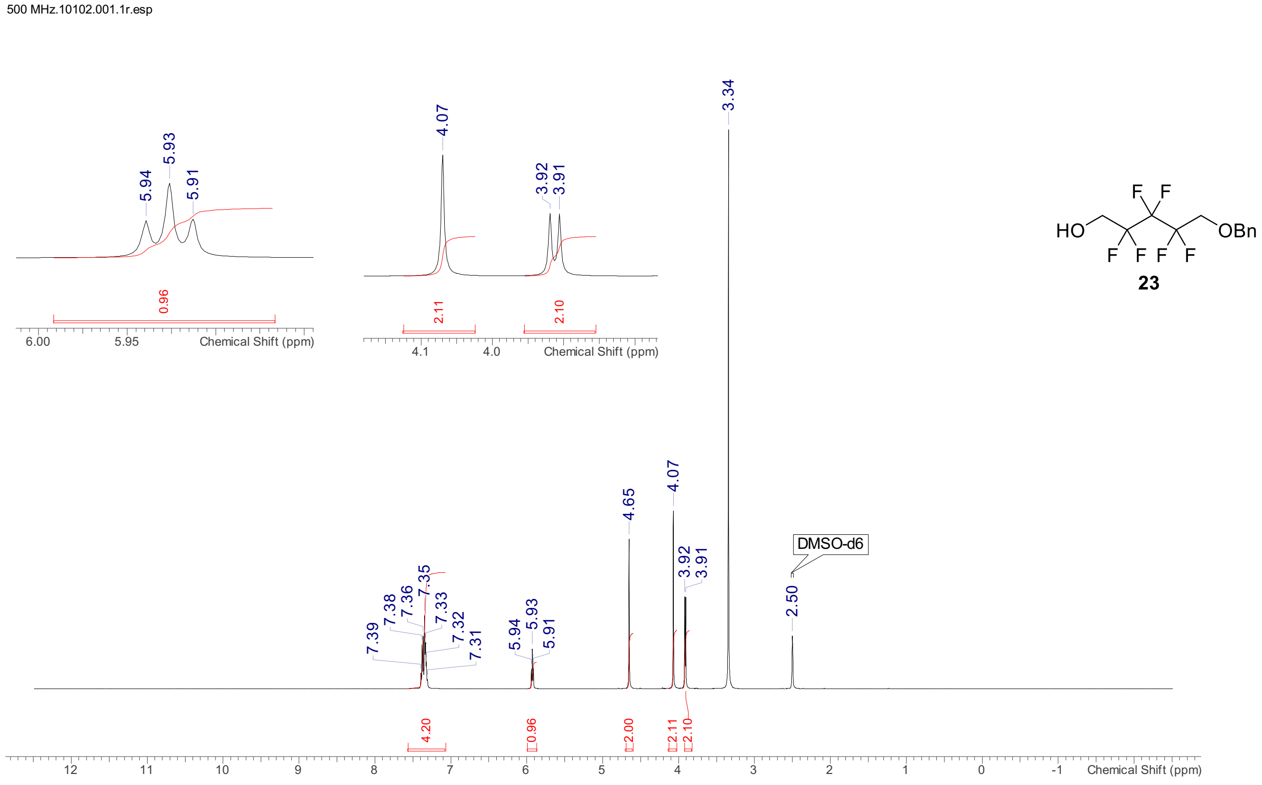

c) ^{19}F NMR (DMSO-d^6, 376 MHz)

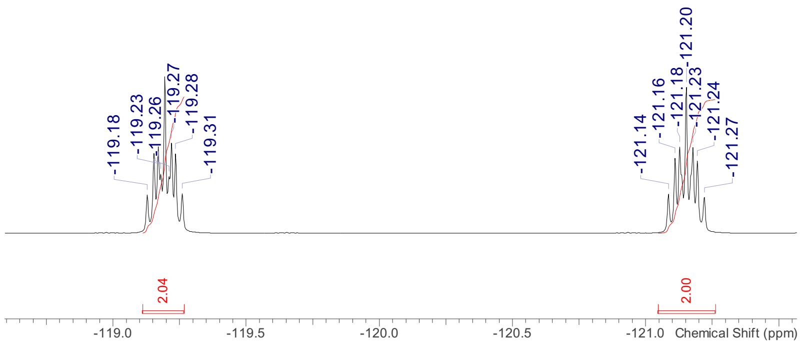

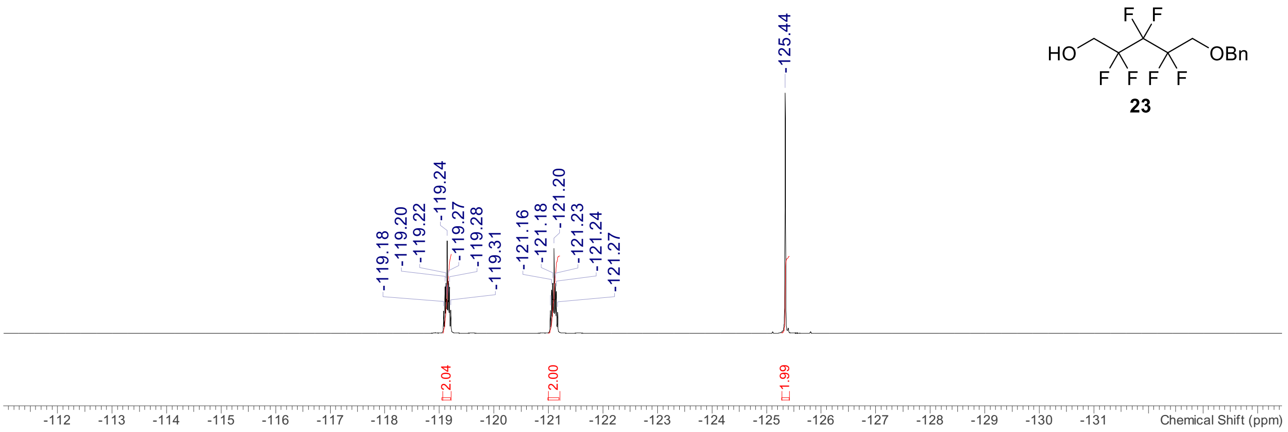

23

d) ^{19}F{^{1}H} NMR (DMSO-d^6, 471 MHz)

500 MHz.10104.001.1r.esp

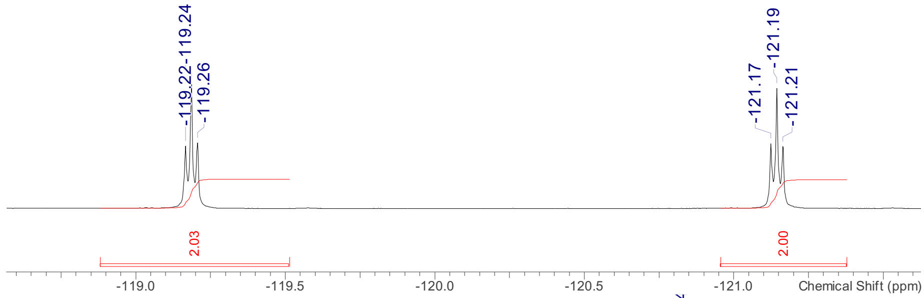

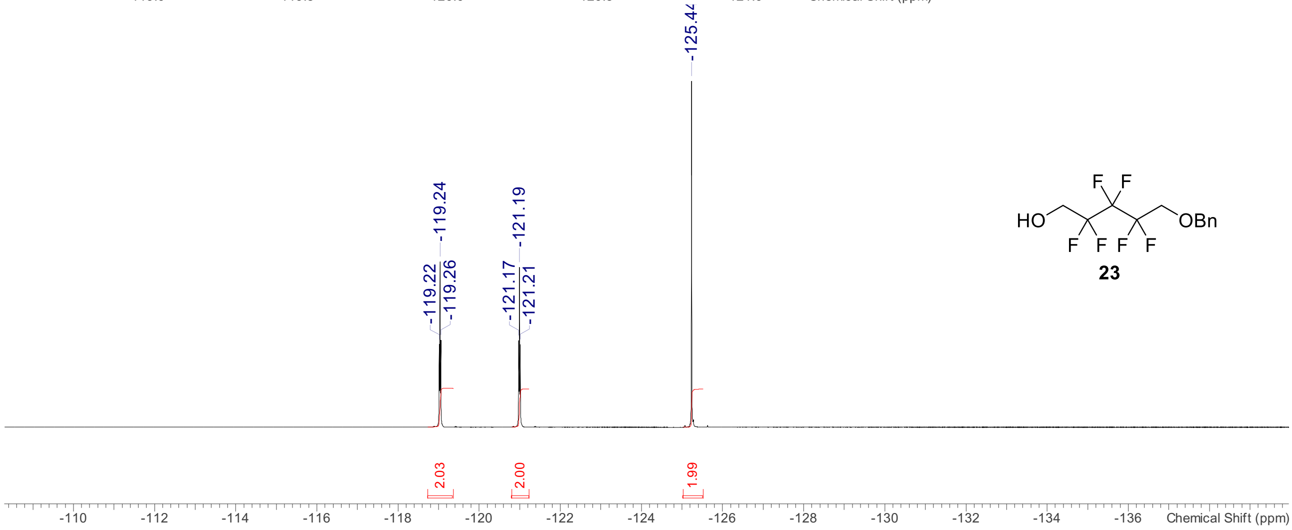

23

e) $^{13}C\{^{1}H\}$ NMR (DMSO-d^6, 101 MHz)

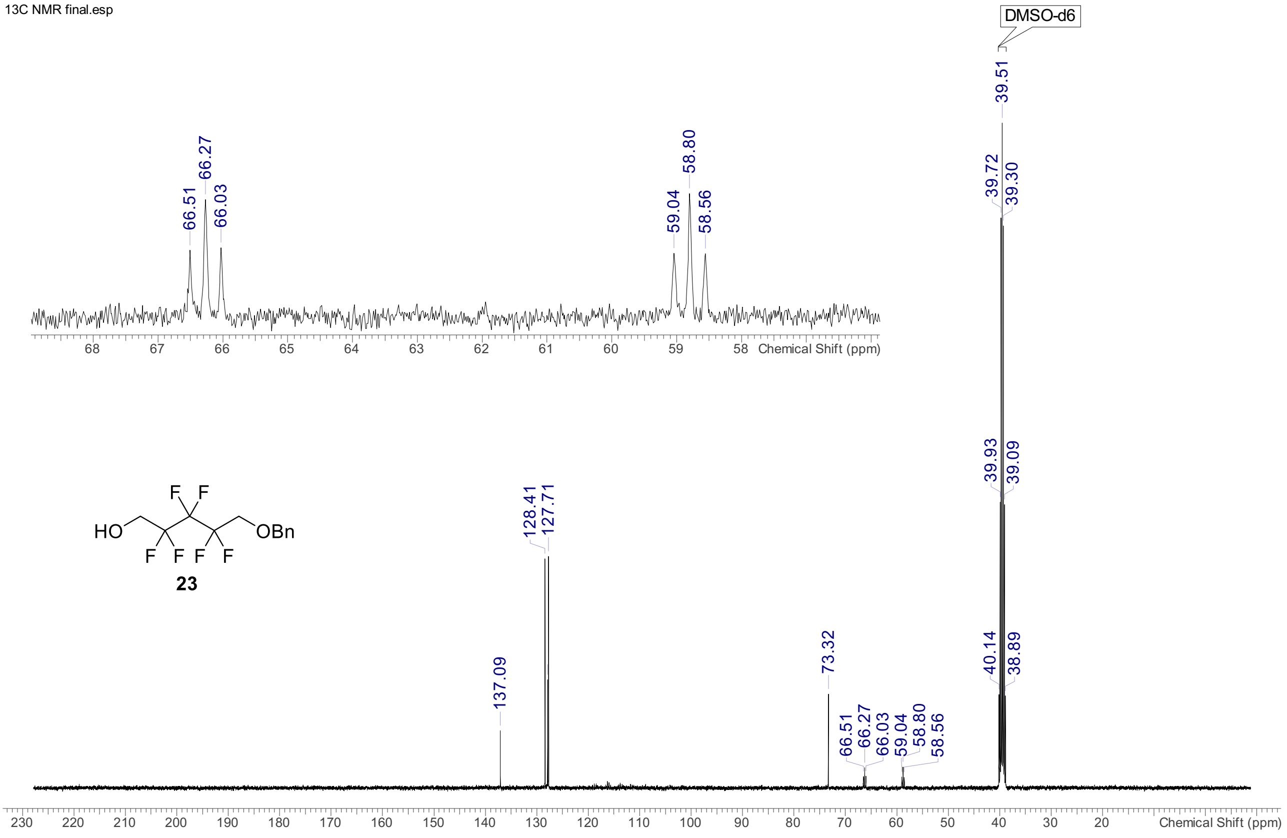

6.4 NMR spectra of 1,5-bis(benzyloxy)-2,2,3,3,4,4-hexafluoropentane (S1)

a) ^{1}H NMR (DMSO-d^{6}, 400 MHz)

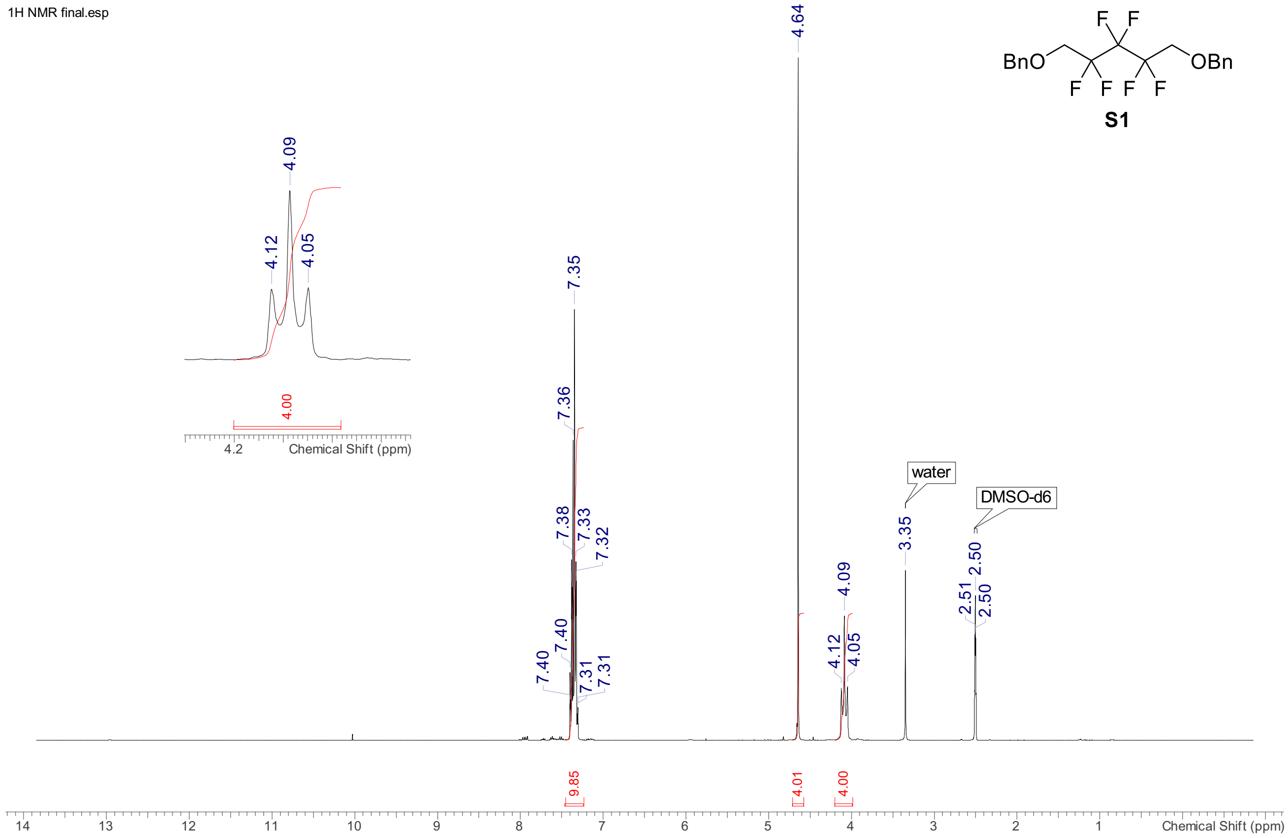

b) $^{1}H\{^{19}F\}$ NMR (DMSO-d^{6}, 500 MHz)

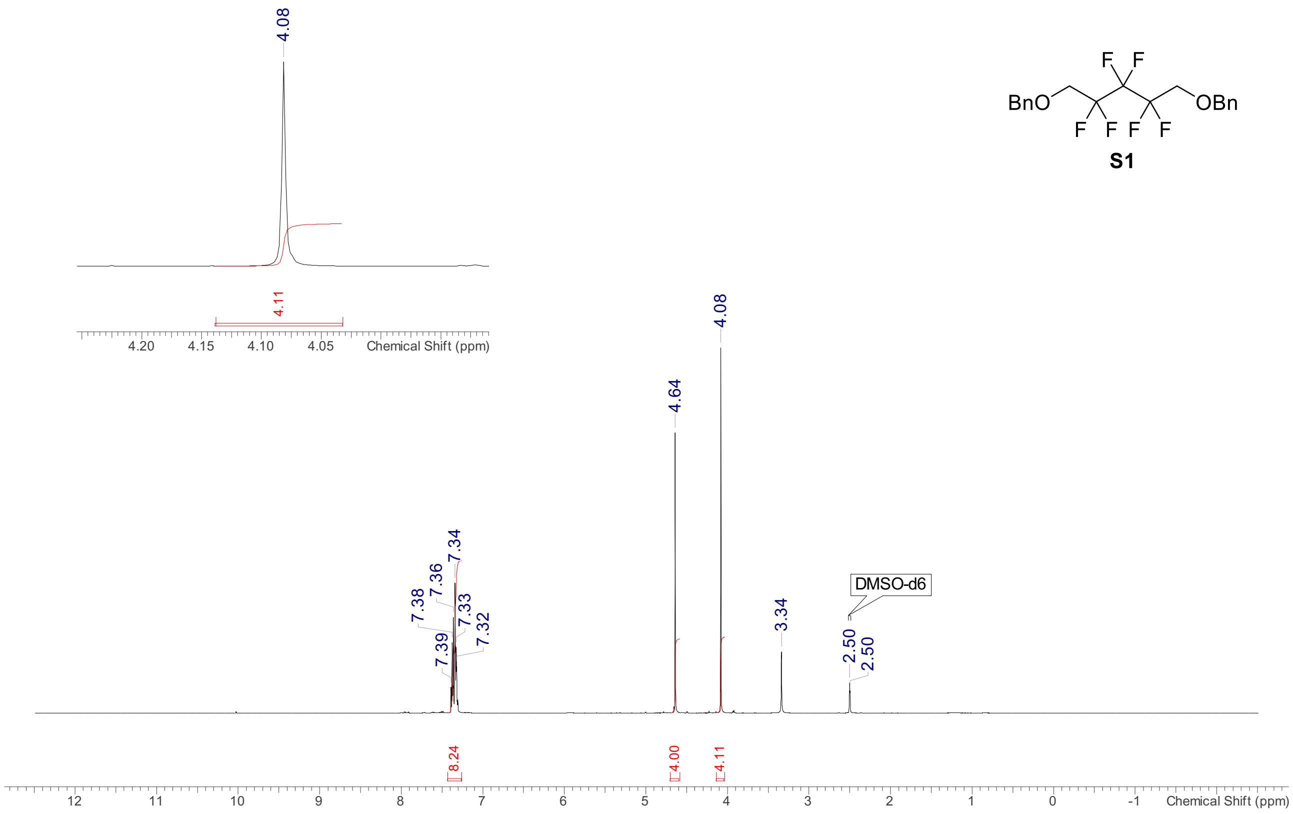

c) ^{19}F NMR (DMSO-d^6, 376 MHz)

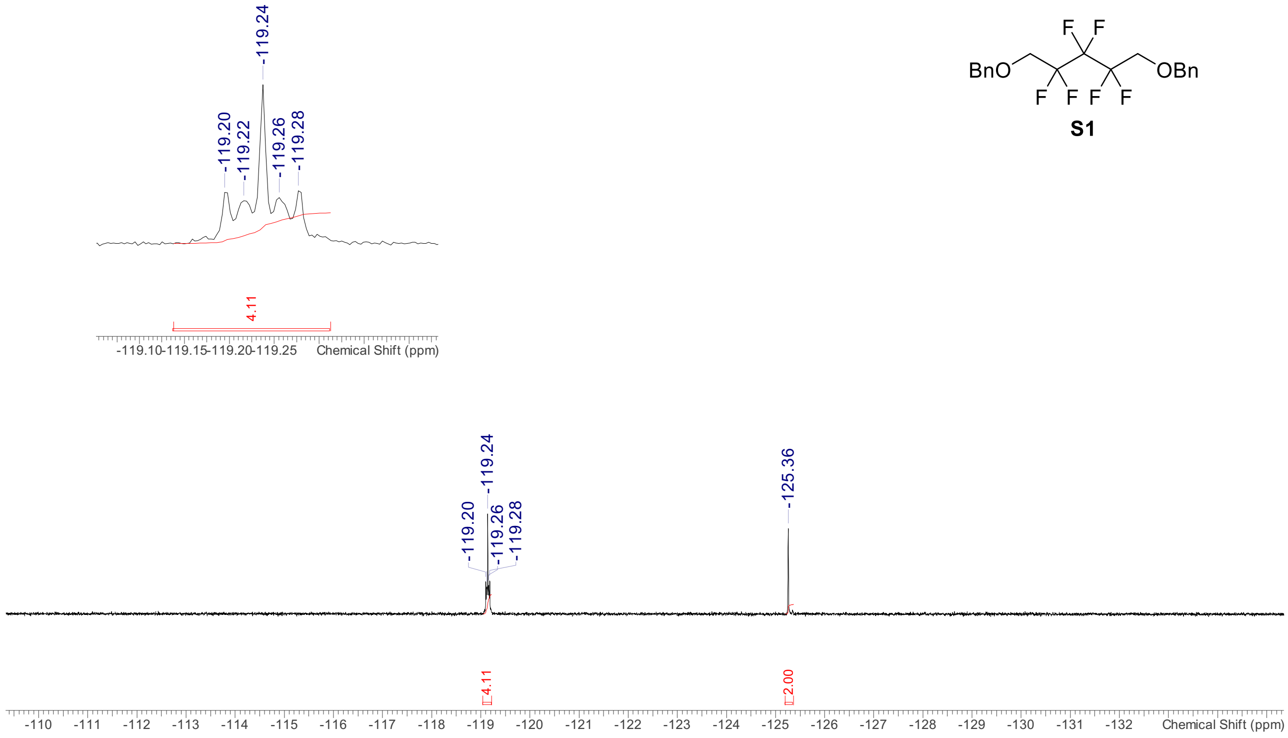

d) ^{19}F{^{1}H} NMR (DMSO-d^6, 471 MHz)

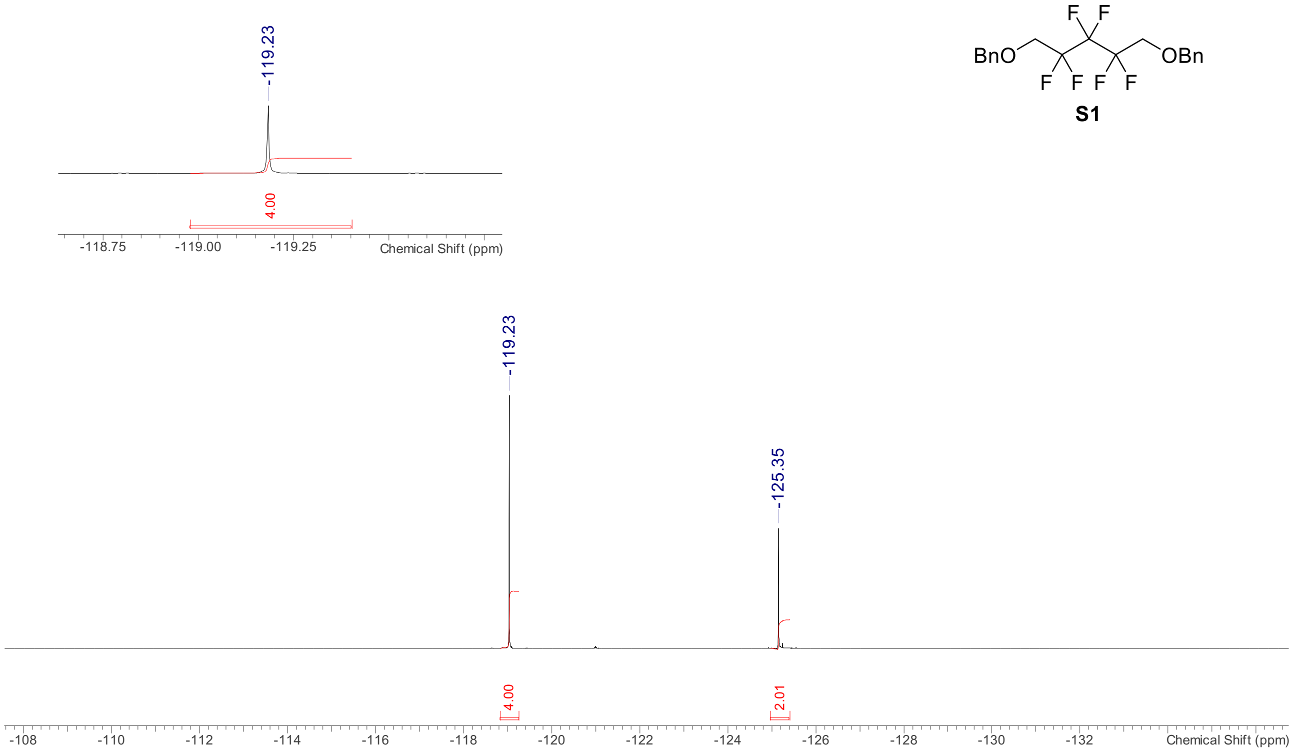

e) $^{13}C\{^{1}H\}$ NMR (DMSO-d^6, 101 MHz)

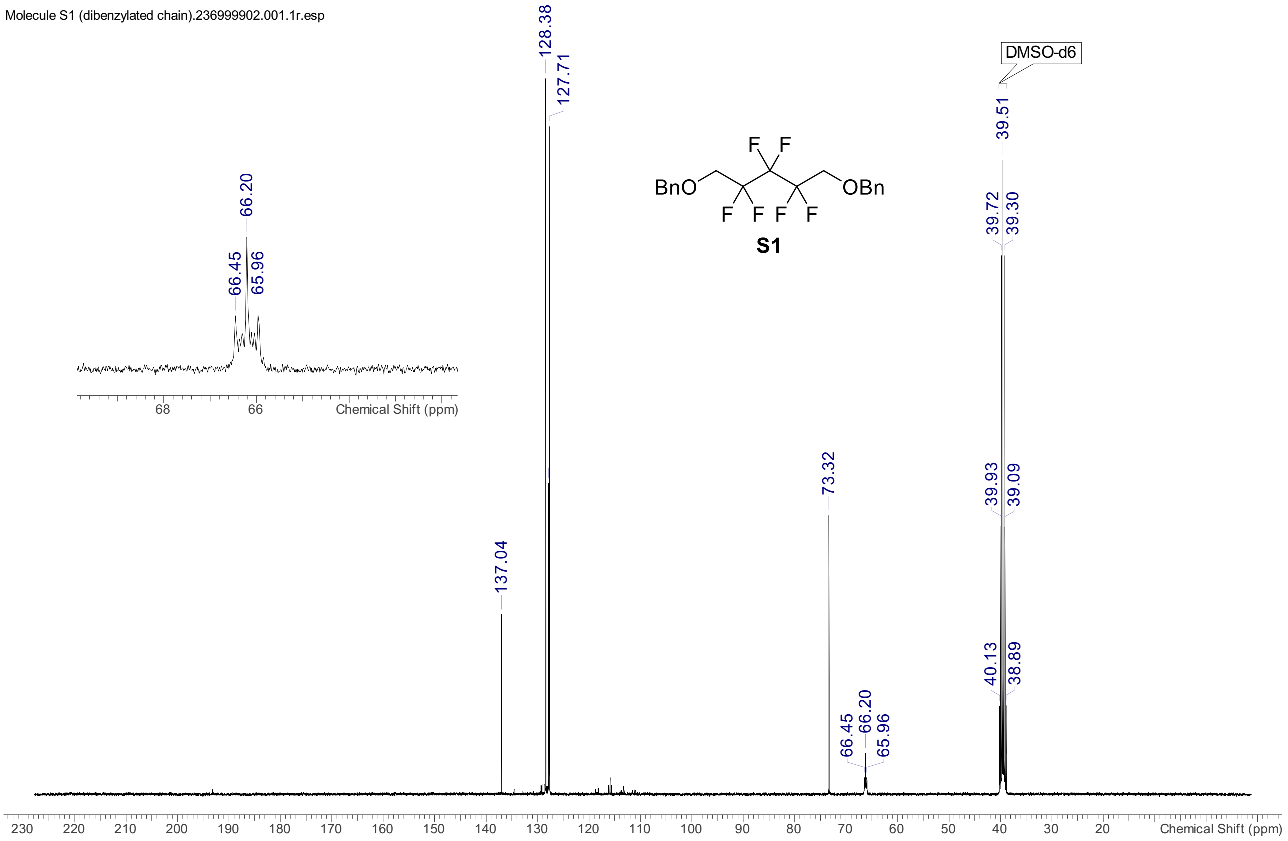

6.5 NMR spectra of 5-(benzyloxy)-2,2,3,3,4,4-hexafluoropentane-1,1-diol (25)

a) ^{1}H NMR (DMSO-d^{6}, 400 MHz)

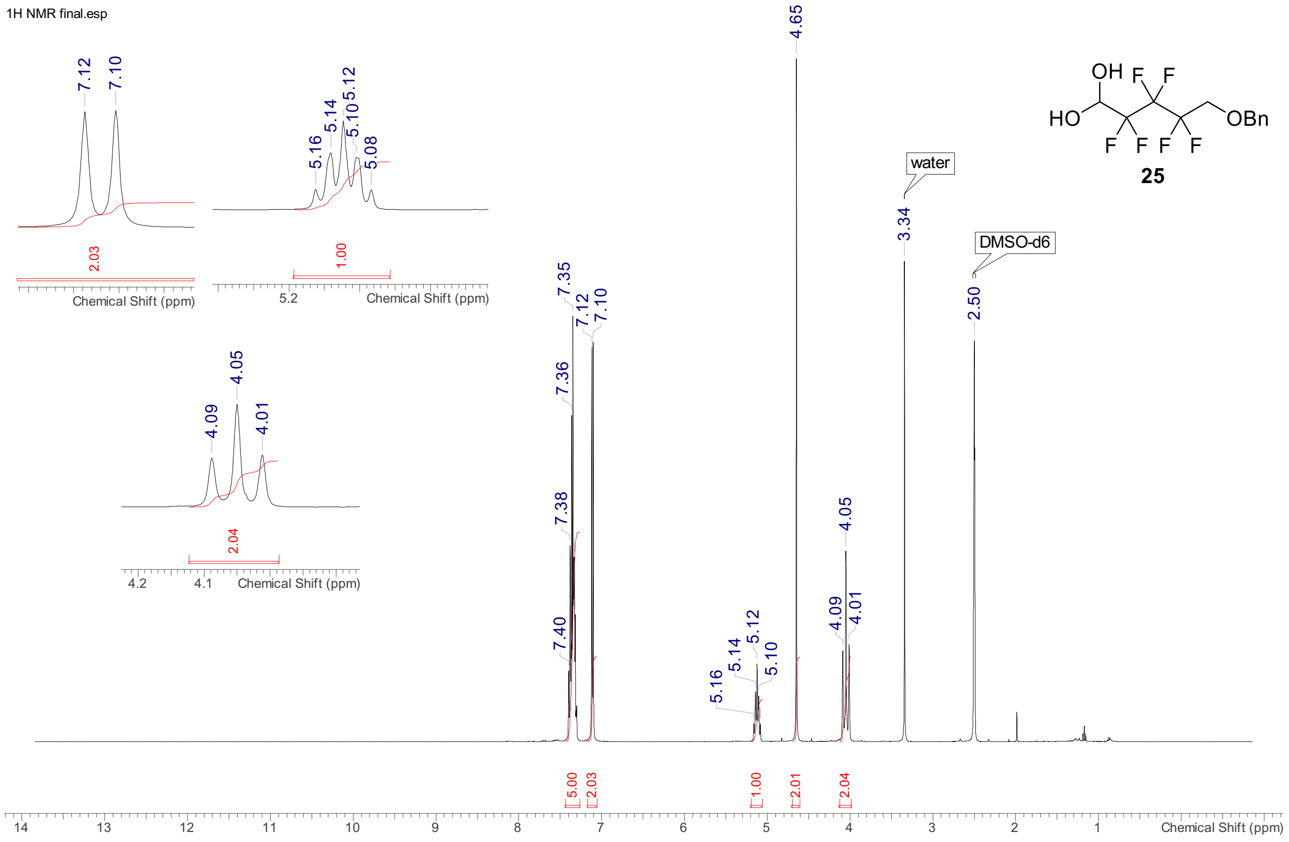

b) $^{1}H\{^{19}F\}$ NMR (DMSO-d^{6}, 500 MHz)

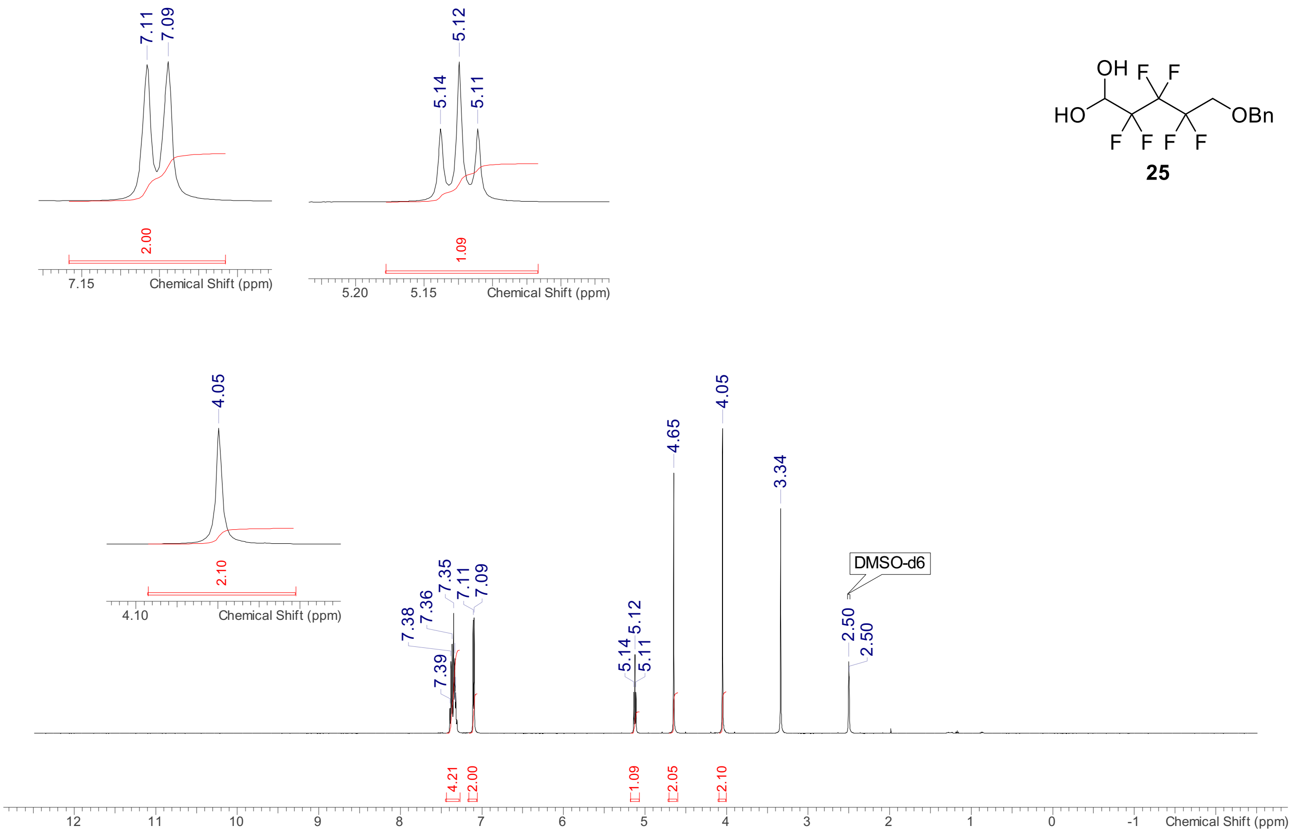

c) ^{19}F NMR (DMSO-d^6, 376 MHz)

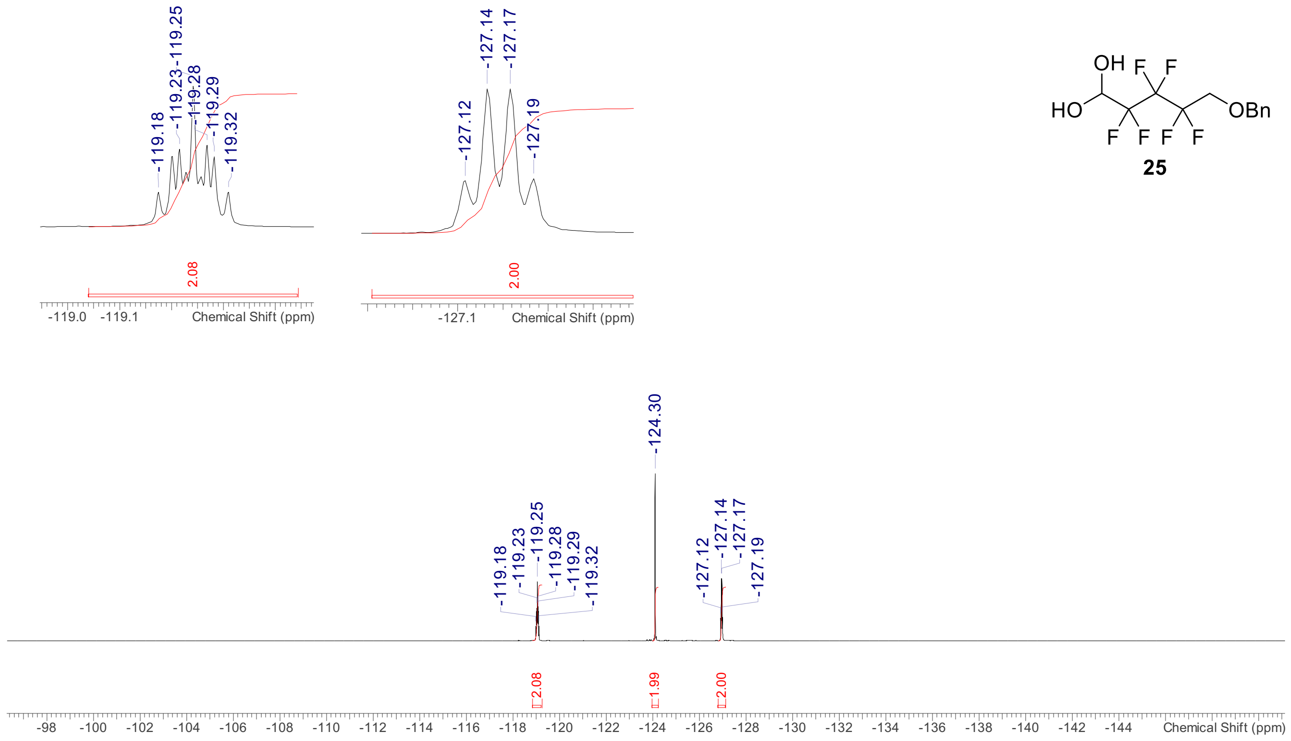

d) ^{19}F{^{1}H} NMR (DMSO-d^6, 471 MHz)

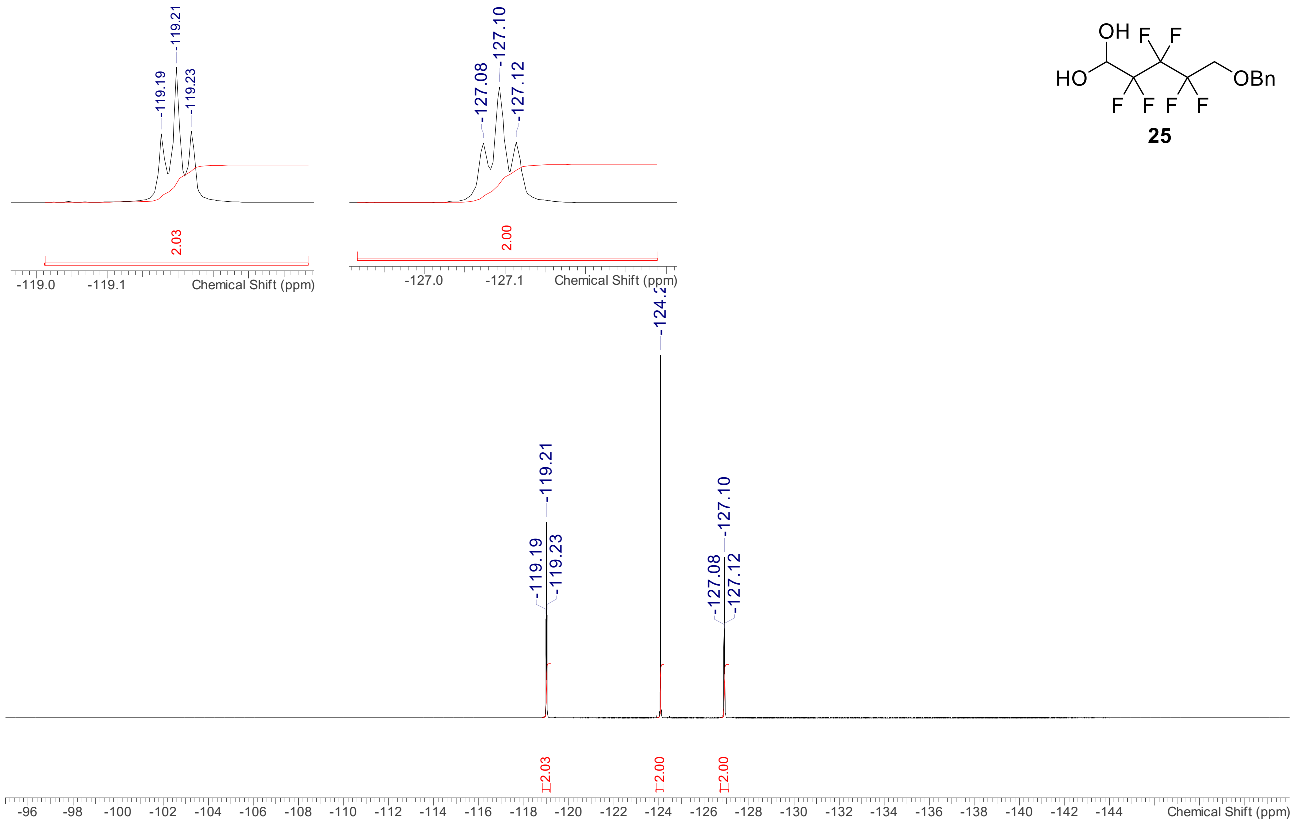

e) $^{13}C\{^1H\}$ NMR (DMSO-d^6, 101 MHz)

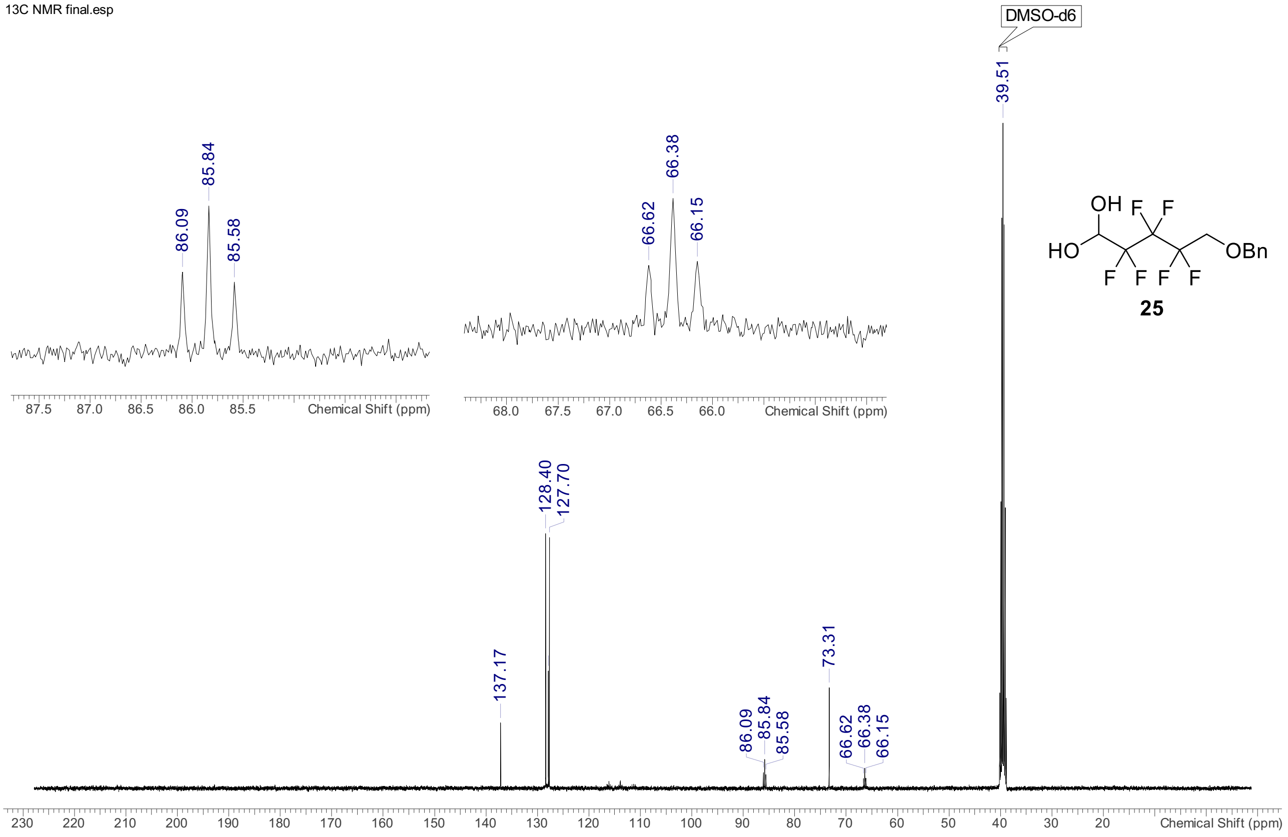

6.6 NMR spectra of methyl (*E*)-7-(benzyloxy)-4,4,5,5,6,6-hexafluorohept-2-enoate (26)

a) ^{1}H NMR (DMSO-d^6, 400 MHz)

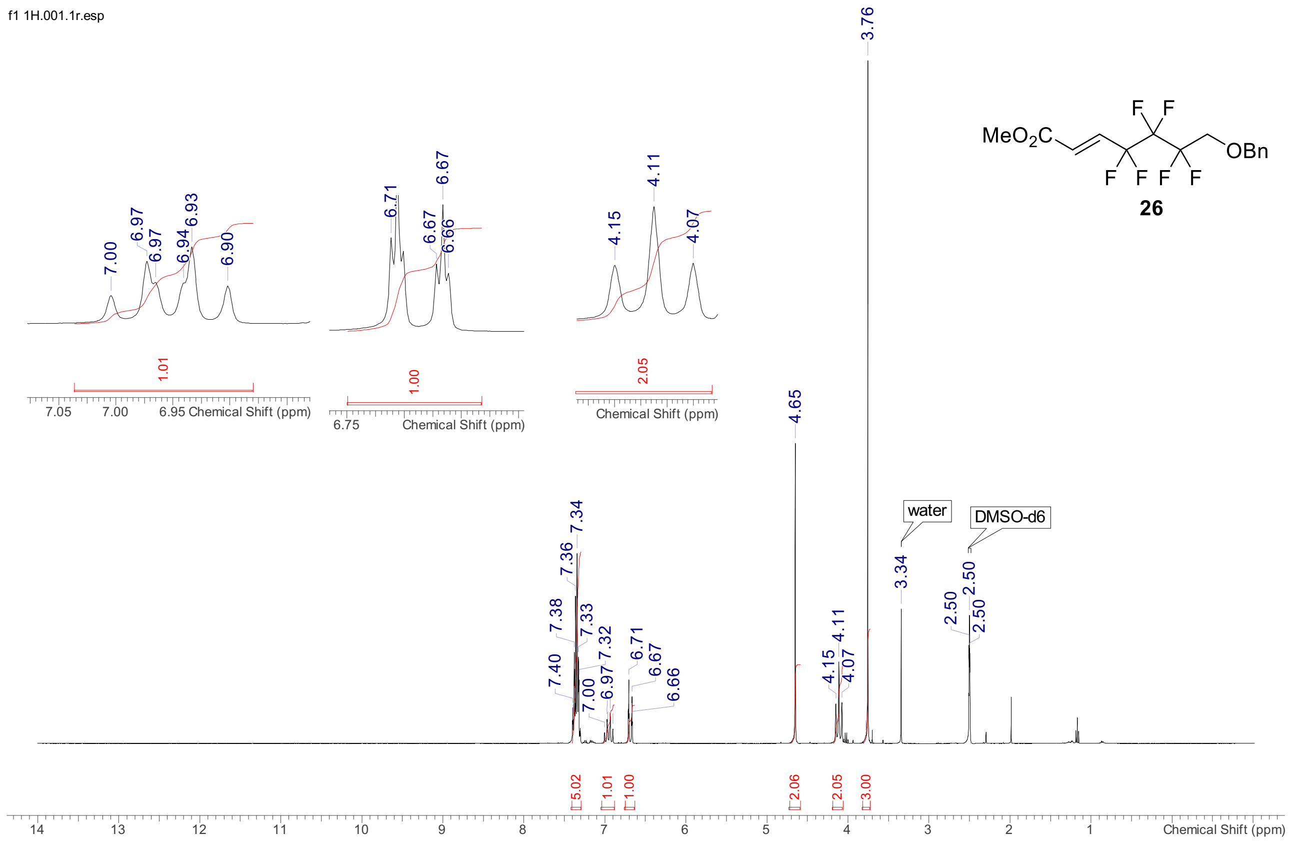

b) ^{1}H {^{19}F} NMR (DMSO-d^6, 500 MHz)

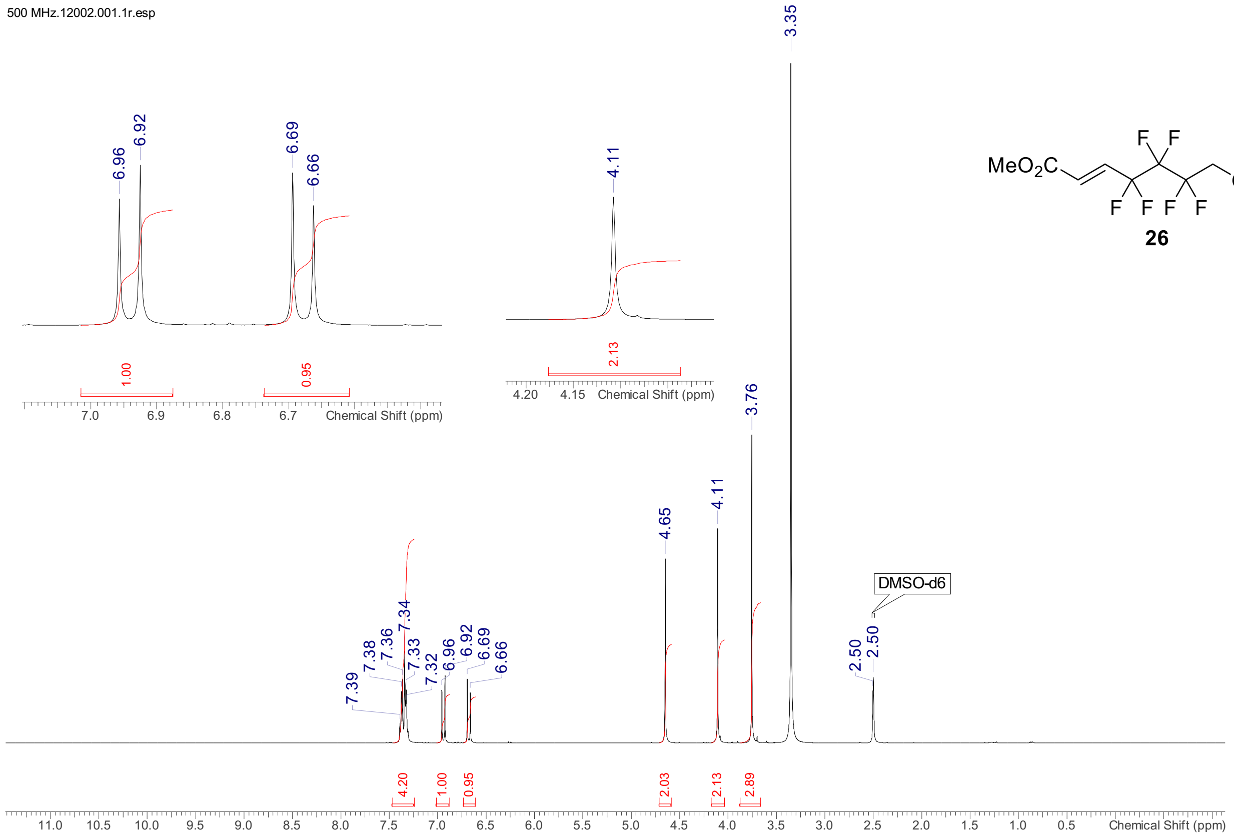

c) ^{19}F NMR (DMSO-d^6, 376 MHz)

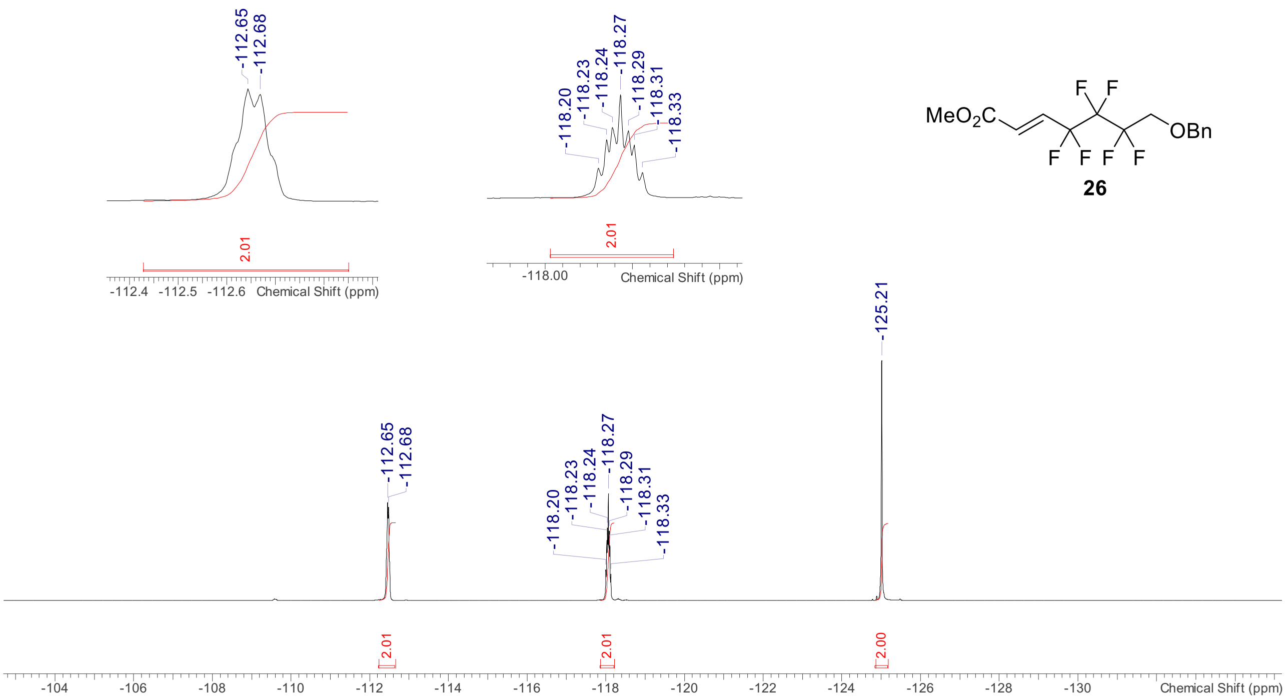

26

d) ^{19}F{^{1}H} NMR (DMSO-d^6, 471 MHz)

500 MHz.12004.001.1r.esp

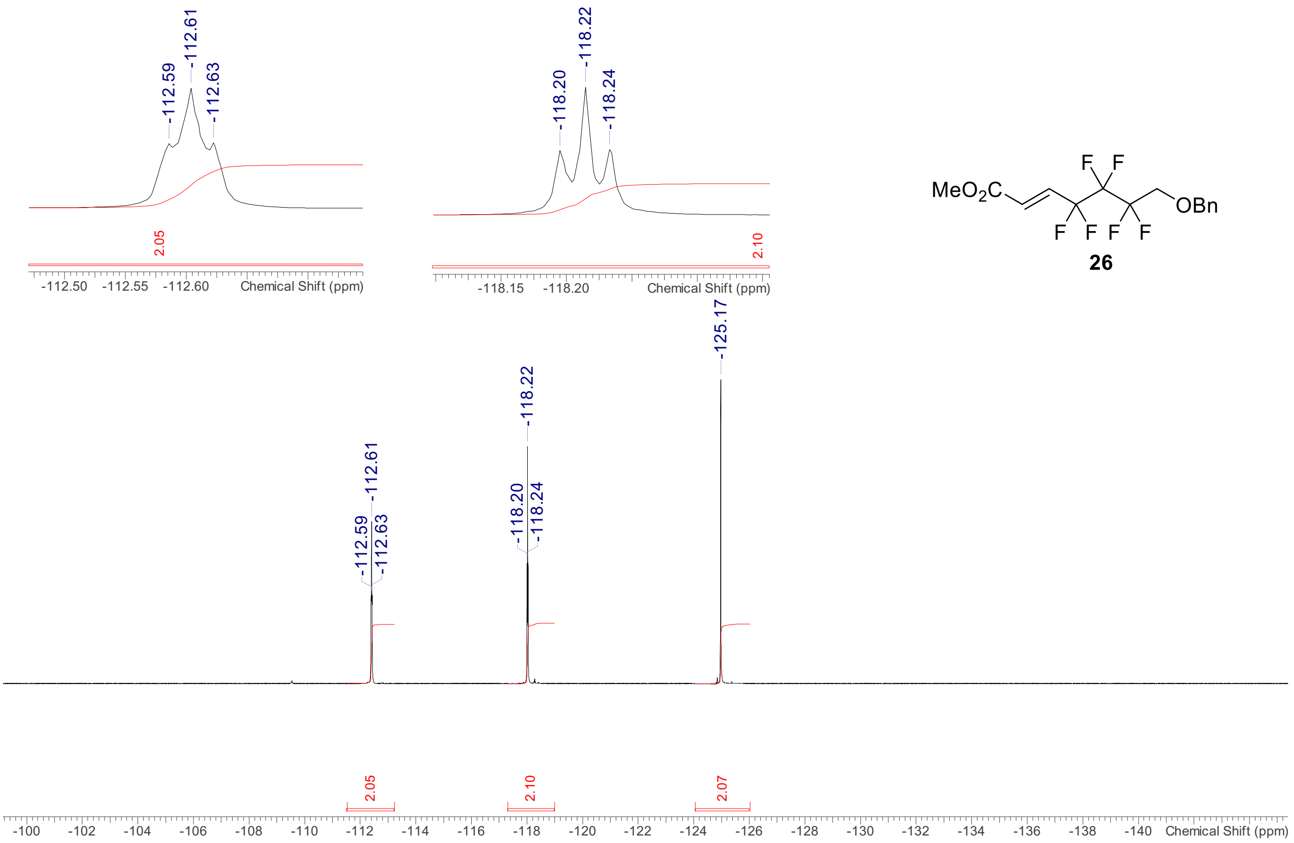

26

e) $^{13}C\{^{1}H\}$ NMR (DMSO-d^6, 101 MHz)

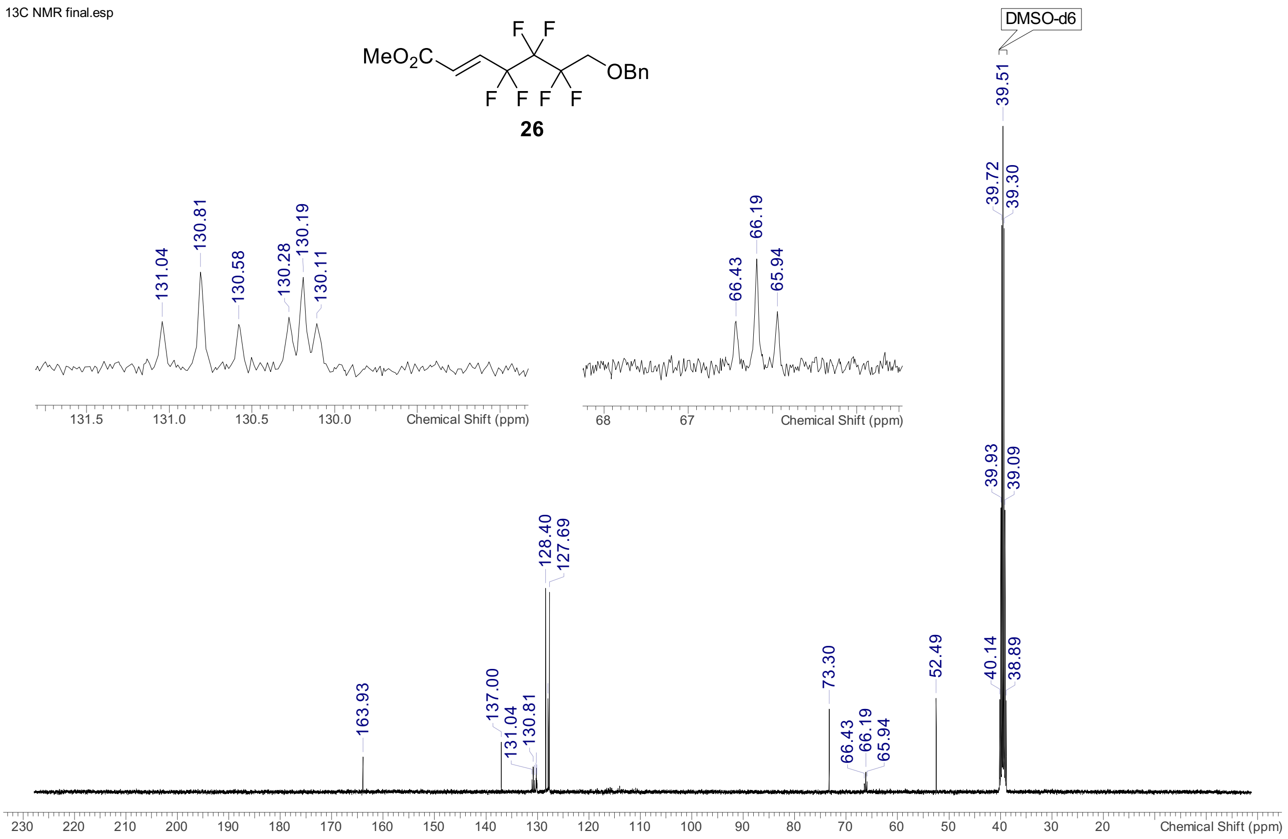

6.7 NMR spectra of (*E*)-7-(benzyloxy)-4,4,5,5,6,6-hexafluorohept-2-en-1-ol (27)

a) ^{1}H NMR (DMSO-d^6, 400 MHz)

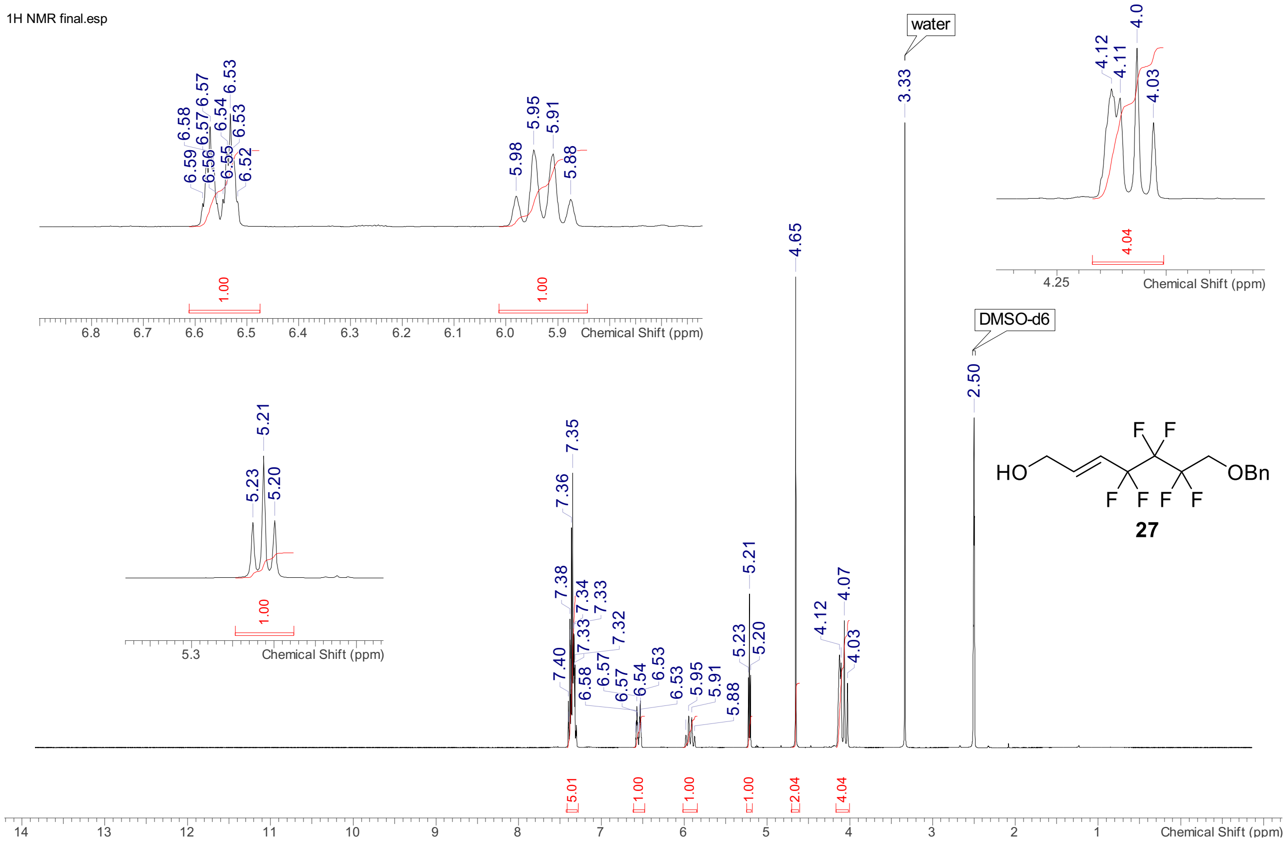

b) ^{1}H {^{19}F} NMR (DMSO-d^6, 500 MHz)

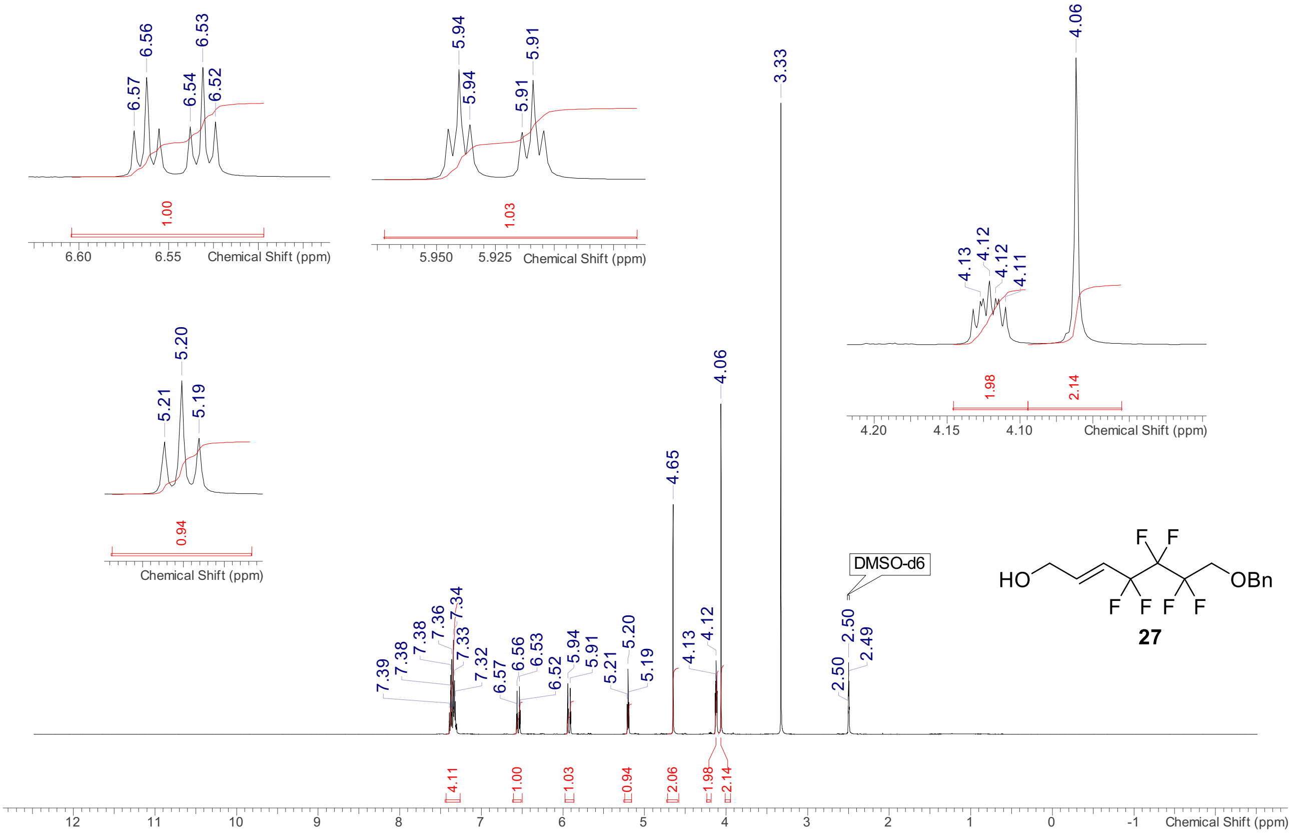

c) ^{19}**F NMR (DMSO-*d*^{6}, 376 MHz)**

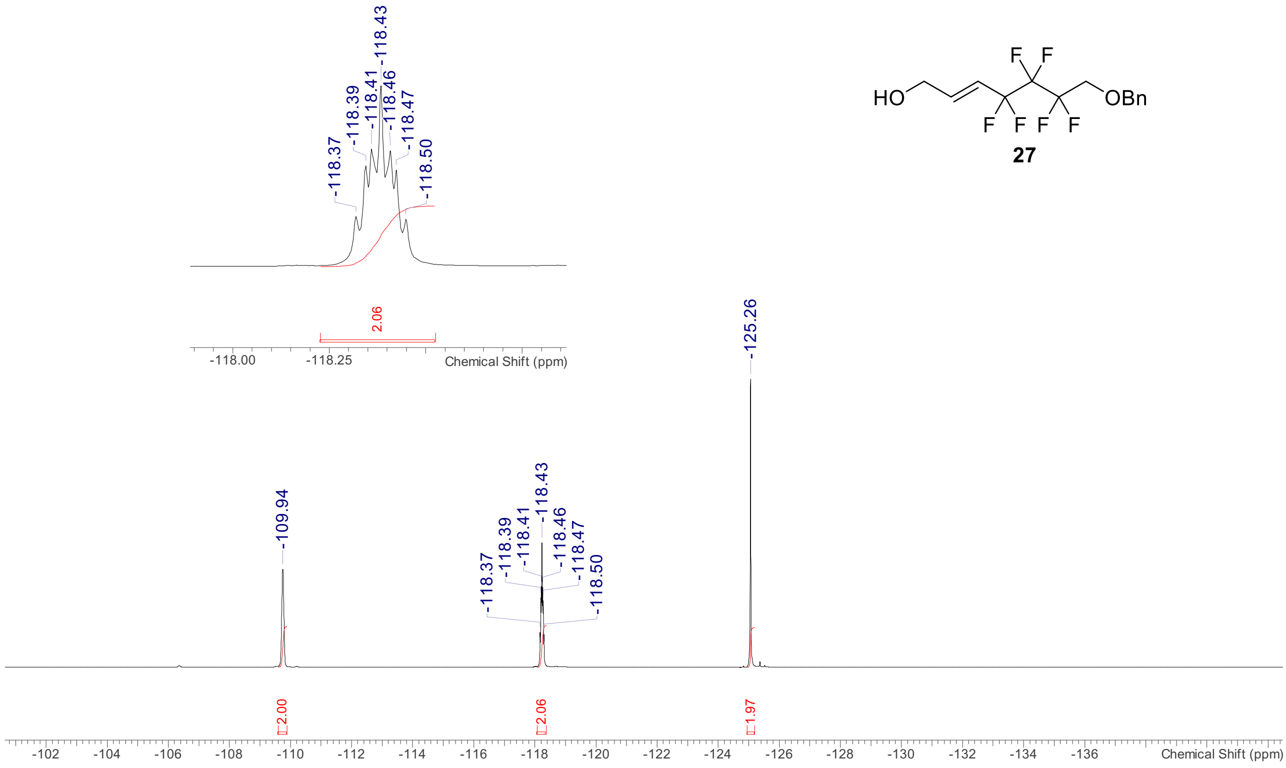

27

d) ^{19}**F{^{1}H} NMR (DMSO-*d*^{6}, 471 MHz)**

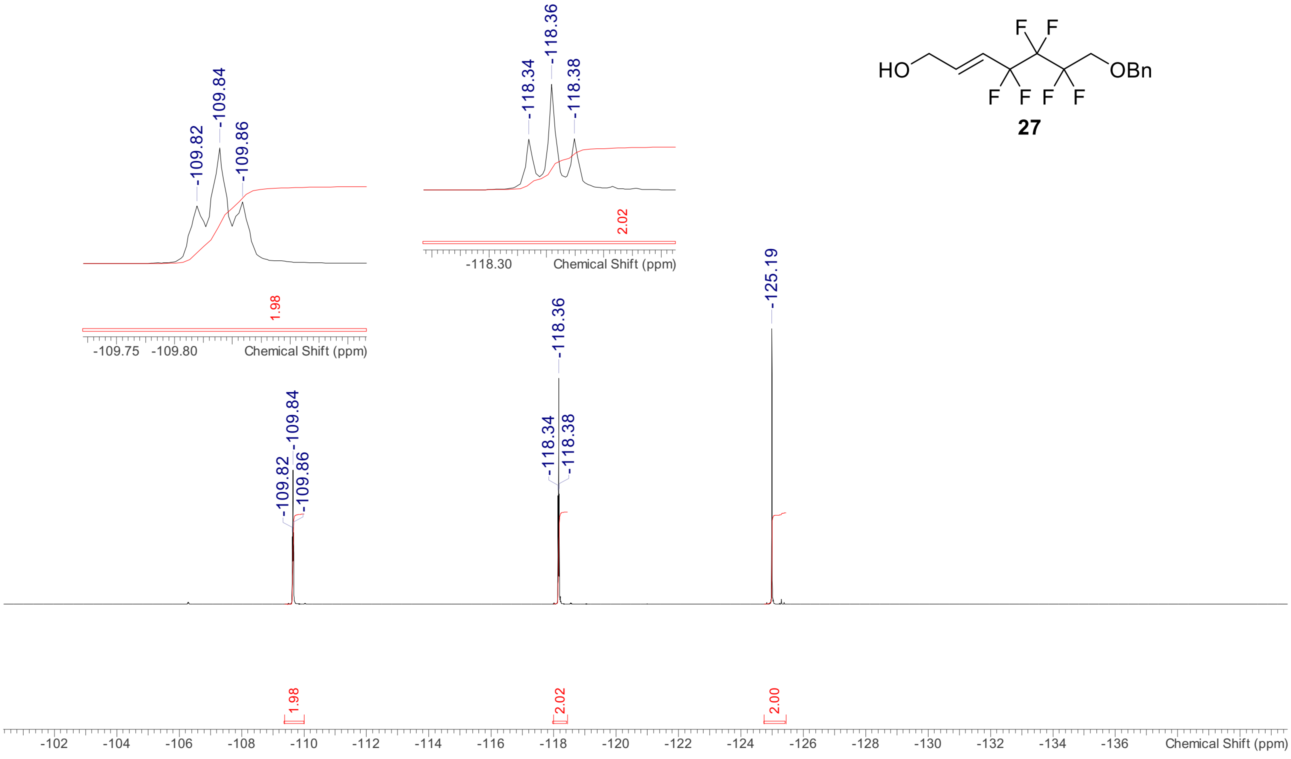

27

e) $^{13}C\{^1H\}$ NMR (DMSO-d^6, 101 MHz)

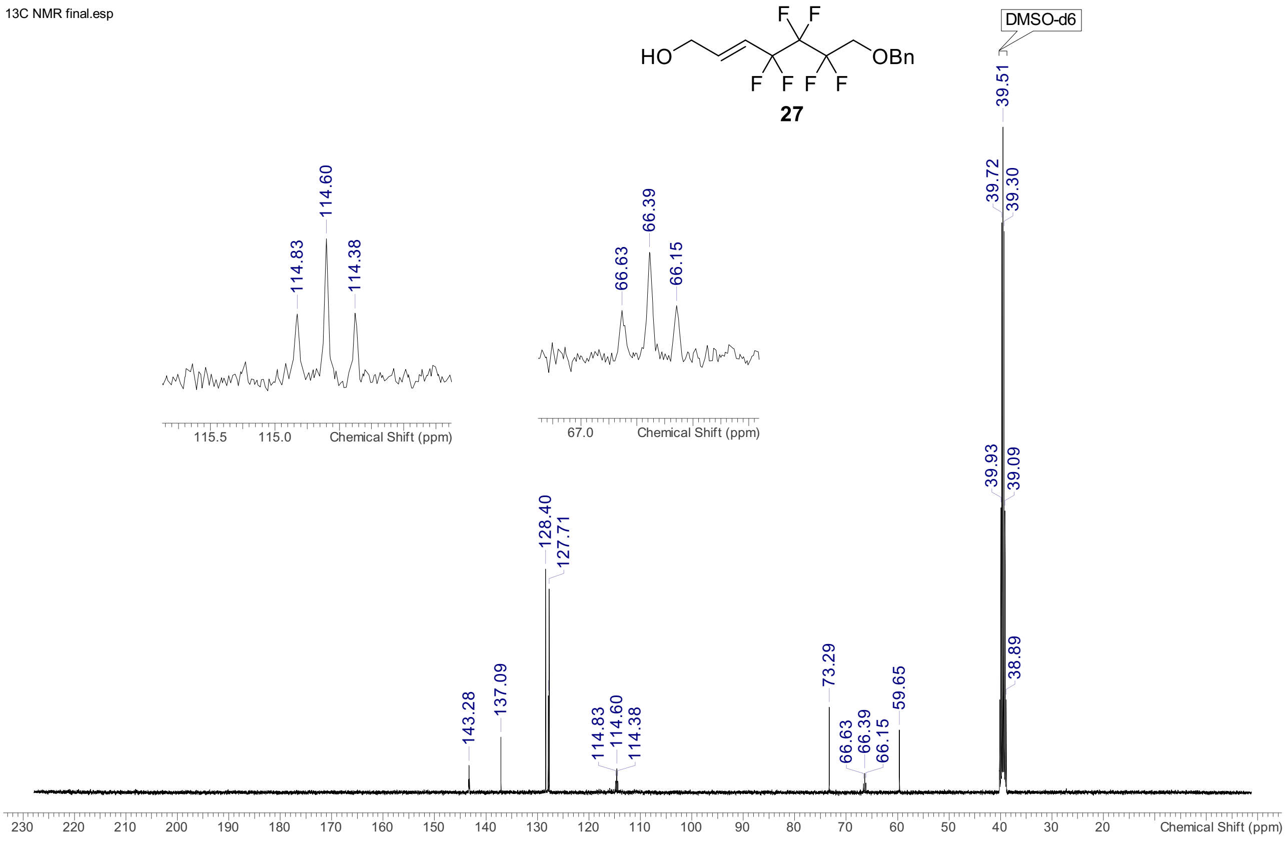

6.8 NMR spectra of (*E*)-1,7-bis(benzyloxy)-4,4,5,5,6,6-hexafluorohept-2-ene (14)

a) ^{1}H NMR (DMSO-d^6, 400 MHz)

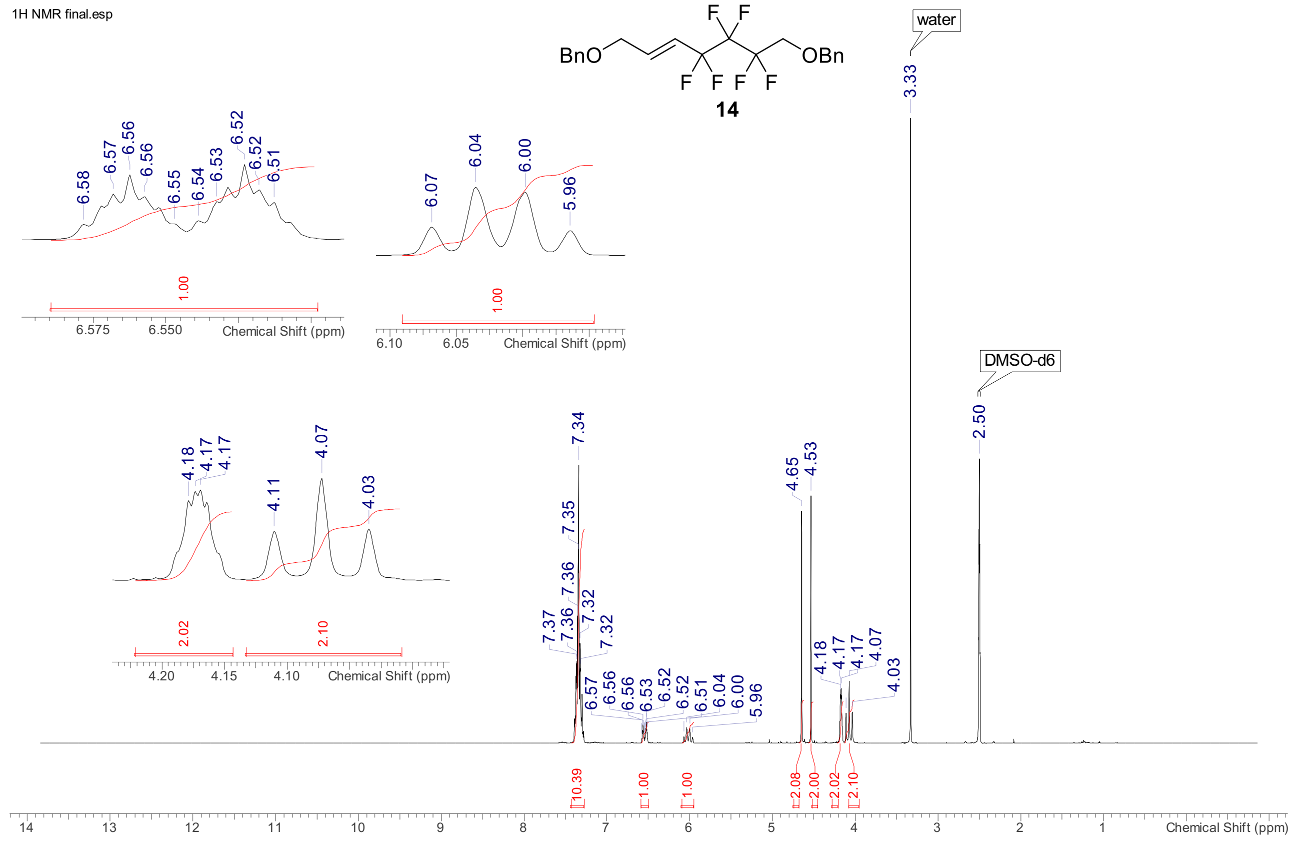

b) ^{1}H {^{19}F} NMR (DMSO-d^6, 500 MHz)

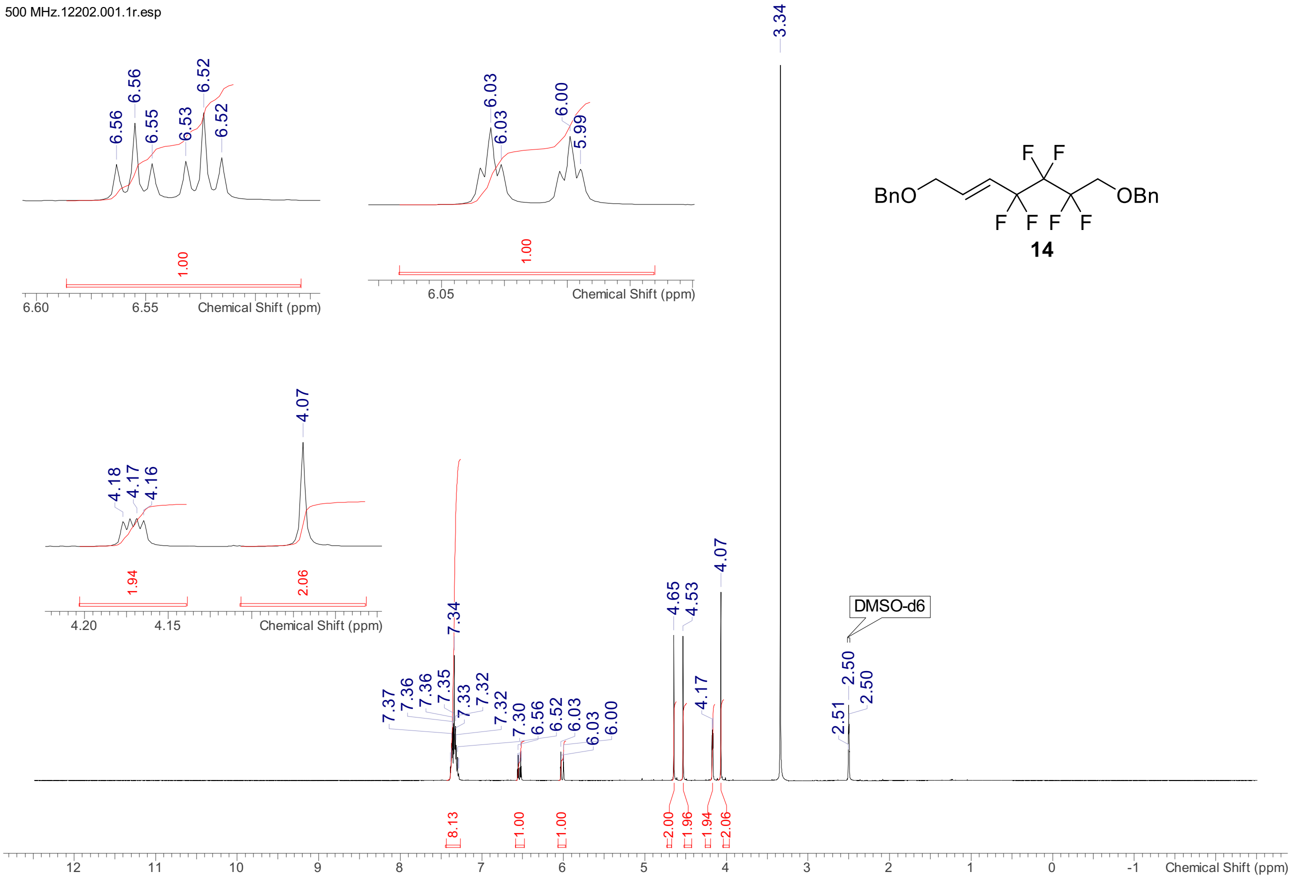

c) ^{19}F NMR (DMSO-*d*[6], 376 MHz)

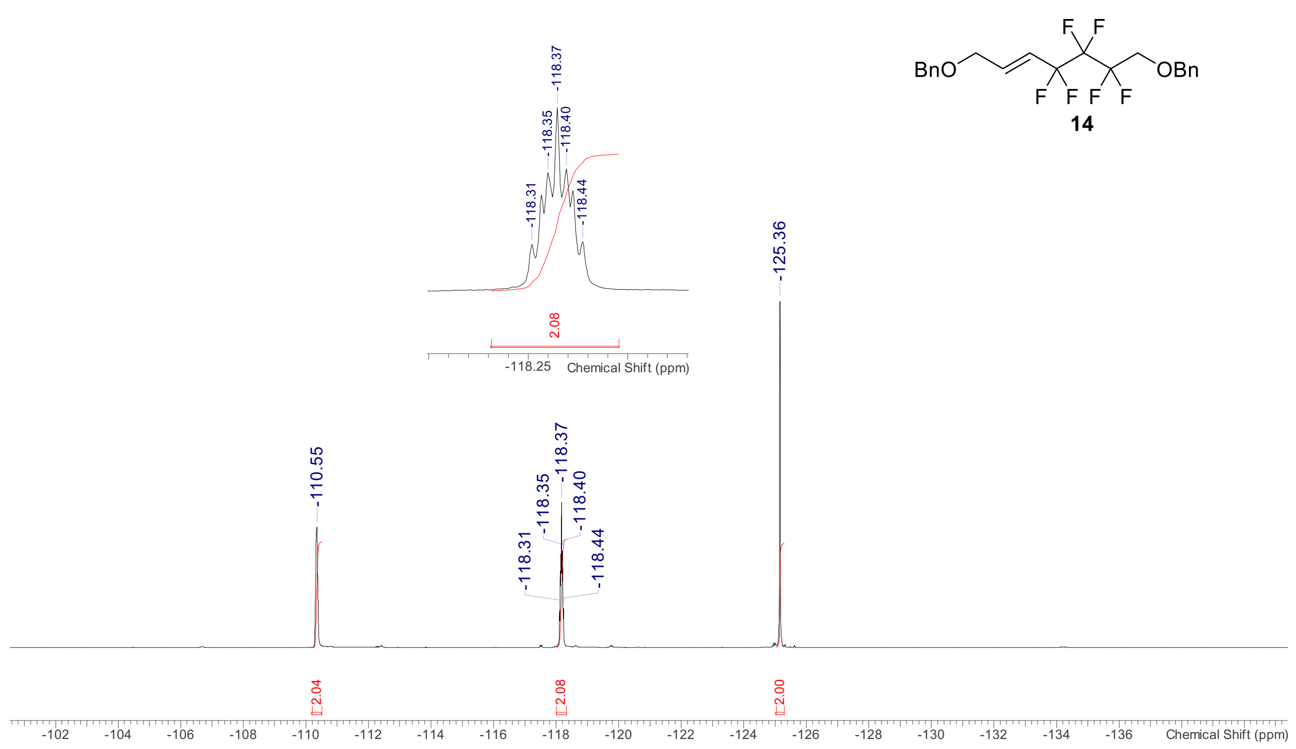

d) ^{19}F{^{1}H} NMR (DMSO-*d*[6], 471 MHz)

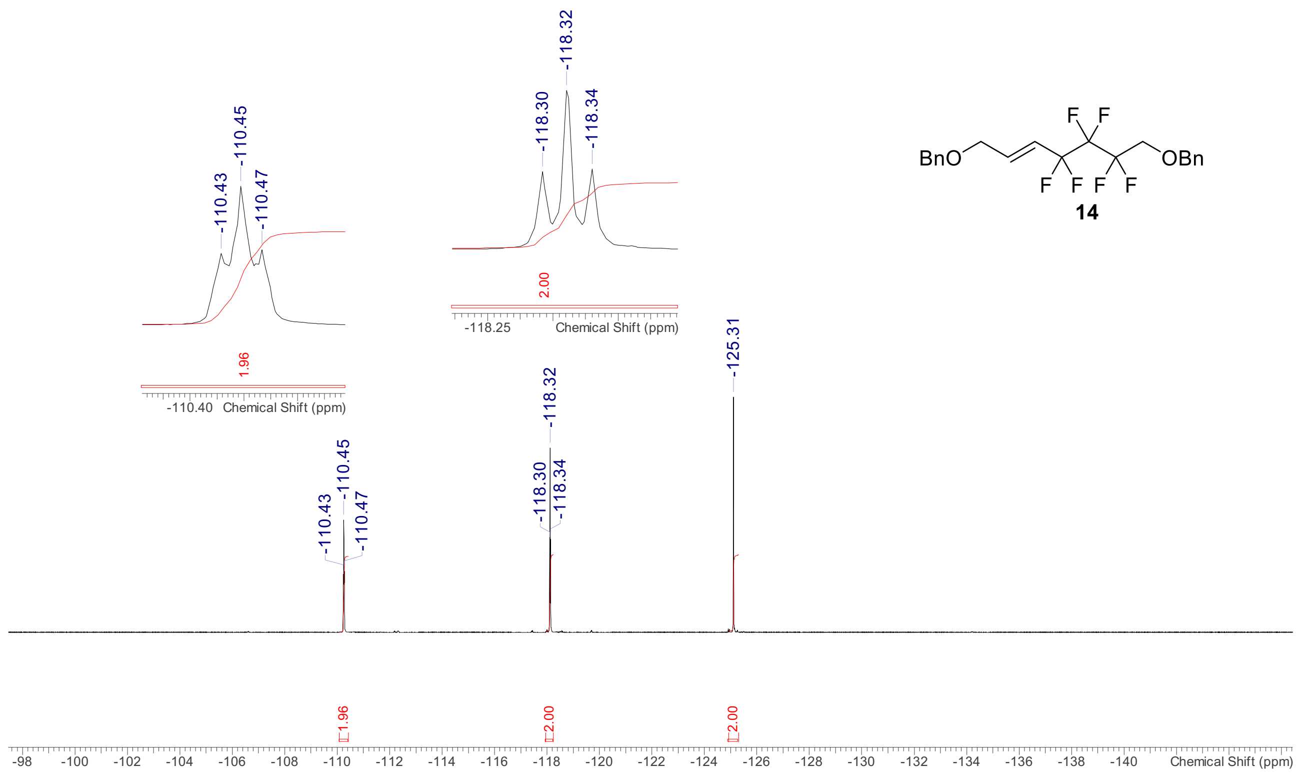

e) $^{13}C\{^1H\}$ NMR (DMSO-*d*^{6}, 101 MHz)

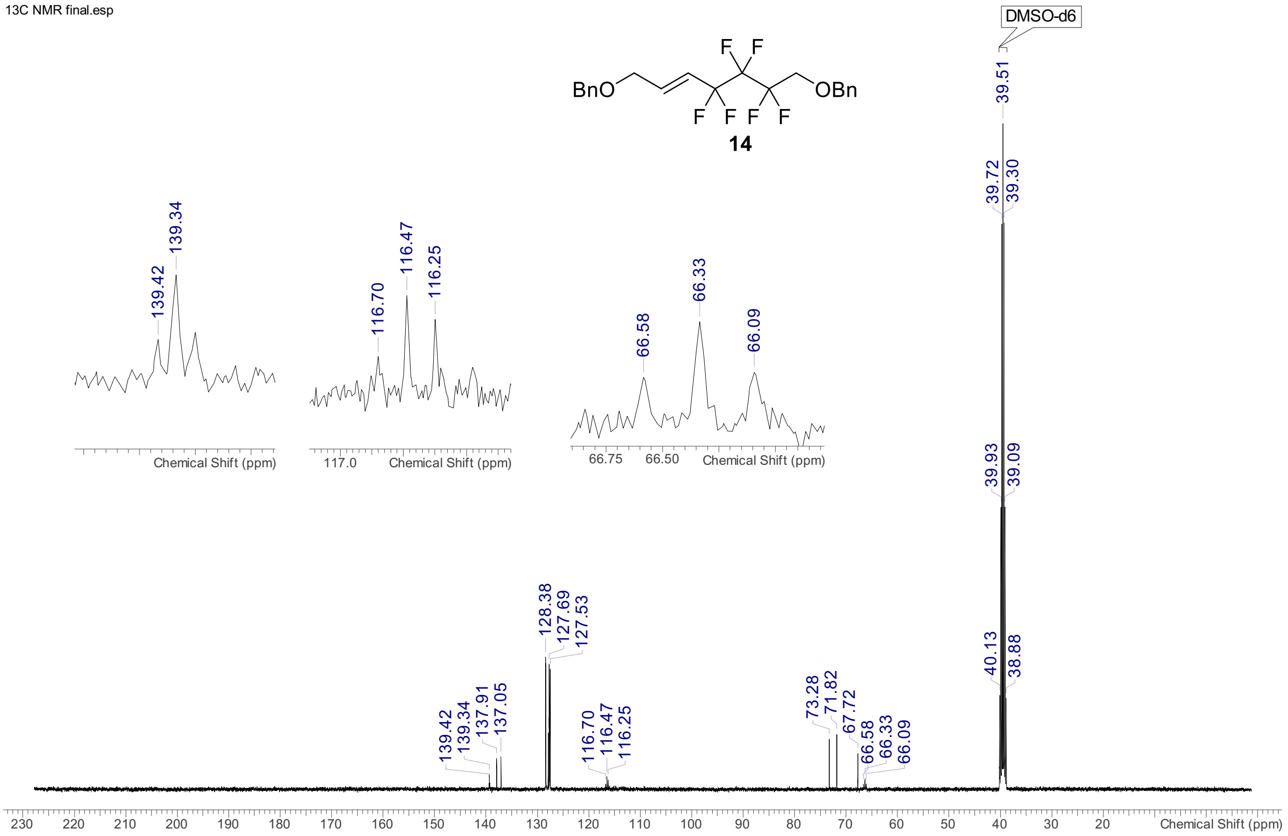

6.9 NMR spectra of (2*S*,3*R*)-1,7-bis(benzyloxy)-4,4,5,5,6,6-hexafluoroheptane-2,3-diol (28)

a) ^{1}H NMR (DMSO-*d*^{6}, 400 MHz)

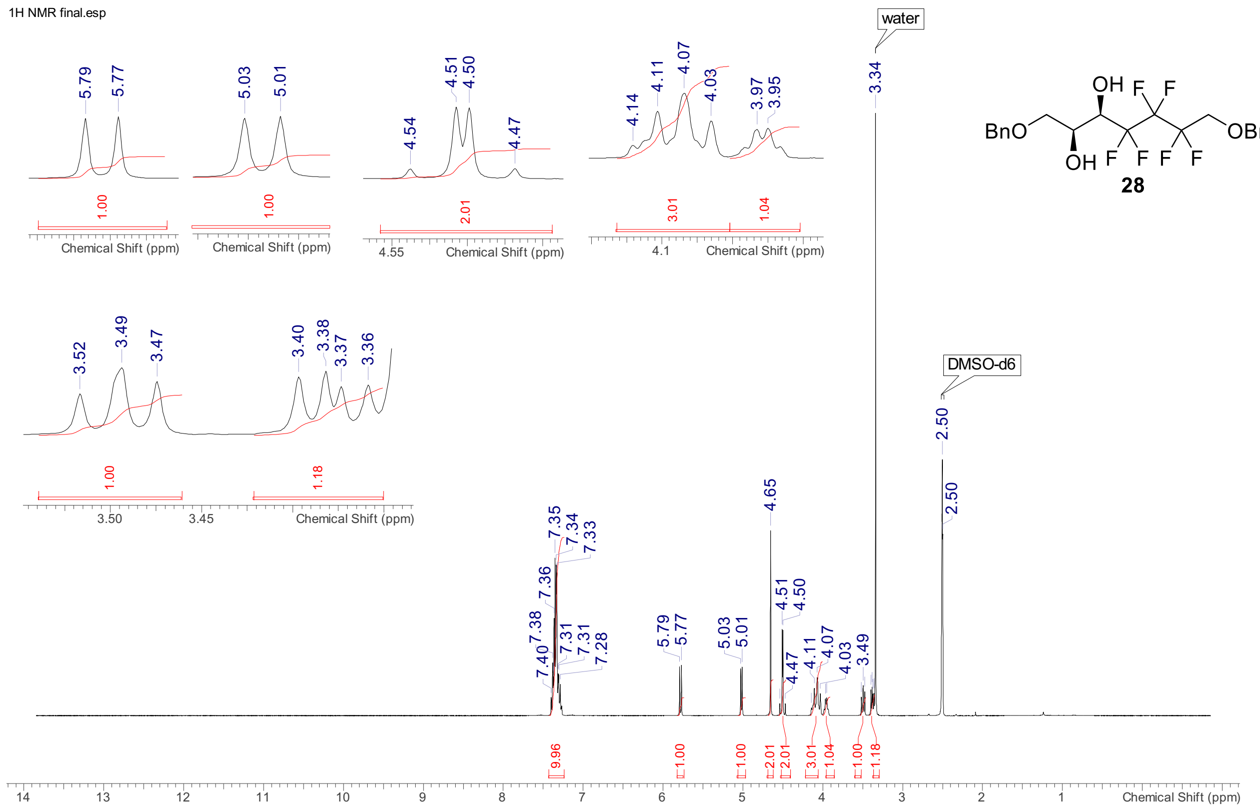

b) ^{1}H {^{19}F} NMR (DMSO-*d*^{6}, 500 MHz)

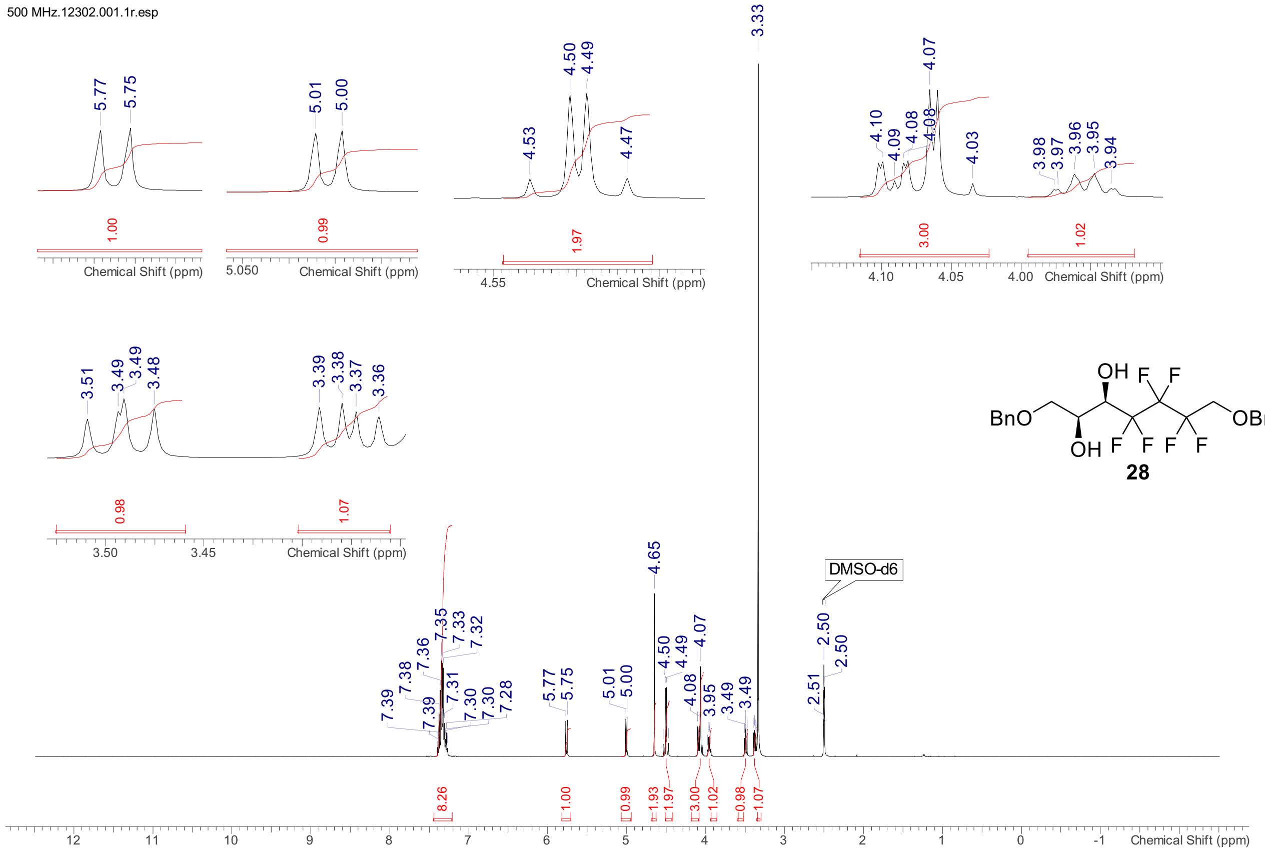

c) ^{19}F NMR (DMSO-d^6, 376 MHz)

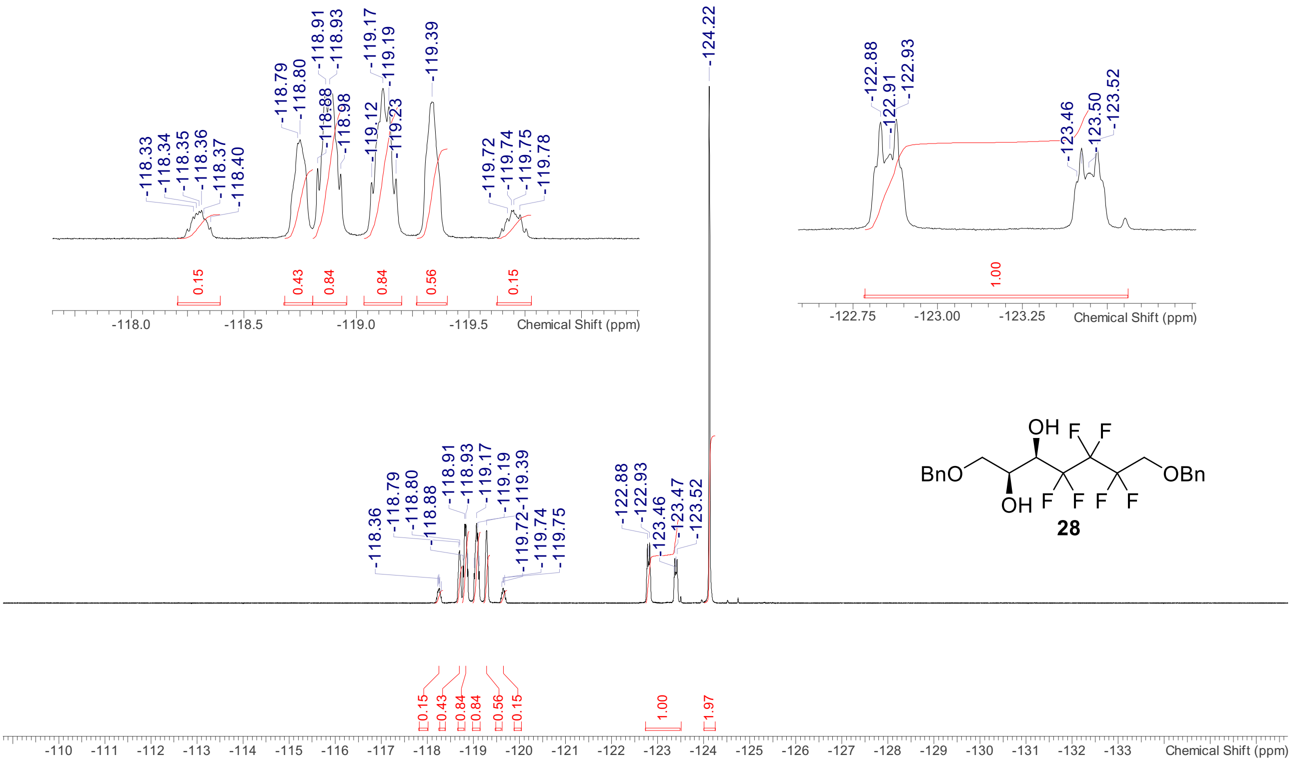

d) ^{19}F{^{1}H} NMR (DMSO-d^6, 471 MHz)

500 MHz.12304.001.1r.esp

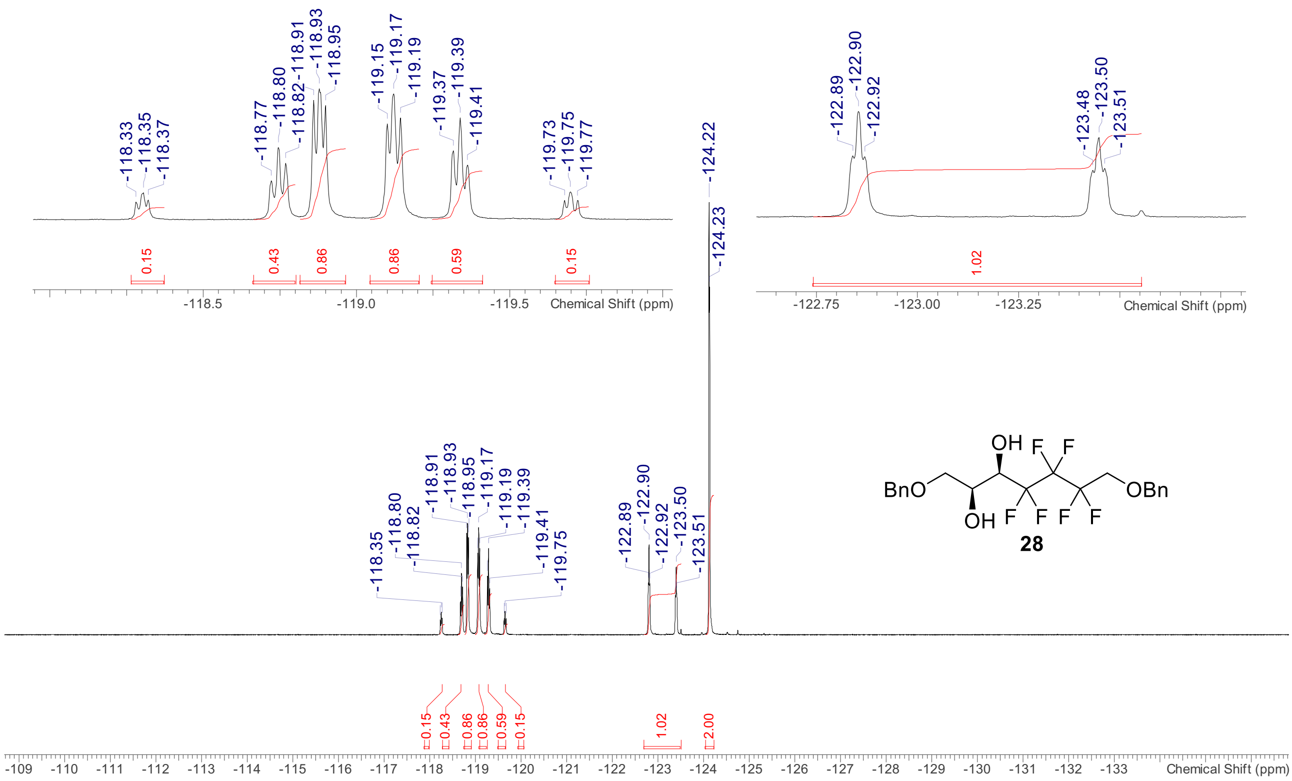

e) $^{13}C\{^{1}H\}$ NMR (DMSO-d^6, 101 MHz)

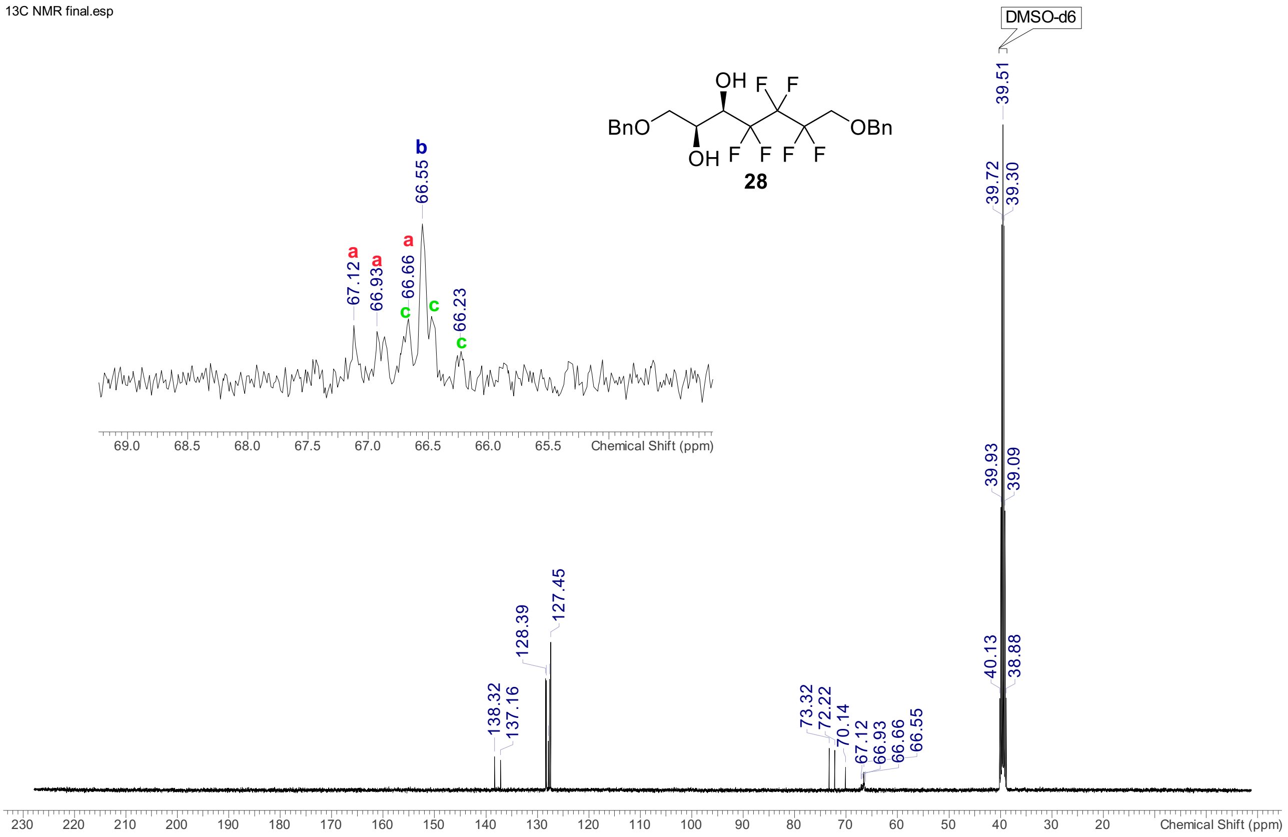

Using HSQC-DEPT analysis, it could be confirmed that **C-1** and **C-2** (being multiplets because of coupling with fluorine atoms), as well as **C-3** are overlapping in the 67.1-66.2 ppm range (see zoomed view of the spectrum **e)** above). When zoomed enough, the HSQC-DEPT spectrum allows unambiguous identification of these three overlapping carbon atoms: signal of **C-2** (doublet of doublet) is marked with **a**, signal of **C-3** (singlet) is marked with **b**, and signal of **C-1** (triplet) is marked with **c**.

6.10 NMR spectra of (2*S*,3*R*)-4,4,5,5,6,6-hexafluoroheptane-1,2,3,7-tetraol (29)

a) ^{1}H NMR (DMSO-*d*^{6}, 400 MHz)

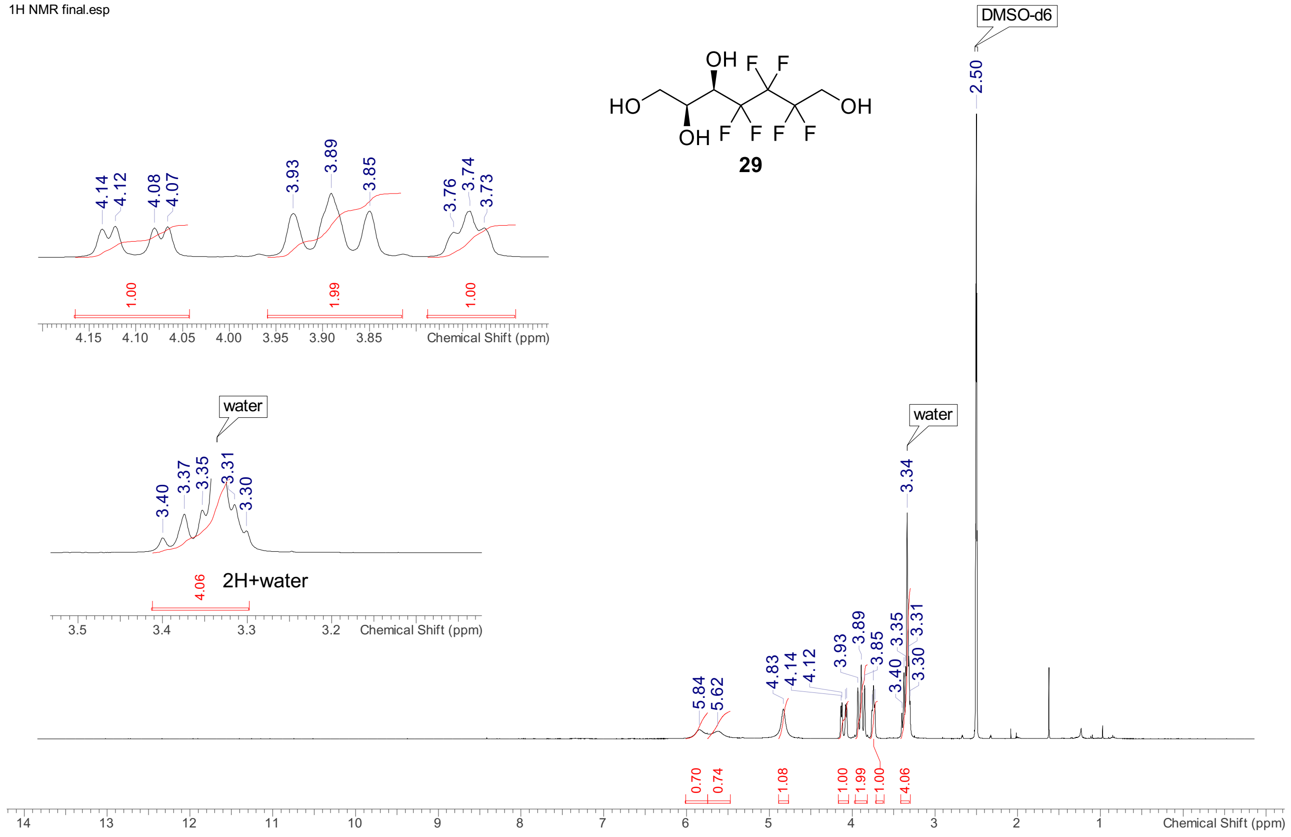

b) ^{1}H {^{19}F} NMR (DMSO-*d*^{6}, 500 MHz)

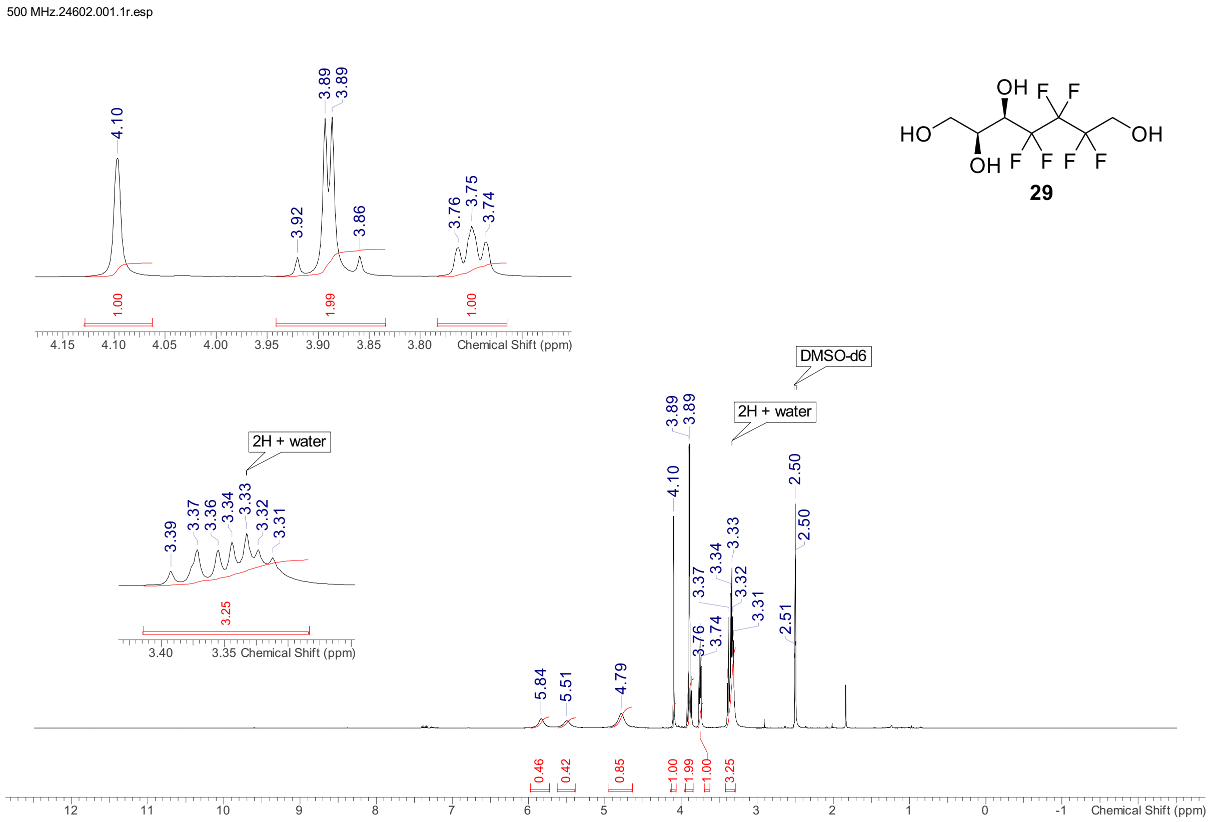

c) ^{19}F NMR (DMSO-d^6, 376 MHz)

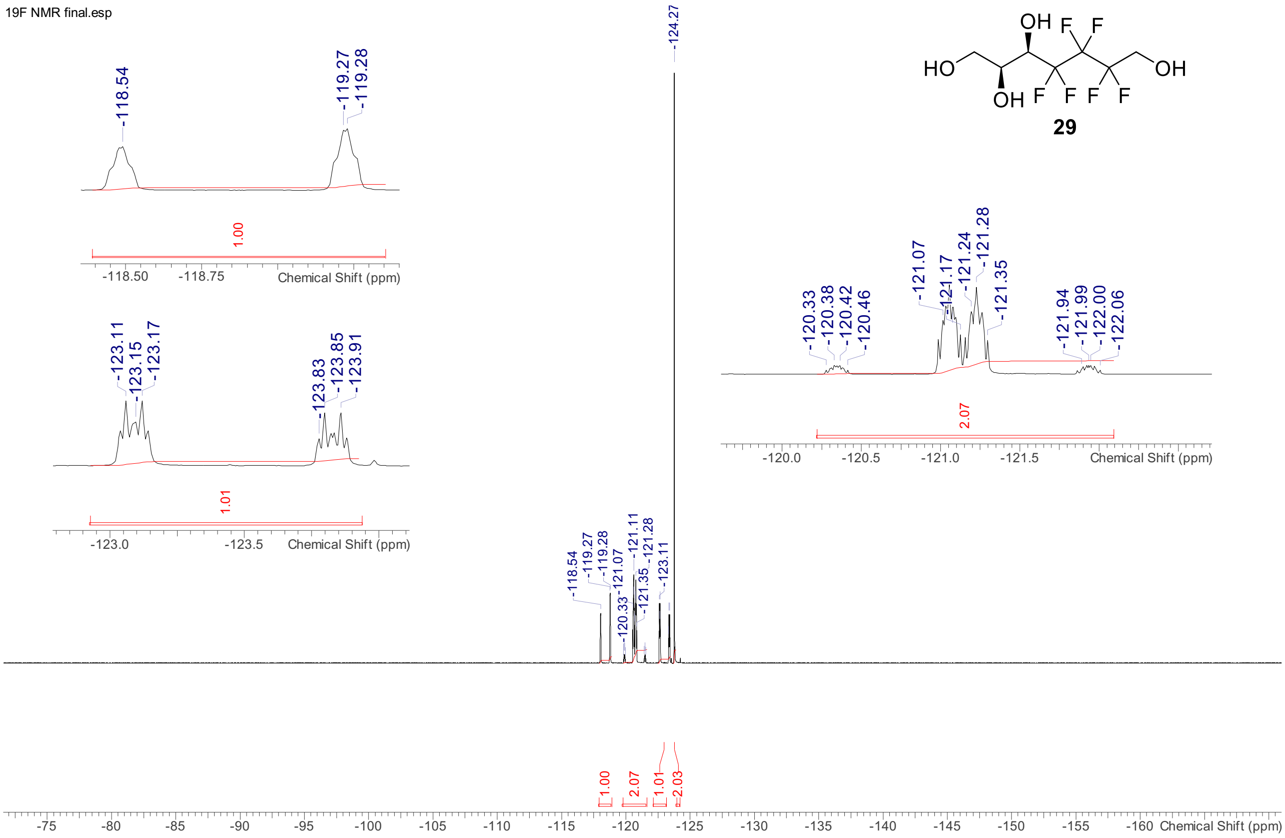

d) ^{19}F{^{1}H} NMR (DMSO-d^6, 471 MHz)

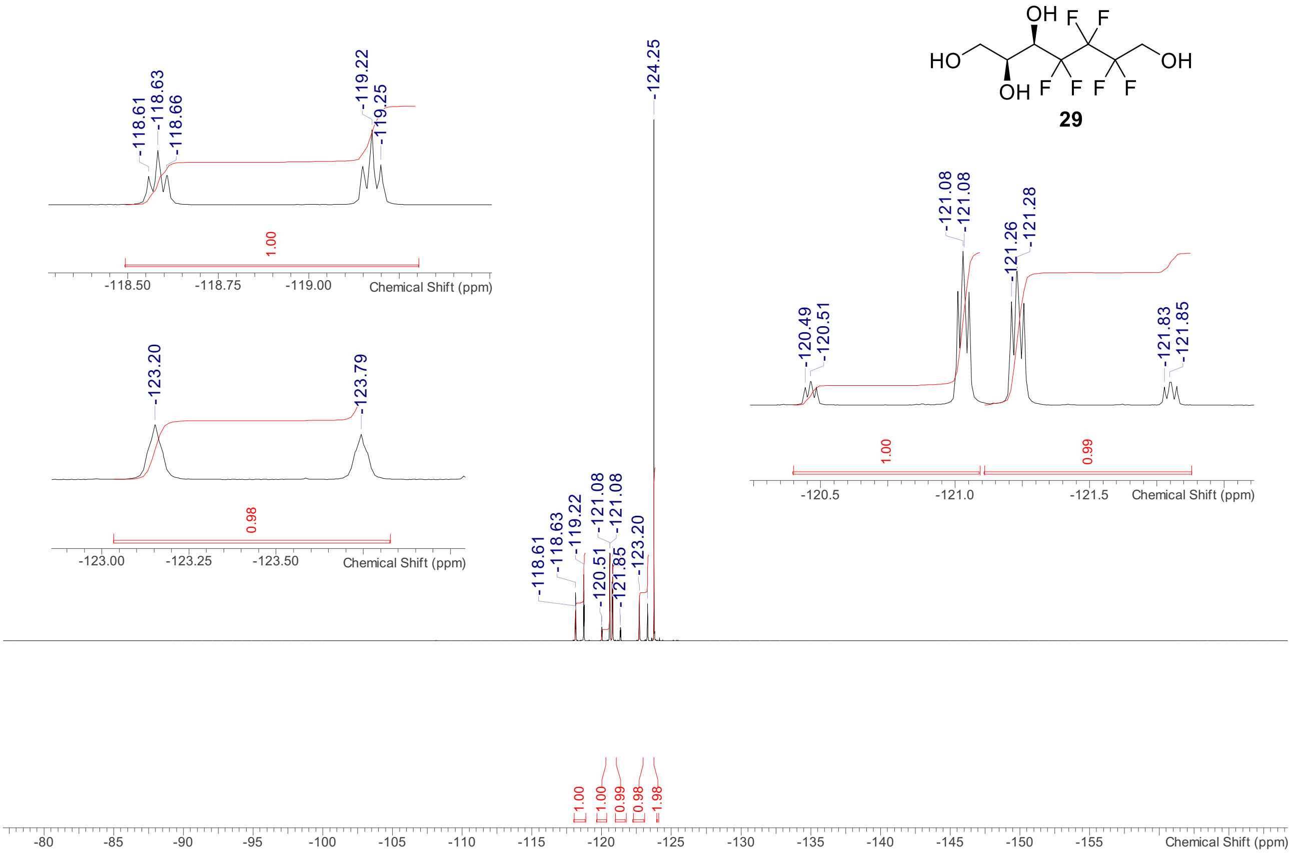

e) $^{13}C\{^{1}H\}$ NMR (DMSO-d^6, 101 MHz)

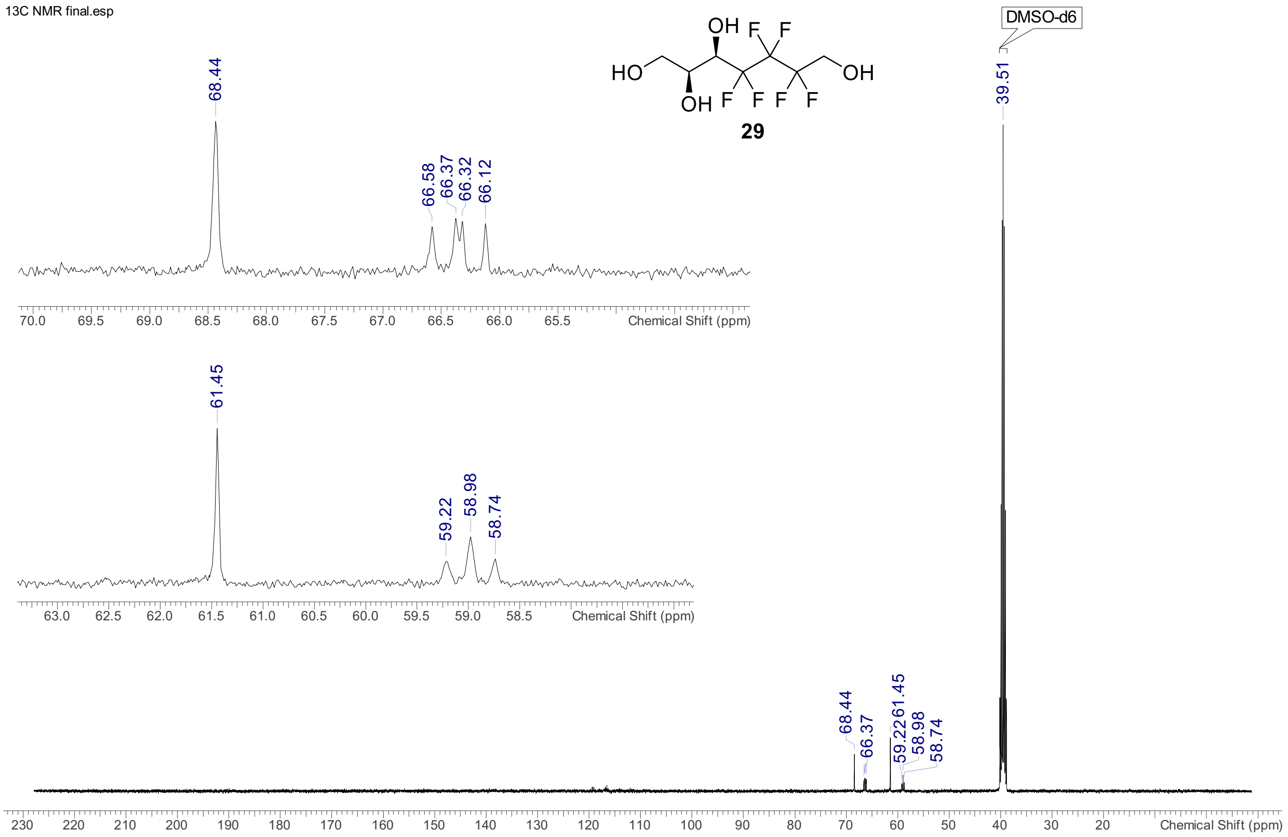

6.11 NMR spectra of (2*S*,3*R*)-4,4,5,5,6,6-hexafluoro-1,2-*O*-isopropylideneheptane-3,7-diol (16)

a) ^{1}H NMR (DMSO-*d*^{6}, 400 MHz)

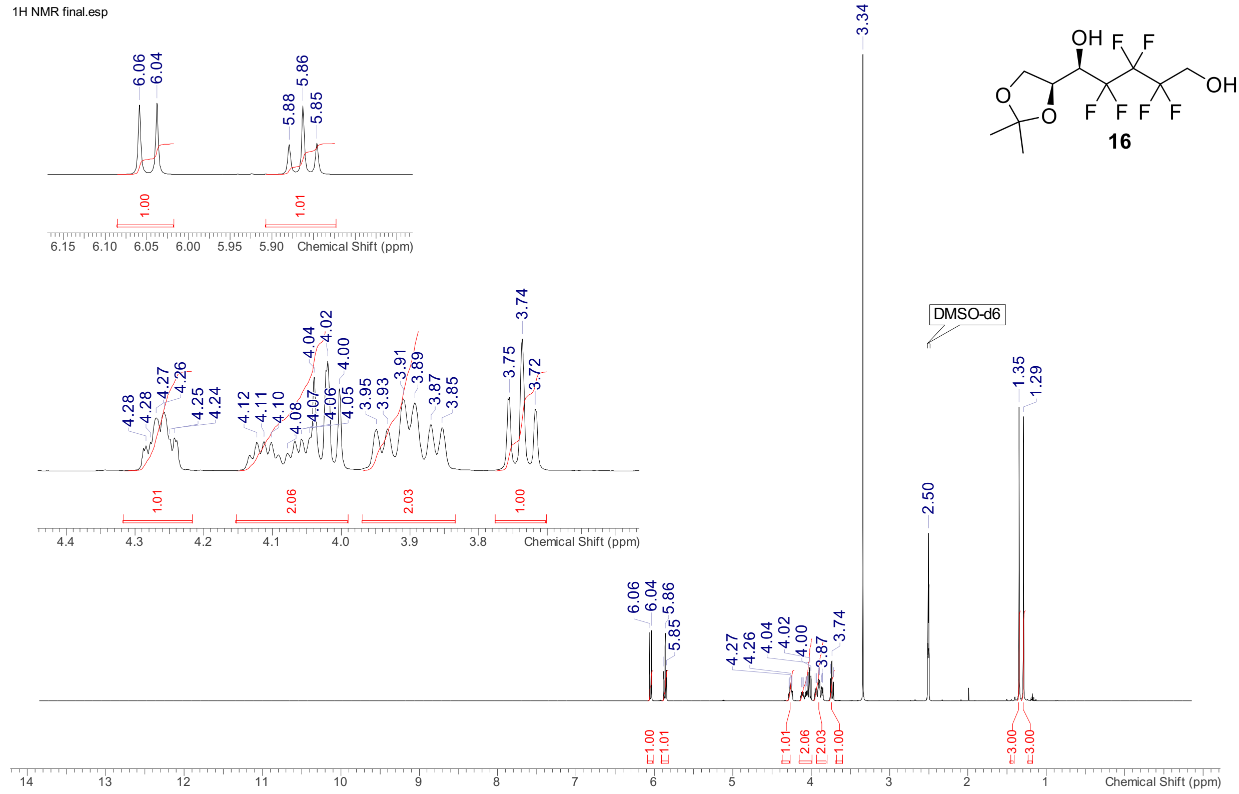

b) ^{1}H {^{19}F} NMR (DMSO-*d*^{6}, 500 MHz)

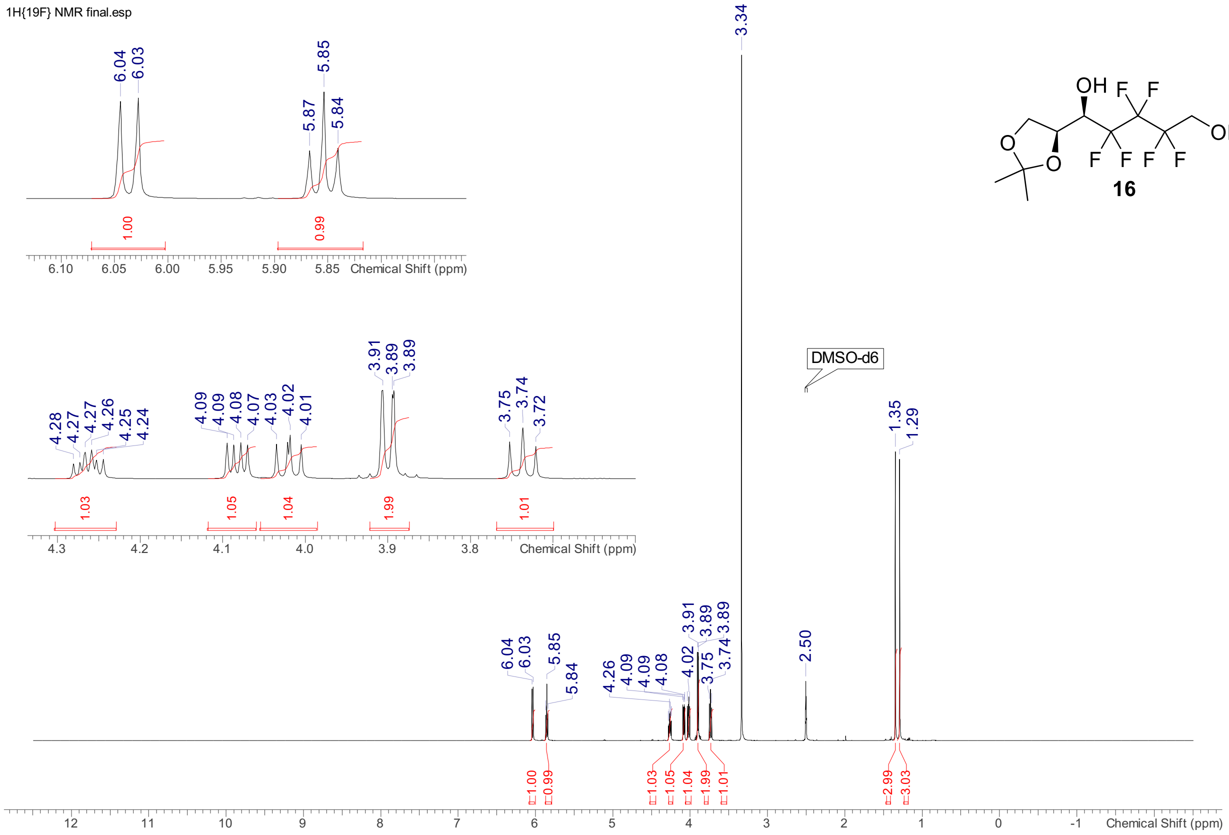

c) ^{19}F NMR (DMSO-d^6, 376 MHz)

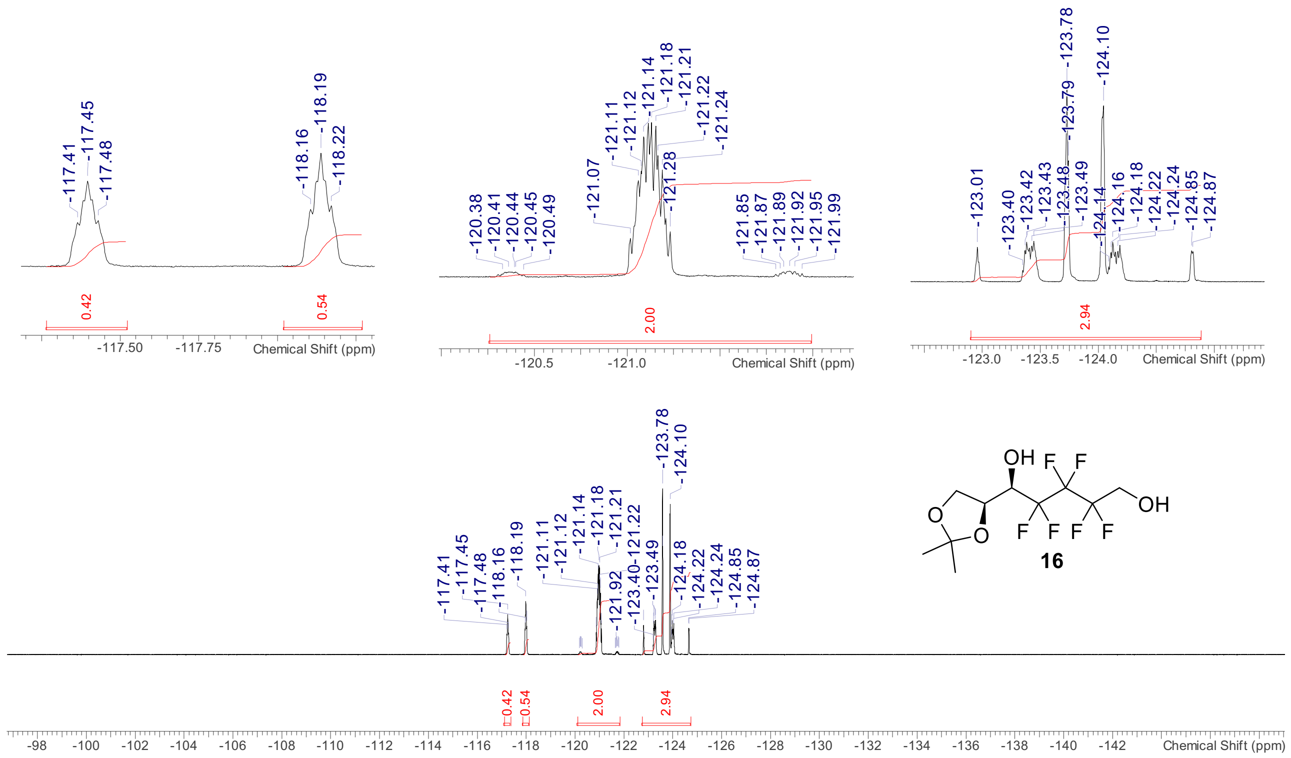

d) ^{19}F{^{1}H} NMR (DMSO-d^6, 471 MHz)

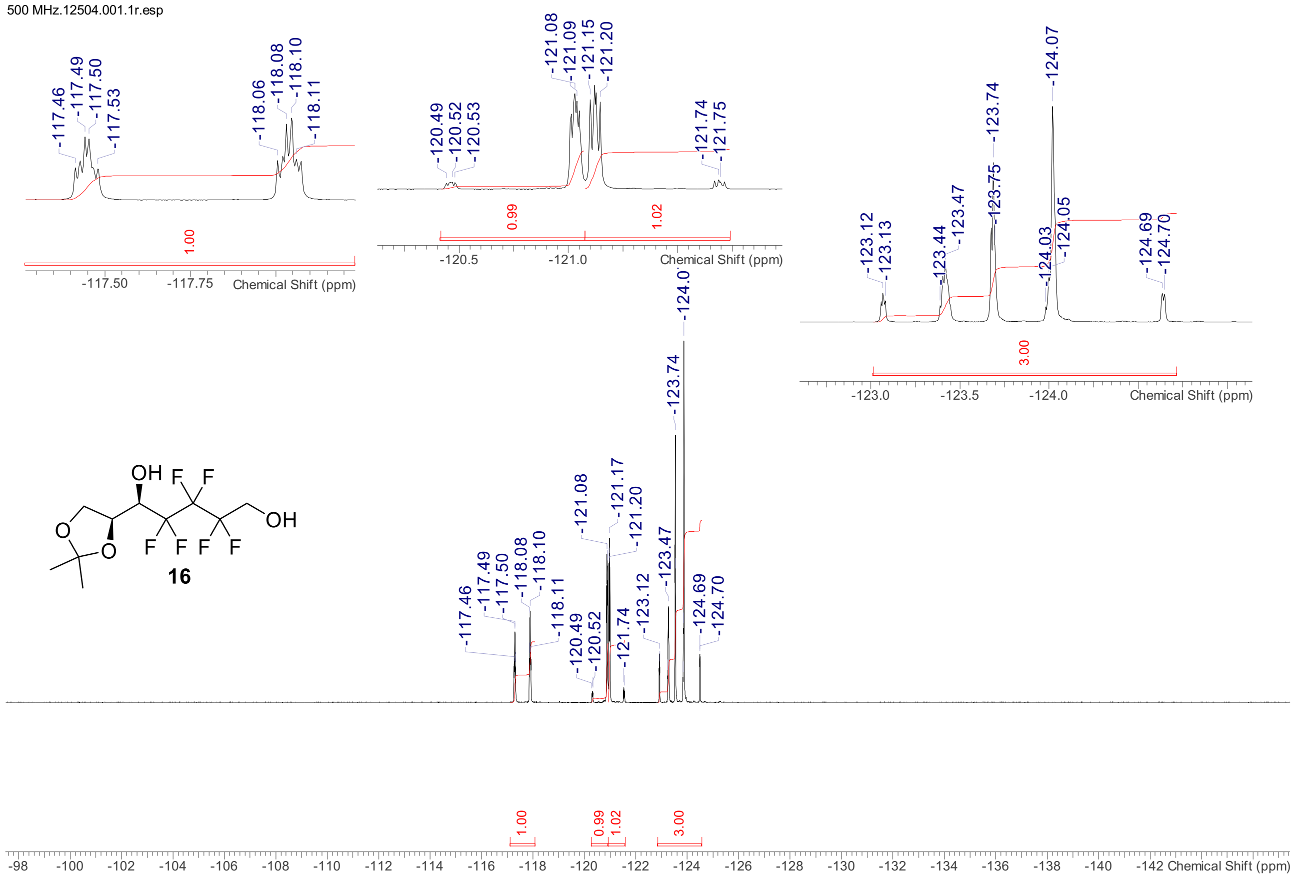

e) $^{13}C\{^{1}H\}$ NMR (DMSO-d^6, 101 MHz)

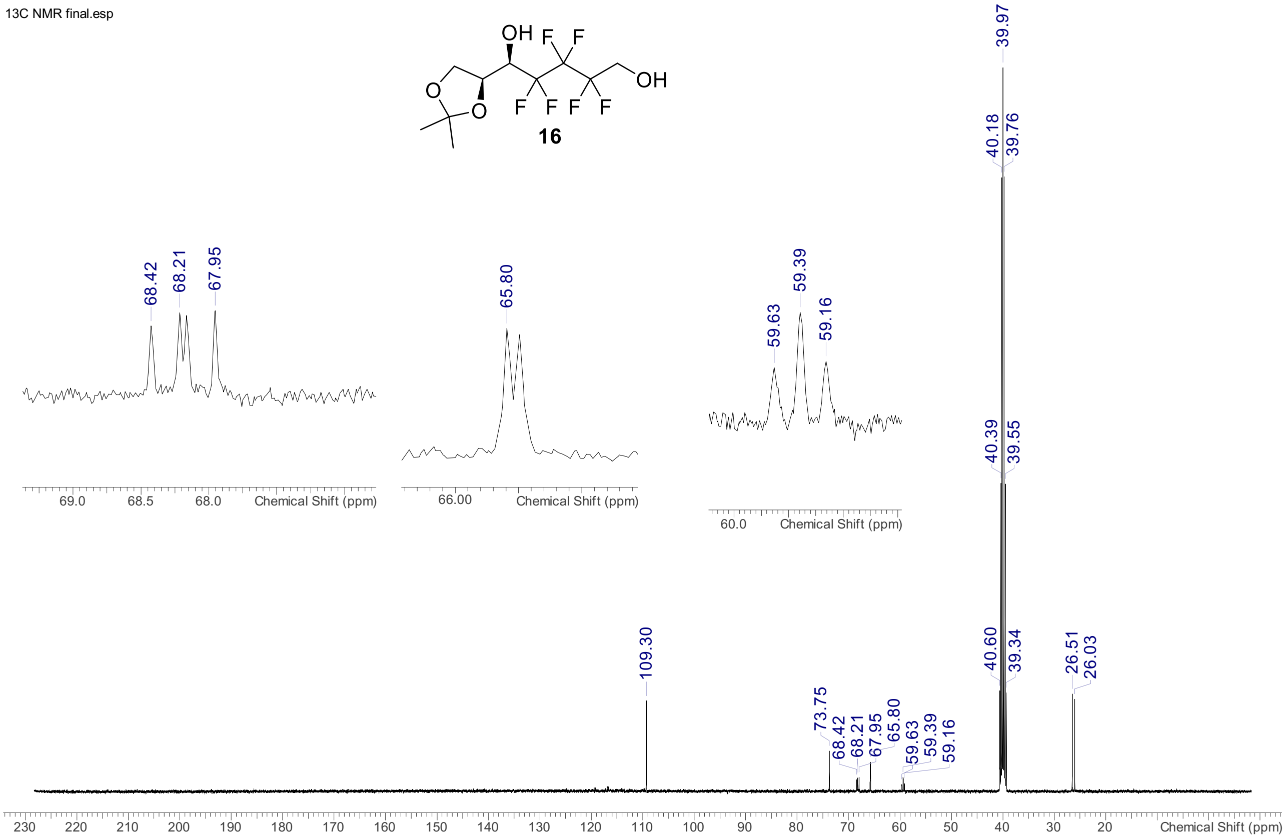

6.12 NMR spectra of 2,3,4-trideoxy-6,7-*O*-isopropylidene-2,2,3,3,4,4-hexafluoro-L-*threo*-heptopyranose (L-31)

a) ^{1}H NMR (Acetone-d^{6}, 400 MHz)

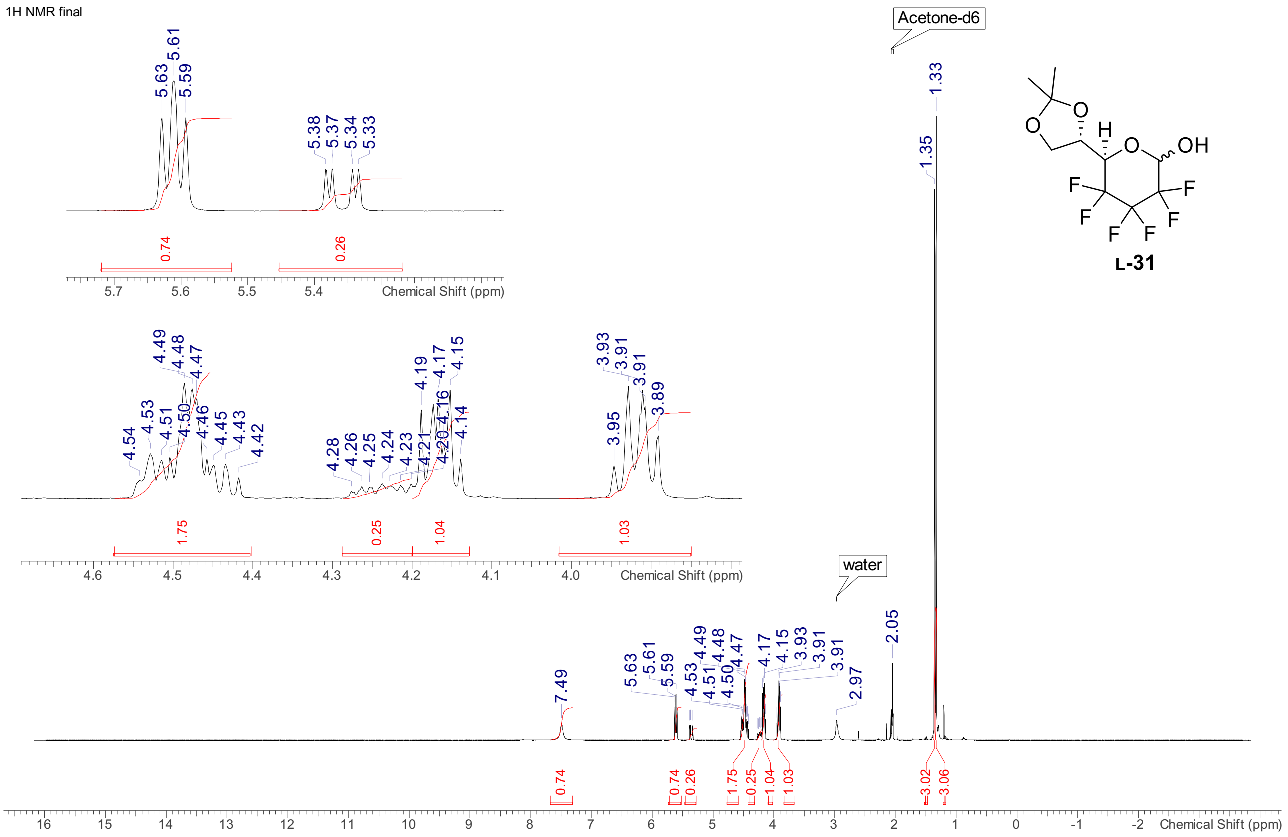

b) ^{1}H{^{19}F} NMR (Acetone -d^{6}, 500 MHz)

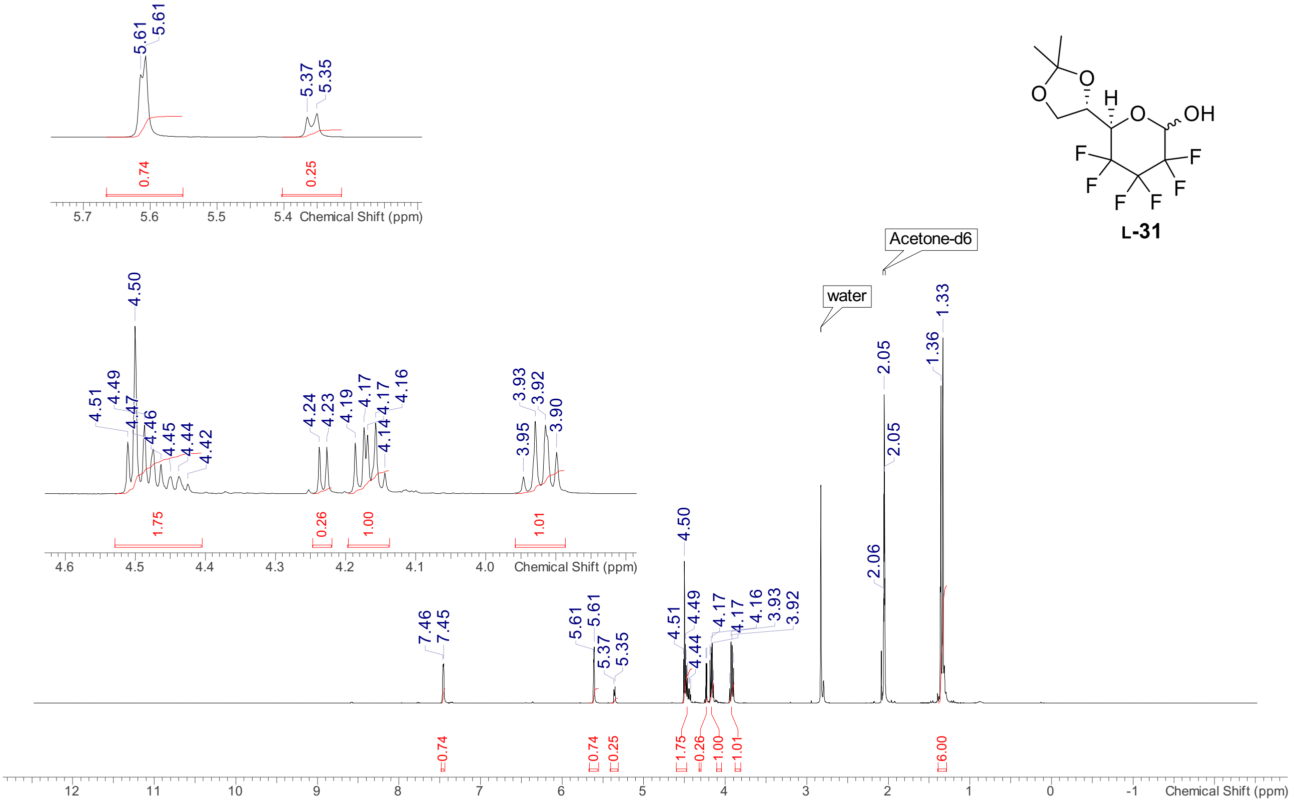

c) ^{19}F NMR (Acetone -d^6, 376 MHz)

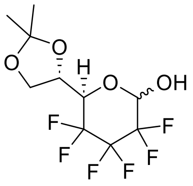

L-31

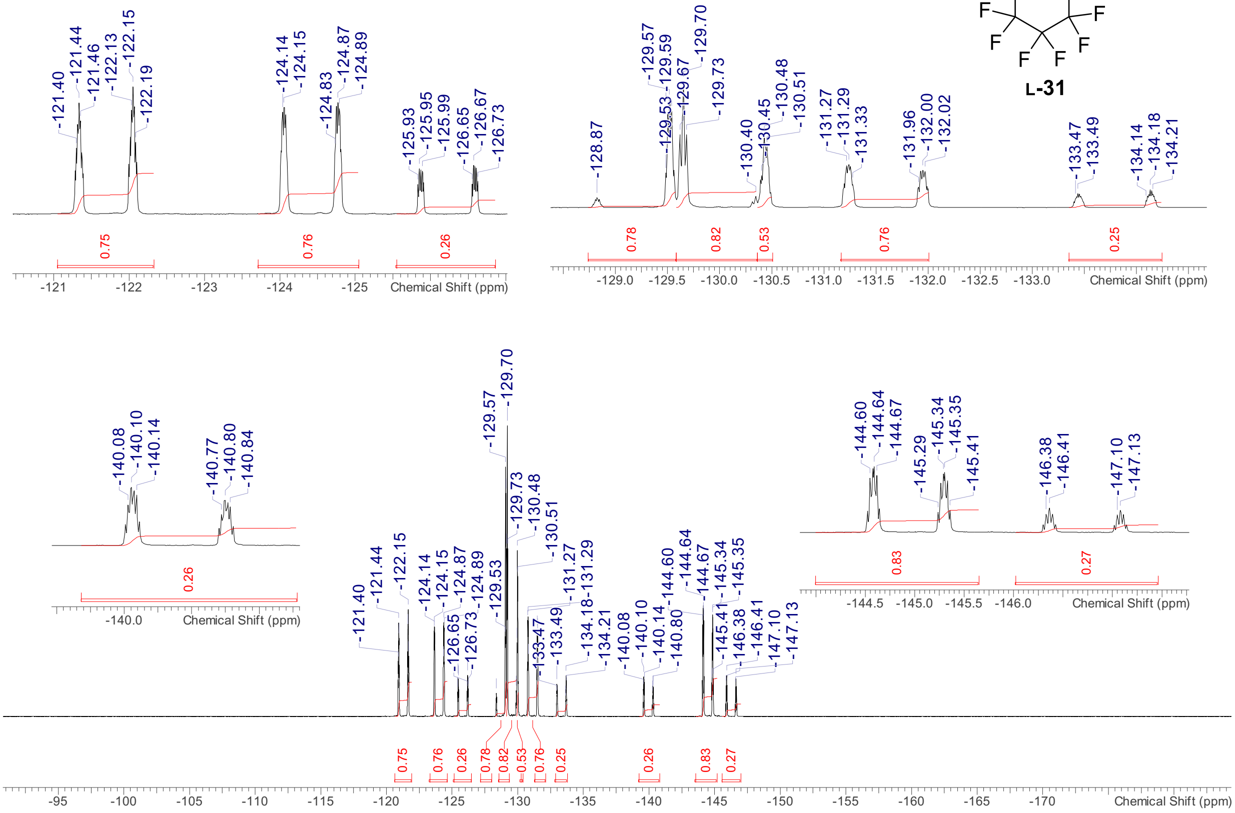

d) ^{19}F{^{1}H} NMR (Acetone -d^6, 471 MHz)

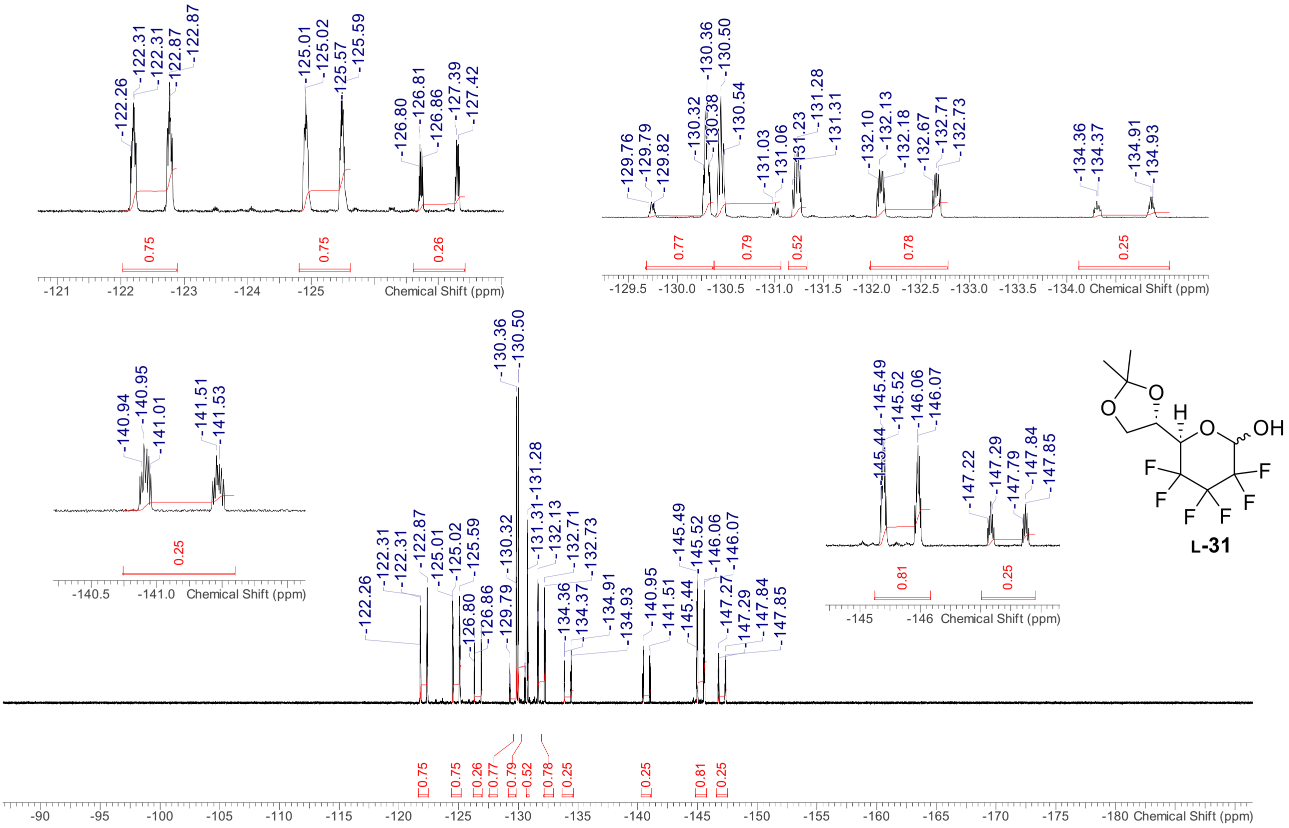

L-31

e) $^{13}C\{^{1}H\}$ NMR (Acetone -d^6, 101 MHz)

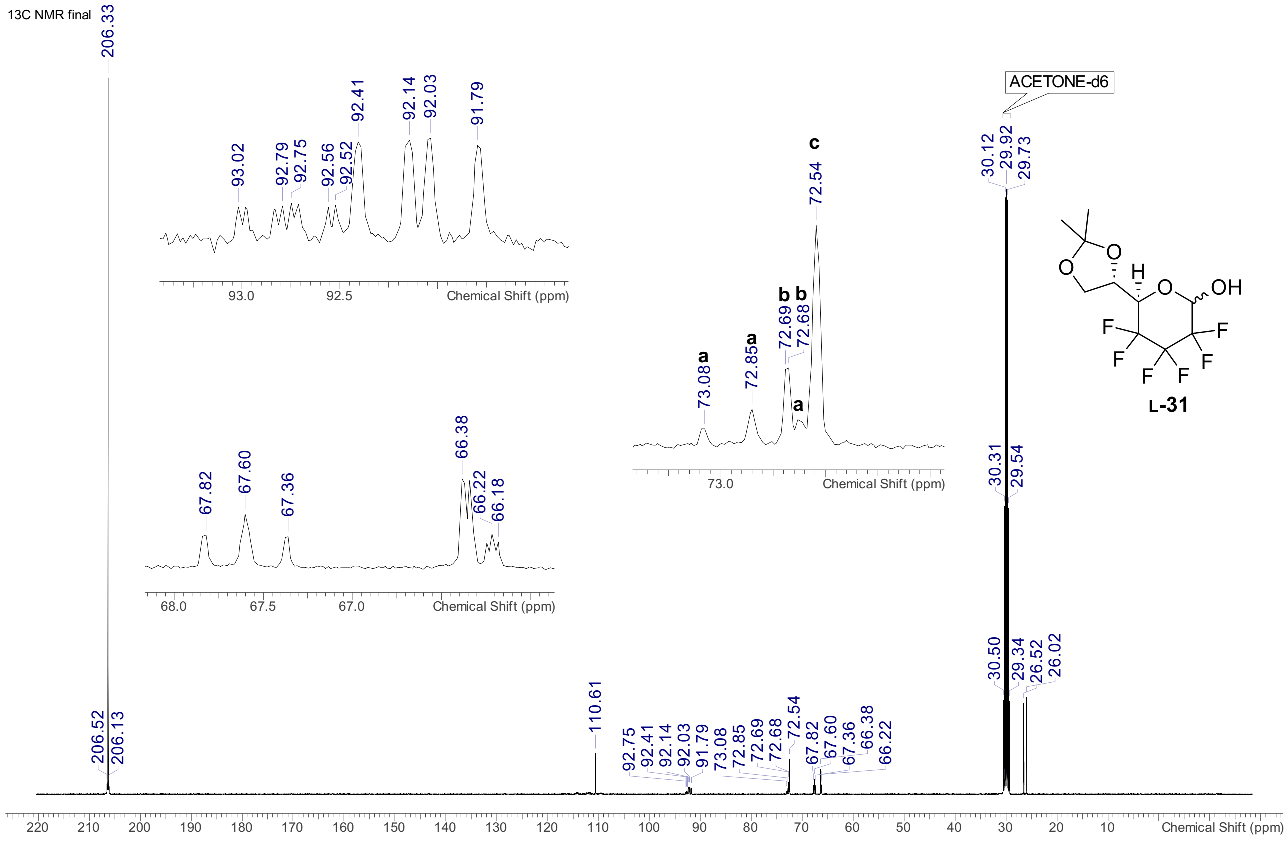

6.13 NMR spectra of 4,5,6-trideoxy-1,2-*O*-isopropylidene-4,4,5,5,6,6-hexafluoro-D-*glycero*-hept-3-ulopyranose (D-32)

a) ^{1}H NMR (CDCl$_3$, 400 MHz)

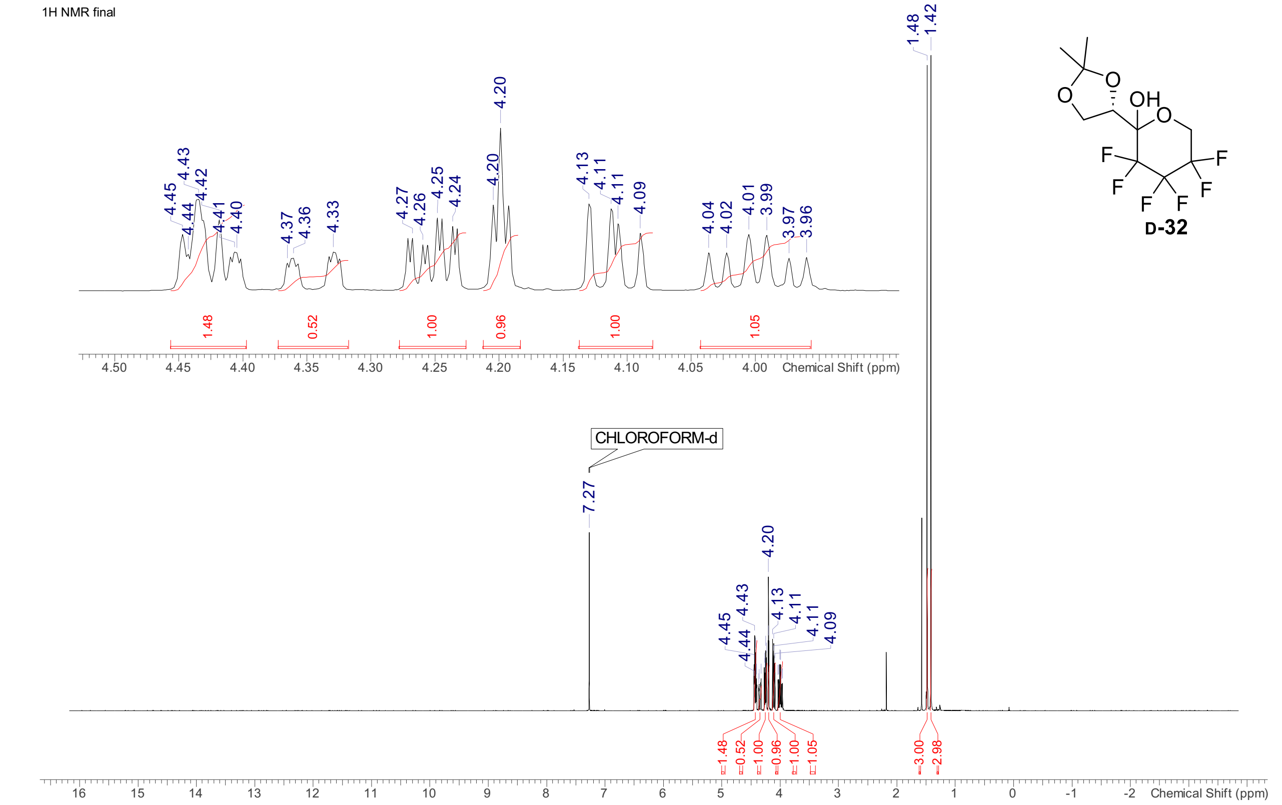

b) ^{19}F NMR (CDCl$_3$, 376 MHz)

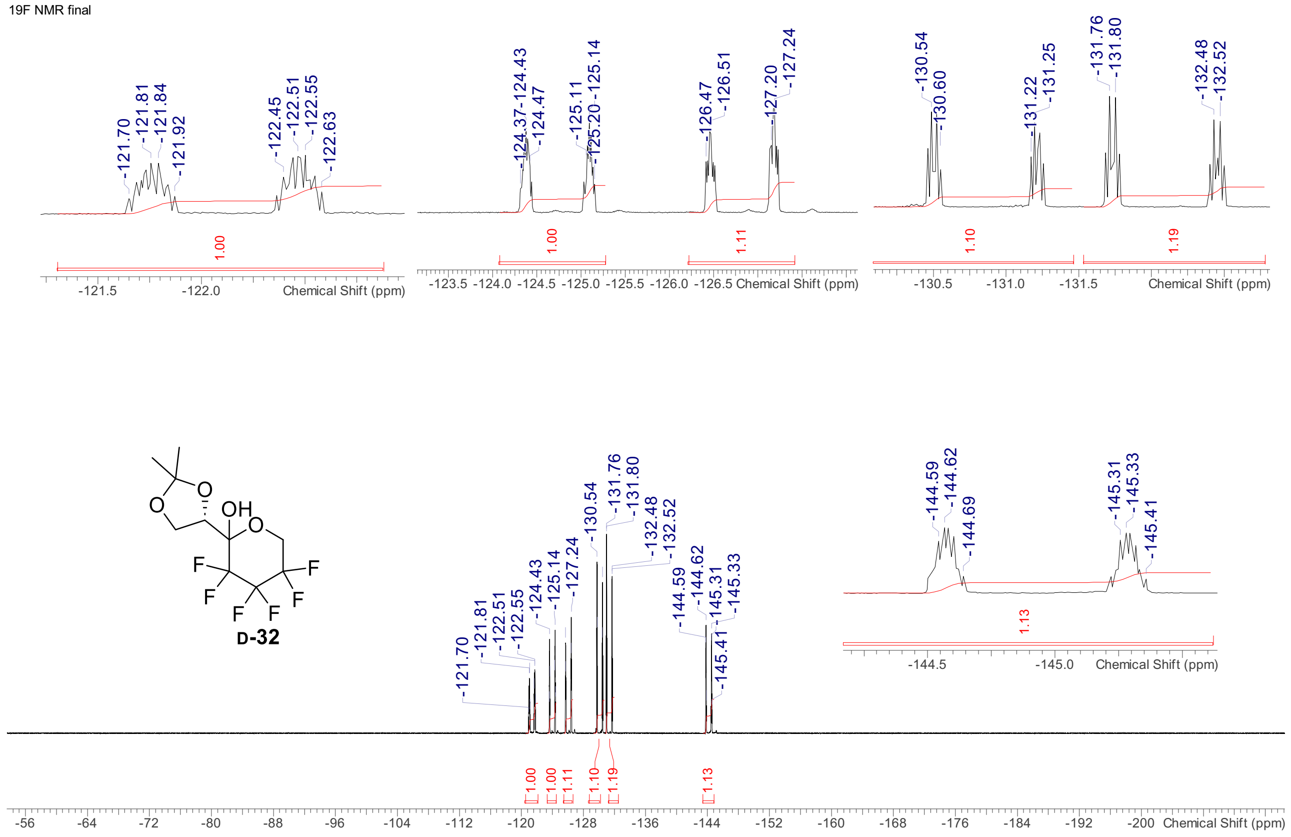

c) $^{13}C\{^1H\}$ NMR ($CDCl_3$, 101 MHz)

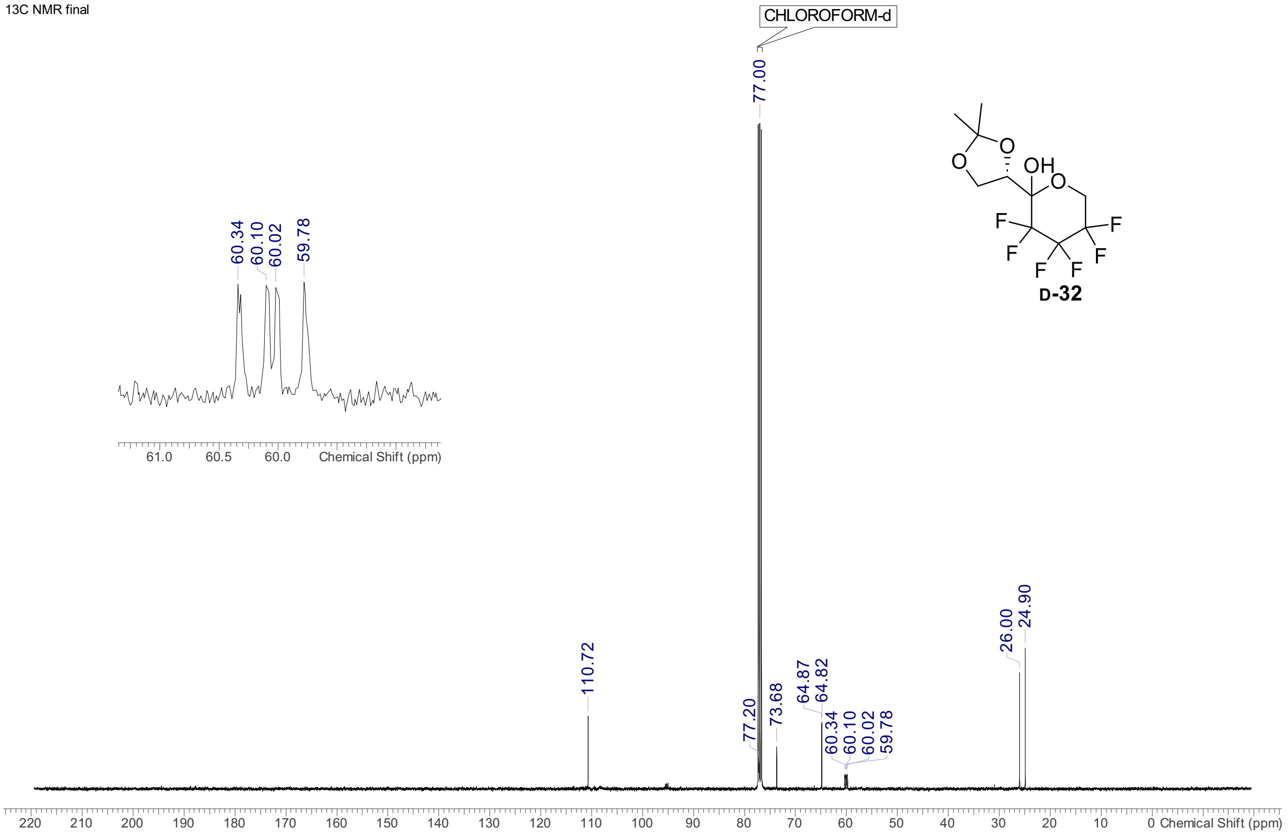

6.14 NMR spectra of benzyl-2,3,4-trideoxy-6,7-*O*-isopropylidene-2,2,3,3,4,4-hexafluoro-β-L-*threo*-heptopyranoside (β-L-33)

a) ^{1}H NMR ($CDCl_3$, 400 MHz)

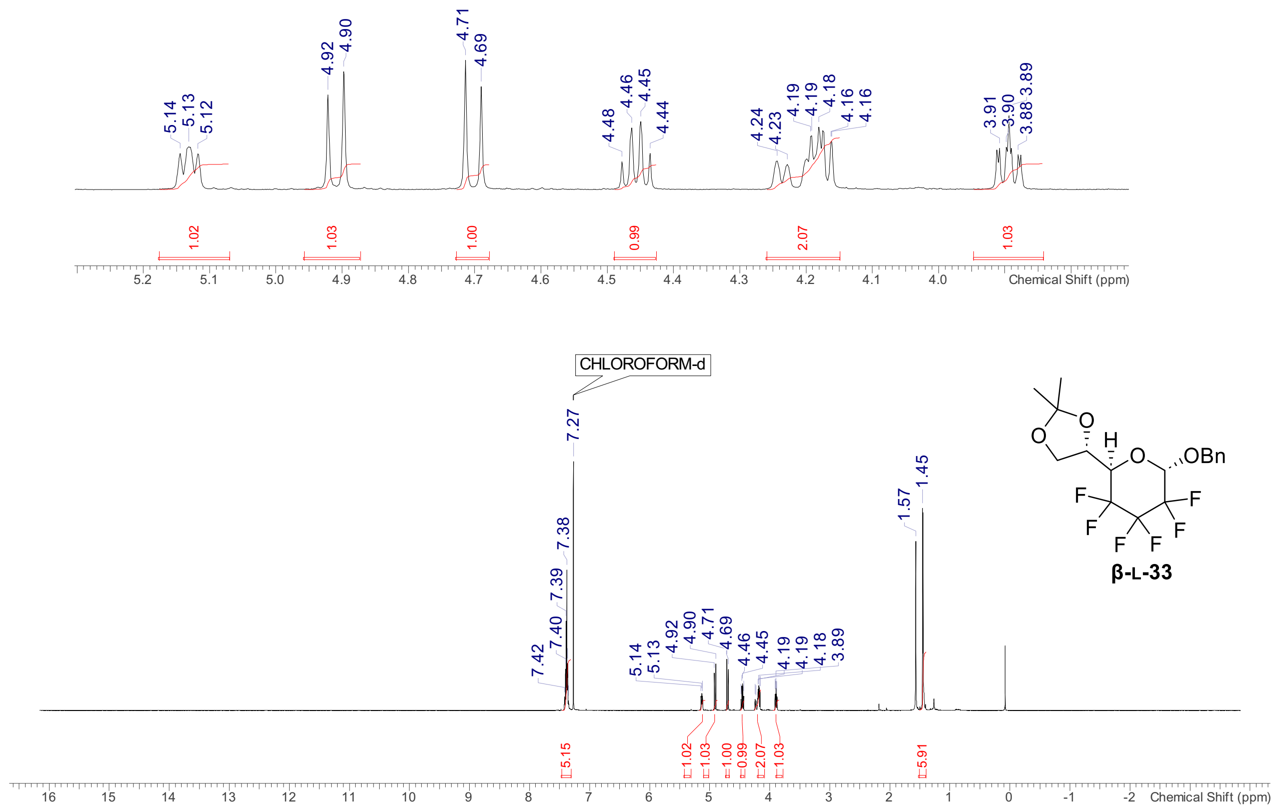

b) $^{1}H\{^{19}F\}$ NMR ($CDCl_3$, 500 MHz)

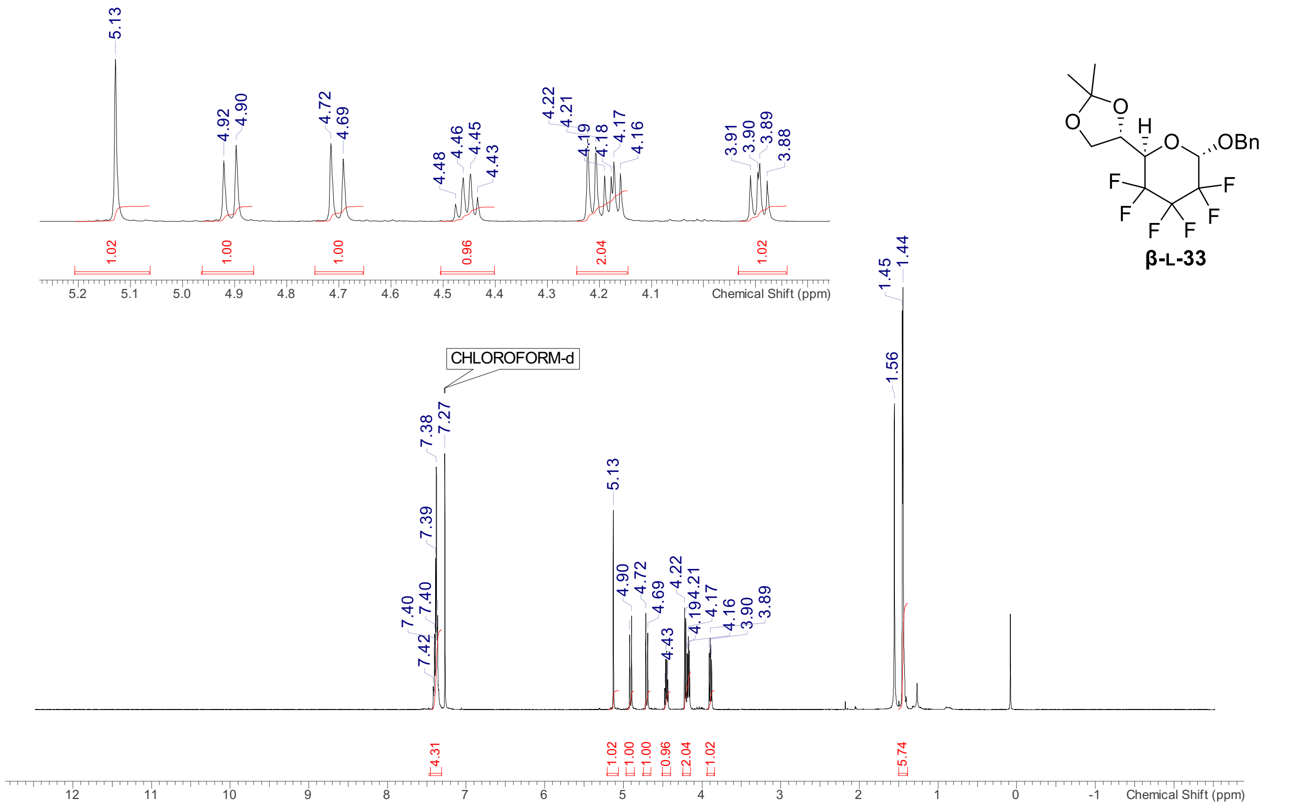

c) ^{19}F NMR ($CDCl_3$, 376 MHz)

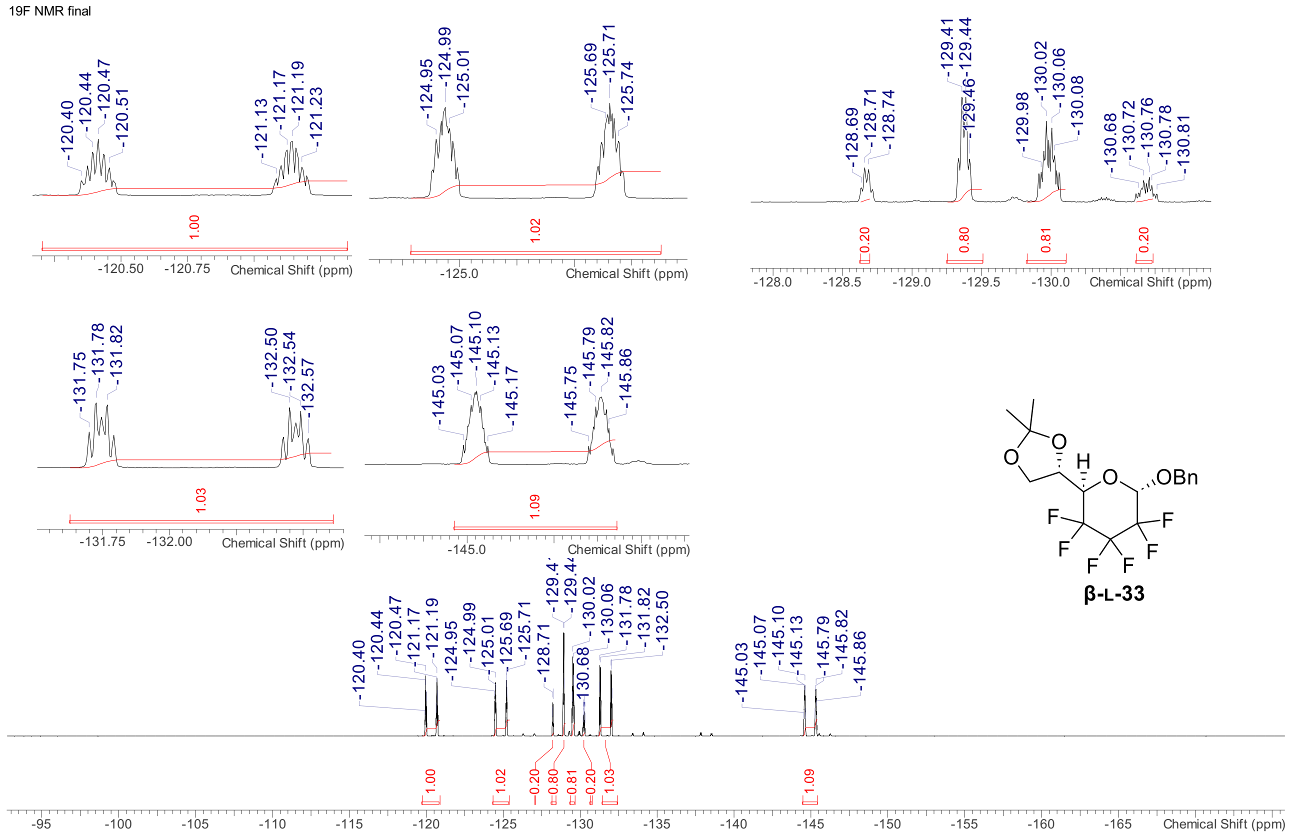

d) $^{19}F\{^1H\}$ NMR ($CDCl_3$, 471 MHz)

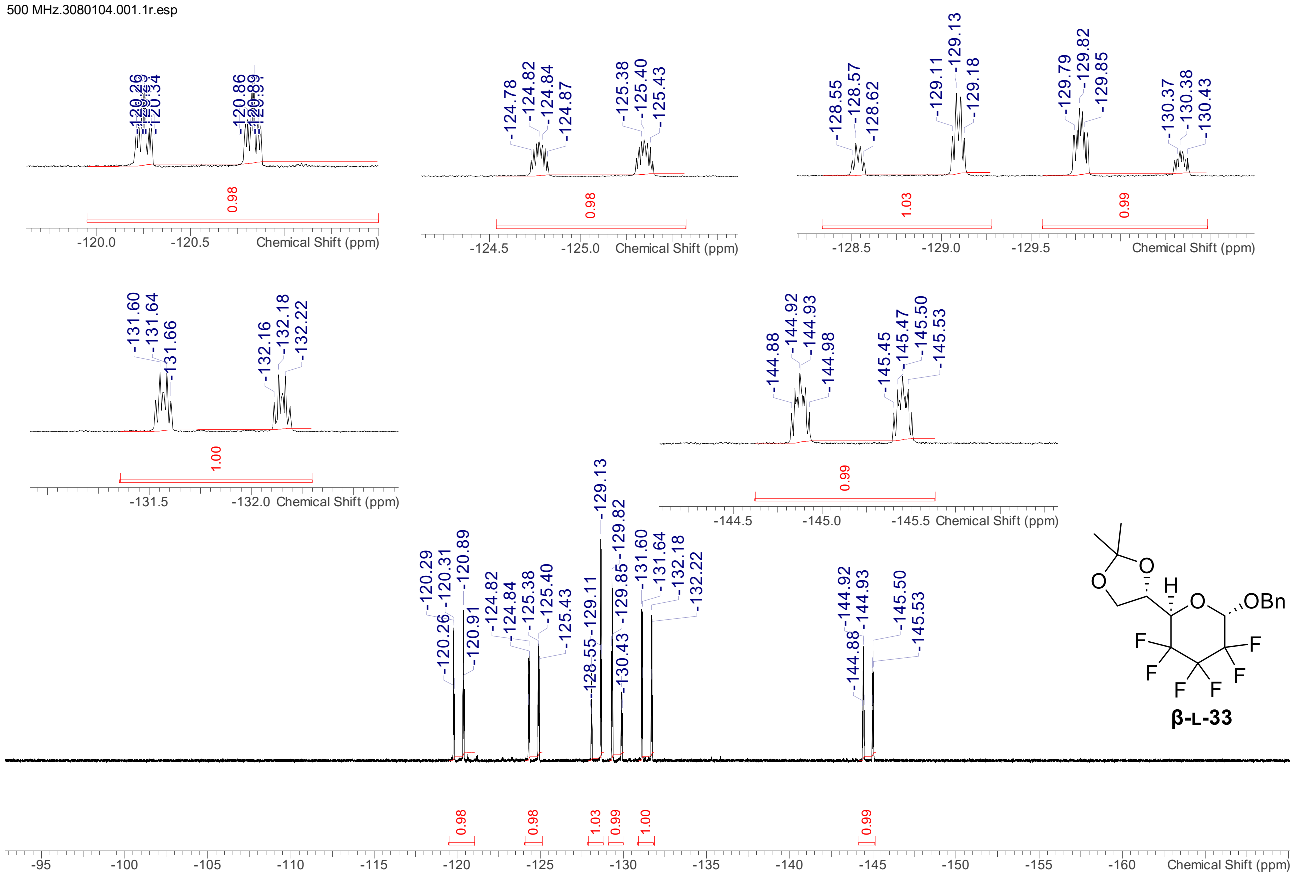

e) $^{13}C\{^{1}H\}$ NMR ($CDCl_3$, 101 MHz)

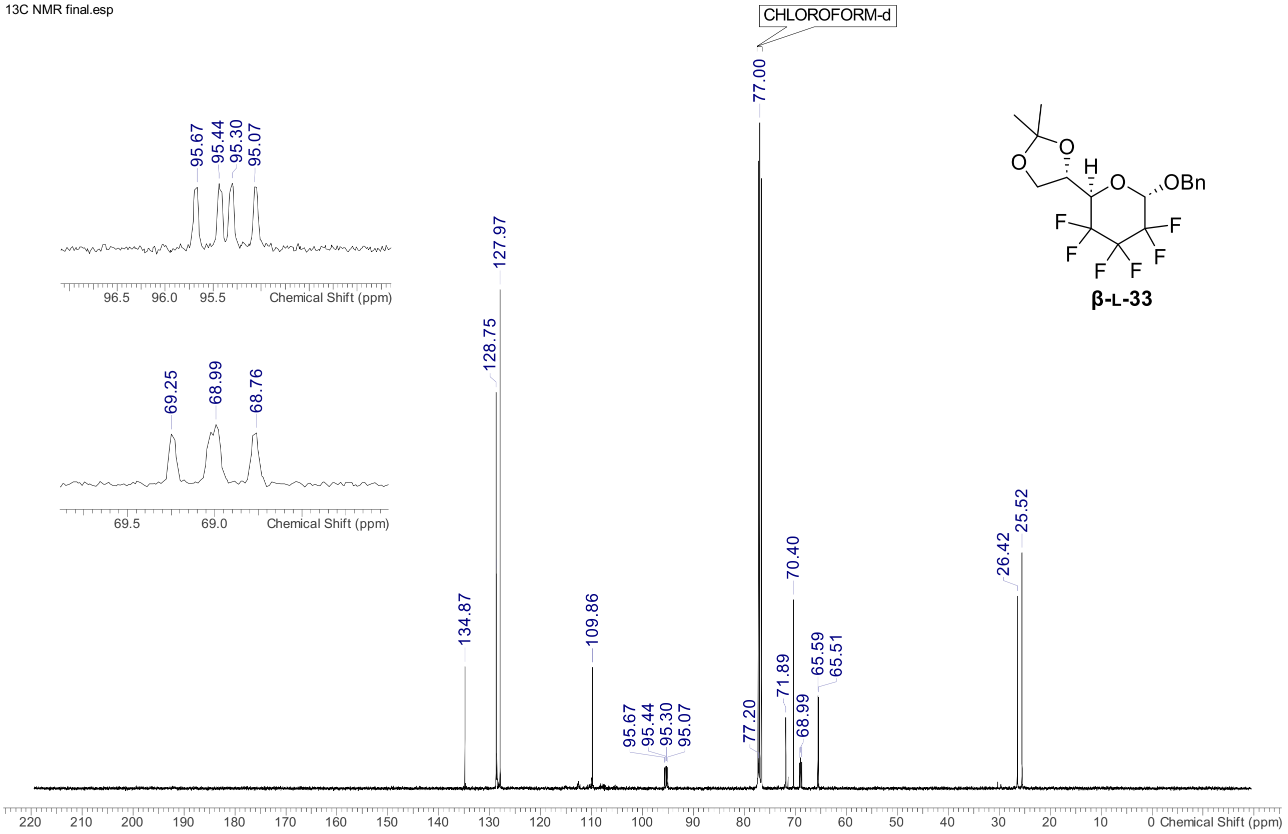

6.15 NMR spectra of benzyl-2,3,4-trideoxy-6,7-*O*-isopropylidene-2,2,3,3,4,4-hexafluoro-α-L-*threo*-heptopyranoside (α-L-33)

a) ^{1}H NMR ($CDCl_3$, 400 MHz)

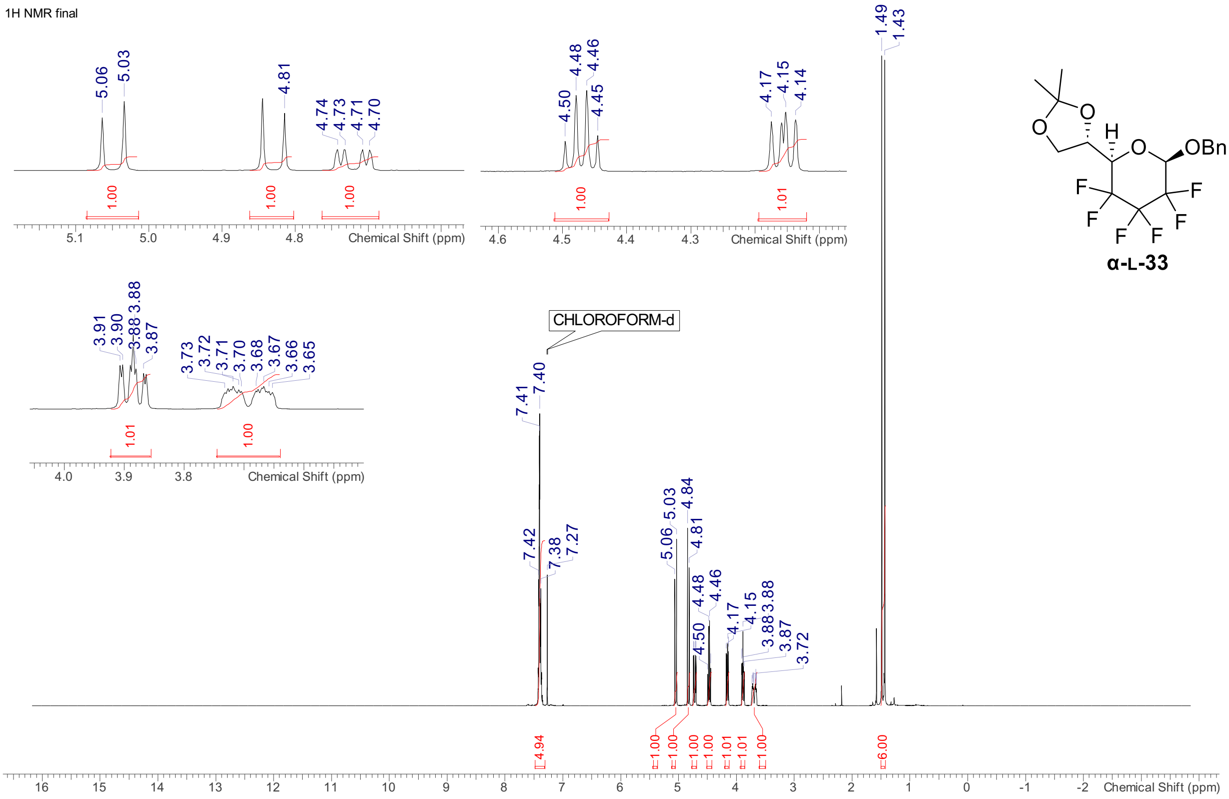

b) $^{1}H\{^{19}F\}$ NMR ($CDCl_3$, 500 MHz)

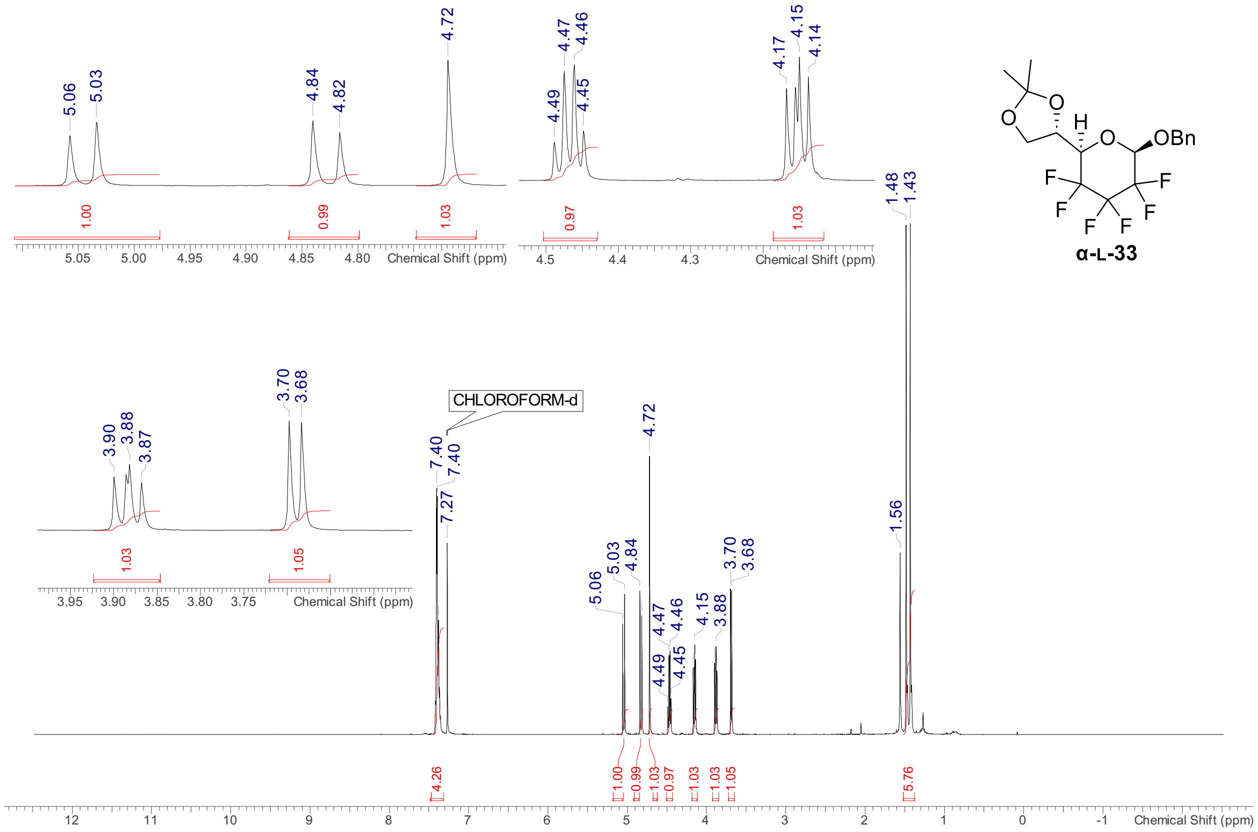

c) ^{19}F NMR ($CDCl_3$, 376 MHz)

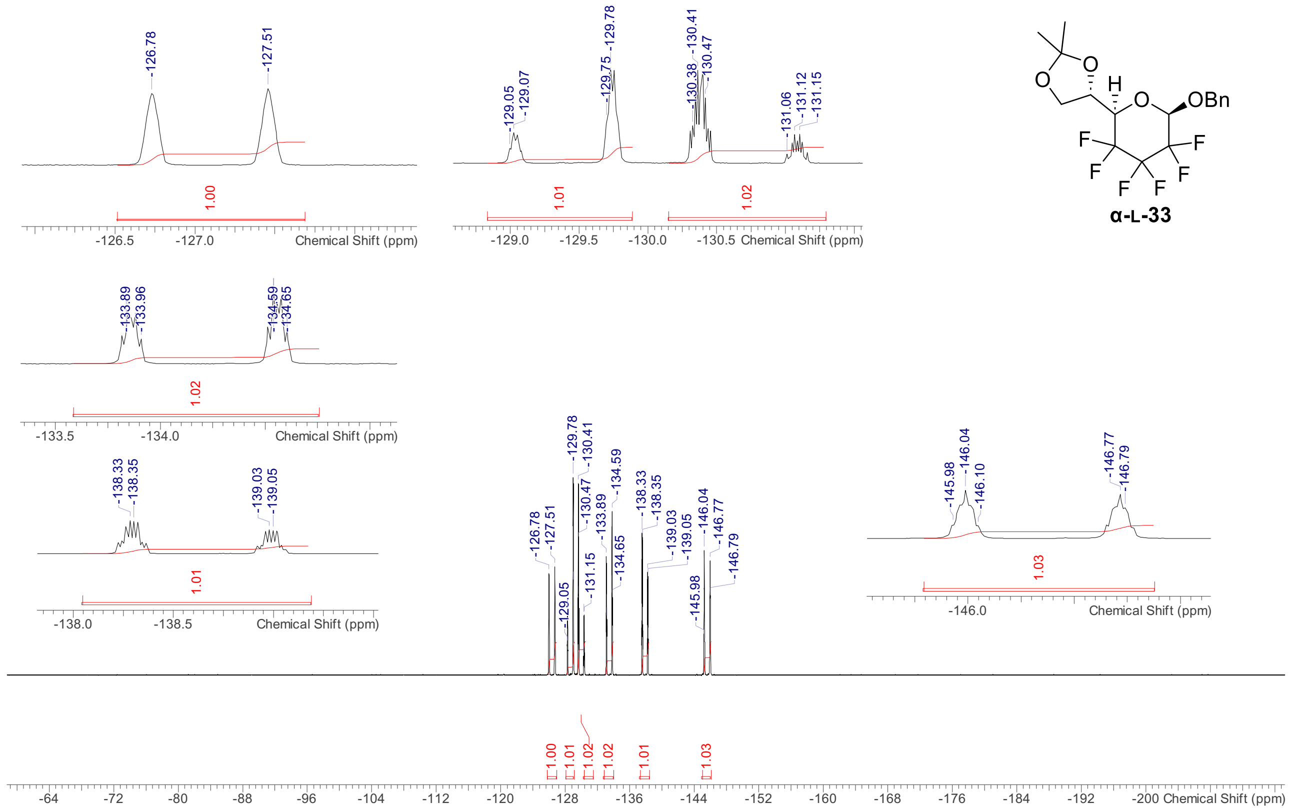

d) $^{19}F\{^1H\}$ NMR ($CDCl_3$, 471 MHz)

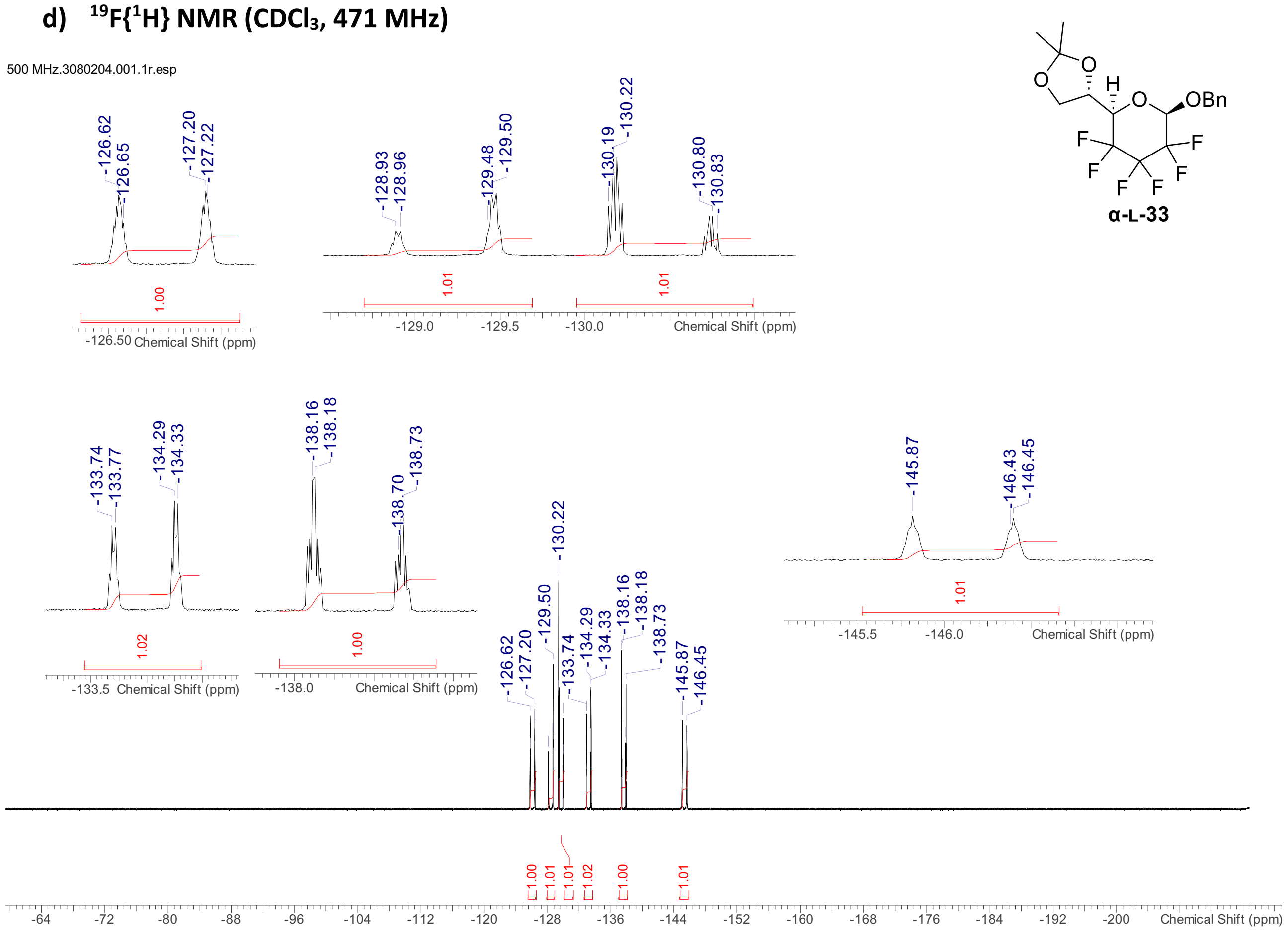

e) $^{13}C\{^{1}H\}$ NMR ($CDCl_3$, 101 MHz)

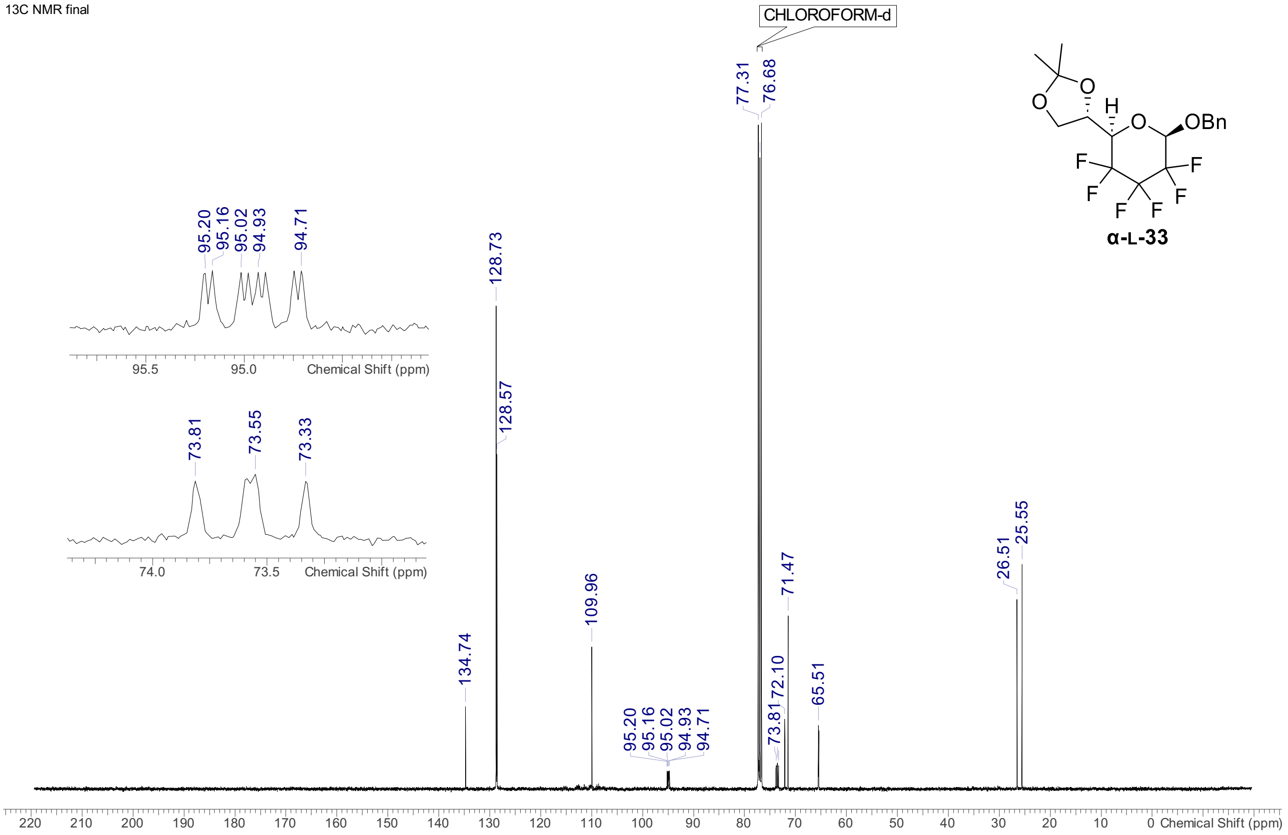

6.16 NMR spectra of benzyl-2,3,4-trideoxy-2,2,3,3,4,4-hexafluoro-β-L-*threo*-heptopyranoside (β-L-34)

a) ^{1}H NMR ($CDCl_3$, 400 MHz)

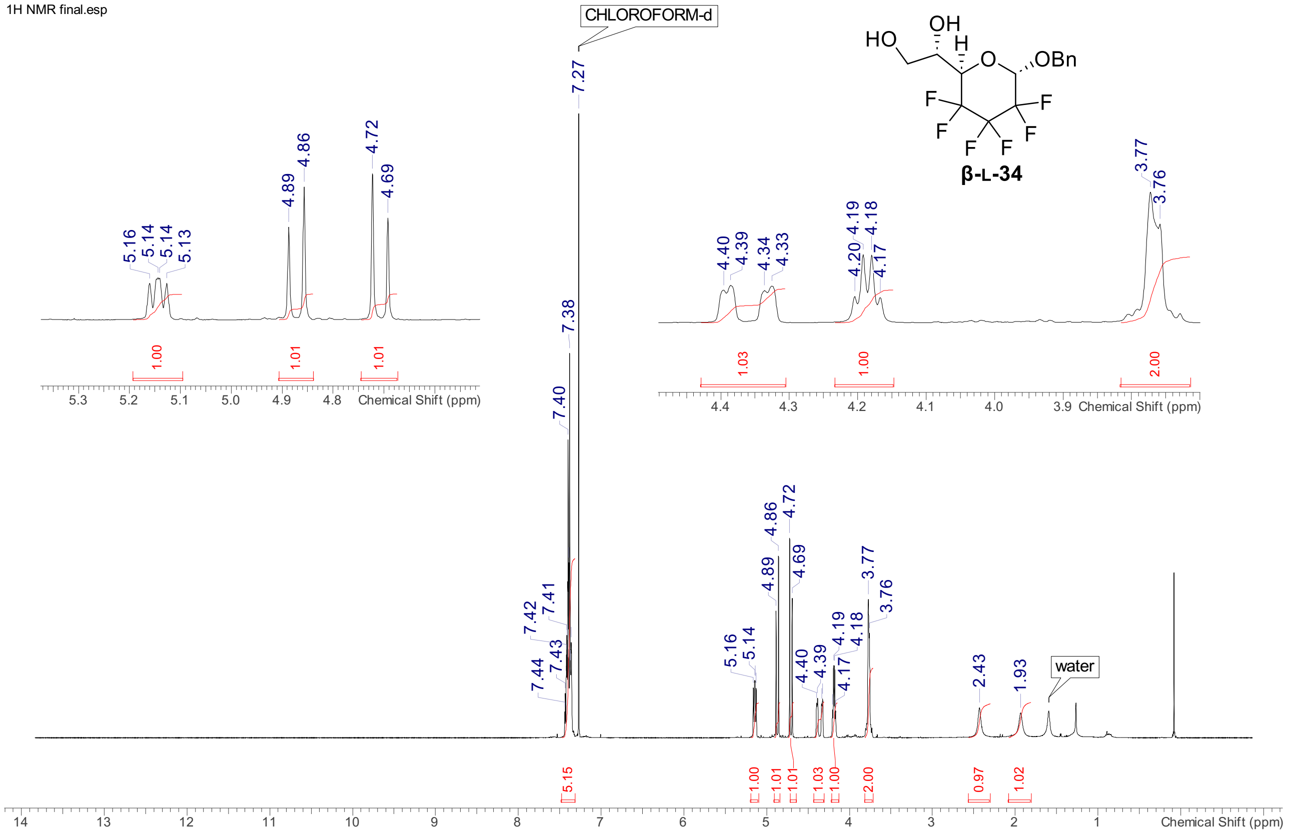

b) $^{1}H\{^{19}F\}$ NMR ($CDCl_3$, 500 MHz)

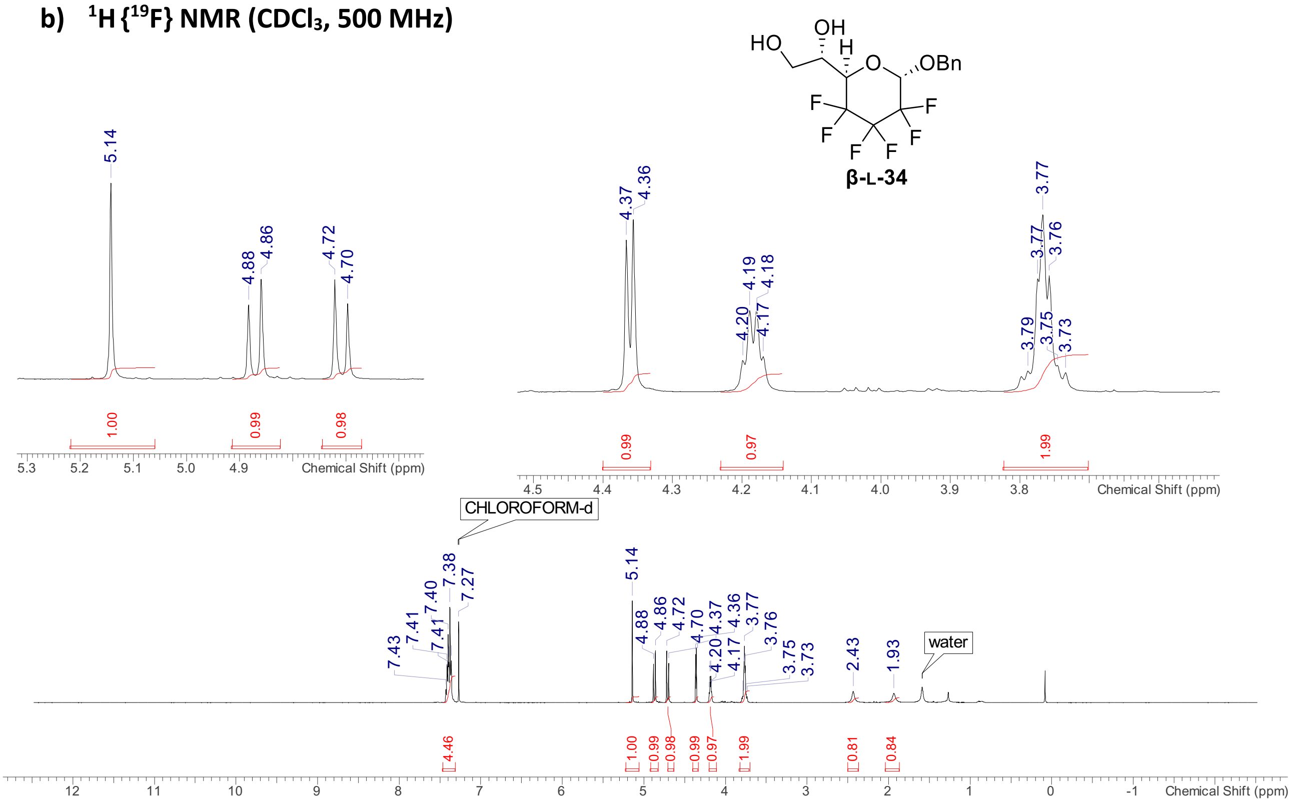

c) ^{19}F NMR ($CDCl_3$, 376 MHz)

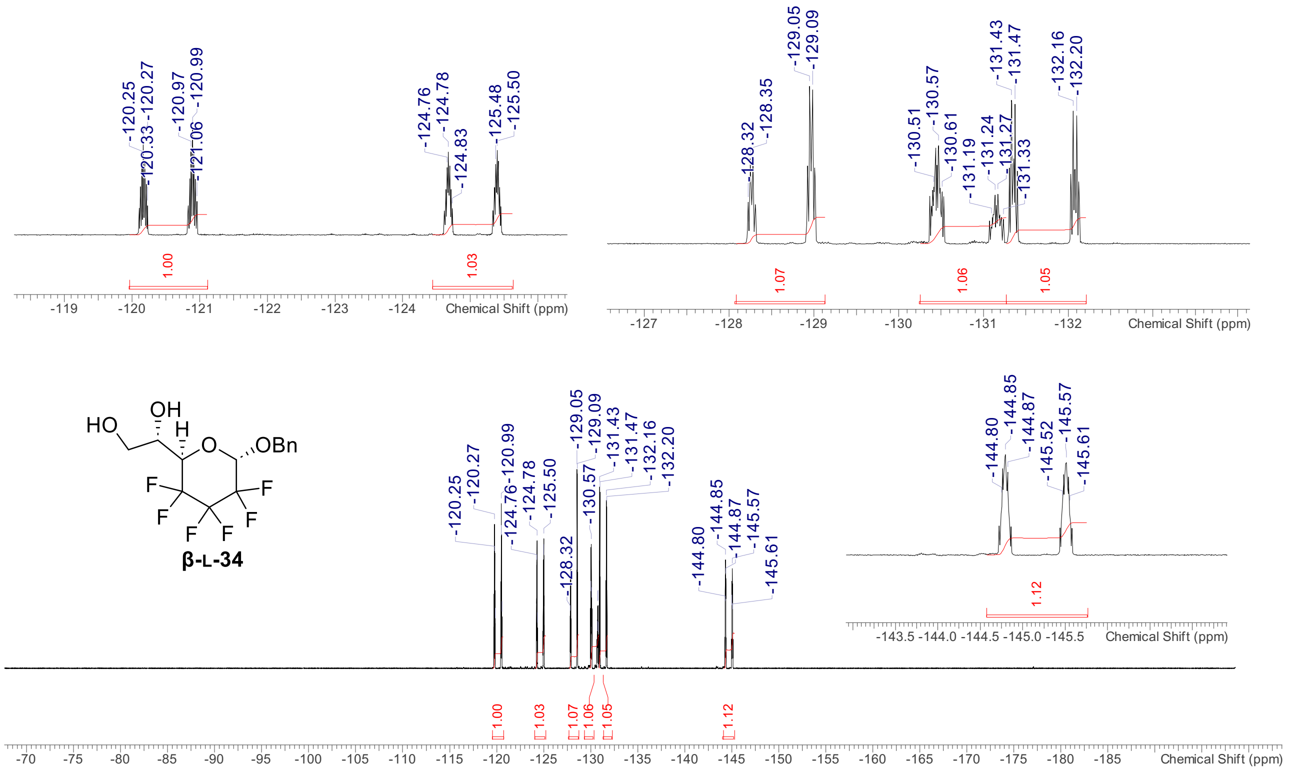

d) ^{19}F{^{1}H} NMR ($CDCl_3$, 471 MHz)

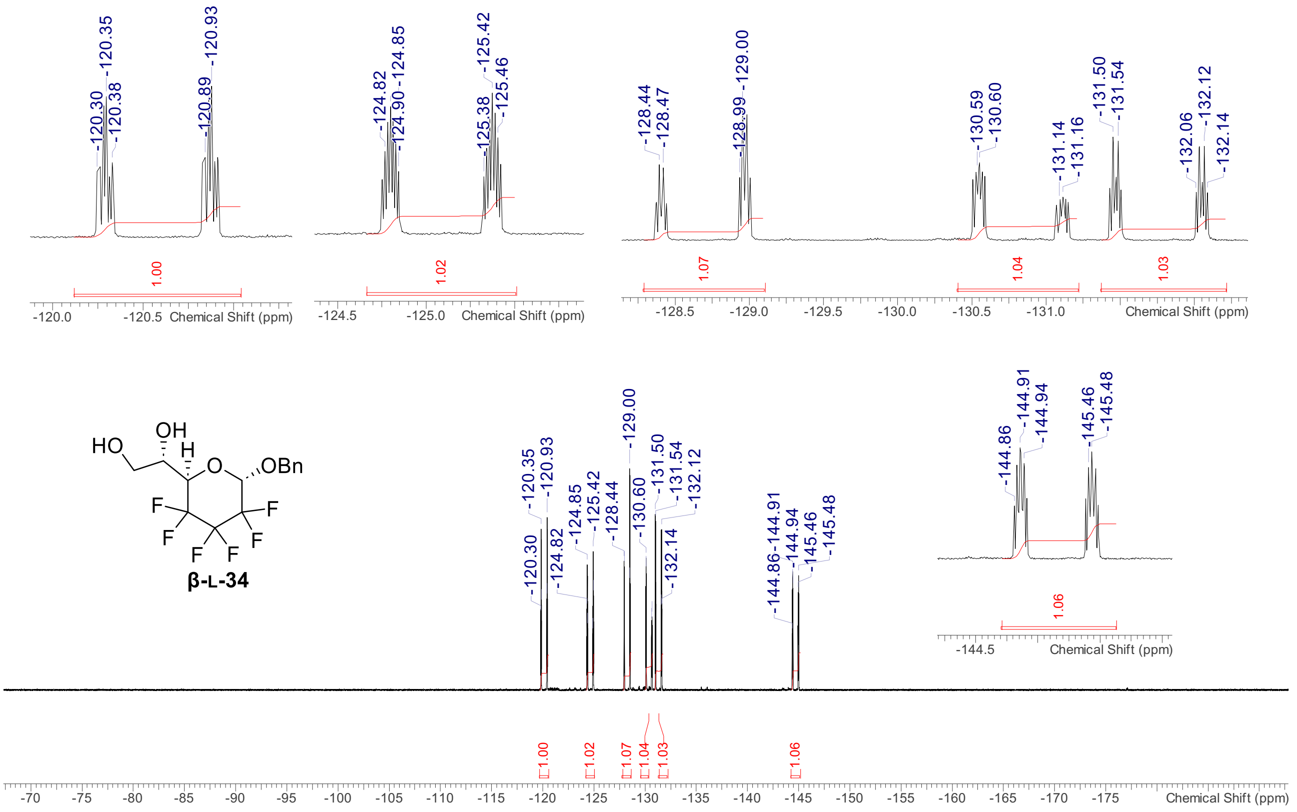

e) $^{13}C\{^1H\}$ NMR ($CDCl_3$, 101 MHz)

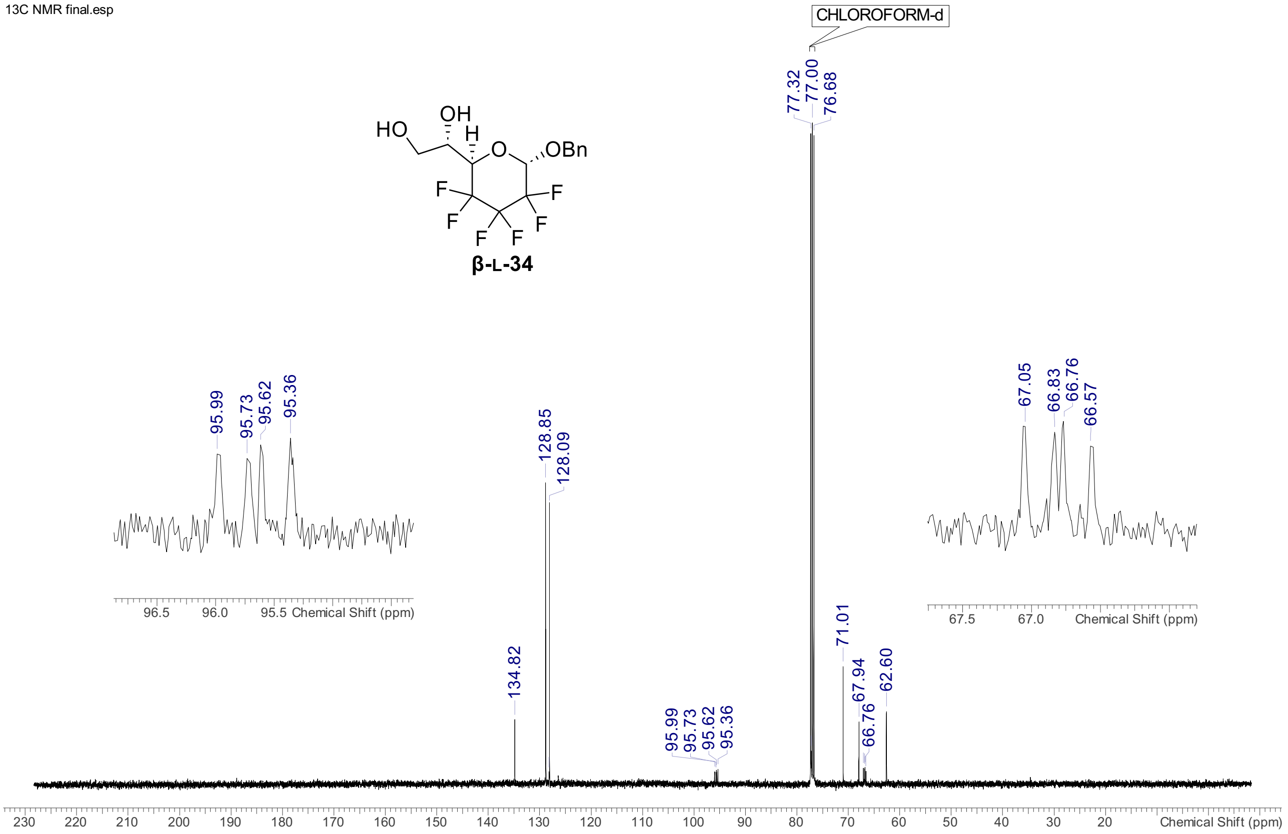

6.17 NMR spectra of benzyl 2,3,4-trideoxy-2,2,3,3,4,4-hexafluoro-α-D-*glycero*-hexopyranoside (α-D-35)

a) ^{1}H NMR (Acetone-d^{6}, 400 MHz)

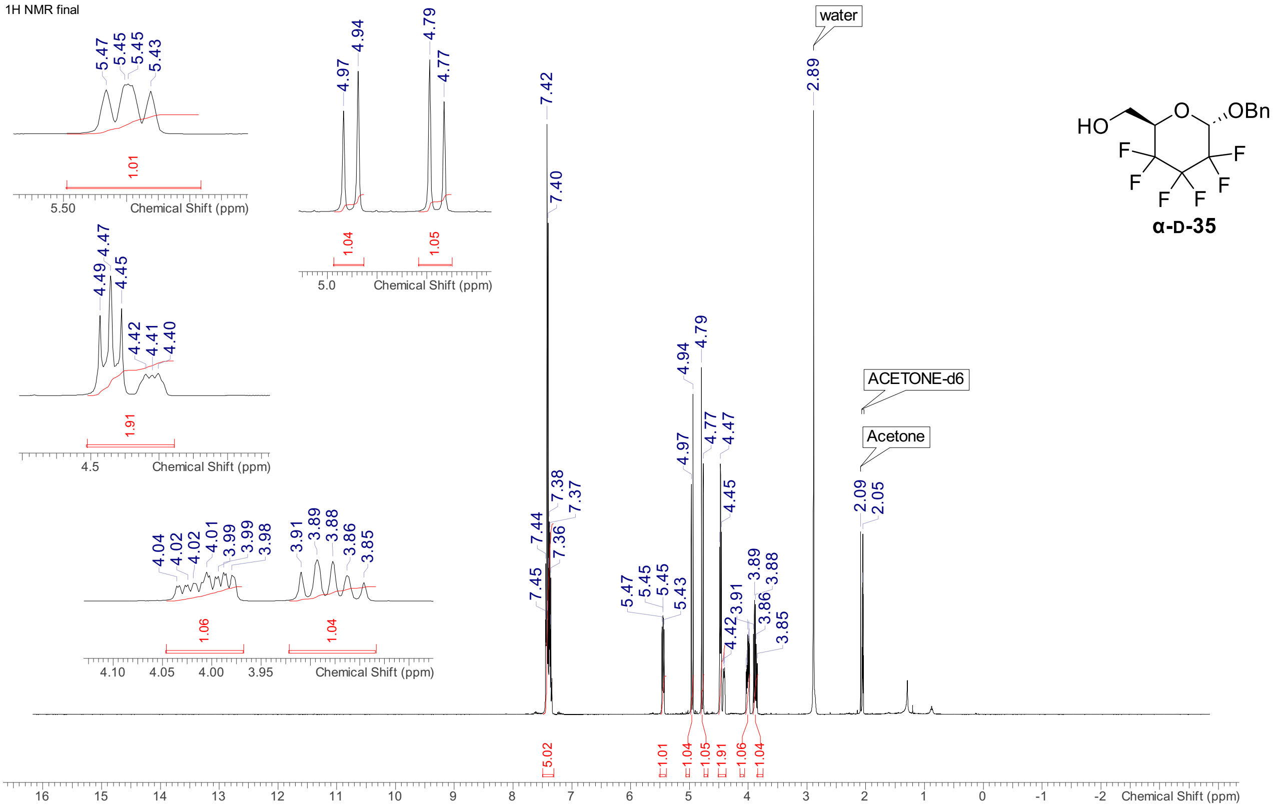

α-D-35

b) ^{1}H {^{19}F} NMR (Acetone-d^{6}, 500 MHz)

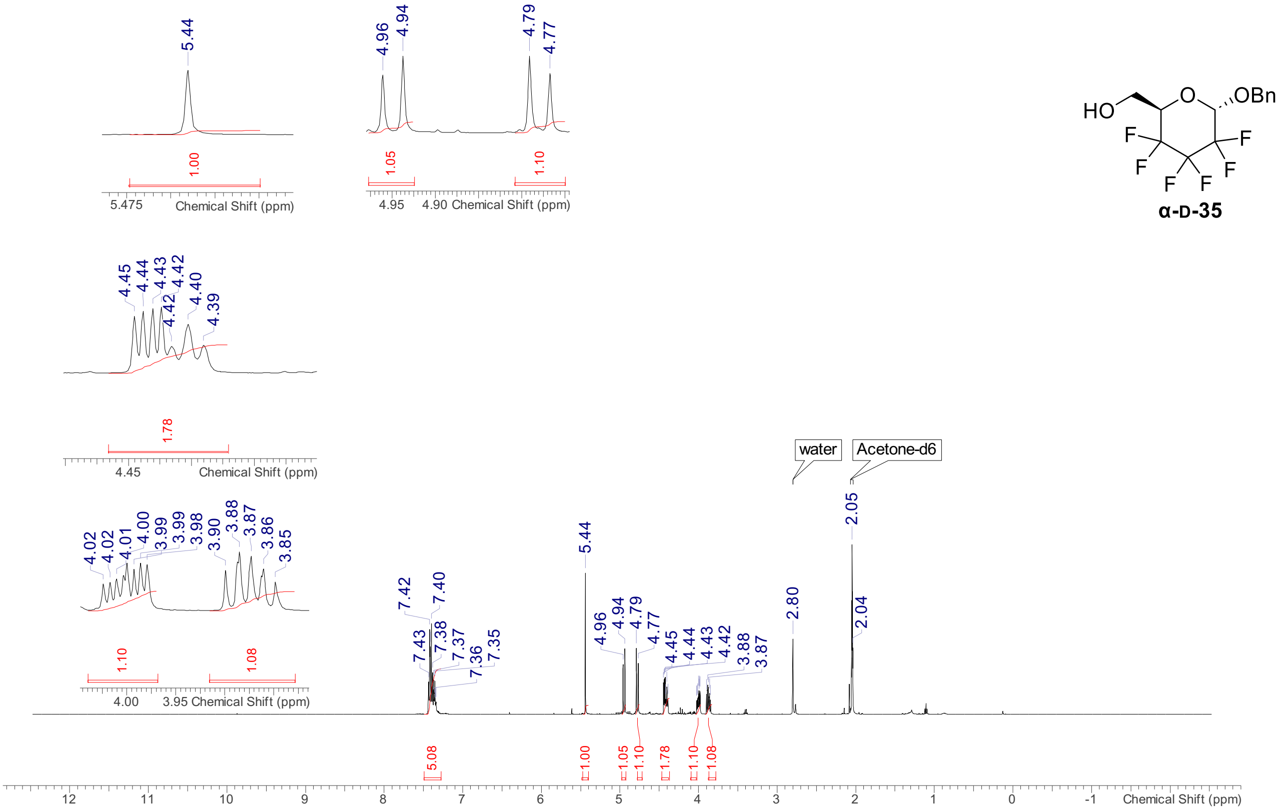

α-D-35

c) ^{19}F NMR (Acetone-d^6, 376 MHz)

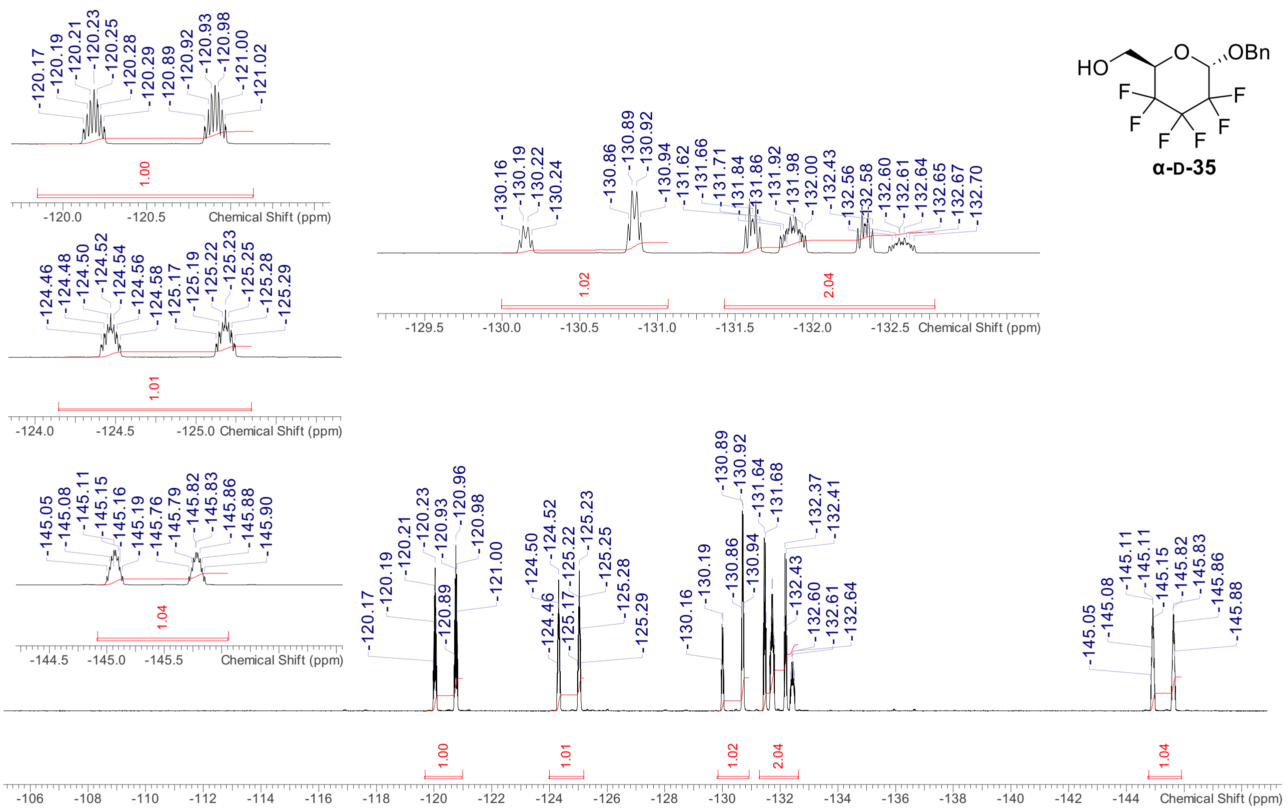

d) ^{19}F{^{1}H} NMR (Acetone-d^6, 471 MHz)

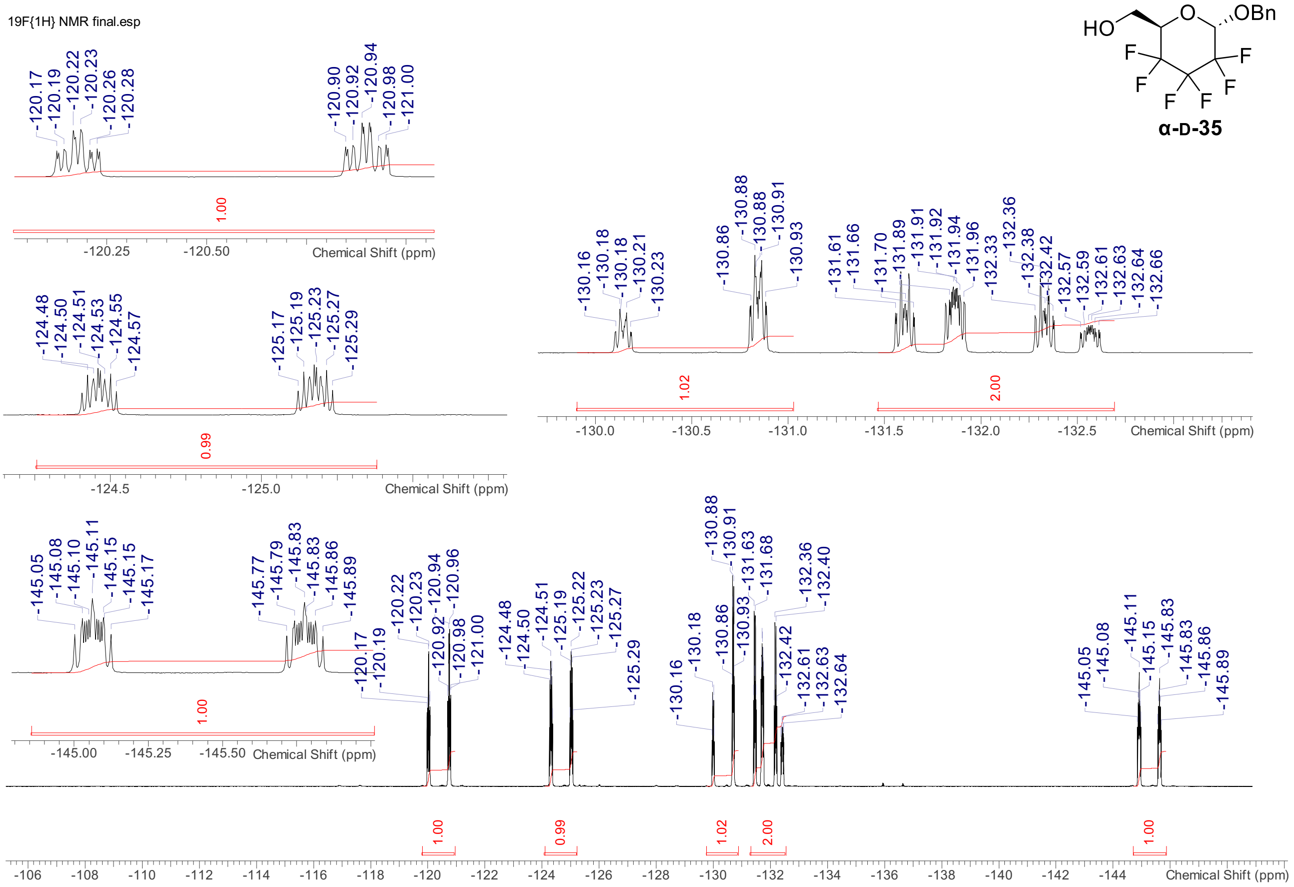

e) $^{13}C\{^{1}H\}$ NMR (Acetone-d^6, 101 MHz)

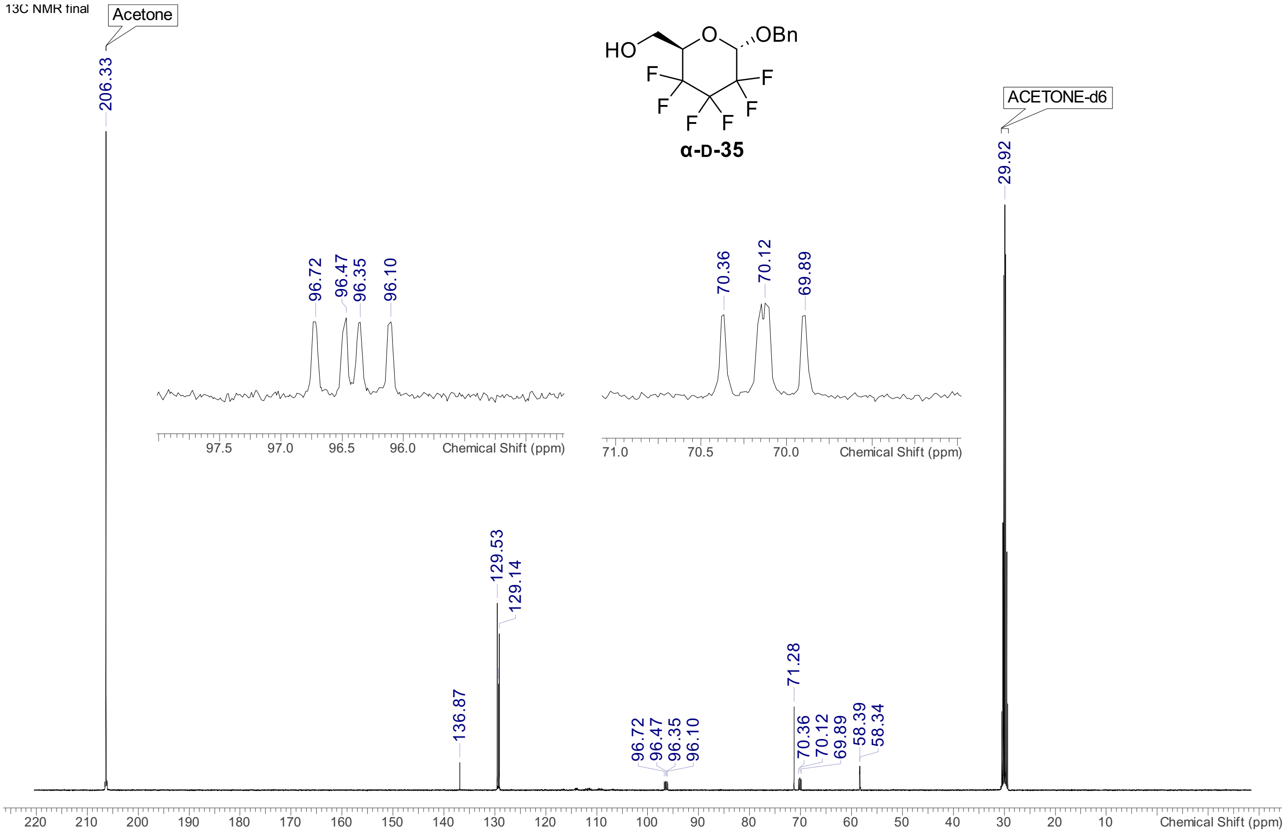

6.18 NMR spectra of 2,3,4-trideoxy-2,2,3,3,4,4-hexafluoro-D-*glycero*-hexopyranose (D-4)

a) ^{1}H NMR (Acetone-d^{6}, 400 MHz)

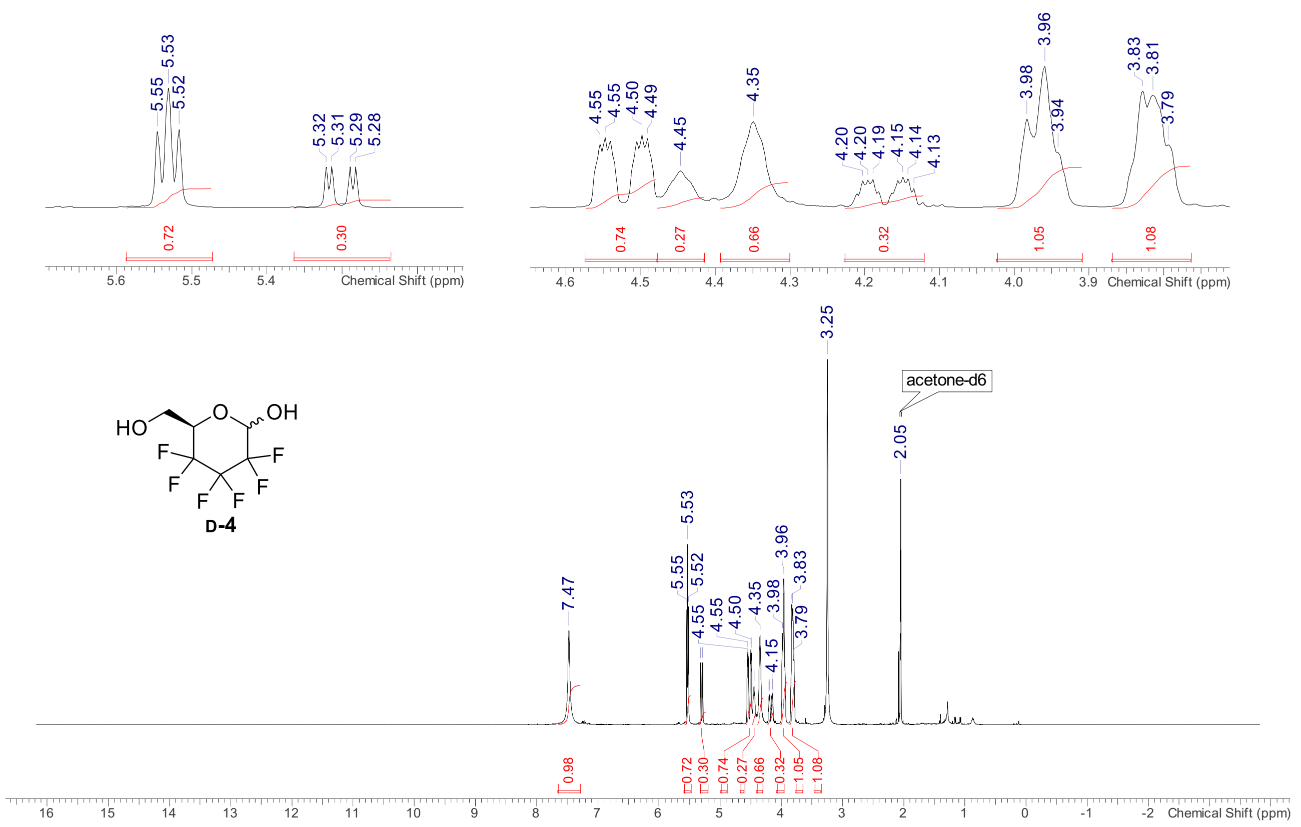

b) ^{1}H {^{19}F} NMR (Acetone-d^{6}, 500 MHz)

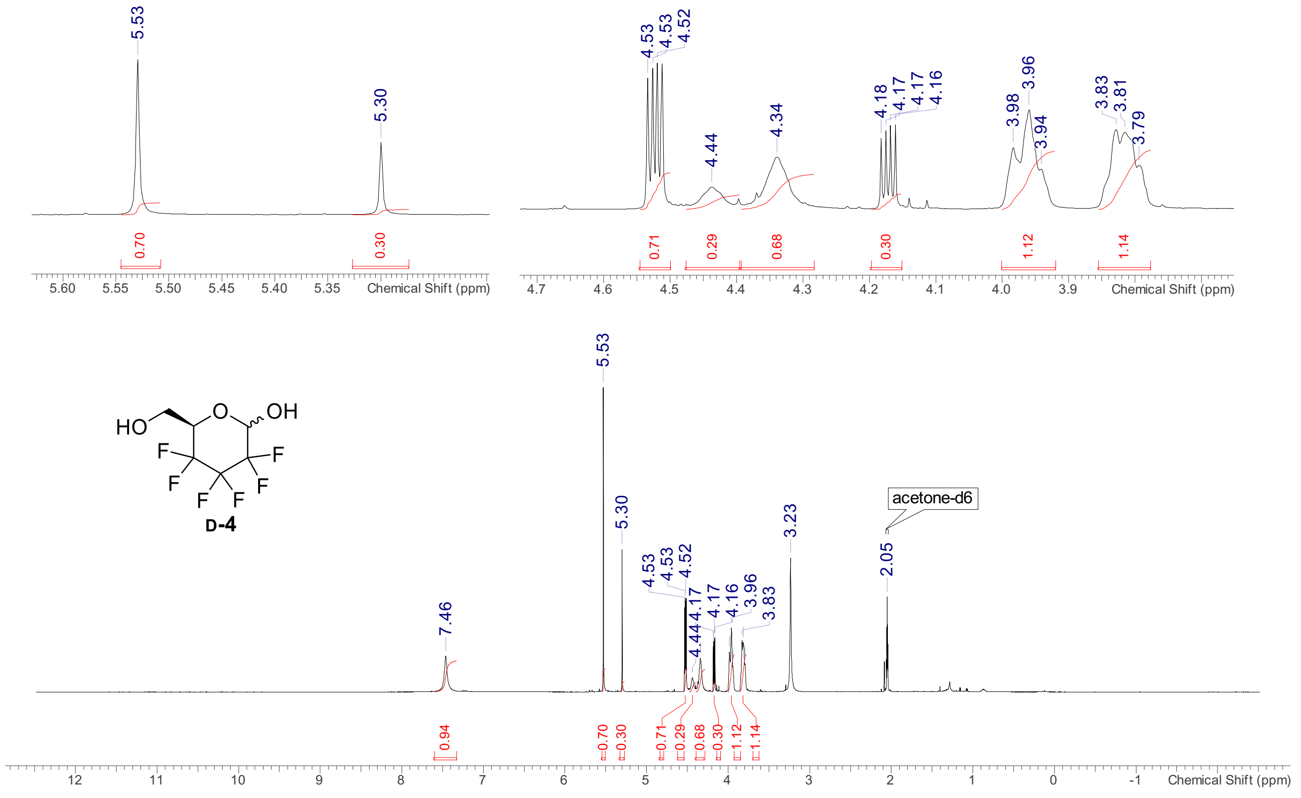

c) ^{19}F NMR (Acetone-d^6, 376 MHz)

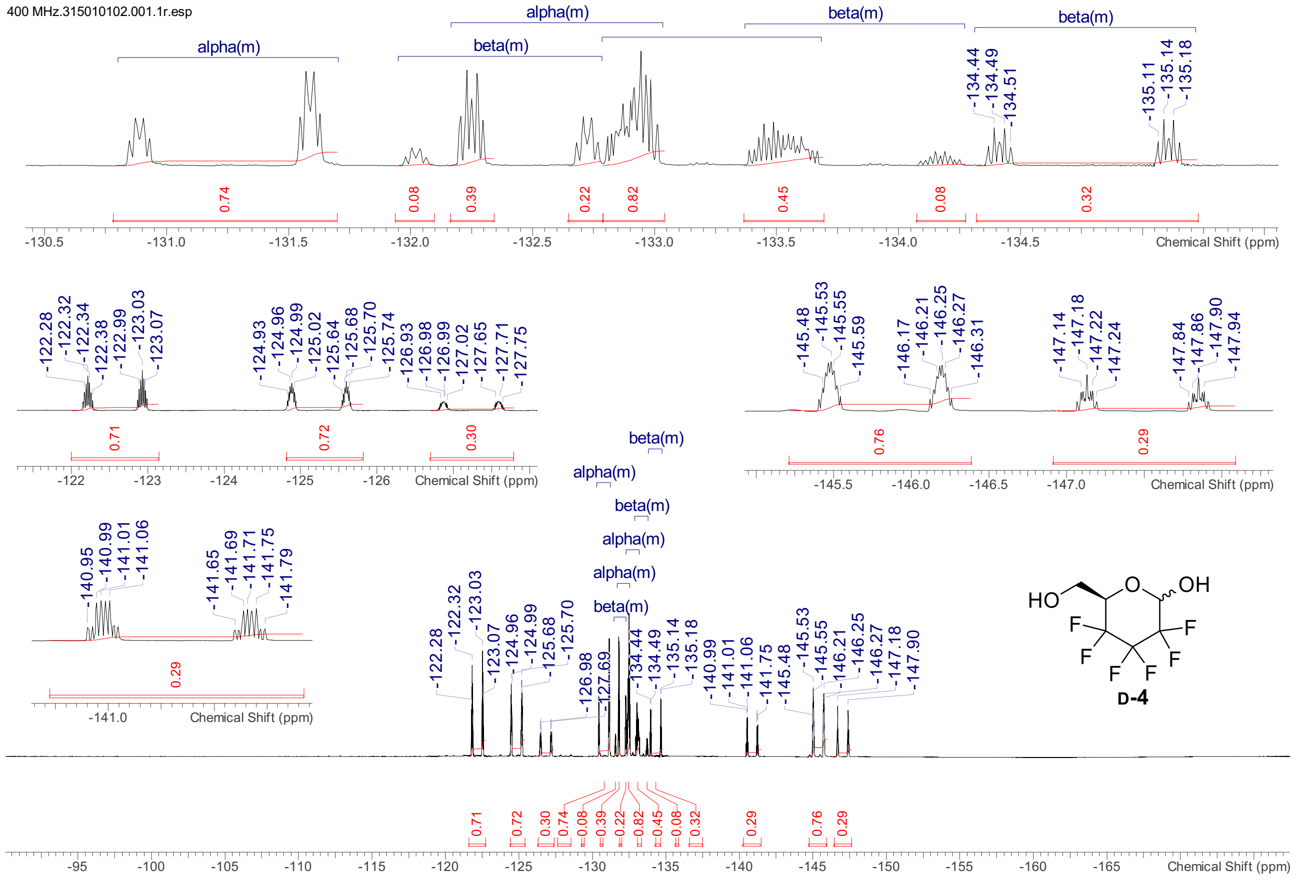

d) ^{19}F{^{1}H} NMR (Acetone-d^6, 471 MHz)

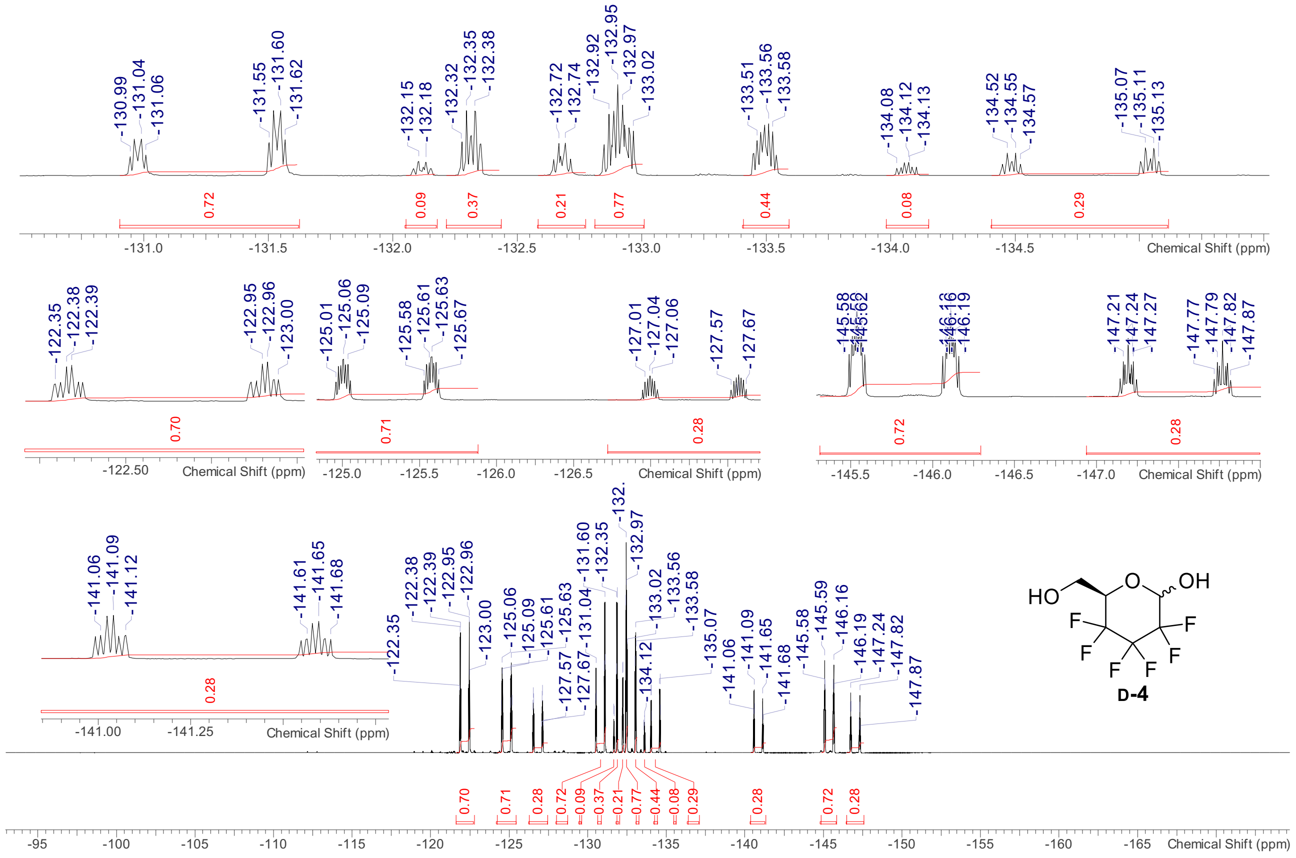

e) $^{13}C\{^{1}H\}$ NMR (Acetone-d^6, 101 MHz)

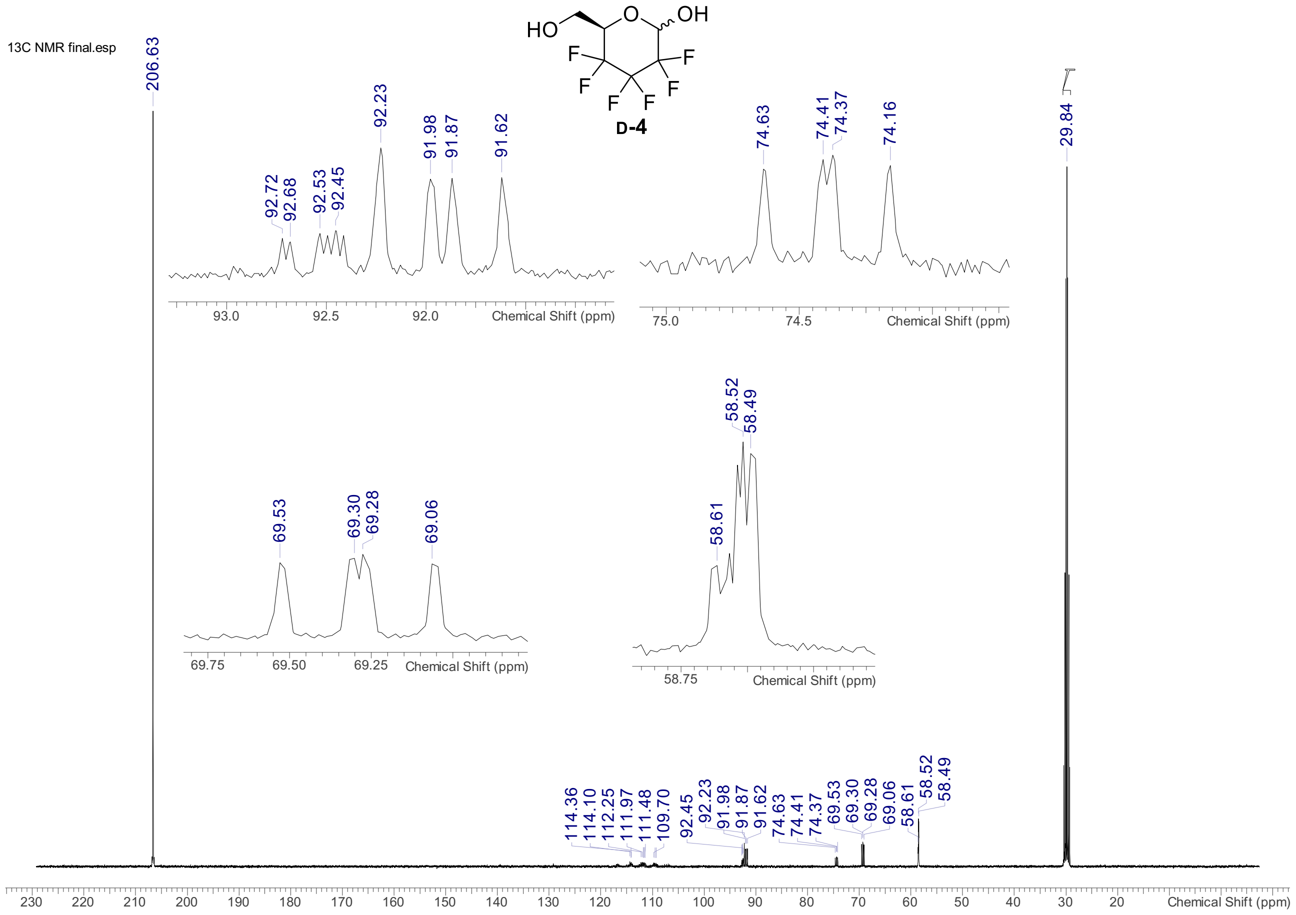

6.19 NMR spectra of 2,3,4-trideoxy-2,2,3,3,4,4-hexafluoro-L-*threo*-heptopyranose (L-18)

a) ^{1}H NMR (Acetone-d^6, 400 MHz)

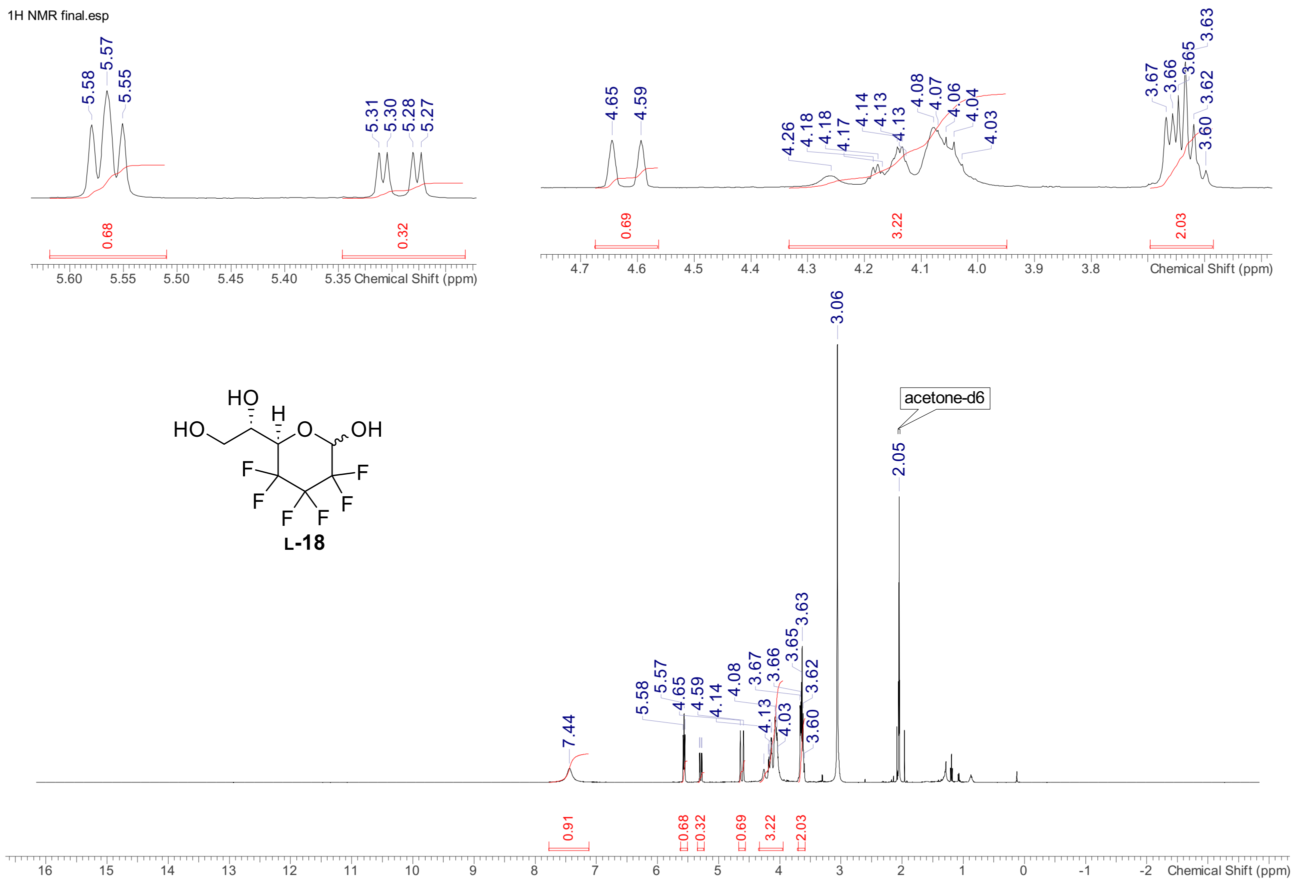

b) ^{1}H {^{19}F} NMR (Acetone-d^6, 500 MHz)

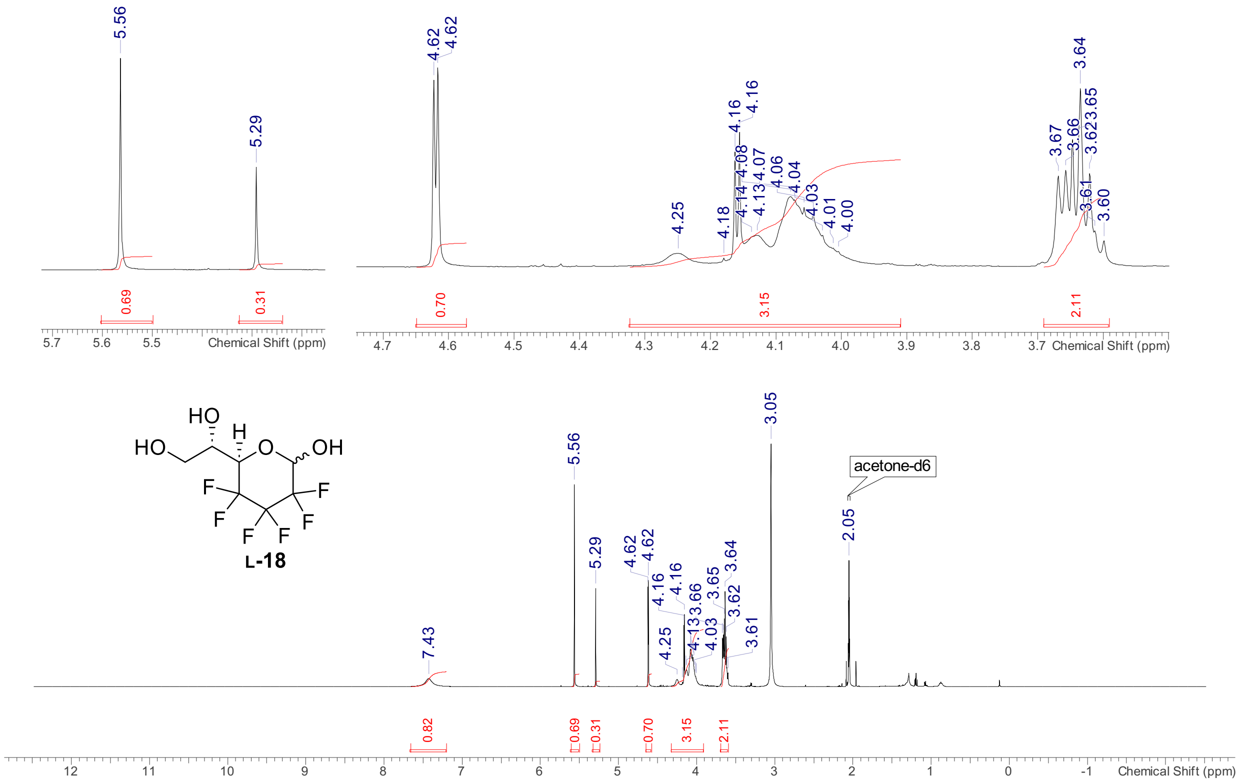

c) ^{19}F NMR (Acetone-d^6, 376 MHz)

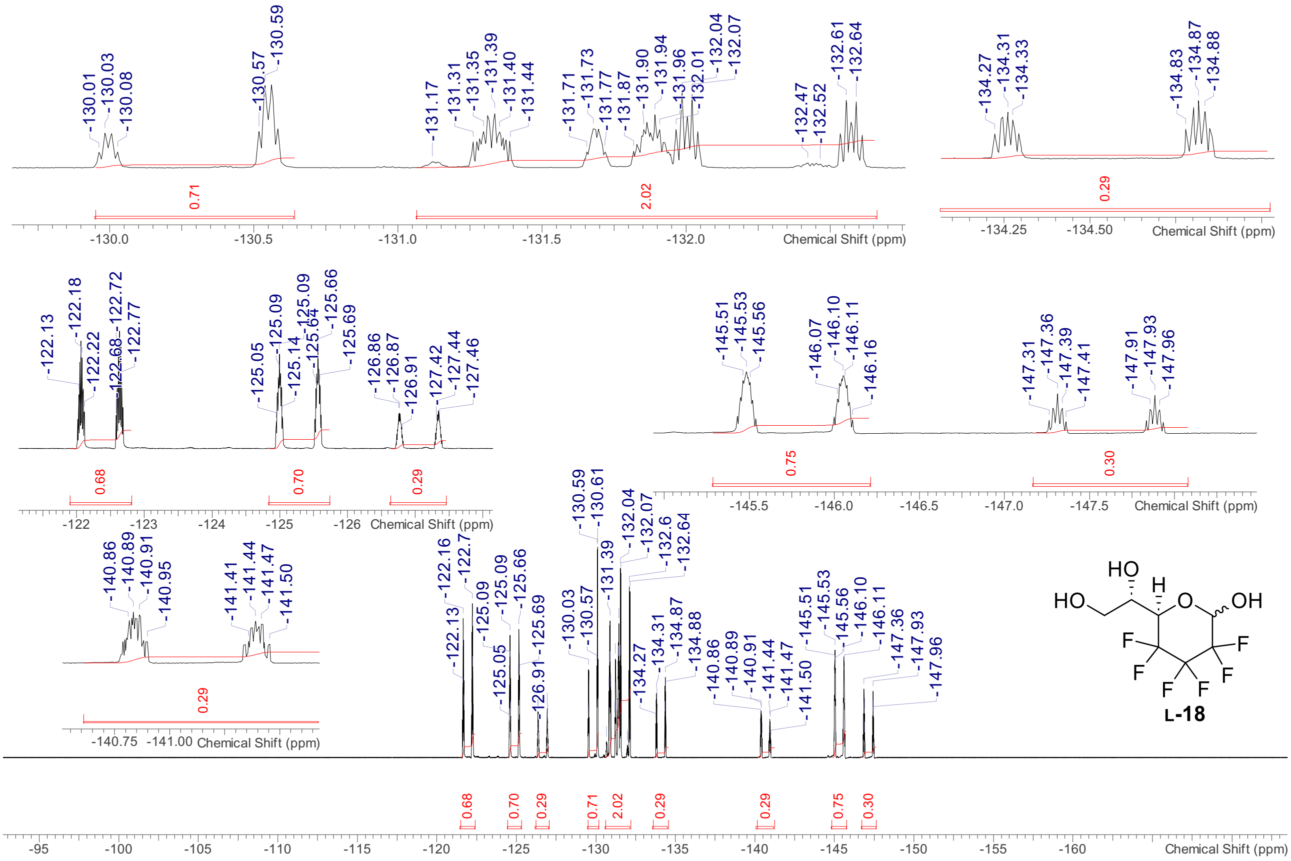

d) ^{19}F{^{1}H} NMR (Acetone-d^6, 471 MHz)

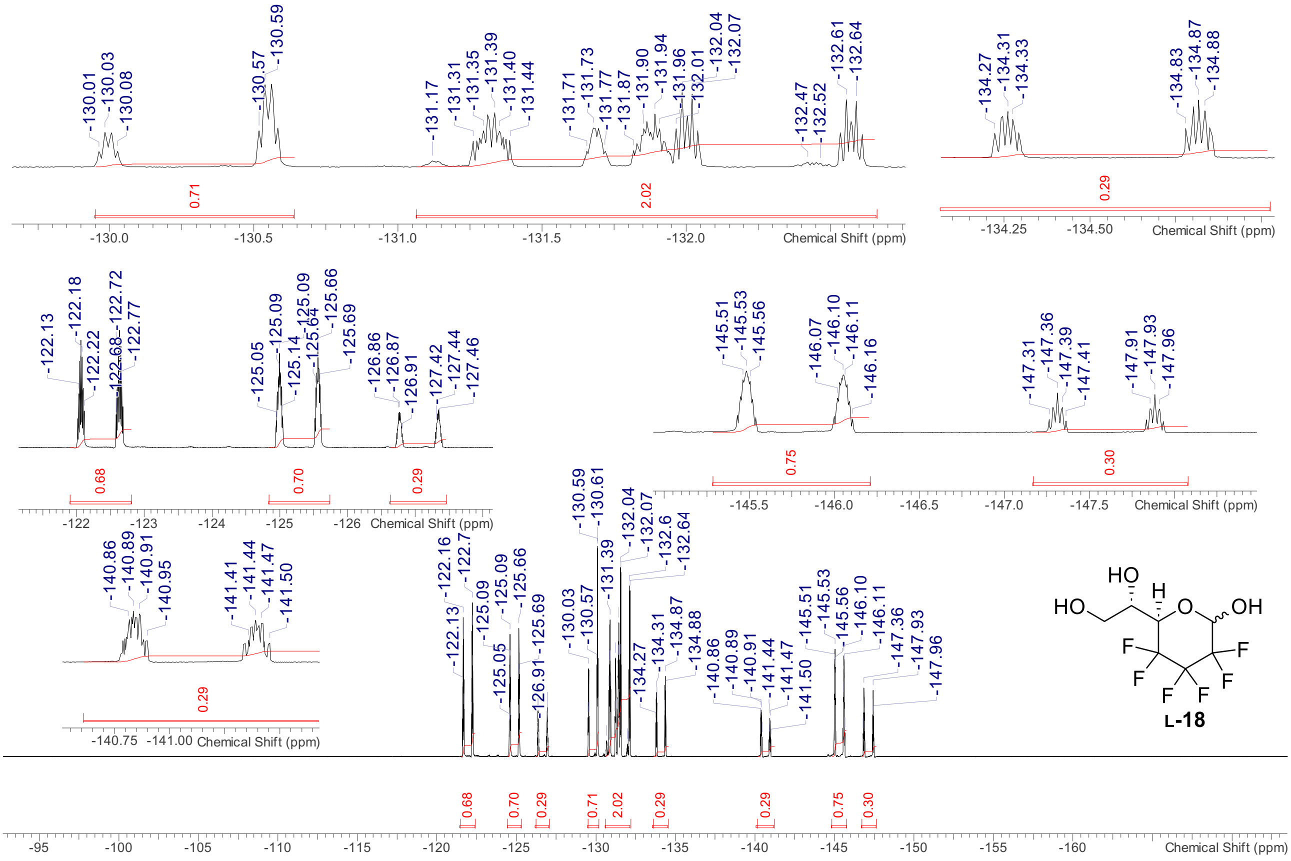

e) $^{13}C\{^{1}H\}$ NMR (Acetone-d^6, 101 MHz)

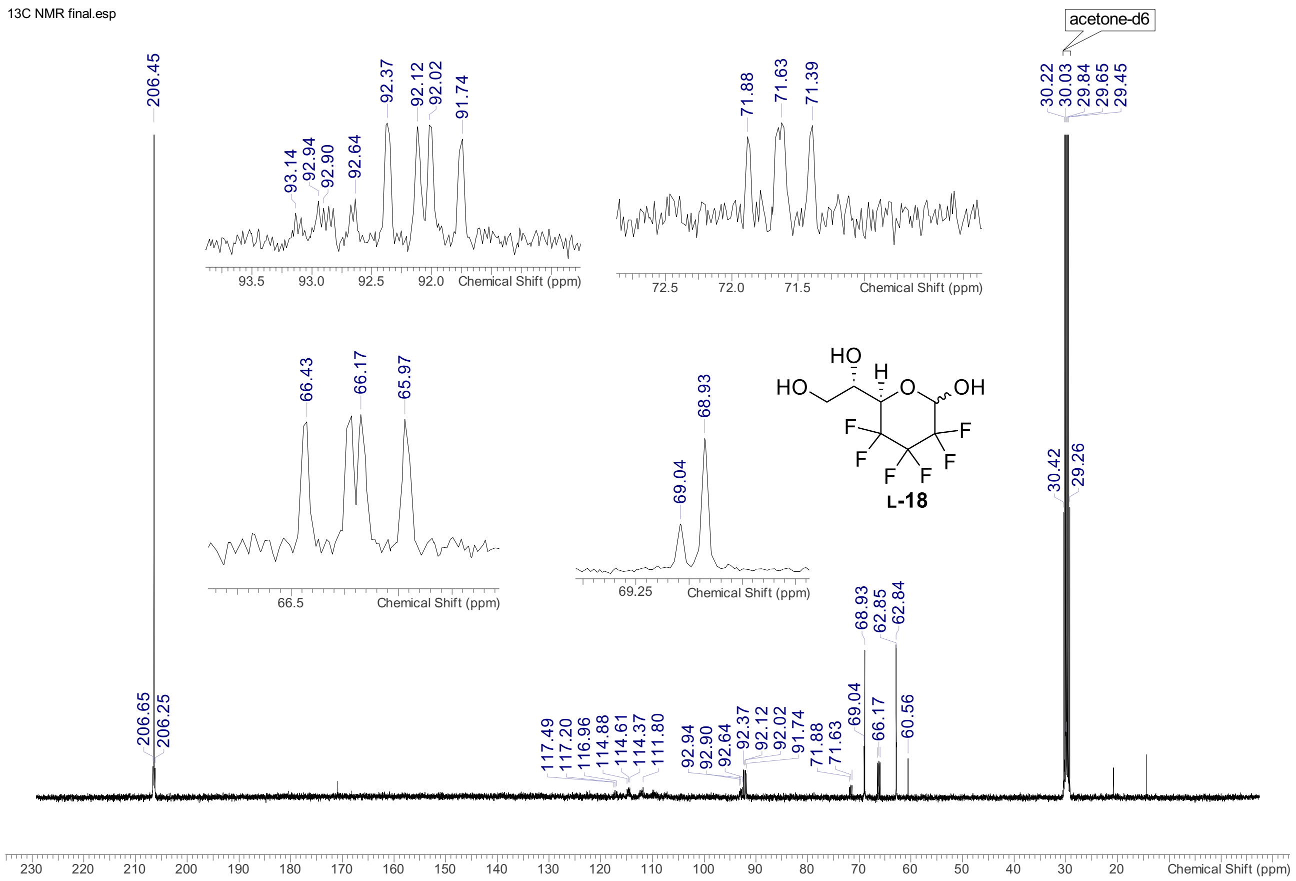

7 Crystallographic data

7.1 (2*S*,3*R*)-1,7-bis(benzyloxy)-4,4,5,5,6,6-hexafluoroheptane-2,3-diol (28)

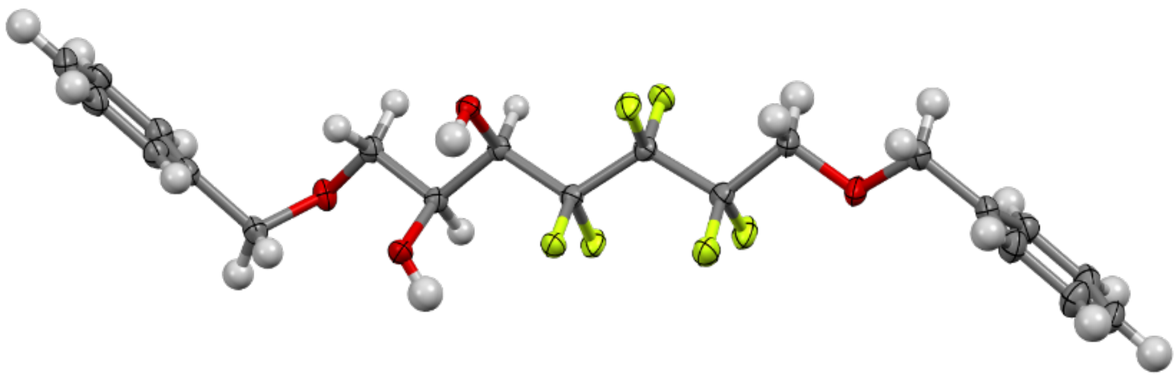

Thermal ellipsoids drawn at the 50% probability level.

Experimental: **28** (1.95 g) was suspended in hexane (40 mL) and heated to 40 °C, followed by addition of Et_2O (5 mL). Slow evaporation at room temperature over 5 days afforded crystalline solids (1.83 g, 94% recovery) that were analysed by X-ray. A suitable crystal was selected and mounted on a LithoLoop on a SuperNova, Dual, Cu at home/near, Atlas diffractometer. The crystal was kept at T = 100(2) K during data collection. Using Olex2 [1], the structure was solved with the SHELXT [2] structure solution program using Intrinsic Phasing and refined with the SHELXL [3] refinement package using Least Squares minimisation.

Crystal data: $C_{21}H_{22}F_6O_4$, *Mr* = 452.39 g/mol, monoclinic, $P2_1$ (No. 4), a = 4.95950(10) Å, b = 35.5289(8) Å, c = 5.8955(2) Å, β = 103.902(3)°, V = 1008.39(5) Å^3, Z = 2, T = 100(2) K, μ(CuKα) = 1.211 mm^{-1}, *Dcalc* = 1.490 g/cm^3, 28625 reflections measured (9.958° ≤ 2Θ ≤ 147.876°), 4033 unique (R_{int} = 0.0425, R_{sigma} = 0.0239) which were used in all calculations. The final R_1 was 0.0296 (I > 2σ(I)) and wR_2 was 0.0705 (all data).

7.2 (2*S*,3*R*)-4,4,5,5,6,6-hexafluoro-1,2-*O*-isopropylideneheptane-3,7-diol (16)

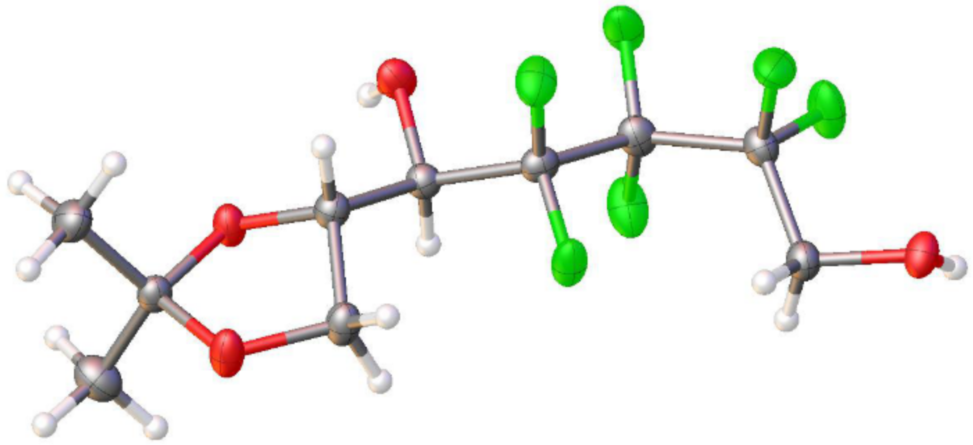

Thermal ellipsoids drawn at the 50% probability level.

Experimental: Single clear colourless prism-shaped crystals of **16** were recrystallised from a mixture of hexane and Et_2O by slow evaporation. A suitable crystal (0.50×0.38×0.35) mm^3 was selected and mounted on a MITIGEN holder silicon oil on a Rigaku R-AXIS Spider diffractometer. The crystal was kept at T = 100(2) K during data collection. Using Olex2 [1], the structure was solved with the SHELXT [2] structure solution program, using the Direct Methods solution method. The model was refined with the SHELXL [3] refinement package using Least Squares minimisation.

Crystal data: $C_{10}H_{14}F_6O_4$, *Mr* = 312.21 g/mol, monoclinic, *P2₁* (No. 4), a = 5.3543(2) Å, b = 14.9816(5) Å, c = 8.1108(3) Å, β = 95.876(7)°, $\alpha = \gamma$ = 90°, V = 647.20(4) Å^3, Z = 2, Z' = 1, T = 100(2) K, μ(CuKα) = 1.580 mm^{-1}, 7561 reflections measured, 2415 unique (R_{int} = 0.0383) which were used in all calculations. The final R_1 was 0.0306 (I > 2σ(I)) and wR_2 was 0.0777 (all data).

7.3 2,3,4-trideoxy-6,7-*O*-isopropylidene-2,2,3,3,4,4-hexafluoro-L-*threo*-heptopyranose (L-31)

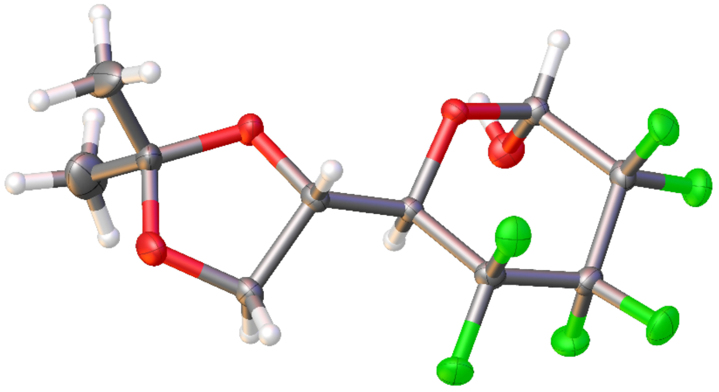

Thermal ellipsoids drawn at the 50% probability level.

Experimental: Single clear colourless prism-shaped crystals of **31** were recrystallised from $CHCl_3$ by slow evaporation. A suitable crystal (0.45×0.31×0.28) mm^3 was selected and mounted on a MITIGEN holder silicon oil on a Rigaku AFC12 FRE-HF diffractometer. The crystal was kept at T = 100(2) K during data collection. Using Olex2 [1], the structure was solved with the SHELXT [2] structure solution program, using the Intrinsic Phasing solution method. The model was refined with the SHELXL [3] refinement package using Least Squares minimisation.

Crystal data: $C_{10}H_{12}F_6O_4$, Mr = 310.20 g/mol, tetragonal, $P4_12_12$ (No. 92), a = 8.97170(10) Å, b = 8.97170(10) Å, c = 31.6723(4) Å, $\alpha = \beta = \gamma = 90°$, V = 2549.35(7) Å^3, Z = 8, Z' = 1, T = 100(2) K, μ(MoKα) = 0.176 mm^{-1}, 49395 reflections measured, 3270 unique (R_{int} = 0.0187) which were used in all calculations. The final R_1 was 0.0264 (I > 2σ(I)) and wR_2 was 0.0695 (all data).

7.4 2,3,4-trideoxy-2,2,3,3,4,4-hexafluoro-D-*glycero*-hexopyranose (D-4)

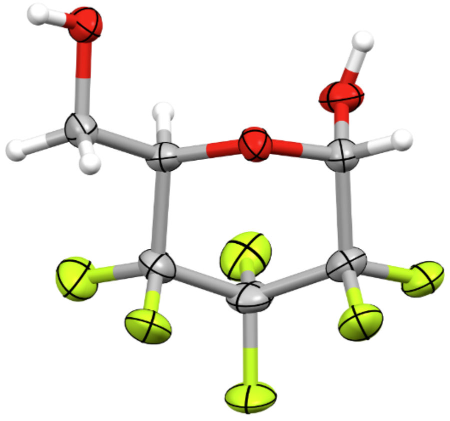

Thermal ellipsoids drawn at the 50% probability level.

Experimental: D-**4** (~40 mg) was dissolved in 1.5 mL of Et_2O. 3 mL of hexane were added and slow evaporation (2 weeks) afforded crystalline solids that were analysed by X-ray. A suitable crystal was selected and mounted on a LithoLoop on a SuperNova, Dual, Cu at home/near, Atlas diffractometer. The crystal was kept at T = 100(2) K during data collection. Using Olex2 [1], the structure was solved with the SHELXT [2] structure solution program using Intrinsic Phasing and refined with the SHELXL [3] refinement package using Least Squares minimisation.

Crystal data: $C_{16}H_{22}F_{12}O_7$, Mr = 554.33 g/mol, monoclinic, I_2 (No. 5), a = 10.0308(7) Å, b = 7.5131(5) Å, c = 29.4870(14) Å, β = 95.237(6)°, V = 2212.9(2) Å^3, Z = 4, T = 100(2) K, μ(CuKα) = 1.727 mm^{-1}, $Dcalc$ = 1.664 g/cm^3, 16481 reflections measured (6.02° ≤ 2Θ ≤ 148.014°), 4362 unique (R_{int} = 0.0657, R_{sigma} = 0.0528) which were used in all calculations. The final R_1 was 0.0514 (I > 2σ(I)) and wR_2 was 0.1362 (all data).

8 References

1. O.V. Dolomanov and L.J. Bourhis and R.J. Gildea and J.A.K. Howard and H. Puschmann, Olex2: A complete structure solution, refinement and analysis program, *J. Appl. Cryst.*, (2009), **42**, 339-341.

2. Sheldrick, G.M., ShelXT-Integrated space-group and crystal-structure determination, *Acta Cryst.*, (2015), **A71**, 3-8.

3. Sheldrick, G.M., Crystal structure refinement with ShelXL, *Acta Cryst.*, (2015), **C27**, 3-8.
